# Prevalence and severity of mental health problems in early-career researchers: a systematic review and meta-analysis

**DOI:** 10.1038/s41562-026-02505-5

**Published:** 2026-06-29

**Authors:** Aljoscha Dreisoerner, Vanessa Goetz, David Frohnmayer, Ulrich S. Tran, Martin Voracek, Urs M. Nater

**Affiliations:** 1 Research Platform: The Stress of Life (SOLE)—Processes and Mechanisms Underlying Everyday Life Stress, University of Vienna, Vienna, Austria; 2 Department of Cognition, Emotion, and Methods in Psychology, Faculty of Psychology, University of Vienna, Vienna, Austria; 3 Department of Clinical and Health Psychology, Faculty of Psychology, University of Vienna, Vienna, Austria

**Keywords:** Education, Human behaviour

## Abstract

Mental health concerns among early-career researchers (ECRs) have gained attention, but prevalence and severity estimates remain fragmented. Here we conducted a preregistered systematic review and multilevel meta-analysis (PROSPERO: CRD42024596813) to quantify psychological distress among ECRs. Scopus, Web of Science, ERIC and PubMed were searched through 28 October 2025, yielding 148 studies and four primary databases (*k* = 228; *N* = 138,446). On the basis of validated case-identification thresholds, 29.9% (95% confidence interval, 26.1–33.9%) of ECRs report elevated psychological distress across all outcomes. Specifically, pooled estimates indicated that 29.8% (26.1–33.8%) reported elevated depressive symptoms, 29.7% (25.7–33.8%) anxiety symptoms, 28.3% (23.6–33.3%) eating disorder symptoms, 22.9% (18.4–27.9%) alcohol misuse, 18.6% (14.4–23.2%) non-suicidal self-injury and 18.8% (15.2–22.6%) suicidal ideation. The prevalence of depressive symptoms was approximately two to three times higher and anxiety symptoms three to five times higher than in age-matched general-population samples. Symptom severity estimates, capturing mean symptom levels on continuous scales, on average were ‘mild’ for depressive and anxiety symptoms and ‘moderate’ for stress. Moderator analyses showed that outcome and scale type accounted for the largest share of variance, whereas demographic and individual characteristics had limited associations with prevalence and severity estimates. These findings underscore the need for structural reforms and implementation of evidence-based support services for ECRs.

## Abstract (web summary)

Mental health concerns among early-career researchers are increasingly gaining attention. This preregistered systematic review and multilevel meta-analysis quantifies psychological distress among early-career researchers, contributing to a better understanding of the prevalence and severity of mental health issues.

## Main

Mental health concerns among early-career researchers (ECRs), including predoctoral (PhD students) and early postdoctoral researchers, have been gaining attention and point towards a potential mental health crisis in academia^1–7^. While ECRs generally report high job satisfaction and intrinsic motivation^8^, recent studies suggest that this population experiences a disproportionate degree of psychological distress, including clinically defined syndromes such as depression, generalized anxiety disorder (GAD) and burnout, or individual symptoms such as stress, poor sleep and suicidal ideation^1,9–11^. ECRs cite a range of structural and individual stressors, such as short-term employment contracts and related job insecurity, high competition, perceived isolation, feeling like an impostor, lack of feedback and power abuse as contributors to poor mental health^12–18^. Doctoral students and postdoctoral researchers share many of these challenges, though some stressors may be more pronounced in doctoral students (for example, supervisor dependency, thesis progress pressure, financial strain and impostor feelings)^2,13,14^ and in postdoctoral researchers (for example, short-term contracts, career uncertainty and pressure to publish or secure funding)^12,19^. These structural and individual stressors are accompanied by several concerning trends. Doctoral attrition remains at alarmingly high rates, with estimates between 30% and 50% of doctoral candidates leaving their programmes before graduating^20–23^, pointing towards wasted research potential and associated mental health issues. A recent meta-analysis on doctoral students and medical residents estimated that 9% reported suicidal ideation in the past year^24^. Population-based studies report lower prevalence in comparable age groups (4–5%)^25–27^, suggesting that suicidal ideation among doctoral students is roughly two times higher than in the general population. In response to these trends, awareness of and interest in mental health issues among ECRs have increased in recent years, prompting some universities, governments and professional organizations to call for reforms and increased support for ECRs^28–30^. However, despite this increase in awareness, the full extent of psychological distress among ECRs is not known, as several important gaps remain open.

Existing meta-analyses estimate that 21–24% of predoctoral researchers exceed screening cut-offs for depressive symptoms and 17–29% for anxiety symptoms. When compared with age-matched groups in the general population, prevalence rates for ECRs are markedly elevated, with population-based studies reporting prevalence rates from 8% to 17% for depressive symptoms and 6% to 10% for anxiety symptoms^31–33^. Average symptom severity typically falls in the mild range^9–11^. However, there is also variability in the severity of symptoms. One meta-analysis of doctoral students reported that 36% experienced mild, 12% moderate and 9% severe depressive symptoms^10^. Another meta-analysis of doctoral students found that 23% experienced mild, 17% moderate and 14% severe anxiety symptoms^34^. Despite the growing literature on this topic^35–42^, most reviews and meta-analyses have focused on depression, anxiety or suicidal ideation, with prevalence and severity estimates limited to depression^10^ and GAD^9^. A broader systematic evaluation of other relevant mental health issues remains lacking, including clinically defined syndromes such as social phobia, obsessive-compulsive disorder (OCD), post-traumatic stress disorder (PTSD), attention deficit hyperactivity disorder (ADHD), personality disorders, eating disorders, alcohol and drug abuse, and burnout, as well as common symptoms of psychological distress such as stress, sleep disturbance, non-suicidal self-injury, suicidal ideation and somatic symptoms. In addition, there has been a surge of research on mental health in ECRs since the COVID-19 pandemic, but no meta-analysis so far has synthesized these more recent studies. For example, the most recent estimates on depression include data up to November 2019^11^. It also remains unknown whether psychological distress among ECRs increased during the pandemic or how it evolved in the post-pandemic period.

The high prevalence of psychological distress among ECRs underscores the need for a systematic investigation of individual- and study-level factors that moderate their prevalence and severity. To date, only two meta-analyses have empirically examined such moderators—and only in relation to depressive^10^ and anxiety symptoms^9^. These studies identified several study-level moderators. Prevalence estimates were generally lower in studies with random sampling and higher study quality, and both prevalence and severity estimates varied depending on which type of measurement scale was used. In contrast, sample-level moderators such as national income level, gender and calendar year showed no consistent effects. Systematic and narrative reviews highlight additional sample-level moderators, including protective and risk factors^35–42^. These include financial challenges^43^, self-doubt^44^, and social and academic loneliness^14,45^ as risk factors, and suggest social support, a positive and future-oriented mindset, and self-care^14^ as potential protective factors. Supervisor relationships have been described as either a risk or protective factor depending on their quality^14,46^. Despite these insights, many potentially important sample-level moderators, including demographic characteristics (for example, age and ethnicity), contextual variables (such as country, academic discipline and career stage) and individual factors (such as pre-existing mental health diagnoses and life or job satisfaction) have yet to be systematically assessed across the broader spectrum of mental health outcomes beyond depression and anxiety. Evaluating these moderators is critical for understanding psychological distress in ECRs and for developing evidence-based interventions.

To summarize the available evidence on a wide range of mental health issues among ECRs and to identify factors that modulate this burden, we conducted a systematic review and multilevel meta-analysis^47^ that quantifies the prevalence and severity of a broad range of mental health syndromes and symptoms included under the umbrella term ‘psychological distress’. Our analysis includes both predoctoral researchers actively pursuing a PhD and postdoctoral researchers and provides pooled estimates of prevalence and severity across a spectrum of conditions (for example, depressive symptoms, anxiety symptoms, social phobia, OCD, PTSD, ADHD, personality disorders, eating disorders, alcohol and drug abuse, and burnout) and individual symptoms (for example, stress, sleep disturbance, non-suicidal self-injury, suicidal ideation and somatic symptoms). We also systematically evaluated the moderating roles of both individual-level factors (gender, age, career stage, country, ethnicity, academic discipline, pre-existing mental health diagnosis, job satisfaction, life satisfaction, supervisor relationship quality, social support and loneliness) and study-level characteristics (study quality, sampling method and utilized measurement scale).

## Results

### Study identification and selection

**Fig. 1:**
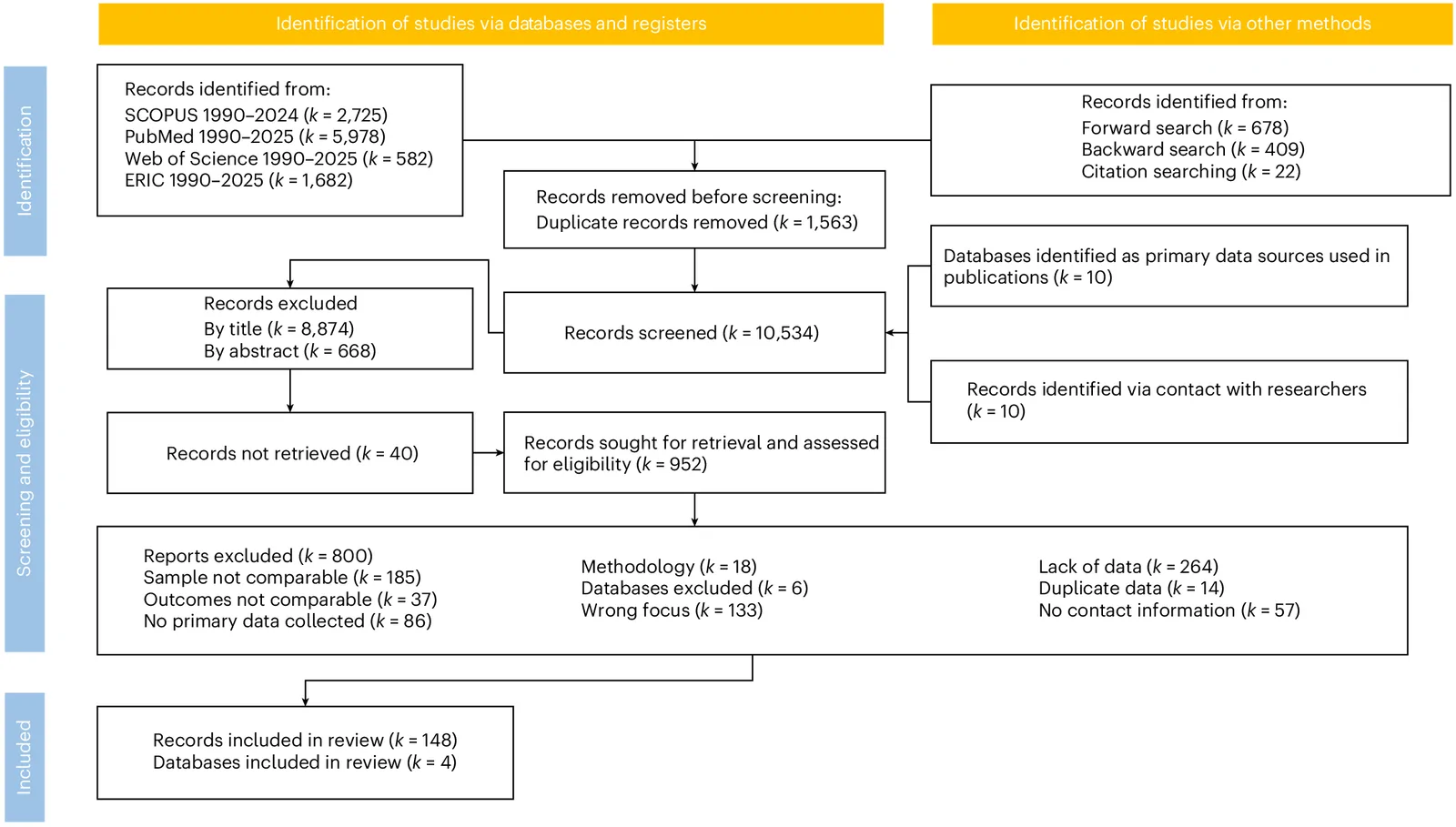
PRISMA 2020 flow diagram. PRISMA 2020 flow diagram of the literature search and the screening of records.

The PRISMA 2020 flow diagram shows the study identification and selection process for the meta-analysis (Fig. 1). Of 10,534 screened records, 148 records and four databases qualified for inclusion, yielding a total of 228 samples. For the analysis, we differentiated the Healthy Minds Network into 15 unique datasets, yielding a total of 166 included records and datasets. More information on the respective samples, their characteristics and references can be found in Supplementary Table 1.

### Characteristics of the studies

The final sample for meta-analysis consisted of 138,446 individuals (134,572 doctoral, 3,522 postdoctoral; pre- or postdoctoral status not reported for 352 ECRs). Most of the data were collected between 2018 and 2024, with a peak in 2019, and most studies were published in 2024 (Supplementary Figs. 1 and 2). The overall sample was 63.3% female, with a mean age of 30.5 years (s.d. = 4.3). Studies that reported on ethnicity had predominantly white (61.9%), Asian (20.8%), Black (8.8%) and Latin (8.4%) samples. Doctoral fields were grouped according to broader categories of academic disciplines (Supplementary Table 2), with social sciences representing the largest share (32.2%), followed by natural sciences (26.9%), applied sciences (26.7%), humanities (10.9%) and formal sciences (3.2%). Within social sciences, psychology accounted for 24.6% of samples. Among applied sciences, medicine represented the second-biggest category (12.6%), followed by engineering (5.6%), communication science (5.9%) and law (2.0%).

Countries were exploratorily clustered on the basis of similar working conditions for ECRs, using large language models (LLMs) (see Supplementary Material 1 and Supplementary Tables 3–7 for details). Most samples (114 samples, 50.0%) originated from countries with flexible, grant-dependent research careers (Cluster 1), such as the USA (*k* = 88) and the United Kingdom (*k* = 13). The second-largest cluster (Cluster 2, *k* = 43, 18.8%) included countries with high government support, structured contracts and career security (Cluster 2), such as France (*k* = 13), Germany (*k* = 11) and the Netherlands (*k* = 7). Cluster 3 (*k* = 31, 13.6%) included countries with self-funded or low-funding systems, such as Italy (*k* = 8) and Spain (*k* = 7). Cluster 4 (*k* = 23, 10.1%) included countries with emerging research systems with variable funding sources and career structures, such as China (*k* = 8) and Brazil (*k* = 6). Cluster 5 (*k* = 14, 6.1%) served as a residual category for countries that did not fit any of the other categories. Cluster 6 (*k* = 3, 1.3%) was characterized by competitive fellowships with limited benefits and short-term contracts, including Japan (*k* = 1) and Hong Kong (*k* = 2).

Samples provided data on depression (*k* = 166), GAD (*k* = 121), stress (*k* = 69), suicidal ideation (*k* = 49), psychological distress in general (*k* = 39), burnout, eating disorders (*k* = 28 each), non-suicidal self-injury (*k* = 23), alcohol abuse (*k* = 24), sleep quality (*k* = 9), somatic symptoms (*k* = 4), drug abuse (*k* = 4), ADHD, OCD, bipolar disorder (*k* = 2 each), social phobia, specific phobia, agoraphobia, panic disorder, PTSD and personality disorders (*k* = 1 each). Analyses on prevalence and severity were based on overlapping but not identical sets of studies, and consequently, their study characteristics also differed. A detailed comparison of the study characteristics between the two study sets and a breakdown of available effect sizes are provided in Supplementary Tables 8 and 9. Some of the syndromes and symptom domains were also dominated by the data of two identified databases (see Supplementary Table 10 for details). In addition, reporting on sample-level demographics and other moderators was inconsistent among the studies included in the prevalence and severity models, with missing data in variables such as gender, age, ethnicity and academic discipline, ranging from 5% to 58% (Supplementary Table 11).

### Risk of bias

Risk-of-bias assessment was based on nine criteria from the Joanna Briggs Institute (JBI) checklist: sample frame, sampling, sample size, study description, data analysis, validity of methods, measuring of condition, statistical analysis and response rate^48^. Overall study quality was low to moderate and varied substantially between samples (*M* = 4.6; s.d. = 1.2; range, 2.5–7.2; Supplementary Table 13 and Supplementary Fig. 3). This may partly be because in many studies ECRs were not the focal group of interest, but rather graduate students or university staff in general. Only 57.9% of studies had sufficient sample sizes (*k* = 132). Only one study used external assessments to measure psychological distress^49^, and random sampling was used rarely (for exceptions, see refs. ^50–58^). Also, only a fraction (*k* = 23; 10.1%) of studies reported an adequate survey response rate (≥45%), and prevalence estimates were almost never accompanied by confidence intervals (see Supplementary Table 12 for details).

### Main results

#### Pooled prevalence and severity estimates

Across all outcomes, nearly one in three ECRs reported elevated psychological distress (pooled prevalence, 29.9%; 95% confidence interval (CI), 26.1–33.9%; *k* = 86). Symptom severity across all outcomes was 33.9 (95% CI, 31.7–36.1; *k* = 151) on a normalized scale from 0 to 100, using per cent of maximum possible (POMP) scores (see Methods for the details). Heterogeneity was high (amounts of heterogeneity within and between samples were roughly equal for severity estimates; for prevalences, heterogeneity was higher between than within samples; Supplementary Tables 14 and 15), with 95% prediction intervals ranging from 0.2% to 71.1% for prevalence and 0.8% to 66.9% for severity estimates. This indicated wide ranges for the expected results of future individual studies. Forest and caterpillar plots are available in Supplementary Figs. 4–12.

#### Prevalence and severity estimates from the PHQ-9, item 9 of the PHQ-9 and the GAD-7

The pooled prevalence of depressive symptoms in the most used scale, the Patient Health Questionnaire (PHQ-9 and PHQ-8), was 28.5% (95% CI, 24.3–32.9), and severity estimates indicated that 33.1% (95% CI, 30.1–35.5) of ECRs reported mild symptoms, 17.0% (95% CI, 15.2–18.9) moderate, 8.1% (95% CI, 6.8–9.5) moderately severe and 4.7% (95% CI, 3.7–5.9) severe depressive symptoms. The pooled prevalence of suicidal ideation (item 9 of the PHQ-9) was 17.4% (95% CI, 13.8–21.4). The pooled prevalence of anxiety symptoms in the most used scale, the GAD-7, was 27.6% (95% CI, 23.4–31.9), with 30.9% (95% CI, 28.4–33.4) reporting mild, 16.0% (95% CI, 14.1–18.0) moderate and 9.7% (95% CI, 8.2–11.4) severe anxiety.

Post-hoc sensitivity analyses showed that the pooled prevalence of depression in the PHQ-9 was slightly higher (by 0.37%; 95% CI, 0.07–0.90; explaining *R*^2^ = 5.7% of the true effect-size variance) than in all other scales measuring depression, while the pooled severity of anxiety in the GAD-7 was slightly higher (by 7.27 POMP scores; 95% CI, 3.70–10.84; *R*^2^ = 2.6%) than in all other scales measuring anxiety (see Supplementary Table 16 for the full details).

### Moderators

Moderators that remained statistically significant after correcting for a false discovery rate (FDR) of 5% are reported in Table 1 and in the text. The full results are provided in Supplementary Tables 18 and 19.

**Table 1:** Significant moderators of prevalence and severity

				95% CI					
Moderator	*k*	#ES	*β*	LL	UL	*F*(d.f._1_,d.f._2_)	Adjusted *P*	*R*^2^	*Q*(d.f.)
**Prevalence**									
Syndrome or symptom domain	86	352				11.41 (18, 333)	0.007	21.24%	21,621.66 (333)***
Utilized scale	86	352				9.65 (49, 302)	0.007	34.39%	10,995.34 (302)***
Age (mean-centred)	61	296	−0.01*	−0.03	−0.00	5.20 (1, 294)	0.048	7.55%	30,333.33 (294)***
US sample						3.83 (1, 350)	0.015	5.12%	26,630.25 (350)***
US sample	41	224	26.03***	21.01	31.39				
Non-US sample	47	129	0.66	−0.00	2.63				
Mental health diagnosis	18	135	0.002**	0.000	0.006	7.45 (1, 133)	0.016	23.46%	11,660.50 (133)***
Loneliness	11	78	−0.002*	−0.011	−0.000	4.13 (1, 76)	0.015	18.75%	8,413.85 (76)***
COVID-19						6.49 (2, 349)	0.010	8.66%	29,012.68 (349)***
Before COVID-19	38	150	24.29***	19.86	29.03				
During COVID-19	39	138	1.19***	0.24	2.85				
After COVID-19	15	65	0.99*	0.00	3.66				
Year						5.41 (2, 349)	0.015	6.14%	28,902.31 (349)***
Year (mean-centred)	86	352	0.000	−0.115	0.115				
Year^2^	86	352	0.000	−0.000	0.001				
Overall study quality	86	352	−0.14**	−0.40	−0.01	7.56 (1, 350)	0.016	11.65%	27,645.71 (350)***
Sampling strategy						11.36 (1, 350)	0.007	9.45%	27,227.51 (350)***
Convenience sampling	69	209	33.33***	29.07	37.74				
Random or full sampling	18	144	−2.61***	−6.48	−0.46				
**Severity**									
Syndrome or symptom domain	151	536				31.27 (14, 521)	0.005	48.67%	250,198.24 (521)***
Utilized scale	151	536				10.15(97, 438)	0.005	53.47%	95,277.32 (438)***
US sample						3.31(1, 534)	0.031	2.48%	849,127.79 (534)***
US sample	89	278	31.58***	28.28	34.87				
Non-US sample	65	258	4.00	−0.32	8.32				
Overall study quality	151	536	−3.06***	−4.65	−1.46	14.14 (1, 534)	0.005	7.69%	842,444.53 (534)***
Sampling strategy						41.28 (1, 534)	0.005	15.31%	863,425.95 (534)***
Convenience sampling	134	410	36.36***	34.25	38.49				
Random or full sampling	19	126	−17.72***	−23.13	−12.29				

**Fig. 2:**
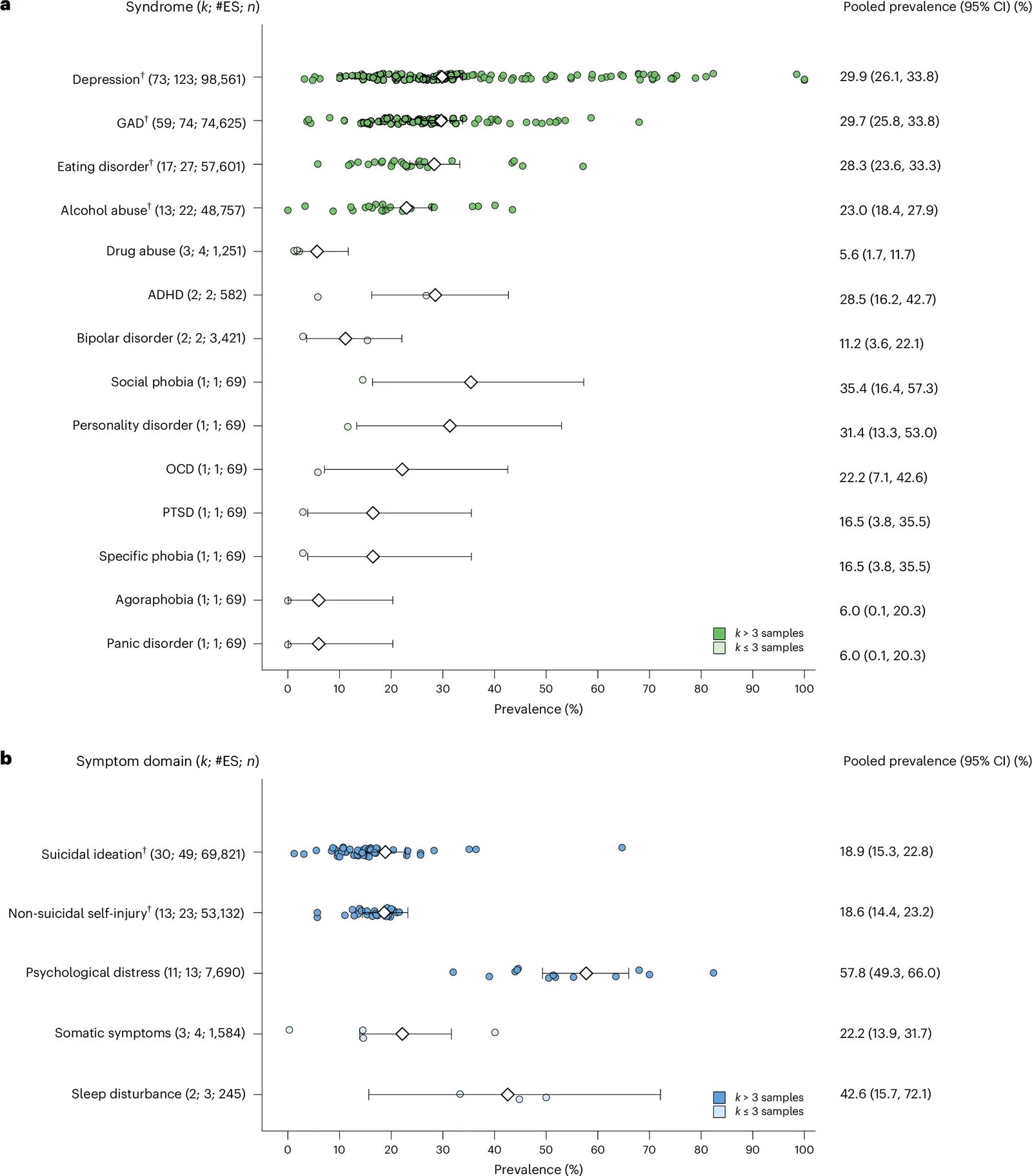
Prevalence rates by syndrome and symptom domain. **a**,**b**, Pooled prevalence estimates for psychiatric syndromes (**a**) and symptom domains (**b**). The diamonds represent pooled prevalence estimates, expressed as percentages, from the multilevel random-effects meta-analysis, based on validated screening thresholds. The pooled prevalence estimates were single-arcsine-transformed and back-transformed. The error bars indicate 95% CIs around those pooled prevalence estimates. Because the pooled prevalences are model-based estimates rather than simple averages of observed sample prevalences, they may differ from the observed prevalence values, particularly for syndromes or symptom domains based on few effect sizes. The model included random effects at the study and effect-size levels. Inference was based on *t*-distributions. All tests were two-sided, and no adjustment for multiple comparisons was applied. Psychological distress refers to information on overall mental health and psychological distress that was reported in some studies. Pooled prevalences for all syndromes and symptom domains were significant at *P* < 0.001, except for panic disorder and agoraphobia at *P* < .0.05. *n* indicates the cumulative sample size. The daggers indicate pooled prevalence estimates that stemmed predominantly from the Healthy Minds Network^52^: alcohol abuse 27.5%, depression 61.9%, GAD 76.19%, eating disorder 90.9%, suicidal ideation 87.3% and non-suicidal self-injury 98.1%.

**Fig. 3:**
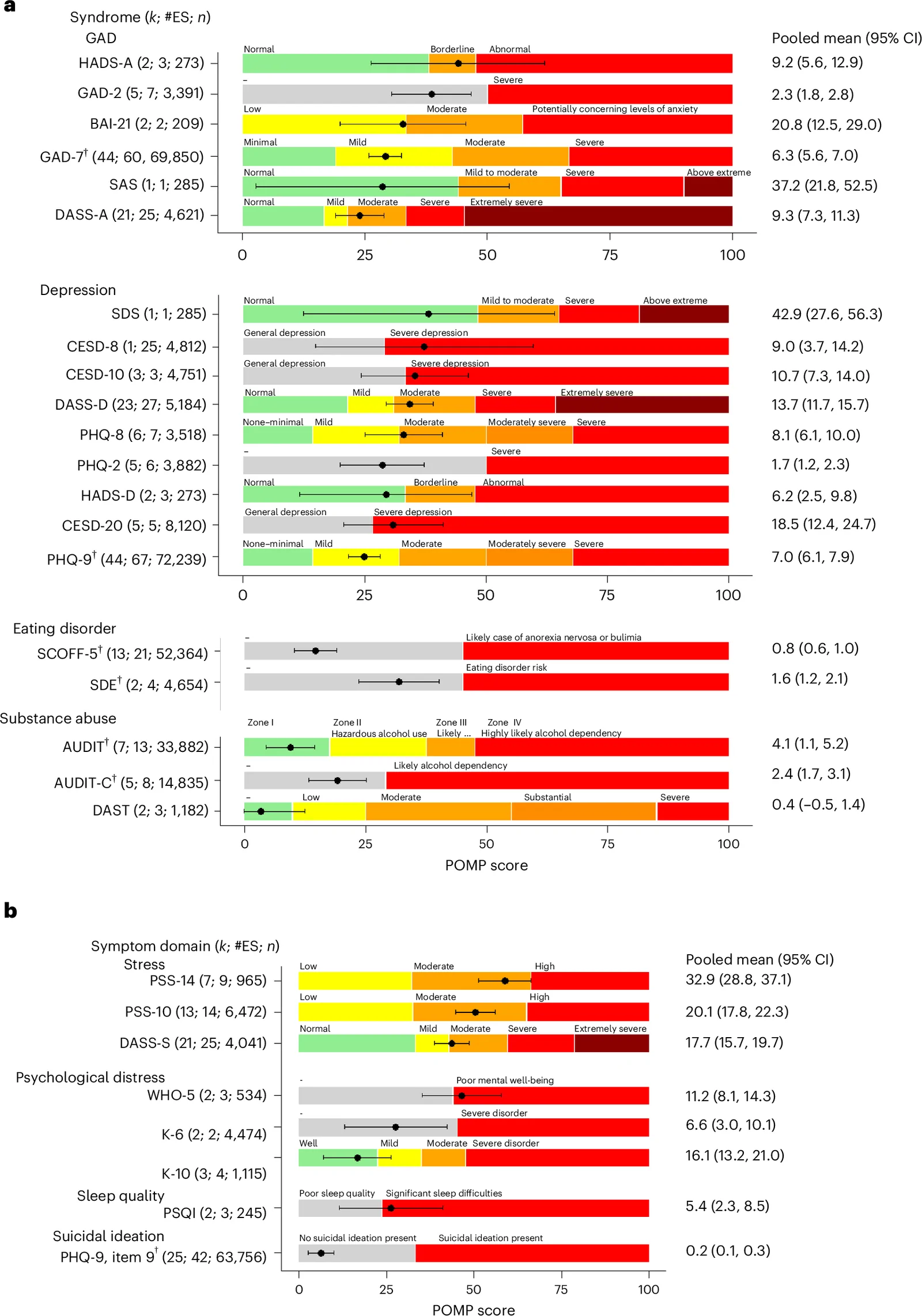
Severity levels for the most frequently used scales per syndrome and symptom domain. **a**,**b**, Dots indicate pooled severity estimates for each scale per syndrome (**a**) and syndrome domain (**b**), displayed on a normalized (POMP) scale ranging from 0 to 100, to allow comparisons across measurement scales, with bars representing 95% CIs. The coloured bands indicate the severity categories derived from the original measurement scales. The pooled severity estimates per measurement scale including 95% CIs are also shown on the right. The model included random effects at the study and effect-size levels. Inference was based on *t*-distributions. All tests were two-sided, and no adjustment for multiple comparisons was applied. Pooled estimates for all the scales were significant at *P* < 0.001, except for the DAST, which did not differ significantly from zero. Estimates pertain to the most frequently used scales for each syndrome in cases where several alternative scales were encountered in primary studies. The WHO-5 has been reverse-scored to enable interpretation in the same direction as the K-6 and K-10, such that higher scores corresponded to higher severity of psychological distress. For several instruments, much of the data stemmed from the Healthy Minds Network datasets (indicated by daggers): AUDIT-C 90.4%, GAD-7 82.0%, PHQ-9 84.7%, PHQ-9 item 9 96.2%, SCOFF-5 and SDE combined 100%. *n* indicates the cumulative sample included in the analysis per moderator. HADS-A, Hospital Anxiety and Depression scale, anxiety subscale; GAD-2, Generalized Anxiety Disorder, 2-item version; DASS-A, Depression, Anxiety and Stress scale, 21- and 42-item version, anxiety subscale; GAD-7, Generalized Anxiety Disorder, 7-item version; SAS, Zung Self-Rating Anxiety Scale; BAI-21, Beck anxiety inventory; SDS, Zung Self-Rating Depression Scale; CESD-8, Center for Epidemiological Studies Depression scale, 8-item version; CESD-10, Center for Epidemiological Studies Depression scale, 10-item version; DASS-D, Depression, Anxiety and Stress scale, 21- and 42-item version, depression subscale; PHQ-8, Patient Health Questionnaire, 8-item version; PHQ-2, Patient Health Questionnaire, 2-item version; HADS-D, Hospital Anxiety and Depression scale, depression subscale; CESD-20, Center for Epidemiological Studies Depression scale, 20-item version; PHQ-9, Patient Health Questionnaire, 9-item version; SCOFF-5, Sick, Control, One, Fat, Food Questionnaire, 5-item version; AUDIT, Alcohol Use Disorder Identification Test, 10-item version; AUDIT-C, Alcohol Use Disorder Identification Test—Consumption; DAST, Drug Abuse Screening Test, 10-item version; PSS-14, Perceived Stress Scale, 14-item version; PSS-10, Perceived Stress Scale, 10-item version; DASS-S, Depression, Anxiety and Stress Scale, 21- and 42-item version, stress subscale; WHO-5, World Health Organization well-being index, 5-item version; K-6, Kessler Psychological Distress Scale, 6-item version; K-10, Kessler Psychological Distress Scale, 10-item version; PSQI, Pittsburgh Sleep Quality Index.

Only moderators that remained significant after controlling for the FDR are presented. For categorical moderators, baseline categories are provided in the first line, and estimates of the other categories are deviances from the respective baseline categories. For syndrome or symptom domain and utilized scale, pooled estimates are provided in Figs. 2 and 3. Prevalence data were back-transformed into percentages. *k* indicates the number of studies, *β* is the pooled effect size estimate, adjusted *P* is the FDR-adjusted *P* value and *R*^2^ indicates the amount of attributable true effect-size variance. #ES, number of effect sizes; LL, lower limit; UL, upper limit.

*P < 0.05; **P < 0.01; ***P < 0.001.

#### Syndromes and symptom domains

Prevalence estimates varied strongly between syndromes and symptom domains (*R*^2^ = 21.2%), but all individual estimates deviated significantly from zero (Fig. 2). In the severity model, *R*^2^ of the syndrome and symptom domain was 48.7%.

Since burnout is a multidimensional construct, and ECRs may experience its subdimensions to varying degrees, we conducted post hoc exploratory analyses (without FDR control) examining the severity across the three burnout subscales (emotional exhaustion, depersonalization and personal accomplishment; Supplementary Table 16). Severity was lowest for depersonalization (−7.5; 95% CI, −8.4 to −6.9), followed by emotional exhaustion (8.5; 95% CI, 1.6–15.3), while reduced personal accomplishment had the highest severity (26.7; 95% CI, 17.1–36.3). These results suggest that ECRs most often reported feelings of being ineffective or lacking personal accomplishment.

#### Utilized scale

The scale used to assess mental health syndromes or symptom domains moderated both prevalence and severity estimates (*R*^2^ = 34.4% and 53.5%, respectively). As shown in Fig. 3, severity levels varied widely depending on which measurement scale was used. To facilitate interpretation, the severity estimates displayed in Fig. 3 were back-transformed into their original units of measurement. For example, the pooled severity for depressive symptoms as assessed with the PHQ-9 (44 studies, 67 effect sizes; *n* = 72.239) was 6.99 (95% CI, 6.12–7.86), indicating symptom levels in the mild range. Additional details on each respective scale, including scale ranges, scoring method and severity thresholds, are available in Supplementary Tables 20–29.

Overall severity estimates for depression ranged from mild to severe, and for GAD from mild to borderline abnormal, whereas assessments of eating disorders and substance abuse were consistently below clinical thresholds. At the symptom level, moderate levels of stress and psychological distress were observed (scoring below clinical thresholds), with psychological distress defined here by broad screening tools (WHO-5, K-6 and K-10) rather than as an umbrella construct as in the rest of the present meta-analysis. Likewise, suicidal ideation did not exceed the clinical cut-off. However, clinical thresholds for suicidal ideation have been increasingly criticized, as systematic reviews show that these cut-off scores have low predictive value of actual suicide. Importantly, subthreshold levels of suicidal ideation still include suicidal thoughts, which can be clinically relevant^59,60^. Sleep quality ranged between unproblematic and poor, but few data were available. Effect-size estimates per scale are available in Supplementary Tables 30 and 31, including comparisons to the most recent meta-analyses for depression and GAD.

#### Sample characteristics, including risk and protective factors

We tested a broad range of sample-level characteristics as potential moderators of psychological distress in both the prevalence and severity models. These included academic position (pre- versus postdoctoral), gender, ethnicity, academic discipline, age, country cluster, US sample, history of mental health disorders, social support, supervisor relationship quality, job satisfaction and life satisfaction (descriptive data on age and protective and risk factors can be found in Supplementary Table 32). Samples from the USA reported a lower prevalence of psychological distress (by 0.66%; 95% CI, −2.63 to 0.00) and severity (by 4.00 POMP score points; 95% CI, −8.32 to −0.32) than all other samples (*R*^2^ = 5.1% and 2.9%, respectively). In the prevalence model, the presence of a mental health diagnosis prior to the study increased prevalence estimates (0.002% per 1% increase of prior mental health diagnosis; 95% CI, 0.000–0.006), and loneliness decreased prevalence estimates (−0.002% per 1 POMP-score point increase of loneliness; 95% CI, −0.011 to −0.000), which explained among the small number of studies that reported this information (*k* = 18 and 11) large amounts of the true effect-size heterogeneity (*R*^2^ = 23.5% and 18.7%). Age was associated with lower prevalence (by −0.01%; 95% CI, −0.03 to −0.00); thus, per ten-year increase in age, the overall prevalence for mental health syndromes and symptoms was 1% lower. Together, these findings indicate that the prevalence and severity of psychological distress were largely similar across demographics and individual protective and risk factors, with only minor associations with sample-level moderators.

#### COVID-19 pandemic

**Fig. 4:**
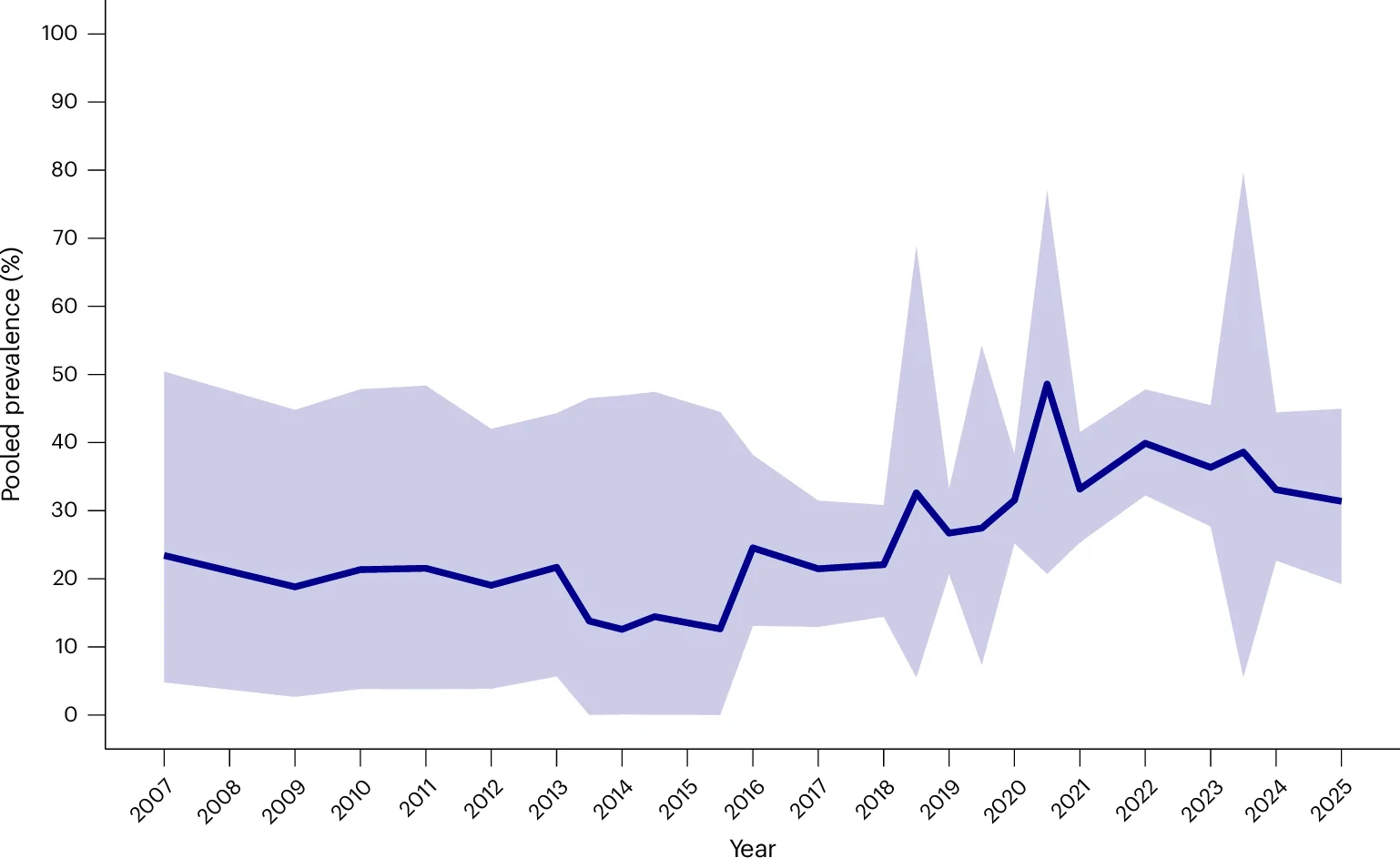
Pooled prevalence of psychological distress over the years 2007–2025. The dark blue line illustrates annual pooled prevalence estimates of psychological distress, expressed as percentages, from multilevel random-effects meta-analytic models for publication years 2007 to 2025. The shaded region is the 95% confidence band. The confidence band is wider prior to 2017 and for several later years, indicating greater uncertainty around annual pooled point estimates.

The analysis on how psychological distress changed from before (before 2020) to during (2020–2022) and after (2023 and onwards) the COVID-19 pandemic yielded mixed results. In the prevalence model, COVID-19 significantly moderated the pooled prevalence of psychological distress (*R*^2^ = 8.7%), such that the prevalence was significantly higher during the COVID-19 period (34.2%; 95% CI, 29.4–39.2) than before (24.3%; 95% CI, 19.8–29.02). Post-pandemic prevalence remained significantly elevated (33.3%; 95% CI, 26.0–41.0; see also Fig. 4). Assessing the linear and quadratic association between prevalence and year of data collection indicated a similar effect of the time of the COVID-19 pandemic.

The severity model indicated that psychological distress was higher during the COVID-19 period (POMP score, 34.63 (31.48, 37.77)) than during the period after (POMP score, 31.89 (26.23, 37.55)), but these estimates were not significantly different from the pre-pandemic period (POMP score, 33.81 (30.62, 36.99); *R*^2^ = 0.25%). Additionally, a polynomial moderator analysis with publication year modelled as a linear and quadratic predictor revealed no significant time trends (Supplementary Table 18 and Supplementary Fig. 13).

#### Study design and quality

All pooled prevalence and severity estimates reported above were based on data from all eligible studies, irrespective of study design or quality. Study quality was a significant moderator, such that higher study quality was associated with lower prevalence (−0.14% per JBI score point; 95% CI, −0.40 to −0.01; *R*^2^ = 11.6%) and severity estimates (−3.06 POMP score points per JBI score point; 95% CI, −4.65 to −1.46; *R*^2^ = 7.7%).

Sampling strategy was a significant moderator as well, with studies using full or random sampling reporting lower prevalence (−2.61%; 95% CI, −5.37 to −0.83; *R*^2^ = 11.6%) and lower severity estimates (−17.7 POMP score points; 95% CI, −23.1 to −12.29; *R*^2^ = 15.3%) than those using convenience sampling.

#### Multivariate moderator analyses

To evaluate the extent that FDR-adjusted significant moderators without missingness in the data collectively explained variance above the sampling error, we conducted multivariate moderator analysis for both the prevalence and severity of psychological distress. For the prevalence model, we included the type of syndrome or symptom domain, utilized scale, whether the sample was from the USA or not, overall study quality, sampling strategy, assessment strategy, COVID-19 period and year. For the severity model, we used the same predictors minus assessment strategy, COVID-19 period and year. The multivariate moderator model explained 43.1% of the true effect-size heterogeneity in the prevalence model (*F*(55, 296) = 9.61, *P* < 0.001) and 52.4% in the severity model (*F*(100, 435) = 10.04, *P* < 0.001).

### Risk of publication bias

The qualitative assessments of asymmetry in the funnel plots of the prevalence and severity models (Supplementary Figs. 14 and 15) were both indicative of publication bias (via small-study effects—that is, larger effects among smaller and less precise studies). This was confirmed by Egger’s regression tests for the severity model but not the prevalence model (prevalence: *F*(1, 350) = 2.58, *P* = .109; severity: *F*(1, 534) = 22.96, *P* < .001). A sensitivity analysis revealed that publication bias did not impact the significance of the already significant moderators (Supplementary Table 18).

## Discussion

This multilevel meta-analysis assessed the prevalence and severity of psychological distress among ECRs across a broad range of mental health syndromes and symptoms. To understand the underlying factors affecting the prevalence and severity of psychological distress, we conducted moderation analyses on a wide set of sample- and study-level characteristics. From 228 samples drawn from 148 studies and four databases (*N* = 138,446), our results indicate that 29% of ECRs scored above screening thresholds for at least one syndrome or symptom. In terms of severity, ECRs reported mild levels of depression and anxiety, moderate stress and generally poor sleep quality, while levels of severity of suicidal ideation and eating disorders fell below clinical cut-offs. Moderation analyses indicated that prevalence and severity estimates were largely unaffected by demographic or individual factors, but rather due to study-level characteristics such as sampling strategy, study quality and type of measurement scale. In addition, the prevalence of psychological distress increased during the COVID-19 pandemic and has remained elevated since. The consistency of these findings across sample characteristics further underscores the widespread extent of mental health issues among ECRs.

Our findings are broadly consistent with those of other meta-analyses, but they fall on the higher end of previously reported ranges of 21–24% for depressive symptoms and 17–30% for anxiety symptoms^9–11^. When compared with age-matched groups in the general population, our results indicate that depressive (29%) and anxiety symptoms (29%) were markedly elevated among ECRs. Although the magnitude of this difference depends on the reference data^61^, population-based studies of young adults using comparable screening thresholds (for example, PHQ-9 ≥ 10; GAD-7 ≥ 10) typically report prevalences of 8–17% for depressive symptoms and 6–10% for anxiety symptoms^31–33^. This suggests that depressive symptom prevalence among ECRs is approximately two to three times higher, and anxiety symptom prevalence about three to five times higher, than among same-age peers in the general population. While the prevalence moderately increased during the COVID-19 pandemic, our findings indicate that elevated rates of depressive symptoms in particular and psychological distress in general predate the pandemic and have persisted over time^10^. Beyond depressive and anxiety symptoms, elevated prevalence was also observed for other important syndromes and symptoms, such as poor sleep quality, eating disorder symptoms, alcohol abuse, suicidal ideation and non-suicidal self-injury. Together, the consistently elevated prevalence across a wide range of mental health outcomes underscores the broad scope of psychological distress among ECRs.

Another central aim of this meta-analysis was to quantify the severity of psychological distress across a wide range of mental health syndromes and symptoms. According to the current data, a large proportion of ECRs reported elevated levels of psychological distress. Depending on the measurement scale, the average symptom severity across syndromes ranged from mild to severe for depressive symptoms and from mild to borderline abnormal for anxiety symptoms, and fell below screening-based severity cut-offs for eating disorder symptoms, substance abuse (for example, alcohol abuse) and suicidal ideation. For symptoms, average sleep quality was poor (with a wide estimate range across studies), and stress was consistently reported as moderate, underscoring the wide range of stressors experienced by ECRs^62^. Because prevalence is estimated as the proportion of individuals in a sample scoring above case-identification thresholds, whereas symptom severity reflects whole-sample mean scores on continuous scales, these two metrics capture distinct but complementary information about ECR mental health. The high prevalence of psychological distress is accompanied by, on average, generally mild symptom severity in the overall investigated samples, which reflects that mental-health issues are widespread rather than focused on subgroups with high symptom severity. In other words, even mild or subclinical symptom severity can indicate cause for concern and elevated future risk^60,63^.

In addition to estimating the prevalence and severity, this meta-analysis assessed sample characteristics for potential moderating effects, including gender, age, career stage, country, ethnicity and history of mental health disorders, as well as protective and risk factors such as social support, supervisor relationship quality, job and life satisfaction, and loneliness. Prevalence was not consistently associated with most sample characteristics. Small but statistically significant associations were observed for loneliness and age (both were associated with lower prevalence) and history of mental health disorders (associated with higher prevalence). However, these effect sizes were minimal and unlikely to be practically meaningful. For severity, there was no evidence that sample characteristics acted as moderators. These findings are broadly consistent with those of other meta-analyses^10,11^, which also reported no consistent effect of gender, country income level or academic system. The limited evidence for sample-level moderators suggests that psychological distress among ECRs is not limited to specific subgroups but instead reflects a broader and systematic issue in academia. For example, prevalence and severity estimates were comparable across disciplines, genders and career stages. However, post hoc sensitivity analyses indicated that studies conducted exclusively with samples from the USA reported significantly lower prevalence and severity of psychological distress than studies with non-US samples.

While sample-level moderators were not consistently associated with prevalence and severity estimates, several study-level moderators were. The type of measurement scale and the syndrome or symptom domain explained a substantial proportion of the variance (*R*^2^ of up to 51%), indicating that which outcome is measured and how it is measured strongly shapes prevalence and severity estimates. In addition, moderator analyses on the role of the COVID-19 pandemic indicated that pooled prevalence and severity were higher during the pandemic, and that prevalence (but not severity) has remained significantly elevated. Finally, prevalence and severity were lower with higher study quality and with random or full sampling. These findings are in line with those of previous meta-analyses, which found that low-quality primary studies or those using non-random sampling techniques reported higher prevalence rates for depressive^10^ and anxiety symptoms^11^. Taken together, these results underscore the methodological challenges in estimating psychological distress among ECRs. To obtain a more accurate estimate of the prevalence and severity of psychological distress among ECRs, future studies should incorporate semistructured clinical interviews conducted by trained professionals to establish clinical diagnoses of mental disorders, as self-administered mental health scales only screen for potential cases^64^. For regular monitoring or large-scale epidemiological assessments, or when structured clinical interviews are not feasible, future research should rely on well-validated scales.

At the same time, these conclusions on moderators should be interpreted with caution. A number of sample-level moderators were only sparsely reported (for example, academic discipline and history of mental health disorders), and some study-level moderators also explained substantial variation in prevalence and severity estimates of psychological distress (for example, outcome measures and utilized scale). In addition, other unmeasured individual-level factors could explain variations in mental health. For example, the impostor phenomenon (or impostor syndrome)^65^, characterized by persistent self-doubt despite objective success, has been linked to increased stress and depressive symptoms in ECRs^55^. Impostor phenomena may also overlap with a reduced sense of personal accomplishment (a subscale of burnout), which was observed in this meta-analysis. Future research should therefore incorporate a range of individual-level moderators and longitudinal designs to enable more precise meta-analytic estimates and to establish temporal dynamics, mechanistic pathways and causal relationships underlying psychological distress among ECRs.

The modest associations of individual-level moderators with psychological distress suggest that the primary drivers of poor mental health among ECRs are structural rather than personal. The most frequently cited systemic factor is the mismatch between the growing number of PhD graduates and the comparatively limited number of permanent positions in academia^4,40,66–68^. As a result, most ECRs work on short-term or part-time contracts that involve career uncertainty, frequent relocations and financial insecurity. At the same time, norms about research productivity have shifted towards metric-based performance indicators (for example, *h*-index, citation count, external funding and publication output), which increasingly shape hiring, tenure and funding decisions^69^. Critics argue that universities themselves are increasingly structured around market-oriented or neoliberal principles^40,70^, such as performance-based funding, external audits and university rankings, which further reinforce metric-driven evaluations^71,72^. These shifts incentivize research that is safe, short-term and likely to generate high citation counts, while discouraging riskier, long-term or niche projects^4,68,73,74^. The result of these shifts in productivity norms, combined with the lack of permanent positions and stable career paths, is a climate of heightened competition^75^ and a work culture that valorizes overwork (for example, publish-or-perish narratives), characterized by blurred boundaries between work and leisure time, lack of breaks and guilt about taking them^40,76,77^. Finally, for ECRs from underrepresented groups, additional barriers may exist, such as discrimination, limited mobility and unequal access to mentorship and resources^40,78,79^. Together, these structural factors create substantial psychological strain and probably contribute to elevated levels of anxiety, stress and sleep problems among ECRs.

The present meta-analysis has several implications for research institutions and universities, funders and policymakers, supervisors and ECRs themselves. Although this meta-analysis did not evaluate the effects of specific interventions, the modest associations of individual-level factors with psychological distress suggest that systemic factors probably play a substantial role and therefore require structural and institutional changes. For institutions, the findings underscore the need to improve working conditions. Institutions should expand access to support services (such as structured coaching programmes and confidential ombudspersons) and dedicated mental health counselling, for example through collaborations with existing medical or psychology departments. They could also mandate supervisor training focused on mentoring quality, expectation-setting, effective feedback, communication and early distress recognition as part of professional-development requirements. Annual institutional or faculty-level surveys could monitor mental health and evaluate the effects of implemented policies. Academic institutions should also provide career guidance^80^ (for example, structured individual development plans integrated into annual performance reviews), preparing ECRs for careers inside and outside of academia. Policymakers and funders can address structural issues by increasing the number of permanent positions, promoting fairness and transparency in promotion criteria, and establishing safeguards against power abuse and discrimination such as independent whistleblower channels. Nationwide surveys on mental health and academic working conditions could be established or expanded to monitor systemic progress over time^2,52^. Supervisors can actively address mental health issues, participate in relevant skills training and foster working environments in which employees feel safe to voice their concerns and discuss challenges^81^. Finally, for ECRs themselves, the findings can help normalize conversations about mental health issues, destigmatize seeking help and encourage engagement with individual-focused interventions. At the same time, evidence on interventions targeting mental health in ECRs remains limited and is much needed. Potentially helpful interventions include stress management, cognitive behaviour therapy^82^, mindfulness- and compassion-based interventions^46,83,84^ or social support interventions (for example, peer support networks^58^).

We note four methodological limitations and suggest directions for future research. First, the quality of the primary studies eligible for this meta-analysis varied greatly. For example, only one of the studies conducted an external assessment via clinical-diagnostic interviews to establish diagnoses, rather than relying on self-rating scales^49^. Additionally, only a few studies (predominantly databases) used random (instead of convenience) sampling techniques^50–53,56–58^. However, both the use of self-rating scales and convenience sampling appear to inflate effect sizes^10,85^. Furthermore, due to convenience sampling, generalizing the results to a greater sample frame remains difficult, as the pooled effect sizes might be biased by self-selection^86^. Second, postdoctoral ECRs made up less than 3% of the overall sample^5,52,57,66,68,87–93^, yet they face slightly different challenges than doctoral students, and thus, more research should be dedicated to this group. In doing so, we recommend that future research clearly differentiate between the different student levels and academic positions, as this would have enabled the inclusion of more records. Third, for some syndromes and symptoms, the available evidence was drawn exclusively from studies conducted in the USA (for example, eating disorders, non-suicidal self-injury and alcohol abuse), and for syndromes or symptoms, it was limited overall (for example, sleep quality and personality disorders). Future research should prioritize lesser-studied outcomes and expand to geographically diverse samples, as the current evidence disproportionately draws on Western (especially US-based) samples, which may obscure variation in ECR mental health across academic systems and cultural contexts^94,95^. Fourth, our clustering of countries was exploratory. Although different LLMs yielded converging classifications, further research is needed to develop and validate more robust frameworks for grouping countries on the basis of academic working conditions.

As for future inquiry along these lines, our review has underscored the need for further investigations into potential risk and protective factors, as existing studies often did not report these variables. Although this meta-analysis did not find any significant buffering or exacerbating effects of supervisor relationships, social support, loneliness, or life and work satisfaction, this does not mean that such effects do not exist. As almost all tests of these moderators lacked power, future research should direct more efforts to determine potential protective and risk factors in a congruent manner and thereby deduce effective and evidence-based interventions and implement systemic changes to reduce the psychological distress of ECRs. Such moderators could be the contract or funding type^34,96,97^, supervisor variables (for example, leadership style or perceived support from the supervisor)^14^, financial strain^50,98^, socio-economic^99^ or minority status, and additional predictors of psychological distress that may be uniquely intensified or moderated in the ECR population.

This meta-analysis found that nearly one in three ECRs reported elevated psychological distress across all outcomes, with mild average symptom severity for depressive and anxiety symptoms, moderate stress and poor sleep quality. These patterns were consistent across academic disciplines, genders and career stages. These results indicate a widespread and systemic burden of psychological distress among ECRs, with mild overall symptom severity nonetheless signalling heightened risk for future mental problems. Targeted reforms at the institutional and policy levels, ongoing monitoring and strengthened individual coping resources could help improve mental health and working conditions in academia.

## Methods

This review was conducted in accordance with the PRISMA 2020^100^ guidelines. The study protocol was registered on 13 October 2024 in PROSPERO (ID: CRD42024596813). Deviations from the preregistration are recorded in Supplementary Table 33.

### Outcomes and definitions

For this systematic review and multilevel meta-analysis, two key terms necessitated a more precise operationalization: ECRs and mental health. ECRs, as defined by the European Council^101^, are researchers enrolled in or working for higher education institutions who are actively pursuing a PhD or researchers with a PhD who work in the initial stages of their academic research career (corresponding to R1 and R2 levels, respectively). This includes pre- and postdoctoral students, research associates, graduate students, doctoral candidates, PhD students, postdocs, postdoctoral researchers, postdoctoral fellows, (junior) lecturers, readers and junior researchers. Predoctoral research assistants (for example, master’s students) who were involved in research projects but were not actively enrolled in a PhD programme were not included. In the present analysis, a cut-off of no more than six years of postdoctoral working experience was applied to distinguish between ECRs and mid-career researchers, as this criterion was adopted from one of the studies included in the review^68^. In terms of mental health outcomes, the review was guided by definitions of mental health syndromes and screening criteria outlined in the 11th revision of the International Classification of Diseases^102^. Following these definitions, we included a wide range of mental health outcomes subsumed under the umbrella term ‘psychological distress’, including clinically defined syndromes as operationalized via screening instruments such as depression, GAD, social phobia, OCD, PTSD, ADHD, personality disorders, eating disorders, alcohol and drug abuse, and burnout, as well as common symptoms of psychological distress such as stress, sleep disturbance, non-suicidal self-injury, suicidal ideation and somatic symptoms. We use the term ‘symptom domains’ to refer to both composite or grouped symptoms (for example, stress and sleep disturbance) and individual symptoms (for example, suicidal thoughts). In the more technical parts of the Article, we use ‘symptom domain’ for precision. In the narrative parts of this Article (Abstract, introductory text and Discussion), we use the term ‘symptom’ for readability.

### Search strategy and search string

The search was initially conducted in Scopus and complemented by forward and backward citation search of relevant systematic and narrative reviews and meta-analyses; it was then supplemented by additionally searching the databases ERIC, PubMed and Web of Science (the references of all included studies and databases are listed in Supplementary Table 1; see Supplementary Table 33 for details on deviations from the preregistration). Scopus and PubMed were selected because they range among the largest abstract and citation databases for peer-reviewed literature in the field of biomedical research and (for Scopus) psychological and educational research. ERIC complemented the search in the field of educational research and also provided access to grey literature. Searches in these databases were complemented by a search in Web of Science that offers access to cross-disciplinary and interdisciplinary publications. The databases were searched through 28 October 2025. With references retrieved through backward and forward citation search, this systematic review and meta-analysis synthesized data from 228 samples and 138,446 individuals, representing the largest quantitative synthesis of psychological distress among ECRs to date. The following string, combining key terms defining the type of research position, mental health syndromes and symptoms, and methodological determinants, was used (see also Supplementary Table 34): “early*career researcher” OR “ecr” OR “doctor* candidate” OR “doctor* level” OR “doctor* researcher” OR “graduate student” OR “phd candidate” OR “phd student” OR “pgr” OR “Ph.D.” OR “phd” OR “post-graduate research student” OR “postgraduate research student” OR “postgraduate researcher” OR “post-graduate researcher” OR “post-doc” OR “research student” OR “postdoc*” AND “mental illness” OR “psychiatric illness” OR “psychiatric problems” OR “psychiatric symptom*” OR “psychological well-being” OR “psychopathology” OR “disorder” OR “psychopathology” OR “mental health” OR “post-traumatic” OR “stress” OR “burnout” OR “distress” OR “PTSD” OR “PSS” OR “OCD” OR “obsessive-compulsive” OR “obsessive compulsive” OR “addiction” OR “abuse” OR “substance abuse” OR “addictive behavior” OR “anorexia” OR “eating disorder” OR “ED” OR “body dysmorph*“ OR “binge eating” OR “bulimia” OR “depress*” OR “cyclothymic” OR “mood disorder” OR “suicide” OR “dysthymic” OR “dysphoric” OR “mania” OR “manic” OR “bipolar” OR “affective disorder” OR “anxiety” OR “panic attacks” OR “GAD” OR “general anxiety disorder” OR “anxiety disorder” OR “panic disorder” OR “social phobia” OR “phobia” OR “insomnia” OR “sleep-wake disorder” OR “sleep disorder” OR “delus*” OR “dissociative” OR “hallucinat*” OR “psychosis” OR “psychotic” OR “SAD” OR “schizo*” OR “personality disorder” OR “PD” OR “hoarding” OR “hypochondriasis” OR “loneliness” OR “MDD” OR “self-harm” OR “suicidality” OR “suicide*” OR “drug abuse” OR “DAST” OR “AUDIT” OR “NSSI” AND “quant*” OR “survey” OR “cross-sectional” OR “questionnaire”.

### Inclusion and exclusion criteria

Any study that evaluated and reported the prevalence or summary scores of any mental health disorder syndrome or symptom domain among ECRs was included. No exclusion criteria were formulated regarding the record type. Thus, journal articles, preprints, conference papers, reports, book chapters and theses were all eligible for inclusion. All included studies and samples in the current meta-analysis and systematic review^5,13,33,34,46,49–58,66,68,87–93,96–99,103–205^ are listed in Supplementary Table 1. Overall, 32 non-peer-reviewed records were included^52,54,107,111,118,125,132,135,138,148,177,178,191,196,202,206–222^. Articles of any language were included and translated using DeepL, if necessary.

Supplementary Table 39 lists detailed study exclusion reasons. Records were excluded if:

1. The sample comprised undergraduate students; graduate students pursuing a master’s degree or a professional doctorate; mid-stage to senior-stage researchers, such as senior lecturers, senior scientists, senior research fellows, associate professors, assistant professors or full professors; research group leaders; principal investigators; adjunct faculty; adjunct professors; research directors; or heads of departments, or comprised fewer than three participants.
2. The data of samples of interest were not reported separately from the data of the overall, mixed samples assessed in the study.
3. The record used a scale that was not used in at least one other record or did not provide information on the percentage of individuals scoring above a screening threshold.
4. The record did not provide information on any of the outcomes of interest within its main text, supplementary material or any other related material, or via direct contact with the authors.

### Study selection and eligibility

The identification of studies via databases and the other described methods yielded a total of 10,534 articles published up to 28 October 2025. An independent screening based on title, abstract and full text was conducted, which resulted in 148 records that met the predefined inclusion criteria. During the screening, it became apparent that several publications had used the same datasets. In these cases, we decided to include the primary datasets rather than derivative articles, resulting in a screening of the databases. Four databases^14,52,118,208^ were included.

### Data extraction

Data extraction followed two steps and was conducted by V.G. First, all screened articles were documented in Excel, and duplicates were identified by using the ‘duplicate values’ function. In case of duplicate records or records that used the same data, only one record was included. Exclusions based on ‘title’, ‘abstract’ and ‘full text’ were then tracked. For reports screened in full text, the reasons were documented (see Supplementary Table 39 and Fig. 1). In cases where the original report was not accessible or the report lacked information on the exact sample composition, the corresponding authors were contacted via a standardized mail template (Supplementary Table 35) up to two times, and their responses were documented in Excel. If the exact sample composition (pre- and postdoctoral or master’s students) could not be clarified, the study was excluded. Of the 464 authors contacted, we received 188 responses (40.5% response rate; not counting authors that could not be contacted due to missing contact information).

Second, information from the included articles was extracted, using a coding scheme that was iteratively adjusted (Supplementary Table 36). The extracted information encompassed data on the source (authors, publishing year, the type of report and whether or not it was peer-reviewed), study characteristics (the year in which data were collected, country, sample size, sampling method, scales used to assess the outcomes and moderators), sociodemographic information on the participants (academic position, age, percentage female and diverse, ethnicity and faculty affiliation), mental health information on the participants (history of mental health disorders, summary scores and proportion of those above a predetermined threshold) and potential additional diagnostic criteria and individual-level factors (job satisfaction, life satisfaction, supervisor relationship quality, social support and loneliness). To ensure coding reliability and to mitigate inconsistencies in the data extraction^223^, 30 articles were randomly selected and independently coded by author D.F. Any inconsistencies were discussed to reach consensus, also drawing on a third author (U.S.T.), if needed.

Studies were coded as pre-COVID-19 (data collected before 2020), during COVID-19 (2020–2022), and post-COVID-19 (after 2022) to examine the impact of the COVID-19 pandemic. For studies that did not explicitly report the year of data collection, we contacted the authors. In case of no response, the data collection year was estimated by subtracting 1 from the publication year.

Countries were exploratively clustered using two LLMs (ChatGPT-4o by OpenAI and DeepSeek R1 by DeepSeek), because there is no universally standardized classification system for predoctoral (for example, PhD candidates) and postdoctoral positions that groups countries on the basis of criteria such as funding schemes, duration, contract type, working hours or other relevant factors. Both tools were instructed with the same prompt to cluster countries according to similar shared systemic job and working characteristics for pre- and postdoctoral positions. The authors subsequently discussed the (already quite similar) classifications provided by the two LLMs and merged them into a common system. This classification was then further validated using two other LLMs (Gemini 2 Flash by Google and ChatGPT-5.1 by OpenAI; Supplementary Material 1 and Supplementary Tables 3–7).

### Effect-size measures

The extent of psychological distress among ECRs was investigated separately with respect to prevalence and severity. Prevalence was estimated for outcomes using psychometrically validated or commonly used screening thresholds (for example, PHQ-9 ≥ 10) or binary thresholds (yes/no) response formats (Supplementary Tables 1 and 20–29). Symptom severity was estimated with summary statistics (means and standard deviations) of continuous measures. Because primary studies employed a wide range of instruments to assess psychological distress and related syndromes and symptoms, the summary statistics were not directly comparable across studies. To address this heterogeneity, and following previous meta-analyses^224,225^, we converted summary statistics into POMP scores^226^. POMP scores transform different, arbitrary scores (of tests, scales, surveys and so on) into a standardized scale ranging from 0 to 100. For example, if a respondent scores a 5 on a scale ranging from 1 to 7, this corresponds to a POMP score of 66.67 (see below for details and calculation). A POMP score of 50 corresponds to the midpoint on the original scale, whereas POMP scores above 50 indicate higher and below 50 indicate lower symptom severity than the midpoint of the original scale.

To estimate prevalence and severity, we included studies either reporting the proportion of individuals scoring above a certain cut-off or reporting summary statistics (means and standard deviations). For several measures on positive well-being (that is, WEMWBS, SWEMWBS and WHO-5 Index), POMP scores were reverse-coded, such that higher values consistently reflected poorer well-being. Both analysis models included data on alcohol abuse, drug abuse, ADHD, depression, GAD, eating disorders, sleep quality, general mental health, somatic symptoms and suicidal ideation. The severity model additionally covered stress and burnout, including the subscales of emotional exhaustion, depersonalization and personal accomplishment as subfactors of burnout. The prevalence model also included bipolar disorder, personality disorder, PTSD, OCD, social and specific phobia, agoraphobia, panic disorder and non-suicidal self-injury.

#### Transformation of prevalence

Thresholds identifying a clinically relevant amount or severity of symptoms varied depending on the utilized scale (for an overview of the varying thresholds across the different utilized scales, their scoring methods, ranges and thresholds, see Supplementary Tables 20–29). Thresholds were based on psychometric validation studies or on common practice (for example, PHQ-9 item 9 to assess suicidal ideation).

The range of prevalence rates is doubly bounded, in that they range from zero to unity^227^. This causes the standard error to become artificially compressed for rates close to either 0% or 100%. This overestimation of the precision of individual prevalence rates entails the assignment of too-high weights in the meta-analytic model when estimating the pooled effect size. Hence, prevalence rates were transformed prior to analysis, using the single (not double) arcsine transformation to stabilize the variance, which has been proved to generate more adequate results than the double arcsine transformation when dealing with varying sample sizes^228,229^:

$${\rm{transformed}}\,{\rm{ES}}={\rm{ASIN}}(\sqrt{{\rm{ES}}}),$$

$${\rm{transformed}}\,{\rm{var}}=\frac{1}{4n}.$$

The transformed values were then back-transformed for interpretation of the results using the inverse formula:

$$\mathrm{back}\mathrm{-}\mathrm{transformed}\,\mathrm{prevalence}=\sin {(\mathrm{ES})}^{2}.$$

#### Calculation of POMP scores

POMP scores are calculated via:

$${\rm{POMP}}=\frac{{\rm{observed}}-{\rm{minimum}}}{{\rm{maximum}}-{\rm{minimum}}}\times 100$$

$${\rm{s}}.{\rm{d}}.({\rm{POMP}})=\frac{{\rm{s}}.{\rm{d}}.}{{\rm{maximum}}-{\rm{minimum}}}\times 100$$

In cases where summary scores were not provided, yet information on the percentage per rung or category was given, we calculated expected values of *M* and s.d. in four steps. For scales such as the PHQ-9, GAD-7, DASS or HADS, where percentages were provided per category but not per value^34,53,58,84,105,124,127,134,145,155,162,170,177,179,201,207,230–232^ (see also Supplementary Table 37), we determined the category mean values and calculated the expected values of *M* and s.d. using the following formulas:

$$M=\displaystyle \mathop{\sum }\limits_{i=1}^{n}{x}_{i}\times {p}_{i}$$

$${\rm{s}}.{\rm{d}}.=\sqrt{\displaystyle \mathop{\sum }\limits_{i=1}^{n}{p}_{i}{({x}_{i}-M)}^{2}}$$

Where no probability distribution was provided, such as in the case of item 9 of the PHQ-9, it was necessary to first determine the items’ probability distribution^34,58,105,230,232^. For this, we used the largest database available (Healthy Minds Network, *n* > 50,000), which provided us with the score distribution of 0 = 87.16%, 1 = 9.40%, 2 = 2.13% and 3 = 1.32%. From these figures, we determined that the probability to score 2 is 1/4.41 times the probability to score 1, and the probability to score 3 is 1/7.12 times the probability to score 1. The proportion to score 1 was thus determined via the following formula, solving for *x*:

$${{ \% }}\,{\mathrm{at}}\,{\mathrm{or}}\,{\mathrm{above}}\,{\mathrm{cut}}{-}{\mathrm{off}}\,(\ge 1)=0x+1x+\left(\frac{1}{4.41}\right)x+\left(\frac{1}{7.12}\right)x\approx 1.37x$$

For example, in the case of ref. ^58^, 3.1% scored above the cut-off of ≥1. From this, we concluded that 96.9% must have scored 0, and using the above formula we estimated that 2.27% scored 1, 0.51% scored 2, and 0.44% scored 3. With this, we then used again the above formulas for *M* and s.d. The exact analysis with the raw data is available via OSF (https://osf.io/r9nkd).

### Critical appraisal

We used the JBI critical appraisal tool for epidemiological studies^233^, which evaluates the methodological quality of a study on the basis of nine categories: (1) sample frame, (2) sampling, (3) sample size, (4) description of study subjects and setting, (5) sufficient coverage for data analysis of the identified sample, (6) validity of methods used for identifying the condition/mental health syndromes, (7) reliability and standardization of measures used, (8) appropriateness of the statistical analysis and (9) adequacy of the response rate. The ratings assessed the quality of studies themselves and not of unique effect sizes; all effect sizes from the same study received the same score. Questions were refined by V.G. and U.S.T. to better assess the extent to which information (for example, on subjects or setting) was described in sufficient detail. Rather than awarding only zero or full points, studies were able to score quarter points on the basis of whether they provided key information, such as the age of the participants, the country in which the study was conducted or the gender composition. To evaluate the adequacy of response rates (that is, the rate of persons contacted by the study authors who also consented to participate in the study), an external cut-off criterion of 45% was employed, on the basis of the average online survey response rate^234^. Studies could score a full point if the response rate was adequate (≥45%), a quarter point if the response rate was provided but below 45% and no points if no information was provided. The full coding scheme and all scores per study are provided in Supplementary Tables 12 and 13. For moderator analysis, the scores on the individual categories were summed.

### Analysis

Analyses were conducted in R Quarto (version 4.4.3) using the following packages: metafor (4.8-0)^235^, metaforest (0.1.5)^236^, tidyverse (2.0.0)^237^ and the integrated ggplot2 (4.0.0) for data visualization^238^. For parameter estimation, restricted maximum likelihood estimation was applied, and for standard errors, *P* values and 95% CIs, Knapp and Hartung’s adjustment^47,227^ was used. For further specifications about the analysis, the R script and the data are available in Supplementary Materials 1 and 2 (https://osf.io/r9nkd).

#### Model fit and heterogeneity

As studies reported more than one effect size (for example, depression and suicidal ideation), we opted for a multilevel meta-analysis with three levels (for example, ref. ^47^), a random-effect model, which enables modelling the dependent effect sizes from the same study. Variation in the effect sizes was partitioned into three levels: participants (Level 1; sampling error), outcomes (Level 2; here the syndromes and symptoms) and studies (Level 3). Model fit was determined via analysis of variance, on the basis of the Akaike and Bayesian information criteria, and a likelihood ratio test, comparing a classic two-level model and the three-level model. For parameter estimation, restricted maximum likelihood estimation was applied. Heterogeneity was assessed using the *Q* test (indicating whether the variance in the data exceeded the sampling error), the *I*^2^ statistic (quantifying the amount of variation beyond variance attributable to the sampling error^227^) and *τ* (the standard deviation of the true effect sizes) as well as the 95% prediction interval (providing information on the range into which outcomes of future studies should fall^227^). An overall *I*^2^ greater than 50% was considered to be indicative of substantial heterogeneity^239^.

#### Moderator analysis

To assess the effects of potential moderators, such as participant and study characteristics, syndrome or symptom type, type of measurement scale, study quality, and POMP scores of risk and protective factors, we conducted moderator analyses separately for the prevalence and severity models. We first performed bivariate analyses to examine the association between individual moderators and prevalence and severity estimates. In the subsequent step, we conducted separate multivariate moderator analyses for the prevalence and severity models, including only moderators that remained significant after we controlled for the FDR in the bivariate analyses. Unless otherwise specified, models estimating prevalence and severity used data from all eligible studies, irrespective of study quality. Study quality (overall JBI score) was added later as a moderator in bivariate and multivariate moderator analyses to assess its association with prevalence and severity estimates.

To account for multiple testing, the Benjamini–Hochberg^240^ correction was applied, and the FDR was controlled at 5%. Adjusted *P* values are reported in the main text; adjusted and non-adjusted *P* values are provided in Supplementary Table 19. Several moderators (loneliness, social support and job or life satisfaction) were measured with diverse scales; these scores were transformed into POMP scores prior to analysis (with higher scores representing lower satisfaction or higher loneliness). An originally planned random forest analysis^236^ (to retrieve information on the variable importance and explained variance of the various moderators) was replaced by a multivariate moderator analysis due to high data missingness for many moderators. The results of the random forest analysis are nonetheless provided in Supplementary Material 2 and Supplementary Figs. 16 and 17.

#### Outlier and sensitivity analysis

We assessed Cook’s distance (Supplementary Fig. 18) separately per model, on the basis of the 4/*n* cut-off. Seven outliers were found in the prevalence model^36,96,161,195,221,230^, and 13 in the POMP-score model^55,105,107,121,128,186,189,209,211,216,230,241,242^. There was more than one outlier per study at times, which is why the number of outliers does not match the number of listed publications. No striking differences were found in the overall prevalence and severity estimates, and neither was reduced heterogeneity when excluding outliers (Supplementary Table 38), which is why they were kept in all analyses.

### Publication bias

Publication bias was visually assessed via funnel plots for multilevel meta-analysis using the code provided by Fernández-Castilla and colleagues^243^, and formally with the three-level version of Egger’s regression test^244,245^.

### Use of artificial intelligence

To create a classification system for working conditions in academia, we used LLMs (ChatGPT 4.1 by OpenAI and DeepSeek R1 by DeepSeek, and ChatGPT 5.1 by OpenAI and Gemini 2 Flash by Google for further validation). For details, see Supplementary Material 1 and Supplementary Tables 3–7.

### Reporting summary

Further information on research design is available in the Nature Portfolio Reporting Summary linked to this article.

## Supplementary information

Supplementary Information Supplementary Tables 1–39, Materials 1 and 2 and Figs. 1–18.

Reporting Summary

Peer Review File

## Data Availability

All data used for this meta-analysis are available via OSF at https://osf.io/r9nkd.

## Code availability

All code is available via OSF at https://osf.io/r9nkd.
