# Supplementary material for: Prevalence and severity of mental health problems in early-career researchers: a systematic review and meta-analysis
Source: Nat Hum Behav. 2026 Jun 29;10(9):1698–717. doi: 10.1038/s41562-026-02505-5 (PMC13590413; doi:10.1038/s41562-026-02505-5)
Supplement: Supplementary file 1 [file 41562_2026_2505_MOESM1_ESM.pdf]

nature human behaviour

Supplementary information

https://doi.org/10.1038/s41562-026-02505-5

Prevalence and severity of mental health problems in early-career researchers: a systematic review and meta-analysis

In the format provided by the authors and unedited

Contents

Supplementary Table 1. Samples included in the three-level meta-analysis 3
Supplementary Figure 1. Histogram of the number of studies conducted per year........................... 24
Supplementary Figure 2. Histogram of the number of studies published per year 24
Supplementary Table 2. Academic discipline classification system.. 25
Supplementary Material 1. Approach of the exploratory country classification 26
Supplementary Table 3. Country clustering based on DeepSeek R1.. 28
Supplementary Table 4. Country clustering based on ChatGPT-4o.. 28
Supplementary Table 5. Final country classification system.. 30
Supplementary Table 6. Country clustering based on ChatGPT-5.2 and the OECD report............... 31
Supplementary Table 7. Country clustering based on Gemini 2 Flash.. 32
Supplementary Table 8. Summary of study and sample characteristics included in the TLMA by type of effect size 33
Supplementary Table 9. Number of studies and estimates per syndrome, symptom and mental health 34
Supplementary Table 10. Outcomes dominated by specific databases.. 35
Supplementary Table 11. Variable analysis of missingness... 36
Supplementary Table 12. Risk of bias (JBI) assessment coding scheme... 37
Supplementary Table 13. Risk of bias (JBI) assessment by category and study (or sample in the case of the HMS and ISWS datasets)... 40
Supplementary Figure 3. Study quality ratings 45
Supplementary Table 14. Effect size heterogeneity**,** with arcsine-transformed effect sizes for the prevalence model.. 46
Supplementary Table 15. Model fit statistics for two- and three-level models.................................. 46
Supplementary Figure 4. Forest plot for the POMP-score model by year 47
Supplementary Figure 5. Forest plot for the POMP-score model by author 48
Supplementary Figure 6. Forest plot for the POMP-score model by outcome.................................. 49
Supplementary Figure 7. Forest plot for the POMP-score model by instrument 50
Supplementary Figure 8. Caterpillar plots for the POMP-score model... 51
Supplementary Figure 9. Forest plot for the prevalence model by year with arcsine-transformed effect sizes 52
Supplementary Figure 10. Forest plot for the prevalence model by author with arcsine-transformed effect sizes 53
Supplementary Figure 11. Forest plot for the prevalence model by outcomes with arcsine-transformed effect sizes 54
Supplementary Figure 12. Caterpillar plots for the prevalence model with arcsine-transformed effect sizes 55
Supplementary Table 16. Post-hoc exploratory and sensitivity analyses in the severity and prevalence models 56
Supplementary Table 17. Results of the moderator analysis 58
Supplementary Table 18. Robustness of moderator effects controlling for publication bias............. 62
Supplementary Table 19. Benjamini-Hochberg FDR corrected *p* values 64
Supplementary Table 20. Information on the threshold, psychometrically validated or defined per common practice, and score method per scale used in the included studies measuring depression 65
Supplementary Table 21. Information on the threshold, psychometrically validated or defined per common practice, and score method per scale used in the included studies measuring general anxiety disorder.. 69
Supplementary Table 22. Information on the threshold, psychometrically validated or defined per common practice, and score method per scale used in the included studies measuring alcohol and drug abuse.. 71
Supplementary Table 23. Information on the threshold, psychometrically validated or defined per common practice, and score method per scale used in the included studies measuring suicidal ideation 72

Supplementary Table 24. Information on the threshold, psychometrically validated or defined per common practice, and score method per scale used in the included studies measuring sleep quality .. 73
Supplementary Table 25. Information on the score method per scale used in the included studies measuring burnout 74
Supplementary Table 26. Information on the score method per scale used in the included studies measuring stress 75
Supplementary Table 27. Information on the score method per scale used in the included studies measuring mental health 76
Supplementary Table 28. Information on the threshold, psychometrically validated or defined per common practice, and score method per scale used in the included studies measuring somatic symptomology 78
Supplementary Table 29. Information on the threshold, psychometrically validated or defined per common practice, and score method per scale used in the included studies measuring disordered eating 78
Supplementary Table 30. Back-transformed POMP scores of the severity estimates 79
Supplementary Table 31. Severity estimates per scale and severity categorization 81
Supplementary Table 32. Mean (*SD*) of age and risk and protective factors per moderator and model 82
Supplementary Figure 13. Graphical display of psychological distress for pooled severity over the years 83
Supplementary Figure 14. Funnel plot for the prevalence model by study effect 84
Supplementary Figure 15. Funnel plot for the POMP-score model per effect 85
Supplementary Table 33. Preregistration deviations 86
Supplementary Table 34. Scopus, ERIC, Web of Science and PubMed Search String 90
Supplementary Table 35. Mailing Template 91
Supplementary Table 36. Coding guide 92
Supplementary Table 37. Algebraic recalculation of missing summary scores 99
Supplementary Material 2. Multivariate exploration of important moderators with a random forest analysis 100
Supplementary Figure 16. Convergence and recursive variance importance plot for the prevalence model 101
Supplementary Figure 17. Convergence and recursive variance importance plot for the POMP-score model 103
Supplementary Figure 18. Outlier and sensitivity analysis with Cook's Distance for the 104
Supplementary Table 38. Comparison of models with and without outliers 105
Supplementary Table 39. Information on studies excluded from the meta-analysis based on reasons for exclusion 106

Supplementary Table 1. Samples included in the three-level meta-analysis

k	**Author, Year**	**Country**	**Year**	**Sample**					**Measure**			**QA**
				n	female (diverse)	Level	Age *M*(*SD*)	Field	Scale	Score method	Cutoffs for prevalence estimates	
1	Acharya et al., 2024	IND	2024	391	52.6%	PhD	NR	SS	GHQ-12[1]	0-3	NA	5.25
									MBI-5	(0-6)	NA	
2	Ahalli et al., 2022	FRA	2019	161	42.2%	PhD	25.8 (2.7)	Mixed	PHQ-9	0-3	0-4 mini.; 5-9 mild; *10-14 mod.; 15-19 mod-sev.; ≥ 20 sev.*	7.75
									GAD-7	0-3	0–5 none; 6–10 mild; 11–15 mod.; ≥ 16 sev.	
									PHQ-9, item 9	0-3	NR (*≥ 1*)	
									AWAI-S-30	1-5	NA	
3	Allen et al., 2020	USA	2017	1039	NR	PhD	NR	Mixed	BDI-II 21	0-3	0-13 mini.; 14-19 mild; *20-28 mod.; ≥ 29 sev.*	5.25
									BAI-21	0-3	0–7 none; 8–15 mild; *16–25 mod.; ≥ 26 sev.*	
									PSS-10	0-4	NA	
4	Almasri et al., 2022	USA	2020	308	45%	PhD	NR	SS	PHQ-9	(0-3)	0-4 mini.; 5-9 mild; *10-14 mod.; 15-19 mod-sev.; ≥ 20 sev.*	5
									GAD-7	(0-3)	0-4 mini.; 5-9 mild; *10-14 mod.; ≥ 15 sev.*	
									PHQ-9, item 9	(0-3)	(*≥ 1*)	
5	Amaral-Prado et al., 2021	BRA	2018	25	80%	PhD	29.8 (4.07)	NR	PHQ-9	0-3	0-4 mini.; 5-9 mild; *10-14 mod.; 15-19 mod-sev.; ≥ 20 sev.*	4
									PSS-14	0-4	NA	
6	Amaral-Prado et al., 2021	BRA	2019	64	73.42%	PhD	28.67 (4.09)	NR	PHQ-9	0-3	0-4 mini.; 5-9 mild; *10-14 mod.; 15-19 mod-sev.; ≥ 20 sev.*	4
									PSS-14	0-4	NA	
7	Amaral-Prado et al., 2021	BRA	2020	31	77.42%	PhD	30.09 (2.79)	NR	PHQ-9	0-3	0-4 mini.; 5-9 mild; *10-14 mod.; 15-19 mod-sev.; ≥ 20 sev.*	4
									PSS-14	0-4	NA	
8	Andrade, 2023	HUN	2021/ 2022	292	65.4%	PhD[2]	31.87 (7.16)	Mixed	CBI-S-25	1-5	NA	3.75
									F-SozU K-6	1-5	NA	
9	Anttila et al., 2015	FIN	2006	163	76%	PhD	38	MED	MEDNORD Exhaustion-4	1-5	NA	5.5
									MEDNORD Stress-1	1-5	NA	
10	Aquino et al., 2018	USA	NR	71	NR	PhD	NR	MED	MBI-ES-EE-9	0-6	0-16 low; 17-26 mod.; ≥ 27 high	2.5
									MBI-ES-DP-5	0-6	0-6 low; 7-12 mod.; ≥ 13 high	
									MBI-ES-PA-8	0-6	≥ 37 low; 31-36 mod.; 0-30 high	
11	Aristeidou & Airstidou, 2023	GBR	2020	1780	55.4% (0.2%)	PhD	NR	Mixed	PHQ-8	0-3	0-4 mini.; 5-9 mild; 10-13 mod.; *15-19 mod-sev.; ≥ 20 sev.*	5.75

[1] Items measuring social dysfunction and anxiety were used as a proxy for psychological well-being.

[2] Doctoral students enrolled in PhD or Doctor of Liberal Arts programs.

12	Baghoori et al., 2022	INT	2020	83	NR	PhD	NR	NR	K-10	1-5	10-19 well; 20-24 mild; 25-29 mod.; 30-50 sev.	2.75
									MHC-SF-14	(0-5)	NA	
									MSPSS-12	1-7	NA	
13	Barreira et al., 2018	USA	2018	513	35%	PhD	NR	Econ.	PHQ-9	0-3	0-4 mini.; 5-9 mild; *10-14 mod.; 15-19 mod-sev.; ≥ 20 sev.*	5
									GAD-7	0-3	0-4 mini.; 5-9 mild; *10-14 mod.; ≥ 15 sev.*	
									UCLA-3	(1-3)	NA	
									AUDIT-C	(0-4)	NA	
									ESP-5	(0-1)	*(≥ 2)*	
									ASRS-VI.I-6	(0-1)	*(≥ 4)*	
14	Barry et al., 2018	AUS	2015	81	81.5%	PhD	38.1 (1.3)	NR	K-10	1-5	10-19 well; 20-24 mild; 25-29 mod.; 30-50 sev.	2.75
									PSS-10	(0-4)	NA	
									DASS-4	0-3	NA	
15	Bastos et al., 2025	BRA	2021-2022	101	66.3%	PhD	29.6 (4.38)	NR	PHQ-9	0-3	0-4 mini.; 5-9 mild; *10-14 mod.; 15-19 mod-sev.; ≥ 20 sev.*	3
16	Bergmans & van der Weijden, 2021	NLD	NR	242	65%	PhD	28	Mixed	GHQ-12	NR (0-0-1-1)	*GHQ4+*	3.75
17	Bitter & McCrea, 2023	USA	2020	63	NR	PhD	26.63 (3.62)	NR	DASS-21*2	0-3	NA	2.75
18	Bitter & McCrea, 2023	USA	2020	23	NR	PD	31.61 (3.43)	NR	DASS-21*2	0-3	NA	2.75
19	Bonanomi et al., 2021	ITA	2020	57	71.9%	PhD	29.5 (4.3)	NR	MHC-SF-14	1-6	NA	5.25
									PSS-4	1-5	NA	
20	Bonetto & Adam-Troian, 2022	FRA	NR	124	74.6% (1.8%)	PhD	28.57 (5.99)	Mixed	BDI-FS-7	0-3	*≥ 4*	3.75
									GAD-7	0-3	0-4 mini.; 5-9 mild; *10-14 mod.; ≥ 15 sev.*	
									PSS-4	0-4	NA	
									BMS-10	0-6	NA	
21	Borgogna et al., 2021	USA	NR	114	84.2% (2.6%)	PhD	26.09 (6.22)	PSY clinical	PHQ-9	0-3	0-4 mini.; 5-9 mild; *10-14 mod.; 15-19 mod-sev.; ≥20 sev.*	4
									GAD-7	0-3	0-4 mini.; 5-9 mild; *10-14 mod.; ≥ 15 sev.*	
									PHQ-9, item 9	0-3	NR *(≥ 1)*	
22	Borgogna et al., 2021	USA	NR	80	75% (1.3%)	PhD	27.65 (6.23)	PSY counselling	PHQ-9	0-3	0-4 mini.; 5-9 mild; *10-14 mod.; 15-19 mod-sev.; ≥20 sev.*	4
									GAD-7	0-3	0-4 mini.; 5-9 mild; *10-14 mod.; ≥ 15 sev.*	
									PHQ-9, item 9	0-3	NR *(≥ 1)*	
23	Bork-Young, 2023	USA	2021-2022	7	57.14% (14.28%)	PhD	31.28	NR	PHQ-9	0-3	0-4 mini.; 5-9 mild; *10-14 mod.; 15-19 mod-sev.; ≥ 20 sev.*	3
									BAI-21	0-3	0-21 low; 22-35 mod.; *≥ 36 sev*	
									PHQ-9, item 9	0-3	*≥ 0*	
24	Boyle & McKinzie, 2021	USA	2016	632	79.6% (1,95%)	PhD	NR	ENG	DASS-42	0-3	NA	5.25
25	Brownlow et al., 2022	AUS	NR	143	61.53%	PhD	NR	NR	BRUMS-D-4	0-4	NA	2.75
									BRUMS-A-4	0-4	NA	

26	Busch et al., 2024	USA	NR	1677	59.92% (3.93%)	PhD	27.11	LS & PS	PHQ-9	0-3	0-4 mini.; 5-9 mild; *10-14 mod.; 15-19 mod-sev.; ≥ 20 sev.*	4.75
									GAD-7	0-3	0-4 mini.; 5-9 mild; *10-14 mod.; ≥ 15 sev.*	
27	Byrom & Metcalfe, 2020	GBR	2020	4274	60.17%	PhD	NR	Mixed	WEMWBS	1-5	*≥ 18*	5
									K-6	0-4	*≥ 13*	
									UCLA-3	1-3	*≥ 6*	
									Supervisor Support-6	1-5	*NA*	
28	Byrom et al., 2022	GBR	2018-2019	431	63.33 %	PhD	NR	NR	SWEMWBS	1-5	NR	5
									PSS-4	0-4	NA	
29	Caesens et al., 2014	BEL	2012	343	57.14 %	PhD	28.27 (4.43)	NR	PSS-4	1-5	NA	5
									JSQ-4	1-6	NA	
									Job Satisfaction-4	1-7	NA	
									SPOS-4	1-7	NA	
30	Carr et al., 2022	GBR	2020	484	56%	PhD[3]	NR	NR	PHQ-9	(0-3)	*≥ 10*	625
									GAD-7	(0-3)	*≥ 10*	
									AUDIT-10	(0-4)	*≥ 8*	
31	Castelló et al., 2017	GBR/ ESP	2015	282	NR	PD	35.9	Mixed	MED NORD Burnout-8<br>EE-4<br>DP-4<br>Supervisor Support-4	1-7	NA	3.75
32	Cerio et al., 2024	PHL	2021	51	NR	PhD	NR	NR	GAD-7	0-3	0-4 mini.; 5-9 mild; *10-14 mod.; ≥ 15 sev.*	2.5
33	Charles et al., 2022	USA	2016	2756	52.80%	PhD	NR	Mixed	CESD-R-20 transformed to CESD-20	0-3	*≥ 16 clinical; ≥* 28 sev.	7.25
34	Chen et al., 2024	HKG	NR	2578	50.70%	PhD	28.8	Mixed	CESD-5[4]	(0-3)	NA	4.5
									BI-EE-4[5]	(0-6)	NA	
35	Cilli et al., 2023	ITA	2022	92	59%	PhD	NR	Mixed	DASS-21	(0-3)	NA	3.75
36	Cilli et al., 2023	ITA	2022	42	43%	PD	NR	Mixed	DASS-21	(0-3)	NA	3.75
37	Cullinan et al., 2020	IRL	2018	263	61.60%	PhD	NR	NR	DASS-21*2	(0-3)	NA	5.5
38	Dhont et al., 2020	INT	2020	125	NR	PhD	NR	MED	HADS-14	0-3	*≥ 8 borderline abnormal*	3.5
39	Dhont et al., 2020	INT	2020	48	NR	PD	NR	MED	HADS-14	0-3	*≥ 8 borderline abnormal*	3.5
40	Divaris et al., 2011	GRC	2010	17	64%	PhD	30.4 (4.2)	Dental	MBI-EE-9	0-6	*≥ 26*	3.75
									MBI-DP-5	0-6	*≥ 12*	
									MBI-PA-8	0-6	*≤ 32*	
									GDES-A	1-4	NA	

[3] Postgraduate research students, which included PhDs and other research students (e.g., DClinPsy), but primarily were PhD students (personal communication, 02.12.2024).

[4] Chen et al. revised the Center for Epidemiological Disease, Depression Scale and included only five items.

[5] Chen et al. revised the Maslach Burnout Inventory and included only four items for emotional exhaustion.

41	Dong et al., 2024	USA	2021	136	69.40%	PhD	29.16 (7.4)	NR	DASS-21*2	0-3	NA	3.75
42	Doom et al., 2021	USA	2020	25	61.5% (15.4%)	PhD	30.54 (4.58)	NR	PHQ-9	0-3	0-4 mini.; 5-9 mild; *10-14 mod.; 15-19 mod-sev.; ≥ 20 sev.*	3.75
									PHQ-9, item 9	0-3	≥ 1	
									GAD-7	0-3	0-4 mini.; 5-9 mild; *10-14 mod.; ≥ 15 sev.*	
									UCLA-3	1-3	≥ 3	
43	Dumitrescu, 2016	USA	2016	153	75,2%	PhD	33.72 (8.45)	PSY	PSS-10	1-5	NA	3.75
44	Essadek et al., 2022	FRA	2020 April	90	78.89%	PhD	27.41 (4.99)	NR	PHQ-9	0-3	0-4 mini.; 5-9 mild; *10-14 mod.; 15-19 mod-sev.; ≥ 20 sev.*	5.75
									GAD-7	0-3	0-4 mini.; 5-9 mild; *10-14 mod.; ≥ 15 sev.*	
									PHQ-9, item 9	0-3	*≥ 1*	
45	Essadek et al., 2022	FRA	2020 July	62	66.13%	PhD	28.32 (7.32)	NR	PHQ-9	0-3	0-4 mini.; 5-9 mild; *10-14 mod.; 15-19 mod-sev.; ≥ 20 sev.*	5.75
									GAD-7	0-3	0-4 mini.; 5-9 mild; *10-14 mod.; ≥ 15 sev.*	
									PHQ-9, item 9	0-3	*≥ 1*	
46	Essadek et al., 2022	FRA	2020 November	58	63.79%	PhD	25.96 (3.57)	NR	PHQ-9	0-3	0-4 mini.; 5-9 mild; *10-14 mod.; 15-19 mod-sev.; ≥ 20 sev.*	5.75
									GAD-7	0-3	0-4 mini.; 5-9 mild; *10-14 mod.; ≥ 15 sev.*	
									PHQ-9, item 9	0-3	*≥ 1*	
47	Estupiñá et al., 2024	ESP	2022	1018	63.4% (0.8%)	PhD	31.7 (7.7)	Mixed	GHQ-12	0-3	GHQ2+; GHQ3+; *GHQ4+*	5.75
									PHQ-2	0-3	NR	
									GAD-2	0-3	NR	
									BDI-II, item 9	0-3	*(≥ 1)*	
									SWLS-5	1-7	NA	
									Satisfactions with Thesis Supervisor-7	1-10	NA	
48	Farrer et al., 2016	AUS	2014	116	53.44%	PhD	32.69 (9.61)	NR	PHQ-9	0-3	0-4 mini.; 5-9 mild; *10-14 mod.; 15-19 mod-sev.; ≥ 20 sev.*	6
									GAD-7	0-3	0-4 mini.; 5-9 mild; *10-14 mod.; ≥ 15 sev.*	
49	Feizi et al., 2024	CAN	2021	2475	60.8% (2.6%)	PhD	31 (6.53)	NR	MHC-SF-14	1-6	NR	4.75
									PSS-10	1-4	NA	
50	Fernández-Rodríguez et al., 2024	ESP	NR	161	65.83% (1.86%)	PhD	30.02 (6.97)	Mixed	HADS-7	0-3	*≥ 8 borderline abnormal; ≥ 11 clinical case*	5
51	Friedrich et al., 2023	DEU	2021	589	60.3% (0.8%)	PhD	28.62 (2.83)	Mixed	PHQ-2	0-3	*≥ 3*	5.75
									PSS-4	1-5	NA	
									Job Saisfaction-3	1-5	NA	
									Life Satisfaction-1	1-5	NA	
52	Frings et al., 2024	GBR	NR	19	78.94%	PhD	36.36 (14.11)	NR	PHQ-8	0-3	0-4 mini.; 5-9 mild; *10-14 mod.; 15-19 mod-sev.; ≥ 20 sev.*	3

				2	100%	PD	31 (5.65)	NR	GAD-7	0-3	0-4 mini.; 5-9 mild; *10-14 mod.; ≥ 15 sev.*	
53	Gakhal, 2024	GBR	NR	82	62%	PhD	28.48	NR	SWEMWBS	(1-5)	≤ 20 low; 20-27 mod.; ≥ 27 high	4.5
54	Gallea et al., 2021	ARG	NR	153	59%	PhD	28 (3)	LS, PS	PHQ-2	1-4	*≥ 3*	3
									GAD-2	1-4	*≥ 3*	
55	Gaudel et al., 2024	FRA	2019	165	43.1%	PhD	25.5 (2.3)	Mixed	PHQ-9	(0-3)	*≥ 10*	3.75
									GAD-7	(0-3)	*≥ 10*	
56	Gaudel et al., 2024	FRA	2020	149	NR	PhD	NR	Mixed	PHQ-9	(0-3)	*≥ 10*	3.75
									GAD-7	(0-3)	*≥ 10*	
57	Gérard & Nagels, 2013	FRA	2013	429	55.01%	PD	NR	NR	Stress-1	1-5	NA	4.25
58	Giménez Dasi et al., 2024	ESP	2023	46	NR	PhD	NR	PSY	PHQ-9	(0-3)	*≥ 10*	3.25
									GAD-7	(0-3)	*≥ 10*	
59	Gloria & Steinhardt, 2013	USA	NR	200	40.5%	PD	32.06 (3.71)	Mixed	CESD-20	0-3	*≥ 16*	4.5
									STAI-T-20	1-4	NA	
									PSS-10	0-4	NA	
60	Guo et al., 2024	CHN	2023	248	50.4%	PhD	31.4 (3.93)	NR	PHQ-9	0-3	0-4 mini.; 5-9 mild; *10-14 mod.; 15-19 mod-sev.; ≥ 20 sev.*	4
									GAD-7	0-3	0-4 mini.; 5-9 mild; *10-14 mod.; ≥ 15 sev.*	
61	Hanitzsch et al., 2024	INT	2022	241	NR	PhD	NR	COM	K-10	0-4	0-5 low; 6-11 mod.; 12-19 high; ≥ 20 very high	5
									PHQ-8	0-3	0-4 mini.; 5-9 mild; *10-14 mod.; 15-19 mod-sev.; ≥ 20 sev.*	
									GAD-7	0-3	0-4 mini.; 5-9 mild; *10-14 mod.; ≥ 15 sev.*	
62	Hanitzsch et al., 2024	INT	2022	79	NR	PD	36.4 (5.99)	COM	K-10	0-4	0-5 low; 6-11 mod.; 12-19 high; ≥ 20 very high	5
									PHQ-8	0-3	0-4 mini.; 5-9 mild; *10-14 mod.; 15-19 mod-sev.; ≥ 20 sev.*	
									GAD-7	0-3	0-4 mini.; 5-9 mild; *10-14 mod.; ≥ 15 sev.*	
63	Hariharan et al., 2024	USA	NR	NR	NR	PhD	NR	NR	BAI-21	(0-3)	NR	3.5
									PSS-10	(0-4)	NA	
64	Healthy Minds Study, 2007-2013	USA	2007	555	55.31% (0.18%)	PhD	30.07 (6.87)	Mixed	PHQ-9	0-3	0-4 mini.; 5-9 mild; *10-14 mod.; 15-19 mod-sev.; ≥ 20 sev.*	7.25
									PHQ-9, item 9	0-3	*≥ 1*	
									GAD-7[6]	0-3	0-4 mini.; 5-9 mild; *10-14 mod.; ≥ 15 sev.*	
65	Healthy Minds Study, 2007-2013	USA	2009	500	58.4%	PhD	29.47 (6.38)	Mixed	PHQ-9	0-3	0-4 mini.; 5-9 mild; *10-14 mod.; 15-19 mod-sev.; ≥ 20 sev.*	7.25
									PHQ-9, item 9	0-3	*≥ 1*	
									GAD-7[7]	0-3	0-4 mini.; 5-9 mild; *10-14 mod.; ≥ 15 sev.*	
66	Healthy Minds Study, 2007-2013	USA	2010	1411	49.32% (0.28%)	PhD	29.33 (6.35)	Mixed	PHQ-9	0-3	0-4 mini.; 5-9 mild; *10-14 mod.; 15-19 mod-sev.; ≥ 20 sev.*	7.25

[6] Only given for the year 2013, as the GAD-7 utilized in the years before lacked the last response category "3 – Nearly every day"

[7] Only given for the year 2013, as the GAD-7 utilized in the years before lacked the last response category "3 – Nearly every day"

									PHQ-9, item 9	0-3	*≥ 1*	
									GAD-7[8]	0-3	0-4 mini.; 5-9 mild; *10-14 mod.; ≥ 15 sev.*	
67	Healthy Minds Study, 2007-2013	USA	2011	242	57.85% (0.41%)	PhD	32.07 (8.22)	Mixed	PHQ-9	0-3	0-4 mini.; 5-9 mild; *10-14 mod.; 15-19 mod-sev.; ≥ 20 sev.*	5.75
									PHQ-9, item 9	0-3	*≥ 1*	
									GAD-7[9]	0-3	0-4 mini.; 5-9 mild; *10-14 mod.; ≥ 15 sev.*	
68	Healthy Minds Study, 2007-2013	USA	2012	1477	59.71% (0.27%)	PhD	30.26 (6.92)	Mixed	PHQ-9	0-3	0-4 mini.; 5-9 mild; *10-14 mod.; 15-19 mod-sev.; ≥ 20 sev.*	7.25
									PHQ-9, item 9	0-3	*≥ 1*	
									GAD-7[10]	0-3	0-4 mini.; 5-9 mild; *10-14 mod.; ≥ 15 sev.*	
69	Healthy Minds Study, 2007-2013	USA	2013	1356	55.97% (0.07%)	PhD	31.48 (7.53)	Mixed	PHQ-9	0-3	0-4 mini.; 5-9 mild; *10-14 mod.; 15-19 mod-sev.; ≥ 20 sev.*	7.25
									PHQ-9, item 9	0-3	*≥ 1*	
									GAD-7	0-3	0-4 mini.; 5-9 mild; *10-14 mod.; ≥ 15 sev.*	
									SCOFF-5	0-1	*≥ 2*	
70	Healthy Minds Study, 2013-2014	USA	2014	1380	50.50% (0.14%)	PhD	29.1 (6.1)	Mixed	PHQ-9	0-3	0-4 mini.; 5-9 mild; *10-14 mod.; 15-19 mod-sev.; ≥ 20 sev.*	7.25
									PHQ-9, item 9	0-3	*≥ 1*	
									GAD-7	0-3	0-4 mini.; 5-9 mild; *10-14 mod.; ≥ 15 sev.*	
									SCOFF-5	0-1	*≥ 2*	
71	Healthy Minds Study, 2014-2015	USA	2014-2015	2063	47.79% (0.09%)	PhD	28.33 (5.54)	Mixed	PHQ-9	0-3	0-4 mini.; 5-9 mild; *10-14 mod.; 15-19 mod-sev.; ≥ 20 sev.*	7.25
									PHQ-9, item 9	0-3	*≥ 1*	
									GAD-7	0-3	0-4 mini.; 5-9 mild; *10-14 mod.; ≥ 15 sev.*	
									SCOFF-5	0-1	*≥ 2*	
72	Healthy Minds Study, 2015-2016	USA	2015-2016	3227	53.14% (0.96%)	PhD	28.23 (5.81)	Mixed	PHQ-9	0-3	0-4 mini.; 5-9 mild; *10-14 mod.; 15-19 mod-sev.; ≥ 20 sev.*	7.25
									PHQ-9, item 9	0-3	*≥ 1*	
									GAD-7	0-3	0-4 mini.; 5-9 mild; *10-14 mod.;* ≥ 15 sev.	
									SCOFF-5	0-1	*≥ 2*	
73	Healthy Minds Study, 2016-2017	USA	2016	1306	64.01% (0.61%)	PhD	28.39 (5.74)	Mixed	PHQ-9	0-3	0-4 mini.; 5-9 mild; *10-14 mod.; 15-19 mod-sev.; ≥ 20 sev.*	6.25
									PHQ-9, item 9	0-3	*≥ 1*	
									GAD-7	0-3	0-4 mini.; 5-9 mild; *10-14 mod.;* ≥ 15 sev.	
									SCOFF-5	0-1	*≥ 2*	

[8] Only given for the year 2013, as the GAD-7 utilized in the years before lacked the last response category “3 – Nearly every day”

[9] Only given for the year 2013, as the GAD-7 utilized in the years before lacked the last response category “3 – Nearly every day”

[10] Only given for the year 2013, as the GAD-7 utilized in the years before lacked the last response category “3 – Nearly every day”

									AUDIT-10[11]	0-4	0-7 Zone I; 8-15 Zone II; 16-19 Zone III; ≥ 20 Zone IV	
74	Healthy Minds Study, 2016-2017	USA	2017	2593	59.04% (1.04%)	PhD	28.29 (7.18)	Mixed	PHQ-9	0-3	0-4 mini.; 5-9 mild; *10-14 mod.; 15-19 mod-sev.; ≥ 20 sev.*	6.25
									PHQ-9, item 9	0-3	*≥ 1*	
									GAD-7	0-3	0-4 mini.; 5-9 mild; *10-14 mod.;* ≥ 15 sev.	
									SCOFF-5	0-1	*≥ 2*	
									AUDIT-10[12]	0-4	0-7 Zone I; 8-15 Zone II; 16-19 Zone III; ≥ 20 Zone IV	
75	Healthy Minds Study, 2017-2018	USA	2017	1591	52.16% (0.69%)	PhD	28.1 (6.32)	Mixed	PHQ-9	0-3	0-4 mini.; 5-9 mild; *10-14 mod.; 15-19 mod-sev.; ≥ 20 sev.*	6.25
									PHQ-9, item 9	0-3	*≥ 1*	
									GAD-7	0-3	0-4 mini.; 5-9 mild; *10-14 mod.;* ≥ 15 sev.	
									SCOFF-5	0-1	*≥ 2*	
									AUDIT-10[13]	0-4	0-7 Zone I; 8-15 Zone II; 16-19 Zone III; ≥ 20 Zone IV	
76	Healthy Minds Study, 2017-2018	USA	2018	4589	53.99% (0.85%)	PhD	28.82 (6.46)	Mixed	PHQ-9	0-3	0-4 mini.; 5-9 mild; *10-14 mod.; 15-19 mod-sev.; ≥ 20 sev.*	6.25
									PHQ-9, item 9	0-3	*≥ 1*	
									GAD-7	0-3	0-4 mini.; 5-9 mild; *10-14 mod.;* ≥ 15 sev.	
									SCOFF-5	0-1	*≥ 2*	
									AUDIT-10[14]	0-4	0-7 Zone I; 8-15 Zone II; 16-19 Zone III; ≥ 20 Zone IV	
77	Healthy Minds Study, 2018-2019	USA	2018	1763	59.16% (0.9%)	PhD	28.21 (6.15)	Mixed	PHQ-9	0-3	0-4 mini.; 5-9 mild; *10-14 mod.; 15-19 mod-sev.; ≥ 20 sev.*	6.25
									PHQ-9, item 9	0-3	*≥ 1*	
									GAD-7	0-3	0-4 mini.; 5-9 mild; *10-14 mod.; ≥ 15 sev.*	
									SCOFF-5	0-1	*≥ 2*	
									AUDIT-10[15]	0-4	0-7 Zone I; 8-15 Zone II; 16-19 Zone III; ≥ 20 Zone IV	
78	Healthy Minds Study, 2018-2019	USA	2019	2469	61.15% (1.37%)	PhD	29.76 (7.68)	Mixed	PHQ-9	0-3	0-4 mini.; 5-9 mild; *10-14 mod.; 15-19 mod-sev.; ≥ 20 sev.*	6.25
									PHQ-9, item 9	0-3	*≥ 1*	
									GAD-7	0-3	0-4 mini.; 5-9 mild; *10-14 mod.; ≥ 15 sev.*	

[11] AUDIT was an elective module in the HMS, and participants could choose to skip questions. Only full questionnaires were considered valid. For the year 2016, data of the AUDIT is based only on n = 119 from the overall sample of n = 1306, 9.11 %. And for the year 2017 based only on n = 30 from the overall sample of n = 2593, 1.15 %

[12] AUDIT was an elective module in the HMS, and participants could choose to skip questions. Only full questionnaires were considered valid. For the year 2016, data of the AUDIT is based only on n = 119 from the overall sample of n = 1306, 9.11 %. And for the year 2017 based only on n = 30 from the overall sample of n = 2593, 1.15 %

[13] Data on the AUDIT for the year of 2017 is not provided. Data from the AUDIT for 2018 is based on n = 492 from the overall sample of n = 4589, 10.72 %.

[14] Data on the AUDIT for the year of 2017 is not provided. Data from the AUDIT for 2018 is based on n = 492 from the overall sample of n = 4589, 10.72 %.

[15] Data on the AUDIT for the year of 2018 is based on n = 40 from the overall sample of n = 1763, 2.26 %. Data from the AUDIT for 2019 is based on n = 89 from the overall sample of n = 2469, 3.60 %.

									SCOFF-5	0-1	*≥ 2*	
									AUDIT-10[16]	0-4	0-7 Zone I; 8-15 Zone II; 16-19 Zone III; ≥ 20 Zone IV	
79	Healthy Minds Study, 2019-2020	USA	2019	2372	63.28% (1.93%)	PhD	28.63 (6.43)	Mixed	PHQ-9	0-3	0-4 mini.; 5-9 mild; *10-14 mod.; 15-19 mod-sev.; ≥ 20 sev.*	6.25
									PHQ-9, item 9	0-3	*≥ 1*	
									GAD-7	0-3	0-4 mini.; 5-9 mild; *10-14 mod.;* ≥ 15 sev.	
									SCOFF-5	0-1	*≥ 2*	
									AUDIT-10[17]	0-4	0-7 Zone I; 8-15 Zone II; 16-19 Zone III; ≥ 20 Zone IV	
80	Healthy Minds Study, 2019-2020	USA	2020	3959	58.17% (1.43%)	PhD	29.27 (6.7)	Mixed	PHQ-9	0-3	0-4 mini.; 5-9 mild; *10-14 mod.; 15-19 mod-sev.; ≥ 20 sev.*	6.25
									PHQ-9, item 9	0-3	*≥ 1*	
									GAD-7	0-3	0-4 mini.; 5-9 mild; *10-14 mod.;* ≥ 15 sev.	
									SCOFF-5	0-1	*≥ 2*	
									AUDIT-10[18]	0-4	0-7 Zone I; 8-15 Zone II; 16-19 Zone III; ≥ 20 Zone IV	
81	Healthy Minds Study, 2020-2021	USA	2020	2660	64.88% (1.12%)	PhD	29.59 (8.43)	Mixed	PHQ-9	0-3	0-4 mini.; 5-9 mild; *10-14 mod.; 15-19 mod-sev.; ≥ 20 sev.*	6.25
									PHQ-9, item 9	0-3	*≥ 1*	
									GAD-7	0-3	0-4 mini.; 5-9 mild; *10-14 mod.;* ≥ 15 sev.	
									SCOFF-5	0-1	*≥ 2*	
									AUDIT-10[19]	0-4	0-7 Zone I; 8-15 Zone II; 16-19 Zone III; ≥ 20 Zone IV	
									UCLA-3	1-3	3-5 mod. Lonely; ≥ 6 lonely	
82	Healthy Minds Study, 2020-2021	USA	2021	6424	60.02% (1.4%)	PhD	29.16 (6.96)	Mixed	PHQ-9	0-3	0-4 mini.; 5-9 mild; *10-14 mod.; 15-19 mod-sev.; ≥ 20 sev.*	6.25
									PHQ-9, item 9	0-3	*≥ 1*	
									GAD-7	0-3	0-4 mini.; 5-9 mild; *10-14 mod.;* ≥ 15 sev.	
									SCOFF-5	0-1	*≥ 2*	
									AUDIT-10[20]	0-4	0-7 Zone I; 8-15 Zone II; 16-19 Zone III; ≥ 20 Zone IV	
									UCLA-3	1-3	3-5 mod. Lonely; ≥ 6 lonely	
83	Healthy Minds Study, 2021-2022	USA	2021	855	61.4% (1.05%)	PhD	28.53 (6.98)	Mixed	PHQ-9	0-3	0-4 mini.; 5-9 mild; *10-14 mod.; 15-19 mod-sev.; ≥ 20 sev.*	6.25
									PHQ-9, item 9	0-3	*≥ 1*	
									GAD-7	0-3	0-4 mini.; 5-9 mild; *10-14 mod.;* ≥ 15 sev.	

[16] Data on the AUDIT for the year of 2018 is based on n = 40 from the overall sample of n = 1763, 2.26 %. Data from the AUDIT for 2019 is based on n = 89 from the overall sample of n = 2469, 3.60 %.

[17] Data on the AUDIT for the year of 2019 is based on n = 98 from the overall sample of n = 2372, 4.13 %. Data from the AUDIT for 2020 is based on n = 55 from the overall sample of n = 3959, 1.39 %.

[18] Data on the AUDIT for the year of 2019 is based on n = 98 from the overall sample of n = 2372, 4.13 %. Data from the AUDIT for 2020 is based on n = 55 from the overall sample of n = 3959, 1.39 %.

[19] Data on the AUDIT for the year of 2020 is based on n = 141 from the overall sample of n = 2660, 5.3 %. Data from the AUDIT for 2021 is based on n = 143 from the overall sample of n = 6424, 2.22 %.

[20] Data on the AUDIT for the year of 2020 is based on n = 141 from the overall sample of n = 2660, 5.3 %. Data from the AUDIT for 2021 is based on n = 143 from the overall sample of n = 6424, 2.22 %.

									SCOFF-5	0-1	*≥ 2*	
									AUDIT-10[21]	0-4	0-7 Zone I; 8-15 Zone II; 16-19 Zone III; ≥ 20 Zone IV	
									UCLA-3	1-3	3-5 mod. Lonely; ≥ 6 lonely	
84	Healthy Minds Study, 2021-2022	USA	2022	4622	61.12% (2.29%)	PhD	29.03 (6.48)	Mixed	PHQ-9	0-3	0-4 mini.; 5-9 mild; *10-14 mod.; 15-19 mod-sev.; ≥ 20 sev.*	6.25
									PHQ-9, item 9	0-3	*≥ 1*	
									GAD-7	0-3	0-4 mini.; 5-9 mild; *10-14 mod.;* ≥ 15 sev.	
									SCOFF-5	0-1	*≥ 2*	
									AUDIT-10[22]	0-4	0-7 Zone I; 8-15 Zone II; 16-19 ZONE III; ≥ 20 Zone IV	
									UCLA-3	1-3	3-5 mod. Lonely; ≥ 6 lonely	
85	Healthy Minds Study, 2022-2023	USA	2022	1546	71.47% (1.81%)	PhD	29.31 (9.02)	Mixed	PHQ-9	0-3	0-4 mini.; 5-9 mild; *10-14 mod.; 15-19 mod-sev.; ≥ 20 sev.*	6.25
									PHQ-9, item 9	0-3	*≥ 1*	
									GAD-7	0-3	0-4 mini.; 5-9 mild; *10-14 mod.;* ≥ 15 sev.	
									SCOFF-5	0-1	*≥ 2*	
									AUDIT-C[23]	0-4	*≥ 3 for women*<br>*≥ 4 for men*	
									UCLA-3	1-3	3-5 mod. Lonely; ≥ 6 lonely	
86	Healthy Minds Study, 2022-2023	USA	2023	2490	67.14% (3.29%)	PhD	29.97 (7.61)	Mixed	PHQ-9	0-3	0-4 mini.; 5-9 mild; *10-14 mod.; 15-19 mod-sev.; ≥ 20 sev.*	6.25
									PHQ-9, item 9	0-3	*≥ 1*	
									GAD-7	0-3	0-4 mini.; 5-9 mild; *10-14 mod.;* ≥ 15 sev.	
									SCOFF-5	0-1	*≥ 2*	
									AUDIT-C[24]	0-4	*≥ 3 for women*<br>*≥ 4 for men*	
									UCLA-3	1-3	3-5 mod. Lonely; ≥ 6 lonely	
87	Healthy Minds Study, 2023-2024	USA	2023	2690	67.54% (2.19%)	PhD	29.02 (7.76)	Mixed	PHQ-9	0-3	0-4 mini.; 5-9 mild; *10-14 mod.; 15-19 mod-sev.; ≥ 20 sev.*	6.25
									PHQ-9, item 9	0-3	*≥ 1*	
									GAD-7	0-3	0-4 mini.; 5-9 mild; *10-14 mod.;* ≥ 15 sev.	
									SCOFF-5	0-1	*≥ 2*	
									AUDIT-C[25]	0-4	*≥ 3 for women*<br>*≥ 4 for men*	

[21] Data on the AUDIT for the year of 2021 is based on n = 21 from the overall sample of n = 855, 2.45 %. Data from the AUDIT for 2022 is based on n = 1817 from the overall sample of n = 4622, 39.31 %.

[22] Data on the AUDIT for the year of 2021 is based on n = 21 from the overall sample of n = 855, 2.45 %. Data from the AUDIT for 2022 is based on n = 1817 from the overall sample of n = 4622, 39.31 %.

[23] We decided to calculate the AUDIT-C instead of the AUDIT to mitigate dropouts. Data on the AUDIT-C for the year 2022 is based on n = 47 from the overall sample n = 1546, 3.04 %. Data on the AUDIT-C for the year 2023 is based on n = 244 from the overall sample of n = 2490, 9.79 %.

[24] We decided to calculate the AUDIT-C instead of the AUDIT to mitigate dropouts. Data on the AUDIT-C for the year 2022 is based on n = 47 from the overall sample n = 1546, 3.04 %. Data on the AUDIT-C for the year 2023 is based on n = 244 from the overall sample of n = 2490, 9.79 %.

[25] We decided to calculate the AUDIT-C instead of the AUDIT to mitigate dropouts. Data on the AUDIT-C for the year 2023 is based on n = 369 from the overall sample n = 2690, 13.71 %. Data on the AUDIT-C for the year 2024 is based on n = 336 from the overall sample of n = 2212, 15.19 %.

									UCLA-3	1-3	3-5 mod. Lonely; ≥ 6 lonely	
88	Healthy Minds Study, 2023-2024	USA	2024	2212	62.07% (2.66%)	PhD	30,13 (7.85)	Mixed	PHQ-9	0-3	0-4 mini.; 5-9 mild; *10-14 mod.; 15-19 mod-sev.; ≥ 20 sev.*	6.25
									PHQ-9, item 9	0-3	*≥ 1*	
									GAD-7	0-3	0-4 mini.; 5-9 mild; *10-14 mod.; ≥* 15 sev.	
									SCOFF-5	0-1	*≥ 2*	
									AUDIT-C[26]	0-4	*≥ 3 for women*<br>*≥ 4 for men*	
									UCLA-3	1-3	3-5 mod. Lonely; ≥ 6 lonely	
89	Healthy Minds Study, 2023-2024	USA	2024	198	69.63% (2.61%)	PD	39.25 (10.08)	Mixed	PHQ-9	0-3	0-4 mini.; 5-9 mild; *10-14 mod.; 15-19 mod-sev.; ≥ 20 sev.*	5.25
									PHQ-9, item 9	0-3	*≥ 1*	
									GAD-7	0-3	0-4 mini.; 5-9 mild; *10-14 mod.; ≥* 15 sev.	
									SCOFF-5	0-1	*≥ 2*	
90	Healthy Minds Study, 2024-2025	USA	2024	1673	66.84% (2.1%)	PhD	29.82 (8.41)	Mixed	PHQ-9	0-3	0-4 mini.; 5-9 mild; *10-14 mod.; 15-19 mod-sev.; ≥ 20 sev.*	6.25
									PHQ-9, item 9	0-3	*≥ 1*	
									GAD-7	0-3	0-4 mini.; 5-9 mild; *10-14 mod.; ≥* 15 sev.	
									SDE	0-1	*≥ 2*	
									AUDIT-C[27]	0-4	*≥ 3 for women*<br>*≥ 4 for men*	
									UCLA-3	1-3	3-5 mod. Lonely; ≥ 6 lonely	
91	Healthy Minds Study, 2024-2025	USA	2025	2799	63.84% (2.63%)	PhD	26.62 (6.42)	Mixed	PHQ-9	0-3	0-4 mini.; 5-9 mild; *10-14 mod.; 15-19 mod-sev.; ≥ 20 sev.*	6.25
									PHQ-9, item 9	0-3	*≥ 1*	
									GAD-7	0-3	0-4 mini.; 5-9 mild; *10-14 mod.; ≥* 15 sev.	
									SDE	0-1	*≥ 2*	
									AUDIT-C[28]	0-4	*≥ 3 for women*<br>*≥ 4 for men*	
									UCLA-3	1-3	3-5 mod. Lonely; ≥ 6 lonely	
92	Healthy Minds Study, 2024-2025	USA	2024	144	58.69% (3.62%)	PD	39.77	Mixed	PHQ-9	0-3	0-4 mini.; 5-9 mild; *10-14 mod.; 15-19 mod-sev.; ≥ 20 sev.*	4.25
									PHQ-9, item 9	0-3	*≥ 1*	
									GAD-7	0-3	0-4 mini.; 5-9 mild; *10-14 mod.; ≥* 15 sev.	
									SDE	0-1	*≥ 2*	
93	Healthy Minds Study, 2024-2025	USA	2025	38	75.67% (2.7%)	PD	42.5	Mixed	PHQ-9	0-3	0-4 mini.; 5-9 mild; *10-14 mod.; 15-19 mod-sev.; ≥ 20 sev.*	4.25

[26] We decided to calculate the AUDIT-C instead of the AUDIT to mitigate dropouts. Data on the AUDIT-C for the year 2023 is based on n = 369 from the overall sample n = 2690, 13.71 %. Data on the AUDIT-C for the year 2024 is based on n = 336 from the overall sample of n = 2212, 15.19 %.

[27] We decided to calculate the AUDIT-C instead of the AUDIT to mitigate dropouts. Data on the AUDIT-C for the year 2023 is based on n = 369 from the overall sample n = 2690, 13.71 %. Data on the AUDIT-C for the year 2024 is based on n = 336 from the overall sample of n = 2212, 15.19 %.

[28] We decided to calculate the AUDIT-C instead of the AUDIT to mitigate dropouts. Data on the AUDIT-C for the year 2023 is based on n = 369 from the overall sample n = 2690, 13.71 %. Data on the AUDIT-C for the year 2024 is based on n = 336 from the overall sample of n = 2212, 15.19 %.

									PHQ-9, item 9	0-3	≥ *1*	
									GAD-7	0-3	0-4 mini.; 5-9 mild; *10-14 mod.;* ≥ 15 sev.	
									SDE	0-1	≥ *2*	
94	Heinrich, 1979	USA	NR	68	NR	PhD	NR	NR	STAI-40(20)	(1-4)	NA	2.75
95	Heming et al., 2024	DEU	2023	267	64%	PhD	30.15 (6.01)	Mixed	GHQ-12	0-0-1-1 0-3	GHQ2+, GHQ3+, GHQ4+	4.25
									PSS-10	0-4	NA	
96	Heming et al., 2024	DEU	2024	244	60.7%	PhD	30.35 (7)	Mixed	GHQ-12	0-0-1-1 0-3	GHQ2+, GHQ3+, GHQ4+	4.25
									PSS-10	0-4	NA	
97	Herrell & Foster, 2024	USA	NR	34	41.17% (2.94%)	PhD	33.72 (13.66)	Mixed	DASS-21*2	0-3	NA	2.75
98	Hill, 2010	USA	NR	64	64.1%	PhD	NR	PSY	PSS-10	0-4	NA	4.5
									MSPSS-12	1-7	NA	
99	Hindman et al., 2015	USA	NR	6	100%	PhD	26.33 (2.16)	NR	DASS-21*2	0-3	NA	2.75
									SWLS-5	1-7	NA	
100	Hish et al., 2019	USA	NR	69	60.9%	PhD	26.5 (2.3)	LS	PHQ-9	0-3	NR	4
									GSI-R-20	1-7	NA	
									SBI-9	1-6	NA	
									MSPSS-12	1-7	NA	
									AWAI-30	1-5	NA	
101	Hobaica et al., 2021	USA	2019	912	81.5%	PhD	27.21 (3.38)	PSY	PHQ-9	0-3	1-4 mini.; 5-9 mild; *10-14 mod.; 15-19 mod-sev.;* ≥ *20 sev.*	5
									GAD-7	0-3	0-4 min.; 5-9 mild; *10-14 mod.,* ≥ *15 sev.*	
									DAST-10	0-10	0 no; 1-2 low; 3-5 mod.; 6-8 substantial; 9-10 sev.	
									AUDIT-C	1-4	≥ 3 for women<br>≥ 4 for men	
									CIDI	NA	NA	
102	Hogg, 2023	USA	2022	209	77% (5.3%)	PhD	35.14 (8.23)	SW	CESD-20	0-3	≥ *16 clinical;* ≥ 28 sev.	5
									MSSS-33	0-5	NA	
									SPOS-8	0-7	NA	
103	Hu et al., 2024	CHN	NR	505	42.38%	PD	32.87 (3.46)	NR	PHQ-9	0-3	0-4 mini.; 5-9 mild; *10-14 mod.; 15-19 mod-sev.;* ≥ *20 sev.*	6
									PHQ-9, item 9	0-3	≥ *1*	
									PSS-10	0-4	NA	
104	Hulac et al., 2024	USA	NR	95	NR	PhD	NR	PSY	PHQ-9	0-3	0-4 mini.; 5-9 mild; *10-14 mod.; 15-19 mod-sev.;* ≥ *20 sev.*	3.25
									GAD-7	0-3	0-4 mini.; 5-9 mild; *10-14 mod.;* ≥ *15 sev.*	
105	ISWS, 2021	BLG	2020	301	61.13%	PhD	29.09 (5.98)	Mixed	CESD-8	0-3	≥ 7	5
106	ISWS, 2021	CAN	2020	932	60.73%	PhD	30.62 (6.81)	Mixed	CESD-8	0-3	≥ 7	5
107	ISWS, 2021	CHE	2020	107	75.71%	PhD	31.31 (7.12)	Mixed	CESD-8	0-3	≥ 7	3

108	ISWS, 2021	CYP	2020	25	80%	PhD	30.48 (5.93)	Mixed	CESD-8	0-3	≥ 7	3
109	ISWS, 2021	CZE	2020	387	60.72%	PhD	28.75 (4.4)	Mixed	CESD-8	0-3	≥ 7	5
110	ISWS, 2021	DEU	2020	377	61.81%	PhD	26.69 (5.79)	Mixed	CESD-8	0-3	≥ 7	5
111	ISWS, 2021	DNK	2020	189	77.77%	PhD	34.67 (6.64)	Mixed	CESD-8	0-3	≥ 7	3
112	ISWS, 2021	ESP	2020	137	56.20%	PhD	36.58 (10.72)	Mixed	CESD-8	0-3	≥ 7	3
113	ISWS, 2021	FIN	2020	19	73.64%	PhD	38.52 (12.49)	Mixed	CESD-8	0-3	≥ 7	3
114	ISWS, 2021	FRA	2020	264	67.42%	PhD	25.5 (5.93)	Mixed	CESD-8	0-3	≥ 7	3
115	ISWS, 2021	GBR	2020	82	62.19%	PhD	38.13 (11.17)	Mixed	CESD-8	0-3	≥ 7	3
116	ISWS, 2021	GRC	2020	29	65.51%	PhD	31.13 (8.7)	Mixed	CESD-8	0-3	≥ 7	3
117	ISWS, 2021	HUN	2020	507	75.14%	PhD	22.59 (4.69)	Mixed	CESD-8	0-3	≥ 7	5
118	ISWS, 2021	ISL	2020	37	67.56%	PhD	40.05 (11.1)	Mixed	CESD-8	0-3	≥ 7	3
119	ISWS, 2021	ITA	2020	125	50.4%	PhD	28.59 (5.61)	Mixed	CESD-8	0-3	≥ 7	3
120	ISWS, 2021	NLD	2020	50	52%	PhD	28.22 (4.68)	Mixed	CESD-8	0-3	≥ 7	3
121	ISWS, 2021	NOR	2020	132	62.12%	PhD	34.65 (8.99)	Mixed	CESD-8	0-3	≥ 7	3
122	ISWS, 2021	PRT	2020	56	67.85%	PhD	34.84 (8.78)	Mixed	CESD-8	0-3	≥ 7	3
123	ISWS, 2021	ROU	2020	3	66.66%	PhD	24 (4)	Mixed	CESD-8	0-3	≥ 7	3
124	ISWS, 2021	RUS	2020	31	67.74%	PhD	25.16 (1.41)	Mixed	CESD-8	0-3	≥ 7	3
125	ISWS, 2021	SVK	2020	154	57.14%	PhD	23.92 (3.2)	Mixed	CESD-8	0-3	≥ 7	3
126	ISWS, 2021	SWE	2020	116	58.62%	PhD	31.36 (5.91)	Mixed	CESD-8	0-3	≥ 7	3
127	ISWS, 2021	TUR	2020	658	65.04%	PhD	33.11 (6.53)	Mixed	CESD-8	0-3	≥ 7	5
128	ISWS, 2021	USA	2020	5	80%	PhD	25 (4.74)	Mixed	CESD-8	0-3	≥ 7	3
129	ISWS, 2021	ZAF	2020	89	53.93%	PhD	33.85 (7.56)	Mixed	CESD-8	0-3	≥ 7	3
130	Jackman et al., 2023	GBR		200	70.5%	PhD	29.94	Mixed	WEMWBS	1-5	NR	3.75

			2020-2021		(2%)				K6	1-5	≥ 13	
131	Jamshidi et al., 2017	IRN	2014	280	NR	PhD	NR	LS	GHQ-28[29]	0-3	≥ 6	4.25
132	Jing, 2021	HKG	2021	511	47%	PhD	NR	Mixed	DASS-21-D*2	0-3	NA	5
									GSI-AS-13	1-7	NA	
									WHOQOL-BREF-1	0-5	NA	
133	Kaufman, 2004	USA	2003	41	82.92%	PhD	40.95	PSY	PSS-14	(0-4)	NA	3.5
134	Kılıç & Karahan, 2023	TUR	NR	125	NR	PhD	NR	NR	CCAPS-62	0-4	NA	3.25
135	Kogien et al., 2023	BRA	2021	57	78.94%	PhD	36.29 (8.22)	NR	CIDI	0-1	≥ 1	5
136	Kosh, 2014	USA	2013	116	70.2%	PhD	NR	Mixed	MBI-SS-16<br>MBI-SS-EE-5<br>MBI-SS-DP-5<br>MBI-SS-PA-6	1-6	NA	4
									AWAI-R-14	1-5	NA	
137	Kowalczyk et al., 2021	POL	NR	576	55.6%	PhD	28	Mixed	GHQ-28	0-0-1-1	≥ 5	6
138	Krieger et al., 2025	ESP	2022	1265	58.42% (0.71%)	PhD	32.36 (8.2)	Mixed	CESD-R	0-4	*≥ 16*	5.75
									GAD-7	0-3	*≥ 10*	
									SWLS	1-7	NA	
139	Kusurkar et al., 2020	NLD	2018	464	80%	PhD	29	MED	MBI-SS-15	0-6	NA	5.25
140	Larcombe et al., 2022	AUS	2018	1017	62.2 (3.1%)	PhD	NR	Mixed	DASS-21	(0-3)	NA	5.5
141	Lento & Wick, 2024	USA<br>CAN	2021	116	61%	PhD	32 (NR)	BMA	11-item Job Stress Measure	-2 - + 2	NA	3.5
									MBI-12[30]	-3 - + 3	NA	
142	Levecque et al., 2017	BEL	2013	3659	52.01%	PhD	28.37 (4.67)	Mixed	GHQ-12	0-0-1-1	GHQ2+, GHQ3+, *GHQ4+*	5.75
143	Li & Su, 2024	CHN	NR	308	62.33%	PhD	NR	NR	GAD-7	0-3	0-4 mini.; 5-9 mild; 10-14 mod.; ≥ 15 sev.	5
144	Liu et al., 2019	CHN	2017	325	60.3%	PhD	31.1 (5.3)	MED	PHQ-9	0-3	0-4 mini.; 5-9 mild; *10-14 mod.; 15-19 mod-sev.; ≥ 20 sev.*	6
									GAD-7	0-3	0-4 mini.; 5-9 mild; *10-14 mod.; ≥ 15 sev.*	
									AWAI-S-30	1-5	NA	
145	Liu et al., 2020	CHN	2017	50	NR	PhD	NR	Mixed	PHQ-9	0-3	*≥ 10*	4.75
									GAD-7	0-3	*≥ 10*	
146	Lowe, 2015	USA	NR	203	NR	PhD	NR	PSY	PWS-5	1-6	NA	3.75
147	LT et al., 2022	IND	2019-2020	240	70.4 %	PhD	NR	LS, PS	PHQ-9	0-3	0-4 mini.; 5-9 mild; *10-14 mod.; 15-19 mod-sev.; ≥ 20 sev.*	7
									PHQ-9, item 9	0-3	*≥ 1*	

[29] As validated by Yaghoobi N, Nasr M Shah Mohammadi D. Study of epidemiology of mental disorders in urban and rural areas in Someesara in Gilan. Thought and Behavior Journal. 1995; 1(4): 55-65.

[30] This is also a modified version; items per sub-scale are unclear.

148	Lubega et al., 2023	USA	2020	80	43.75%	PhD	NR	LS	PHQ-8	0-3	0-4 mini.; 5-9 mild; *10-14 mod.; 15-19 mod-sev.; ≥ 20 sev.*	6
									GAD-7	0-3	0-4 mini.; 5-9 mild; *10-14 mod.; ≥ 15 sev.*	
									GLS-5	1-5	NA	
149	MacKinnon et al. 2024	CAN	2020	712	69.38% (1.54%)	PhD	32.57 (7.52)	NR	K-10	1-5	10-10 well; 20-24 mild; 25-29 mod.; 30-50 severe	5
150	Mao et al., 2022	CHN	2022	567	56.44%	PhD	NR	Mixed	STAI-40	1-4	NA	4.5
151	Marais et al., 2018	FRA	2016	136	73.5%	PhD	NR	LS	DASS-21	0-3	NA	4
									WEMWBS	1-5	NR	
152	Martinez et al., 2024	USA	2020	422	60.7%	PhD	28.8 (5.17)	NR	PHQ-8	(0-3)	*≥ 10*	4
153	Mavrogalou-Foti et al., 2024	GBR	NR	141	68.8%	PhD	31.21 (8.01)	NR	DASS-21*2	0-3	NA	4.25
154	McGregor et al., 2008	USA	NR	10	70%	PhD	NR	PSY	PSS-14	0-4	NA	3.75
155	Miller & Orsillo, 2020	USA	NR	436	73.8% (3.7%)	PhD	30.51 (8.62)	Mixed	DASS-21	0-3	NA	4.75
156	Mills et al., 2024	AUS	2022	302	72.51%	PhD	34.12 (10.32)	NR	PHQ-9	0-3	NR	4.75
									GAD-7	0-3	NR	
									UCLA-20	0-3	NA	
									QSDI-25	(1-5)	NA	
									SBQ-R	3-18	NR	
157	Moate et al., 2019	USA	NR	528	46.64%	PhD	30.5 (8.66)	Mixed	PSS-14	0-4	NA	4.75
									SWLS-5	1-7	NA	
158	Müller et al., 2022	DEU	2019	NR	NR	PhD	NR	NR	PSS-10	0-4	NA	5
									PHQ-2	0-3	*≥ 3*	
									GAD-2	0-3	*≥ 3*	
159	Muro et al., 2022	ESP	2022	18	66.66%	PhD	NR	Mixed	PHQ-9	0-3	*10-14 mod.; 15-19 mod-sev.; ≥ 20 sev.*	3.75
									GAD-2	0-3	*≥ 3*	
									STAI-S-20	0-3	NA	
160	Muro et al., 2022	ESP	2022	140	NR	PhD	NR	NR	PHQ-9	0-3	*10-14 mod.; 15-19 mod-sev.; ≥ 20 sev.*	3.75
									GAD-2	0-3	*≥ 3*	
									STAI-S-20	0-3	NA	
161	Nagy et al., 2019	USA	NR	69	60.90%	PhD	26.5 (2.23)	MED	PHQ-9	0-3	*≥ 10*	5
									SCID-5-RV	NR	NR	
									SBI-9<br>SBI-EE<br>SBI-DP<br>SBI-PA	9-53<br>9-24<br>3-18<br>2-12	NA	
162	Naumann et al., 2022	DEU	2021	335	64.4% (1.8%)	PhD	29.8 (3.4)	NR	BSI-53	NR	*≥ 60*	4.5
									CBI-13	0-100	NA	
									DJGL-11	Max. 11	NA	
163	Nelson, 2014	USA	2013	156	69.9%	PhD	NR	NR	PSS-14	0-4	NA	3.5

164	Nickel, 2024	DEU	2019	27	74.1%	PhD	28.89 (3.46)	Mixed	GHQ-12	0-0-1-1 0-3	GHQ2+, *GHQ4+*	4
									PWI-A-8	0-10	NA	
									WHO-5	0-5	NA	
165	Nottingham, 2017	USA	NR	116	65.5%	PhD	NR	Mixed	SASS-ED<br>SASS-GAD<br>SASS-AA<br>SASS-D<br>SASS-MH	0-15	Above average 80th percentile;<br>At risk 95th percentile	4.25
									SASS-psychological Distress	0-69		
166	Okun et al., 2022	USA	2020	6	100%	PhD	36 (4)	NR	DASS-21(7)	0-3	NA	4.5
									PSS-14	0-4	NA	
									QIDS-SR-16	0-27	6-10 mild; 11-15 mod.; 16-20 sev.; ≥ 21 very sev.	
									PSQI	0-3	> 5	
167	Oliveira de Freitas et al., 2023	BRA	2020	45	64.44%	PhD	30.97 (7.06)	Dental	DASS-21*2	0-3	NA	5
168	Panayidou & Priest, 2021	GBR	NR	56	70%	PhD	NR	NR	WEMWBS	1-5	*≤ 40*	3.75
169	Panger et al., 2017	USA	2016	3190	NR	PhD	NR	NR	CES-D-R-20 to CES-D-20	(0-4)	≤ *16*	7
170	Park et al., 2021	CDN	2019	48	NR	PhD	NR	NR	DASS-21*2	0-3	NA	3
									MBI-SS-15<br>MBI-SS-EE-5<br>MBI-SS-DP-5<br>MBI-SS-EF-5	0-6	NA	
									MSPSS-12	1-7	NA	
171	Pausik et al., 2022	FRA	2020 May	568	60.38%	PhD	28.22 (5.82)	Mixed	DASS-21*2	0-3	NA	3.75
									WEMWBS	1-5	≤ 41	
172	Pausik et al., 2022	FRA	2020 November	232	65.51%	PhD	28.03 (5.51)	Mixed	DASS-21*2	0-3	NA	3.75
									WEMWBS	1-5	≤ 41	
173	Pausik et al., 2022	FRA	2021 May	167	63.47%	PhD	27.81 (5.03)	Mixed	DASS-21*2	0-3	NA	3.75
									WEMWBS	1-5	≤ 41	
174	Pervez et al., 2020	INT	2019-2020	13	49.6%	PhD	NR	BMA	PHQ-9	0-3	0-4 mini.; 5-9 mild; *10-14 mod.; 15-19 mod-sev.; ≥ 20 sev.*	4
									GAD-7	0-3	0-4 mini.; 5-9 mild; *10-14 mod.; ≥ 15 sev.*	
									MSPSS-12	1-5	NA	
175	Peters, 2007	USA	NR	104	76%	PhD	NR	Mixed	GHQ-21[31]	0-3	NA	4.5
									PSS-14	0-4	NA	
									QOLI	0-198	NA	
176	Petruziello et al.,	ITA	2020	243	53.7%	PhD	29.01	NR	WHO-5 Index	1-5	NA	3.75

[31] Only three out of four subscales were used.

	2024a						(4.19)		DASS-21	0-5	NA	
									Exhaustion-9	1-5	NA	
									Job Satisfaction-1	1-5	NA	
									Supervisor Support-22	1-5	NA	
177	Petruziello et al., 2024a	ITA	2021	264	54.2%	PhD	28.95 (4.01)	NR	WHO-5 Index	1-5	NA	3.75
									DASS-21	0-5	NA	
									Exhaustion-9	1-5	NA	
									Job Satisfaction-1	1-5	NA	
									Supervisor Support-22	1-5	NA	
178	Petruziello et al., 2024b	ITA	2021	216	52.8%	PhD	29.25 (4.95)	NR	MBI-9	1-5	NA	3.75
									Supervisor Support-5	1-5	NA	
179	Pitt et al., 2021	USA	2017	251	65%	PhD	NR	Mixed	GAD-7	0-7	NR	4.5
180	Pyhältö et al., 2020	FIN	2015	380	61.4%	PhD	36.54	Mixed	PIDRS Burnout-8<br>EE-4<br>DP-4	1-7	NA	5.5
181	Pyhältö et al., 2020	GBR	2015	219	61.4%	PhD	36.54	Mixed	PIDRS Burnout-8<br>EE-4<br>DP-4	1-7	NA	5.5
182	Pyhältö et al., 2020	ESP	2015	1815	61.4%	PhD	36.54	Mixed	PIDRS Burnout-8<br>EE-4<br>DP-4	1-7	NA	5.5
183	Pyhältö et al., 2023	FIN	2021	768	67%	PhD	32	Mixed	C-DES<br>Stress-1<br>EE-5<br>DP-6	1-7	NA	5.25
184	Ramadoss et al., 2022	USA	2020	135	62% (2%)	PhD	NR	LS	PHQ-2	0-3	*≥ 3*	4.5
									GAD-2	0-3	*≥ 3*	
185	Ramadoss & Horn, 2022	USA	2021	99	56% (4%)	PhD	NR	LS	PHQ-2	0-3	*≥ 3*	4.5
									GAD-2	0-3	*≥ 3*	
186	Rukmanee et al., 2024	THA	2019 2020	30	NR	PhD	NR	MED	DASS-21*2	0-3	NA	2.75
187	Russell et al., 2025	DEU	2024	555	50.09% (1.08%)	PD	32.73	Mixed	PHQ-8	0-3	0-4 mini.; 5-9 mild; *10-14 mod.; 15-19 mod-sev.; ≥ 20 sev.*	5
									GAD-7	0-3	0-4 min.; 5-9 mild; *10-14 mod., ≥ 15 sev.*	
									SWLS	1-7	NA	
188	Schad et al., 2022	USA	2019	159	NR	PhD	NR	LS & MED	PHQ-9	(0-3)	1-4 mini.; 5-9 mild; *10-14 mod.; 15-19 mod-sev.; ≥ 20 sev.*	5

									GAD-7	(0-3)	0-4 min.; 5-9 mild; *10-14 mod., ≥ 15 sev.*	
									Suicidal Ideation-1	0-1	*≥ 1*	
									AUDIT-10	(0-4)	0-7 Zone I; *8-15 Zone II; 16-19 Zone III; ≥ 20 Zone IV*	
									DAST-10	(0-1)	0 none; 1-2 low; 3-5 mod.; *6-8 sub.; ≥ 9 sev.*	
189	Schad et al., 2022	USA	2020	111	NR	PhD	NR	LS & MED	PHQ-9	(0-3)	1-4 mini.; 5-9 mild; *10-14 mod.; 15-19 mod-sev.; ≥ 20 sev.*	5
									GAD-7	(0-3)	0-4 mini.; 5-9 mild; *10-14 mod.; ≥ 15 sev.*	
									Suicidal Ideation-1	0-1	*≥ 1*	
									AUDIT-10	(0-4)	0-7 Zone I; *8-15 Zone II; 16-19 Zone III; ≥ 20 Zone IV*	
									DAST-10	(0-1)	0 none; 1-2 low; 3-5 mod.; *6-8 sub.; ≥ 9 sev.*	
190	Scheidler, 2008	USA	NR	507	71.6%	PhD	42.34 (9.56)	Mixed	PSS-10	0-4	NA	4.75
									MSPSS-12	7-84	NA	
191	Sellali & El Houda Lahiouel, 2022	DZA	NR	104	NR	PhD	NR	Mixed	MBI-SS<br>MBI-SS-EE-5<br>MBI-SS-DP-4<br>MBI-SS-PA-6	1-5	NA	3.25
192	Seto et al., 2023	JPN	2021	369	NR	PhD	NR	Mixed	PHQ-9	NR	0-4 mini.; 5-9 mild; *10-14 mod.; 15-19 mod-sev.; ≥ 20 sev.*	4.75
									GAD-7	NR	0-4 mini.; 5-9 mild; *10-14 mod.; ≥ 15 sev.*	
193	Shillington et al., 2023	CAN	2023	30	70%	PhD	34.63 (10.19)	Mixed	MHI-38	1-6<br>0-100	NA	2.75
									RAND-36	(0-100)	NA	
194	Smith et al., 2024	USA	2020	442	67% (4%)	PhD	NR	Mixed	PHQ-8	0-3	NR	4.75
195	Solms et al., 2024	NLD	2020	383	62.8%	PhD	31.22 (8.17)	Mixed	Exhaustion-4<br>Cynicism-3	1-7	NA	5.5
									Supervisor Support-5	1-7	NA	
196	Solms et al., 2024	NLD	2022	282	52.2%	PhD	28.22 (5.25)	Mixed	Exhaustion-4<br>Cynicism-3	1-7	NA	5.5
									Supervisor Support-5	1-7	NA	
197	Stubb et al., 2011	FIN	2006	669	70.95%	PhD	39	Mixed	MED NORD<br>Exhaustion-4<br>Stress-1	1-5	NA	5.75
198	Sundström, 2022	FIN	2021	768	65.4% (2.3%)	PhD	36.39	Mixed	C-DES<br>Exhaustion-5<br>Cynicism-6	1-7	NA	5
199	Sverdlik & Hall, 2019	INT	NR	3004	79.6% (1.3%)	PhD	30.39 (6.22)	Mixed	CESD-10	1-4	NR	4.75
									Strain Scale-10	1-5	NA	
200	Sverdlik et al., 2020	CAN	NR	831	76.7% (1.7%)	PhD	30.55 (6.35)	Mixed	CESD-10	1-4	NR	6.75
									Strain Scale-10	1-5	NA	

201	Syropoulos et al., 2021	USA	2020	916	77% (1%)	PhD	27.73 (4.16)	PSY	CESD-10	(0-3)	NR	4.5
									PSS-10	(0-4)	NA	
									SWLS-5	(1-7)	NA	
202	Tadee et al. 2025	USA	2023	299	NR	PhD	NR	Mixed	PSS-4	0-4	NA	4.75
203	Tadee et al. 2025	USA	2023	95	NR	PD	NR	Mixed	PSS-4	0-4	NA	4.75
204	Talarowska et al., 2023	POL	2019	352	55.5%	PhD	28.9 (4.8)	ENG, MED	PSS-10	(0-4)	NA	6
									GHQ-28	(0-0-1-1)	≥ 5	
205	Talarowska et al., 2023	POL	2020	369	69.7%	PhD	22.6 (3.6)	MED, ENG	PSS-10	(0-4)	NA	6
									GHQ-28	(0-0-1-1)	≥ 5	
206	Talkhi et al., 2020	IRN	2018	108	NR	PhD	NR	NR	GHQ-12	0-3	NA	5.75
207	Tan et al., 2023	CHN	2020	285	NR	PhD	NR	NR	SDS-20	1-4	NA	5
									SAS-20	1-4	NA	
									SSRS-10	12-66	NA	
									QPSP-36	1-5	NA	
208	Thiagarajah et al., 2023	MYS	2018	36	NR	PD<br>Tutors and assistant lectures	32.22 (6.39)	NR	PSQI-7	0-3	> 5	7
209	Thiagarajah et al., 2023	MYS	2018	203	NR	PD<br>Lecturers and specialists	37.21 (8.21)	NR	PSQI-7	0-3	> 5	7
210	Thompson, 2024	USA	2023	65	89.2%	PhD	NR	PSY	MBI-HSS-22<br>MBI-HSS-EE-9<br>MBI-HSS-DP-5<br>MBI-HSS-PA-8	0-6	NA	4.5
211	Tommasi et al., 2022	ITA	NA	204	37.3%	PhD	29 (4)	NR	BSI-D	1-5	(≥ *60*)	3.75
									BSI-A	1-7	(≥ *60*)	
212	Trang et al., 2024	USA	2022	52	NR	PD	NR	LS	PHQ-9	(0-3)	*≥ 10*	3.5
									GAD-7	(0-3)	*≥ 10*	
									DASS-42	0-3	NR	
213	Tsybuliak et al., 2024	UKR	2023-2024	83	NR	PhD	NR	NR	GAD-7	0-3	0-4 mini.; 5-9 mild; *10-14 mod.; ≥ 15 sev.*	3.75
214	Tu et al., 2023	USA	2020	290	53.8%	PhD	NR	LS	PHQ-9	0-3	0-4 mini.; 5-9 mild; *10-14 mod.; 15-19 mod-sev.; ≥ 20 sev.*	5
									GAD-7	0-3	0-4 mini.; 5-9 mild; *10-14 mod.; ≥ 15 sev.*	
215	U-DOC, 2021	GBR	2018-2019	3352	65.8% (0.8%)	PhD	30.74 (8.82)	Mixed	PHQ-9	0-3	0-4 mini.; 5-9 mild; *10-14 mod.; 15-19 mod-sev.; ≥ 20 sev.*	5
									GAD-7	0-3	0-4 mini.; 5-9 mild*; 10-14 mod.; ≥ 15 sev.*	
									SBQ-R	(0-6)	(*≥ 7*)	
									ASMRS	NR	NR	
									UCLA-20	(0-3)	NA	

216	Van Beuningen, 2024	NLD	2022	25	60% (4%)	PhD	NR	Mixed	GHQ-12	(0-0-1-1)	(GHQ2+, GHQ3+, *GHQ4+*)	3.5
217	van der Weijden & Teelken, 2023	NLD	2019	676	48% (1%)	PD	35 (4.75)	Mixed	GHQ-12	0-0-1-1	GHQ2+, GHQ3+, *GHQ4+*	5
									Work-family-conflict-1	1-5	NA	
									Guidance by Supervisor-1	1-7	NA	
218	Varley, 2021	USA	2018	53	54.72%	PhD	29.12 (4.99)	Mixed	PSS-10	(0-4)	NA	3.25
									SWLS-5	(1-7)	NA	
									Perceived social support-4	(1-4)	NA	
219	Vilser et al., 2024a	DEU	2022	1275	63.8% (0.9%)	PhD	30.44 (5.98)	Mixed	PHQ-2	1-4	*≥ 3*	6
									GAD-2	1-4	*≥ 3*	
220	Vilser et al., 2024a	DEU	2022	706	67% (0.4%)	PhD	30.1 (6.11)	Mixed	PHQ-2	1-4	*≥ 3*	6
									GAD-2	1-4	*≥ 3*	
221	Vilser et al., 2024b	DEU	2022	1981	65.4% (0.65%)	PhD	30.27 (6.04)	Mixed	PSS-4	1-5	NA	5
222	Vincent et al., 2023	CDN	2022	100	75% (1%)	PhD	32.94 (5.82)	Mixed	DASS-21*2	0-3	NA	4
									DPHS-8	1-5	NA	
223	Wang et al., 2022	CHN	2022	37	86.48%	PhD	24.75 (2.05)	NR	DASS-21	1-4	NA	3
224	Wang et al., 2020	USA	NR	326	NR	PhD	NR	NR	PHQ-9	NR	0-4 mini.; 5-9 mild; *10-14 mod.; 15-19 mod-sev.; ≥ 20 sev.*	5.25
									GAD-7	NR	0-4 mini.; 5-9 mild; *10-14 mod.; ≥ 15 sev.*	
225	Williams et al., 2014	USA	NR	140	72.1% (1.4%)	PhD	29 (6)	Mixed	DASS-42	0-3	NA	3.5
226	Wright, 2006	GBR	2006	15	53.33%	PhD	NR	NR	CORE-OM-34	(0-4)	NA	3.5
227	Yssldyk et al., 2019	INT	NR	146	100%	PD	33.40 (4.45)	Mixed	DASS-21*2	0-3	NA	4.25
									SWLS-5	1-7	NA	
228	Zhang et al., 2024	USA	2023	452	52%	PhD	28.5	LS	PHQ-9	0-3	*≥ 10*	6.25
									GAD-7	0-3	*≥ 10*	
									PHQ-9, item 9	0-3	≥ 1	
									UCLA-8	8-32	NA	
									Advisor Satisfaction-1	1-5	NA	

Note. More information on the study characteristics can be found in the main text (see Study identification and selection, as well as Characteristics of the studies). The provision of data at the level of the individual sample, rather than at the level of the respective studies, is undertaken in order to provide a more detailed level of information. Data on the authors, the publishing year and year of data collection are provided, as well as the sample size, the gender distribution and academic discipline. Data on whether the sample consisted of pre-doctoral (PhD) or post-doctoral (PD) early-career researchers is given as well. All scales utilized to assess the outcome variables, mental health syndromes and symptoms alike, are presented with their respective scoring method and used cut-off. Additionally, information on moderating variables, especially such as social support, advisor relationship, loneliness, life satisfaction and work

satisfaction is provided as well. The cursive indicates the cut-offs used per scale to determine clinical relevance as provided in the Supplementary Tables 10–18. The underlined indicates those studies for which raw data was provided, and summary scores (*M*, *SD*) as well as prevalence rates were calculated based on available raw data. Red indicates such papers with incorrect cut-off scores were used in the paper. Values in brackets indicate that the score method was not provided directly in the publication itself and the common score method per scale as provided in the Supplementary Tables 10–18 was used. If score method was not given, the range was provided. ISWS = COVID-19 International Student Well-Being Study; LS = Life science; ENG = Engineering, CIS = Computer and information science; MAT = Mathematics; PS = Physical science; PSY = Psychology; SS = Social science; SW = Social work; HU = Humanities; EDU = Education; BMA = Business, management and administration; COM = Communication sciences; MED = Medicine including dental medicine; LAW = law; IND = India; FRA = France; USA = United States of America; BRA = Brazil; HUN = Hungary; FIN = Finland; GBR = Great Britain; INT = International sample; AUS = Australia; ITA = Italia; BEL = Belgium; ESP = Spain; PHL = Philippines; HKG = Hongkong; IRL = Ireland; GRC = Greece; CAN = Canada; DEU = Germany; ARG = Argentina; CHN = China; BLG = Bulgaria; CHE = Switzerland; CYP = Cyprus; CZE = Czechia; DNK = Denmark; ISL = Iceland; NLD = Netherlands; NOR = Norway; PRT = Portugal; ROU = Romania; RUS = Russia; SVK = Slovakia; SWE = Sweden; TUR = Turkey; ZAF = South Africa; IRN = Iran; POL = Poland; THA = Thailand; DZA = Algeria; JPN = Japan; MYS = Malaysia; UKR = Ukraine; GHQ-12 = General health questionnaire, 12 items; MBI = Maslach burnout inventory; PHQ-9 = Patient health questionnaire, 9 items; PHQ-8 = Patient health questionnaire, 8 items; PHQ-2 = Patient health questionnaire, 2 items; GAD-7 = General anxiety disorder, 7 items; GAD-2 = General anxiety disorder, 2 items; AWAI-S = Advisory working alliance inventory – student version; AWAI-30 = Advisory working alliance inventory, 30 items; BDI-II-21 = Beck depression inventory, second version, 21 items; BAI-21 = Beck anxiety inventory, 21 items; PSS-10 = Perceived stress scale, 10 items; PSS-14 = Perceived stress scale, 14 items; PSS-4 = Perceived stress scale, 4 items; CBI-13 = Copenhagen burnout inventory, 13 items; CBI-S-25 = Copenhagen burnout inventory – student version, 25 items; F-SOZU = Questions to social support (*Fragen zur sozialen Unterstützung*); K-6 = Kessler psychological distress scale, 6 items; K-10 = Kessler psychological distress scale, 10 items; MBI-ES = Maslach burnout inventory – educators survey; EE = emotional exhaustion subscale; DP = depersonalization subscale; PA = personal accomplishment subscale; MHC-SF-14 = Mental health continuum – short form, 14 items; UCLA-3 = UCLA loneliness scale, 3 items; UCLA-20 = UCLA loneliness scale, 20 items; UCLA-8 = UCLA loneliness scale, 8 items; DASS-42 = Depression, anxiety, stress scale, 42 item version; DASS-21*2 = Depression, anxiety, stress scale, 21 item version multiplied by two for comparison with the DASS-42; ESP-5 = Eating disorder screening tool for primary care, 5 items; BDI-FS = Beck depression inventory – fast screen; BMS-10 = Burnout measure scale, 10 items; WEMWBS = Warwick Edinburgh mental wellbeing scale; SWEMWBS = Short Warwick Edinburgh mental wellbeing scale; JSQ-4 = Jenkins sleep questionnaire, 4 items; BRUMS-D-4 = Brunnel mood scale, depression subscale, 4 items; BRUMS-A-4 = Brunnel mood scale, anxiety subscale, 4 items; ASRS-V1.1-6 = Adult ADHD self-report scale, version 1.1., 6 items; SPOS-4 = Survey of perceived organizational support, 4 items; AUDIT-10 = Alcohol use disorders identification test, 10 items; AUDIT-C = Alcohol use disorders identification test – consumption; CESD-R-20 = Center for epidemiological disease – revised, 20 items; CESD-20 = Center for epidemiological disease, 20 items; CESD-8 = Center for epidemiological disease, 8 items; BI-EE-4 = Burnout inventory, emotional exhaustion, 4 items; HADS = Hospital anxiety and depression scale, 14 items; GDES-A = general dental environment stress scale – adult; SWLS-5 = Satisfaction with life scale, 5 items; STAI-40 = State-trait-anxiety inventory, 40 items; SCOFF-5 = Sick-control-one stone-fat-food, 5 items; MSPSS-12 = Multidimensional Scale of Perceived Social Support, 12 items; GSI-R-20 = Graduate stress inventory – revised, 20 items; SBI-9 = School burnout inventory, 9 items; CIDI = Composite international diagnostic interview**;** DAST-10 = Drug abuse screening test, 10 items; GSI-AS-13 = Graduate stress inventory – revised; CCAPS-62 = Counseling center assessment of psychological symptoms, 62 items; WHOQOL-BREF = WHO quality of life; GLS-5 = General life

satisfaction, fixed form B, 5 items; SBQ-R = Suicide behaviors questionnaire – revised; SCID-5-RV = Structured clinical interview for *DSM*-5, research version; BSI-53 = Brief symptom inventory, 53 items; PWI-A-8= Personal wellbeing index – adult, 8 items; SASS = Symptoms and assets screening scale; QIDS-SR-16 = Quick inventory of depressive symptomatology – self-report; SDS-20 = Zung self-rating depression scale, 20 items; SAS-20 = Zung self-rating anxiety scale, 20 items; PSQI = Pittsburgh sleep quality index; MBI-SS = Maslach burnout inventory – student scale; MBI-HSS = Maslach burnout inventory – human services survey; MHI = Mental health inventory; RAND-36 = RAND health survey, 36 items; SSRS-10 = Social support rating scale, 10 items; QPSP-36 = Questionnaire on psychological stressors of postgraduates, 36 items; BSI-A = Brief symptom inventory, anxiety subscale; BSI-D = Brief symptom inventory, depression subscale; ASMRS = Altman self-rating mania scale; DPHS-8 = Doctoral psychological health scale, 8 items; CORE-OM-34 = Clinical outcomes in routine evaluation, 34 items; DJGL-11 = De Jong Gierveld loneliness scale, 11 items; SPOS-8 = Survey of perceived organizational support 8 items; MSSS-33 = Minority student stress scale, 33 items; PIDRS = Personal interest in doctoral research – scale; mod. = moderate; sev. = severe; mod.-sev. = moderately-severe; mini. = minimal; NR = Not reported; NA = not applicable; Year, Year of the data collection not publication; QA = overall study quality based on the JBI; PD = post-doctoral researcher; PhD = pre-doctoral researcher.

Supplementary Figure 1. Histogram of the number of studies conducted per year

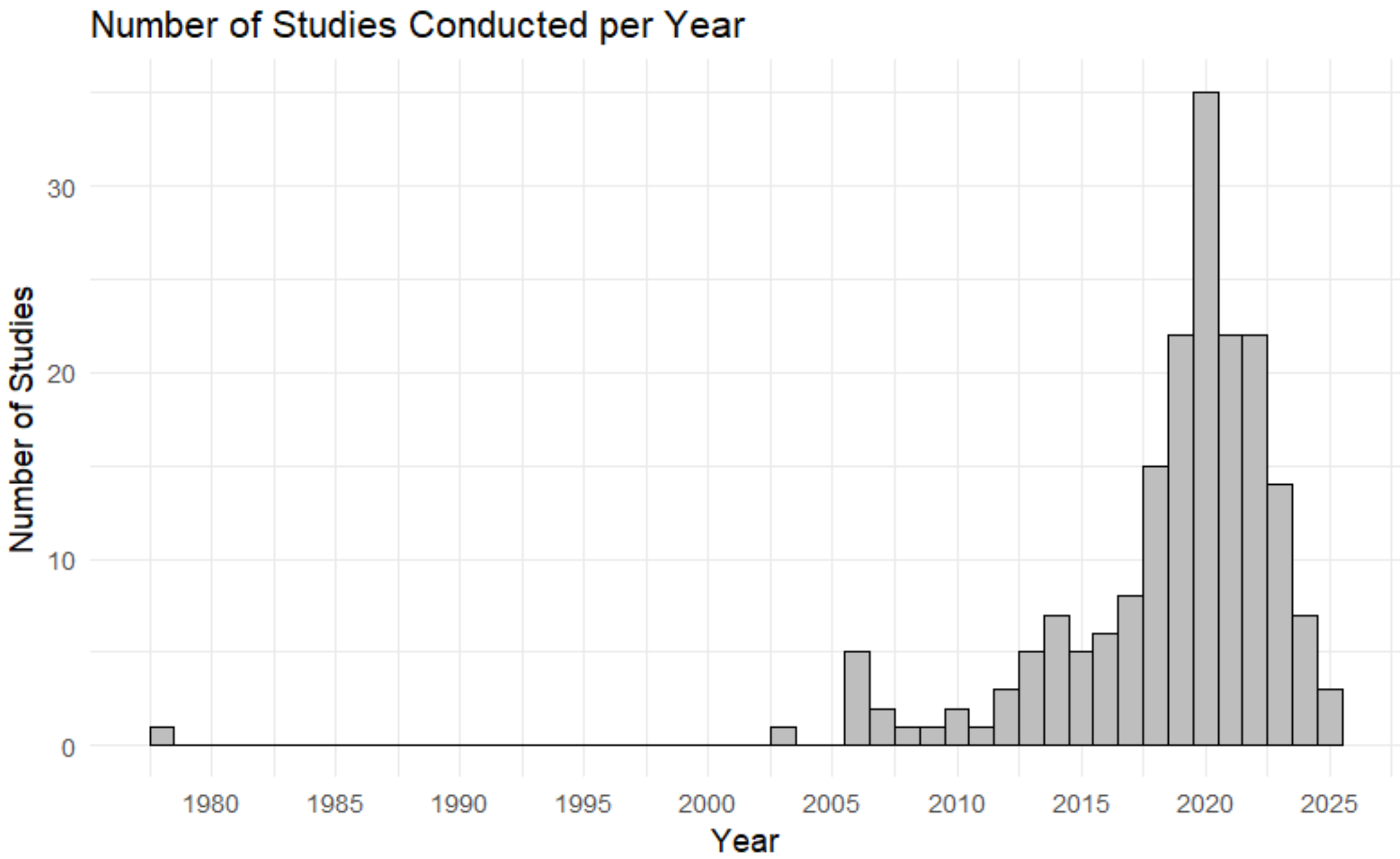

Note. The histogram displays the distribution of studies conducted per year. The first study was conducted in 1978. Similar to the figure below, this figure also highlights the heightened research activity in recent years—particularly during and after the COVID-19 pandemic, with many studies having been conducted during and after 2020.

Supplementary Figure 2. Histogram of the number of studies published per year

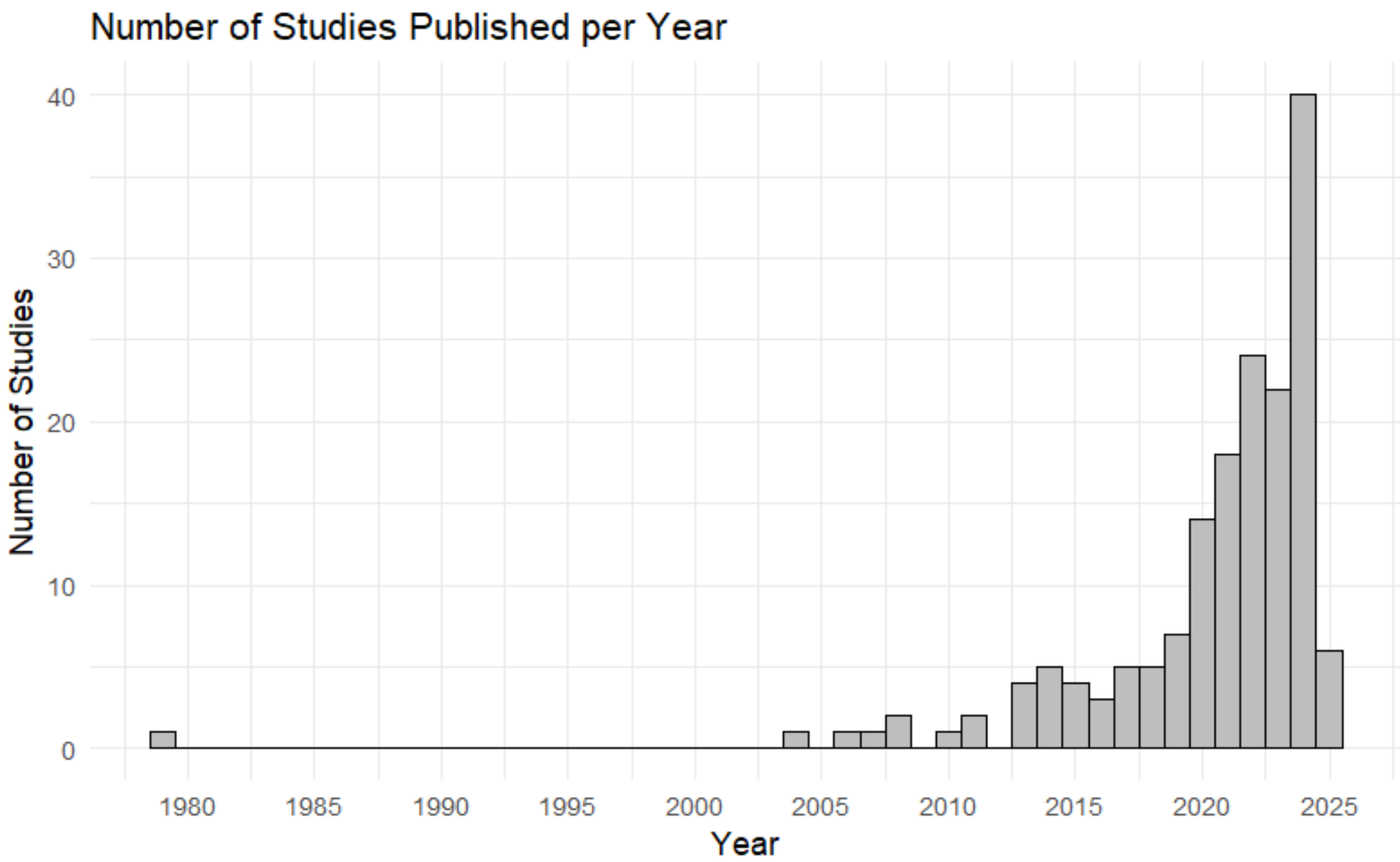

Note. The histogram displays the distribution of included studies by publication year. The first study was published in 1979. A noticeable increase in publications is observed after 2010, with a sharp increase around 2021, indicating heightened research activity in recent years—particularly during and after the COVID-19 pandemic.

Supplementary Table 2. Academic discipline classification system

**Humanities**				
Humanities				
**Social Science**				
Social Science	Psychology			
**Natural Science**				
Life Science	Physical Science			
**Formal Science**				
Mathematics	Computer and Information Systems			
**Applied Science**				
Education<br>Law	Business and Management Administration<br>Engineering	Communication Science<br>Medicine		

Note. This table provides information on the classification system of academic disciplines. This broader classification was conducted in order to retain greater power in the moderator analysis. Classification was conducted based on Wikipedia (2025).

Wikipedia. (2025). Outline of academic disciplines. In *Wikipedia*. https://en.wikipedia.org/w/index.php?title=Outline_of_academic_disciplines&oldid=1276105940

Supplementary Material 1. Approach of the exploratory country classification

As of now, there is no universally standardized classification system for pre-doctoral (e.g., PhD candidates) and post-doctoral positions that groups countries based on criteria such as funding schemes, duration, contract type, working hours, or other relevant factors. However, such a system could be highly valuable for comparing academic and research environments across different countries. To the end of this meta-analysis, an exploratory classification system was generated by utilizing artificial intelligence (AI), namely DeepSeek R1 and ChatGPT-4o. Both AI tools were fed the same prompt, asking them to provide a country classification for pre-doctoral and post-doctoral positions of all countries included in the analysis, based on similar pre- and post-doctoral working conditions, working contract, weekly working hours, and duration. The AI tools were additionally asked to generate other aspects differentiating the varying pre- and post-doctoral position per country from another.

The groupings generated by DeepSeek (Supplementary Table 3) and ChatGPT (Supplementary Table 4) were merged into a common classification system (Supplementary Table 5) via discussion and consensus by the present authors. This classification system was further validated with ChatGPT-5.1, using the same prompt as in the foregoing classifications, but this time also explicitly incorporating the OECD report *Benchmarking Higher Education System Performance* (2019) as a primary source. This resulted in a country grouping (Supplementary Table 6) that was largely consistent with the clustering synthesized from DeepSeek R1 and Chat-GPT-4o (agreement rate 76%; Cohen $\kappa = .71$, $p < .001$). Major differences were that ChatGPT-5.1 identified a new cluster (southern European catch-up systems) with Greece, Italy, Spain, Portugal, and Ukraine (all formerly part of Cluster 3: self-funded or low-funded systems) and put all former Cluster-5 countries (unique or hybrid systems) into other pre-existing clusters (Cyprus and Iran into Cluster 3, Iceland into Cluster 2, and Algeria into Cluster 4). We interpreted this result as mostly confirming the original classification.

We further also instructed Gemini (Version: Gemini 2 Flash) to create a third AI-based country clustering. This country clustering was again (only) based on the predefined criteria used for DeepSeek and ChatGPT. This third clustering (Supplementary Table 7) identified only 5 clusters, putting former Cluster-5 countries into Clusters 3 and 4, but was otherwise even slightly more consistent with the original classification than the ChatGPT-5.1 result (81% agreement rate of the Gemini clustering with the original clustering; Cohen $\kappa = .77$, $p < .001$).

Both validation attempts, using other (versions of) LLMs than the original approach (and, in the first validation attempt also a slightly altered prompt, augmenting the LLM with a source [the OECD report] that can be considered highly relevant to such a categorization), broadly confirmed the original classification. We concede that further research is still needed to find optimal ways of classifying countries is still needed. However, given the broad replicability of the proposed classification system, it was used it present study as-is.

Supplementary Table 3. Country clustering based on DeepSeek R1

<table>
<tr><td colspan="5">High-Funding, Structured Systems
Fully funded positions, employment contracts, structured working hours, strong benefits (health insurance, pensions), and robust support for training and development.</td></tr>
<tr><td>Germany
Sweden</td><td>Finland
Netherlands</td><td>France
Belgium</td><td>Switzerland
Denmark</td><td>Austria
Norway</td></tr>
<tr><td colspan="5">Competitive, Flexible Systems
Mix of fully and partially funded positions, flexible working hours, high-intensity research environments, and access to prestigious fellowships.</td></tr>
<tr><td>USA
Japan</td><td>Australia
Hong Kong</td><td>Canada
Singapore</td><td>Ireland</td><td>UK</td></tr>
<tr><td colspan="5">Emerging Research Economies
Increasing availability of funded positions, growing international collaboration, and improving research infrastructure.</td></tr>
<tr><td>China
Brazil</td><td>India</td><td>South Africa</td><td>Malaysia</td><td>Thailand</td></tr>
<tr><td colspan="5">Self-Funded or Low-Funding Systems
Prevalence of self-funded or partially funded positions, limited benefits, and less structured working environments.</td></tr>
<tr><td>Spain
Italy
Ukraine</td><td>Portugal
Greece
Turkey</td><td>Bulgaria
Czechia
Argentina</td><td>Hungary
Poland
Philippines</td><td>Romania
Slovakia</td></tr>
<tr><td colspan="5">Industry-Driven Systems
Strong emphasis on industry-academia collaboration, competitive funding, and high expectations for productivity.</td></tr>
<tr><td>South Korea</td><td></td><td></td><td></td><td></td></tr>
<tr><td colspan="5">Unique or Hybrid Systems
Countries with unique research ecosystems that don’t fit neatly into the other categories.</td></tr>
<tr><td>Russia</td><td>Cyprus</td><td>Iceland</td><td>Algeria</td><td>Iran</td></tr>
</table>

Supplementary Table 4. Country clustering based on ChatGPT-4o

<table>
<tr><td colspan="5">High Government Support, Structured Contracts, and Career Security
These countries typically have a highly regulated research system, where PhD students and post-docs are often funded by government scholarships or institutional support, with clearly defined contracts and durations.
Germany: Pre-doctoral: 3–4 years, mostly government-funded or university-supported with contracts offering benefits. Post-doctoral: 2–3 years, often government or research-grant funded, with stable contracts and good benefits.
Sweden: Pre-doctoral: 4 years, PhD students are considered employees of universities, with full benefits. Post-doctoral: Typically 2 years, well-funded by research grants, often part of large collaborative projects.
France: Pre-doctoral: 3 years, typically funded by public institutions or research contracts. Post-doctoral: 1–2 years, primarily funded by grants, but contracts usually include some benefits.</td></tr>
<tr><td>Germany
Sweden</td><td>Finland
Netherlands</td><td>France
Belgium</td><td>Switzerland
Norway</td><td>Denmark</td></tr>
<tr><td colspan="5">Flexible and Grant-Dependent Research Careers
Countries where PhD students and post-docs often rely on external research grants or fellowships for funding, with flexible contract lengths and more variation in employment conditions.
United States: Pre-doctoral: 4–6 years, with funding via research assistantships, teaching assistantships, or fellowships. Often flexible in terms of funding sources and support. Post-doctoral: 1–3 years, heavily dependent on external grants (e.g., NSF, NIH), typically with variable benefits and no job security.
UK: Pre-doctoral: 3–4 years, funded through research councils or universities, with some flexibility in terms of funding (e.g., scholarships, assistantships). Post-doctoral: 1–3 years, funded by research grants or fellowships, with varying benefits and job security.</td></tr>
</table>

<table>
<tr><td colspan="5">Australia: Pre-doctoral: 3–4 years, funded through scholarships or research assistant positions, often reliant on external grants. Post-doctoral: 1–3 years, grant-funded or fellowships, with varying contract types and benefits.</td></tr>
<tr><td>USA</td><td>Australia</td><td>Canada</td><td>Ireland</td><td>UK</td></tr>
<tr><td colspan="5">Competitive Fellowships with Limited Benefits and Short-Term Contracts
Countries where post-doctoral researchers are typically on competitive fellowships with shorter durations and limited institutional support.
Japan: Pre-doctoral: 4–5 years, funded by universities or public grants, with relatively independent funding for research. Post-doctoral: 1–2 years, highly competitive fellowships (e.g., JSPS fellowships), often limited benefits.
South Korea: Pre-doctoral: 4–5 years, funded primarily through government scholarships or university positions. Post-doctoral: 1–2 years, fellowship-driven positions, with limited social benefits.
Singapore: Pre-doctoral: 4 years, typically funded by government agencies or research institutes. Post-doctoral: 1–2 years, competitive fellowships with fewer benefits compared to European systems.</td></tr>
<tr><td>Japan</td><td>Hong Kong</td><td>South Korea</td><td>Singapore</td><td></td></tr>
<tr><td colspan="5">Emerging Research Systems with Varied Funding Sources and Career Paths
Countries with growing research sectors where funding mechanisms and employment conditions for PhDs and post-docs are still developing, often with a mix of government and private support.
China: Pre-doctoral: 3–5 years, heavily funded by government scholarships, but with some reliance on university grants or external funding. Post-doctoral: 1–2 years, increasingly dependent on research grants, with a competitive fellowship structure.
India: Pre-doctoral: 4–5 years, funded primarily by government scholarships or institutional funds, with some variability in support levels. Post-doctoral: 1–3 years, fellowship-based with limited job security and fewer benefits.
Brazil: Pre-doctoral: 4–5 years, primarily government-funded scholarships, but positions can be insecure. Post-doctoral: 1–2 years, fellowship-driven, with variable support from government and international grants.</td></tr>
<tr><td>China
India</td><td>Brazil
South Africa</td><td>Argentina
Russia</td><td>Ukraine
Malaysia</td><td>Turkey
Philippines</td></tr>
<tr><td colspan="5">Hybrid or Transitional Systems with Mixed Research Structures
These countries have evolving research career structures with mixed elements from both high-support systems and grant-dependent structures. Job security is generally low for post-docs, but these countries are working to develop more structured research careers.</td></tr>
<tr><td>Czechia
Algeria
Thailand</td><td>Poland
Greece
Cyprus</td><td>Italy
Portugal
Slovakia</td><td>Spain
Hungary
Iceland</td><td>Romania
Bulgaria
Thailand</td></tr>
</table>

Note. ChatGPT generated additional information for several countries, also provided in this table.

Supplementary Table 5. Final country classification system

**Cluster 1: Flexible and Grant-Dependent Research Careers**<br>Countries where PhD students and post-doctoral researchers often rely on external research grants or fellowships for funding, with flexible contract lengths and more variation in employment conditions.				
USA	Australia	Canada	Ireland	UK
**Cluster 2: High Government Support, and Structured Contracts**<br>These countries typically have a highly regulated research system, where PhD students and post-doctoral researchers are often funded by government scholarships or institutional support, with clearly defined contracts and durations. Job security can be considered rather high.[32]				
Germany<br>Sweden	Finland<br>Netherlands	France<br>Belgium	Switzerland<br>Norway	Denmark
**Cluster 3: Self-Funded or Low-Funding Systems**<br>Prevalence of self-funded or partially funded positions, limited benefits, and less structured working environments.				
Spain<br>Italy<br>Ukraine	Portugal<br>Greece<br>Bulgaria	Czechia<br>Argentina<br>Philippines	Hungary<br>Poland	Romania<br>Slovakia
**Cluster 4: Emerging Research Systems with Varied Funding Sources and Career Paths**<br>Countries with growing research sectors where funding mechanisms and employment conditions for PhDs and post-doctoral researchers are still developing, often with a mix of government and private support.				
China<br>India	Brazil<br>South Africa	Russia<br>Thailand	Malaysia	Turkey
**Cluster 5: Unique or Hybrid Systems**<br>Countries with unique research ecosystems that did not fit neatly into the other categories.				
Cyprus	Iceland	Algeria	Iran	
**Cluster 6: Competitive Fellowships with Limited Benefits and Short-Term Contracts**<br>Countries where post-doctoral researchers are typically on competitive fellowships with shorter durations and limited institutional support. Pre-doctoral positions often lack formal employment; thus, job security is rather low. Funding schemes can vary, and institutional support is also low for pre-doctoral researchers.				
Japan	Hong Kong	South Korea	Singapore	

[32] It is important to differentiate between career and job security. We define job security as having a clearly defined job that adheres to certain rules and regulations. We want to emphasize here that career security, in the sense of being able to pursue an academic career that goes beyond a pre- or post-doctoral position and usually leads to a professorship, does not necessarily follow from job security.

Supplementary Table 6. Country clustering based on ChatGPT-5.2 and the OECD report

**Regulated & Well-Funded Systems**<br>*High public funding, transparent regulation, strong welfare support; balanced teaching & research; strong QA; inclusive, equitable systems; high completion, low dropout; moderate-to-high R&D; strong employability, low inequality; social-democratic, high-quality and well-regulated.*				
Germany<br>Sweden	Finland<br>Netherlands	France<br>Belgium	Switzerland<br>Denmark	Iceland<br>Norway
**High-Performance, Competitive Systems**<br>*Market-oriented funding, high tuition & autonomy; research-intensive; global competition; private–public partnership; excellent completion & research outputs; high income premium, competitive pressures; global, research-driven, elite and marketized.*				
USA	Australia	Canada	Ireland	UK
**Continental / Reforming Mid-Resource Systems**<br>*Moderate funding; evolving autonomy; reforms under EU or national transitions; teaching-focused; variable research activity; reforming governance; rising completion, uneven quality; improving employment outcomes; structured systems under transformation.*				
Cyprus<br>Czechia<br>Romania	Bulgaria<br>Slovakia	Poland<br>Philippines	Argentina<br>Iran	Russia<br>Hungary
**Sothern European Catch-Up Systems**<br>*Lower expenditure, centralised regulation, underfunded HEIs; high teaching burden, limited flexibility, low mobility; modest completion & R&D output; moderate earnings premium; reforms ongoing; reform-driven but under-resourced.*				
Spain	Portugal	Ukraine	Italy	Greece
**High-Tech Asian Systems**<br>*High R&D expenditure, competitive access, strong government steering; STEM-dominant, high research output, selective systems; high quality, low diversity; excellent innovation outcomes, strong economic link; innovation-led, export-oriented, research-driven.*				
South Korea	Singapore	Japan	Hong Kong	
**Emerging Lower Expenditure Systems**<br>*Low-to-moderate per-student funding; large public–private gap; Rapid expansion, uneven QA, access-driven; high variability; moderate graduation; moderate-to-low labour returns; large inequalities; expansion-driven, limited resources, evolving governance.*				
Brazil	China	Thailand	Malaysia	India
South Africa	Algeria	Turkey		

Supplementary Table 7. Country clustering based on Gemini 2 Flash

**The 'Employee-Researcher' Model (Statutory & Structured)**<br>*This highly structured model defines both PhD candidates and Postdocs primarily as salaried employees with full social security, pension benefits, union representation, and legally regulated working hours. This provides the strongest legal protection for early career researchers (ECRs).*				
Germany<br>Denmark	Netherlands<br>Finland	Sweden<br>Switzerland	Norway<br>Belgium	France
**The 'Trainee-Scholarship' Model (Flexible & Market-Driven)**<br>*This model views the PhD phase as student training (stipend/scholarship) and the Postdoc phase as advanced, grant-funded research training. It is characterized by high PI (Principal Investigator) power, high mobility expectations, and a stronger link between the academic and industrial job markets.*				
USA	Canada	Australia	UK	Ireland
**The 'Rapid Ascent' Model (State-Driven & Output-Focused)**<br>*Systems characterized by massive, targeted governmental investment aimed at global research ascendancy. The ECR track is highly competitive and output-focused (publications, patents) and often features unique, highly-incentivized postdoctoral contracts as a career gate.*				
China	South Korea	Singapore	Hong Kong	Japan
**The 'Mediterranean & Transitioning' Model (Hierarchical & Hybrid)**<br>*Systems with traditional, often hierarchical structures that are currently transitioning toward more flexible models, but often suffer from underfunding, internal bottlenecks, and high precarity. The ECR track mixes old titles with new temporary contract forms. This cluster absorbs many Central/Eastern European nations due to similar challenges in transitioning academic systems.*				
Spain<br>Poland<br>Bulgaria	Italy<br>Czechia<br>Ukraine	Portugal<br>Hungary<br>Cyprus	Greece<br>Romania<br>Iceland	Turkey<br>Slovakia
**The 'Emerging & Resource-Constrained' Model (Variable & Developing)**<br>*Academic systems that are large and globally influential but face significant resource constraints, high internal variation (between elite and mass institutions), and inconsistent, developing ECR frameworks. The ECR status is often loosely defined, non-standardized, and heavily dependent on specific grant funding.*				
India<br>Russia	Brazil<br>Thailand	South Africa<br>Philippines	Iran<br>Malaysia	Argentina<br>Algeria

Supplementary Table 8. Summary of study and sample characteristics included in the TLMA by type of effect size

Characteristic	***POMP* scores**		***Prevalence***	
	PhD	Post-Doc	PhD	Post-Doc
Range years in which studies were conducted	1978 – 2025	2012 – 2025	2007 – 2025	2012 – 2025
Number of records or databases	143	14	78	9
Number of samples	198	16	127	11
Overall sample size	127.644	2631	102.064	2822
Range of sample sizes	3–6424	23–555	3–6424	36–676
Age, years on sample level				
Mean ± SD<br>*Median*	30.17±4.18<br>29.68	35.49±3.59<br>34.65	29.37±4.01<br>29.03	35.96±3.87<br>35.00
Gender Distribution on sample level				
Female (% ± SD)<br>*Diverse (% ± SD)*	63.96±11.25<br>2.04±2.60	62.02±20.35<br>2.50±1.05	61.95±11.28<br>2.15±2.95	56.25±13.01<br>2.02±1.13
Country on sample level				
Cluster 1 (n)	103	7	64	6
Cluster 2 (n)	38	1	26	2
Cluster 3 (n)	25	1	21	0
Cluster 4 (n)	19	3	12	3
Cluster 5 (n)	10	4	4	0
Cluster 6 (n)	3	0	0	0

Note. POMP = percent of maximum possible; *SD* = standard deviation; Cluster 1 = flexible and grant-dependent research careers; Cluster 2 = high government support, structured contracts; Cluster 3 = self-funded or low-funding systems; Cluster 4 = emerging research systems with varied funding sources and career paths; Cluster 5 = unique or hybrid systems; Cluster 6 = competitive fellowships with limited benefits and short-term contracts.

Supplementary Table 9. Number of studies and estimates per syndrome, symptom and mental health

	**POMP scores**				**Prevalence**			
**Outcome**	k_{record}	k_{sample}	**#ES**	***n***	k_{record}	k_{sample}	**#ES**	***n***
**Syndromes**								
ADHD	1		1	513	2	2	2	582
Agoraphobia	-	-	-	-	1	1	1	69
Alcohol Abuse	13	22	22	48.833	13	22	22	48.757
Bipolar Disorder	-	-	-	-	2	2	2	3421
Depression	95	115	151	99.285	72	92	123	93.637
Drug Abuse	2	3	3	1182	3	4	4	1251
Eating Disorder	16	26	26	57.135	17	27	27	57.601
GAD	82	95	109	78.578	58	70	74	74.584
OCD	-		-	-	1	1	1	69
Panic Disorder	-	-	-	-	1	1	1	69
Personality Disorder	-		-	-	1	1	1	69
PTSD	-		-	-	1	1	1	69
Social Phobia	-	-	-	-	1	1	1	69
Specific Phobia	-	-	-	-	1	1	1	69
**Symptoms**								
Psychological Distress	26	28	35	12.773	11	12	13	7446
Burnout	16	17	19	3326	-		-	-
NSSI	-		-	-	13	22	22	49.173
Sleep Disturbance	3	3	4	385	2	2	3	40
Stress	63	60	70	21.986	-		-	-
Somatic Symptoms	1	1	1	280	3	4	4	1584
Suicidal Ideation	28	44	46	64.981	30	46	49	69.701

Note. POMP = percent of maximum possible; k_{record} = number of records; k_{sample} = number of samples; #ES = number of effect sizes; GAD = generalized anxiety disorder; ADHD = attention deficit hyperactivity disorder; PTSD = posttraumatic stress disorder; OCD = obsessive-compulsive disorder; NSSI = non-suicidal self-injury.

Supplementary Table 10. Outcomes dominated by specific databases

	**Instrument used**	**POMP**		**Prevalence**	
**Healthy Minds Network**		**#ES**	***n***	**#ES**	***n***
**Outcome**					
**Syndrome**					
Alcohol abuse	AUDIT-C	6	13,410	6	13,410
Depression	PHQ-9	30	61,204	30	61,204
Eating disorder	SCOFF-5 until 2024<br>SDE After 2024	21<br>4	52,365<br>4,654	21<br>4	52,365<br>4,654
GAD	GAD-7	25	57,019	25	57,019
**Symptom**					
Suicidal ideation	PHQ-9, item 9	30	61,204	30	61,204
NSSI	In the past year, have you ever done any of the following intentionally?	-	-	21	48,261
**Moderators**					
Loneliness	UCLA-3	127	61,204	148	61,204
Mental health diagnosis	Have you ever been diagnosed with any of the following conditions by a health professional (e.g., primary care doctor, psychiatrist, psychologist, etc.)?	127	61,204	148	61,204
**C19 ISWS**					
Depression	CESD-8	25	4,812	25	4,812

Note. Exact questions on how mental health diagnosis or non-suicidal self-injury was assessed can be found in the codebooks of the Healthy Minds Network. #ES = number of effect sizes; *n* = sample size; C19 ISWS = COVID-19 International Student Well-Being Study; AUDIT-C = Alcohol use disorders identification test – consumption; NSSI = non-suicidal self-injury; UCLA-3 = ; CESD-8 = Center for epidemiological disease, 8 items; PHQ-9 = Patient health questionnaire, 9 items; GAD-7 = Generalized anxiety disorder, 7 items; SCOFF-5 = Sick-control-one stone-fat-food; SDE = Screen for eating disorder.

Supplementary Table 11. Variable analysis of missingness

**Characteristic**	**POMP**	**Prevalence**
**Age** (mean)	25.75%	15.91%
**Gender**		
Percent female	14.92%	9.81%
Percent diverse	60.63%	43.75%
**Ethnicity**		
Caucasian	50.93%	30.39%
Asian	55.59%	34.37%
Latin	62.87%	45.45%
Black	57.09%	36.36%
**Academic Discipline**		
Applied Science	50.37%	40.34%
Social Science	44.78%	38.35%
Natural Science	47.20%	38.35%
Formal Science	82.65%	82.95%
**Risk and Protective Factors**		
Job Satisfaction	98.51%	99.72%
Life Satisfaction	94.77%	96.51%
Advisor Relationship	93.09%	94.89%
Social Support	95.89%	98.86%
Work-Life Balance	-	99.72%
Loneliness	88.06%	77.84%
**History of Mental Disorders**	79.10%	61.65%

Note. This table provides information on missing data per variable in percent and per model. The missingness was analyzed in order to determine which variables data imputation should be conducted. As no variable has a missingness ≤ 10% in both models, we did not utilize data imputation methods. Instead, only such variables with non-missingness were included in the moderator analyses. POMP = percent of maximum possible.

Supplementary Table 12. Risk of bias (JBI) assessment coding scheme

**Criteria**	Criteria Description	Points	Description of points
**Sample Frame**	Was the sample frame appropriate to address the target population?<br>This question relies upon knowledge of the broader characteristics of the population of interest and the geographical area. If the study is of women with breast cancer, knowledge of at least the characteristics, demographics and medical history is needed. The term "target population" should not be taken to infer every individual from everywhere or with similar disease or exposure characteristics. Instead, give consideration to specific population characteristics in the study, including age range, gender, morbidities, medications, and other potentially influential factors. For example, a sample frame may not be appropriate to address the target population if a certain group has been used (such as those working for one organization, or one profession) and the results then inferred to the target population (i.e. working adults). A sample frame may be appropriate when it includes almost all the members of the target population (i.e. a census, or a complete list of participants or complete registry data).	0<br>.5<br><br>1	No comparison between the sample frame and target population.<br>Comparison between the sample frame and target population, but it is stated that it is not generalizable or does not correspond sufficiently.<br>Comparison between the sample frame and target population in regard to socio-demographics, the congruency is deemed sufficient.<br>OR<br>Comparison between the sample frame and target population is made and adjusted (e.g., weighting adjustments) if needed.<br>OR<br>No adjustments were needed as the sample corresponds to the target population.<br>OR<br>A full (random, stratified) sampling was conducted and thus the sample frame is representative of the target population.
**Sampling**	Were study participants sampled in an appropriate way?<br>Studies may report random sampling from a population, and the methods section should report how sampling was performed.<br>Random probabilistic sampling from a defined subset of the population (sample frame) should be employed in most cases, however, random probabilistic sampling is not needed when everyone in the sampling frame will be included/ analyzed. For example, reporting on all the data from a good census is appropriate as a good census will identify everybody.<br>When using cluster sampling, such as a random sample of villages within a region, the methods need to be clearly stated as the precision of the final prevalence estimate incorporates the clustering effect.<br>Convenience samples, such as a street survey or interviewing lots of people at public gatherings are not considered to provide a representative sample of the base population.	0<br><br>1	Convenience sampling (e.g., snowball sampling, social media, volunteers, non-probabilistic sampling).<br>Random, stratified or full sampling.
**Sample Size**	Was the sample size adequate?<br>We are looking for evidence that the authors conducted a sample size calculation to determine an adequate sample size. For conditions with a low prevalence, a larger sample size is needed. Also consider sample sizes for subgroup (or characteristics) analyses, and whether these are appropriate.<br>Sometimes, the study will be large enough (as in large national surveys) whereby a sample size calculation is not required. In these cases, sample size can be considered adequate.<br>When there is no sample size calculation and it is not a large national survey, the reviewers may consider conducting their own sample size analysis.	0<br><br>.5<br><br><br>1	A sample size calculation was not conducted, and the sample is too small ($n < 300$).<br>A sample size calculation was conducted, but the sample is too small.<br>A sample size was < 300, but sample size calculations backed this lower value.<br>OR<br>A sample size calculation was conducted and the sample is sufficiently large.
**Study Description**	Were the study subjects and the setting described in detail?	0<br>.25<br>.25	No further socio-demographic information is provided.<br>Information on the country in which the data was collected is provided.<br>Information on the age distribution of the sample is provided.

	Certain diseases or conditions vary in prevalence across different geographic regions and populations (e.g. Women vs. Men, sociodemographic variables between countries). The study sample should be described in sufficient detail so that other researchers can determine if it is comparable to the population of interest to them.<br><br>Points may be given per criteria that is fulfilled, leading to a maximum score of 1. In case no further criteria is provided, this results in a zero-point score.	.25<br>.25	Information on the gender composition is provided.<br>Further characteristics are given (study level, faculty, ethnicity, etc.).
**Data Analysis**	Was the data analysis conducted with sufficient coverage of the identified sample?<br><br>Coverage bias can occur when not all subgroups of the identified sample respond at the same rate. For instance, you may have a very high response rate overall for your study, but the response rate for a certain subgroup (i.e. older adults) may be quite low.	0<br>.5<br>1	No information on the coverage rate is provided.<br>Coverage rate of ECR is below (n < 300).<br>Coverage rate of ECR is above (n ≥ 300).<br>OR<br>Sample consists solely of ECR (PhD, post-doc).
**Validity of Methods**	Were valid methods used for the identification of the condition?<br><br>Here we are looking for measurement or classification bias. Many health problems are not easily diagnosed or defined and some measures may not be capable of including or excluding appropriate levels or stages of the health problem. If the outcomes were assessed based on existing definitions or diagnostic criteria, then the answer to this question is likely to be yes.<br><br>If the outcomes were assessed using observer reported, or self-reported scales, the risk of over- or under-reporting is increased, and objectivity is compromised. Importantly, determine if the measurement tools used were validated instruments as this has a significant impact on outcome assessment validity.	0<br>.5<br>1	Self-reported measures and assessments were used.<br>A self-reported measure was used, which has been psychometrically validated.<br>An external assessment took place.
**Measuring of Condition**	Was the condition measured in a standard, reliable way for all participants?<br><br>Considerable judgment is required to determine the presence of some health outcomes. Having established the validity of the outcome measurement instrument (see item 6 of this scale), it is important to establish how the measurement was conducted. Were those involved in collecting data trained or educated in the use of the instrument/s? If there was more than one data collector, were they similar in terms of level of education, clinical or research experience, or level of responsibility in the piece of research being appraised? When there was more than one observer or collector, was there comparison of results from across the observers? Was the condition measured in the same way for all participants?	0<br><br>1	Data was not gathered in the same way for all participants. For example, when one scale was not shown to all participants (HMS).<br>Data was gathered in the same way for all participants.
**Statistical Analysis**	Was there appropriate statistical analysis?<br><br>Importantly, the numerator and denominator should be clearly reported, and percentages should be given with confidence intervals. The methods section should be detailed enough for reviewers to identify the analytical technique used and how specific variables were measured. Additionally, it is also important to assess the appropriateness of the analytical strategy in terms of the assumptions associated with the approach as differing methods of analysis are based on differing assumptions about the data and how it will respond.<br><br>Points may be given per criteria that is fulfilled, leading to a maximum score of 1. In case no further criteria is provided, this results in a zero-point score.	0<br>.25<br>.25<br>.5	None of the beneath is provided.<br>Rungs and cut-offs of the used scales are given.<br>Score method of the used scales is given.<br>Percentages are given with confidence intervals.

**Response Rate**[33]	Was the response rate adequate, and if not, was the low response rate managed appropriately?<br>• A large number of dropouts, refusals or "not founds" amongst selected subjects may diminish a study's validity, as can low response rates for survey studies.<br>• The authors should clearly discuss the response rate and any reasons for non-response and compare persons in the study to those not in the study, particularly with regards to their socio-demographic characteristics.<br>• If reasons for non-response appear to be unrelated to the outcome measured and the characteristics of non-responders are comparable to those who do respond in the study (addressed in question 5, coverage bias), the researchers may be able to justify a more modest response rate.	0<br>.25<br><br><br>1	No response rate reported.<br>Response rate is provided yet is under < 45% and no further information is given.<br>OR<br>Response rate is provided verbally, "high" or "low" and no further information is given.<br>Response rate is under <45 % and a justification or argument is provided.<br>Response rate is < 45% and non-response analysis (e.g., sensitivity analysis) has been conducted, linking non-respondents to socio-demographic characteristics.<br>OR<br>Response rate is above ≥ 45%, no further information is needed.

Note. This table provides information on the refinement of the JBI quality assessment criteria. More information can be found in the main text (see critical appraisal).

[33] Fincham, J. E. (2008). Response rates and responsiveness for surveys, standards, and the journal. *American Journal of Pharmaceutical Education*, *72*(2), 43. https://doi.org/10.5688/aj720243; Galea, S., & Tracy, M. (2007). Participation rates in epidemiologic studies. *Annals of Epidemiology*, *17*(9), 643–653; Wu, M.-J., Zhao, K., & Fils-Aime, F. (2022). Response rates of online surveys in published research: A meta-analysis. *Computers in Human Behavior Reports*, *7*, 100206. https://doi.org/10.1016/j.chbr.2022.100206

Supplementary Table 13. Risk of bias (JBI) assessment by category and study (or sample in the case of the HMS and ISWS datasets)

Author	Sample Frame	Sampling Strategy	Sample Size	Study Description	Data Analysis	Assessment Strategy	Measuring of Condition	Statistical Analysis	Response Rate	Overall
Acharya et al., 2024	0	0	1	1	1	0	1	.25	1	5.25
Ahalli et al., 2022	1	1	1	1	1	.5	1	.25	1	7.75
Allen et al., 2020	.5	0	1	.5	1	.5	1	.5	.25	5.25
Almasri et al., 2022	0	0	1	.5	1	.5	1	0	1	5
Amaral-Prado et al., 2021	0	0	1	1	0	.5	1	.5	0	4
Andrade, 2023	0	0	1	1	0	.5	1	.25	0	3.75
Anttila et al., 2015	1	0	.5	1	1	.5	1	.25	.25	5.5
Aquino et al., 2018	0	0	0	.5	0	.5	1	.5	0	2.5
Aristeidou & Airstidou, 2023	1	0	1	.75	1	.5	1	.5	0	5.75
Baghoori et al., 2022	0	0	0	.25	0	.5	1	.5	.5	2.75
Barreira et al., 2018	0	0	0	1	1	.5	1	.5	1	5
Barry et al., 2018	0	0	0	1	0	.5	1	.25	0	2.75
Bastos et al., 2025	0	0	0	1	0	.5	1	.5	0	3
Bergmans & van der Weijden, 2021	0	0	0	1	1	.5	1	.25	0	3.75
Bitter & McCrea, 2023	0	0	0	1	0	.5	1	.25	0	2.75
Bonanomi et al., 2021	.5	0	1	1	1	.5	1	.25	0	5.25
Bonetto & Adam-Troian, 2022	0	0	0	1	1	.5	1	.25	0	3.75
Borgogna et al., 2021	0	0	1	1	0	0.5	1	.5	0	4
Bork-Young, 2023	0	0	0	1	0	.5	1	.5	0	3
Boyle & McKinzie, 2021	0	0	1	1	1	.5	1	.25	.5	5.25
Brownlow et al., 2022	0	0	0	.75	0	.5	1	.25	.25	2.75
Busch et al., 2024	0	0	1	1	1	.5	1	.25	0	4.75
Byrom & Metcalfe, 2020	0	0	1	1	1	.5	1	.5	0	5
Byrom et al., 2022	0	0	1	1	1	.5	1	.5	0.	5
Caesens et al., 2014	0	0	1	1	1	.5	1	.25	.25	5
Carr et al., 2022	1	0	1	1	1	.5	1	.75	0	6.25
Castelló et al., 2017	0	0	0	1	1	.5	1	.25	0	3.75
Cerio et al., 2024	0	0	0	.75	0	.5	1	.25	0	2.5
Charles et al., 2022	1	1	1	1	1	.5	1	.5	.25	7.25
Chen et al., 2024	0	0	1	1	1	.5	1	0	0	4.5
Cilli et al., 2023	0	0	0	1	1	.5	1	0	.25	3.75
Cullinan et al., 2020	.5	0	1	1	0	.5	1	.5	1	5.5
Dhont et al., 2020	0	0	1	.5	0	.5	1	.5	0	3.5
Divaris et al., 2011	0	0	0	1	0	.5	1	.25	1	3.75
Dong et al., 2024	0	0	1	1	0	.5	1	.25	0	3.75
Doom et al., 2021	0	0	1	1	0	.5	1	.25	0	3.75
Dumitrescu, 2016	0	0	0	1	1	.5	1	.25	0	3.75
Essadek et al., 2022	1	0	1	1	0	.5	1	1	.25	5.75
Estupiñá et al., 2024	0	0	1	1	1	.5	1	.25	1	5.75

Farrer et al., 2016	1	1	1	1	0	.5	1	.25	.25	6
Feizi et al., 2024	0	0	1	1	1	.5	1	.25	0	4.75
Fernández-Rodríguez et al., 2024	1	0	1	1	0	.5	1	.5	0	5
Friedrich et al., 2023	.5	0	1	1	1	.5	1	.5	.25	5.75
Frings et al., 2024	0	0	0	1	0	.5	1	.5	0	3
Gakhal, 2024	1	0	0	1	1	.5	1	0	0	4.5
Gallea et al., 2021	0	0	0	1	0	.5	1	.5	0	3
Gaudel et al., 2024	0	0	0	1	1	.5	1	.25	0	3.75
Gérard & Nagels, 2013	0	0	1	.75	1	0	1	.25	.25	4.25
Giménez Dasi et al., 2024	0	0	0	0	1	.5	1	.25	.5	3.25
Gloria & Steinhardt, 2013	0	0	0	1	1	.5	1	.5	.5	4.5
Guo et al., 2024	0	0	1	1	0	.5	1	.5	0	4
Hanitzsch et al., 2024	.5	0	1	1	0	.5	1	1	0	5
Hariharan et al., 2024	0	0	0	1	1	.5	1	0	0	3.5
Healthy Minds Study, 2007-2013 (2007)	1	1	1	1	1	.5	1	.5	.25	7.25
Healthy Minds Study, 2007-2013 (2009)	1	1	1	1	1	.5	1	.5	.25	7.25
Healthy Minds Study, 2007-2013 (2010)	1	1	1	1	1	.5	1	.5	.25	7.25
Healthy Minds Study, 2007-2013 (2011)	1	1	0	1	.5	.5	1	.5	.25	5.75
Healthy Minds Study, 2007-2013 (2012)	1	1	1	1	1	.5	1	.5	.25	7.25
Healthy Minds Study, 2013-2014	1	1	1	1	1	.5	1	.5	.25	7.25
Healthy Minds Study, 2014-2015	1	1	1	1	1	.5	1	.5	.25	7.25
Healthy Minds Study, 2015-2016	1	1	1	1	1	.5	1	.5	.25	7.25
Healthy Minds Study, 2016-2017	1	1	1	1	1	.5	0	.5	.25	6.25
Healthy Minds Study, 2017-2018	1	1	1	1	1	.5	0	.5	.25	6.25
Healthy Minds Study, 2018-2019	1	1	1	1	1	.5	0	.5	.25	6.25
Healthy Minds Study, 2019-2020	1	1	1	1	1	.5	0	.5	.25	6.25
Healthy Minds Study, 2020-2021	1	1	1	1	1	.5	0	.5	.25	6.25
Healthy Minds Study, 2021-2022	1	1	1	1	1	.5	0	.5	.25	6.25
Healthy Minds Study, 2022-2023	1	1	1	1	1	.5	0	.5	.25	6.25
Healthy Minds Study, 2023-2024 (students)	1	1	1	1	1	.5	0	.5	.25	6.25
Healthy Minds Study, 2023-2024 (staff)	0	0	1	1	1	.5	1	.5	.25	5.25
Healthy Minds Study, 2024-2025 (students)	1	1	1	1	1	.5	0	.5	.25	6.25
Healthy Minds Study, 2024-2025 (staff)	0	0	1	1	0	.5	1	.5	.25	4.25
Heinrich, 1979	0	0	0	.25	1	.5	1	0	0	2.75
Heming et al., 2024	0	0	0	1	1	.5	1	.5	.25	4.25

Herrell & Foster, 2024	0	0	0	1	0	.5	1	.25	0	2.75
Hill, 2010	0	0	0	.75	1	.5	1	.25	1	4.5
Hindman et al., 2015	0	0	0	1	0	.5	1	.25	0	2.75
Hish et al., 2019	0	0	0	1	1	.5	1	.25	.25	4
Hobaica et al., 2021	0	0	1	1	1	.5	0	.5	0	5
Hogg, 2023	.5	0	.5	1	1	.5	1	.5	0	5
Hu et al., 2024	0	0	1	1	1	.5	1	.5	1	6
Hulac et al., 2024	.5	0	0	.75	0	.5	1	.5	0	3.25
ISWS, 2021 (Bulgaria)	0	0	1	1	1	.5	1	.5	0	5
ISWS, 2021 (Canada)	0	0	1	1	1	.5	1	.5	0	5
ISWS, 2021 (Switzerland)	0	0	0	1	0	.5	1	.5	0	3
ISWS, 2021 (Cyprus)	0	0	0	1	0	.5	1	.5	0	3
ISWS, 2021 (Czechia)	0	0	1	1	1	.5	1	.5	0	5
ISWS, 2021 (Germany)	0	0	1	1	1	.5	1	.5	0	5
ISWS, 2021 (Denmark)	0	0	0	1	0	.5	1	.5	0	3
ISWS, 2021 (Spain)	0	0	0	1	0	.5	1	.5	0	3
ISWS, 2021 (Finland)	0	0	0	1	0	.5	1	.5	0	3
ISWS, 2021 (France)	0	0	0	1	0	.5	1	.5	0	3
ISWS, 2021 (GBR)	0	0	0	1	0	.5	1	.5	0	3
ISWS, 2021 (Greece)	0	0	0	1	0	.5	1	.5	0	3
ISWS, 2021 (Hungary)	0	0	1	1	1	.5	1	.5	0	5
ISWS, 2021 (Iceland)	0	0	0	1	0	.5	1	.5	0	3
ISWS, 2021 (Italy)	0	0	0	1	0	.5	1	.5	0	3
ISWS, 2021 (Netherlands)	0	0	0	1	0	.5	1	.5	0	3
ISWS, 2021 (Norway)	0	0	0	1	0	.5	1	.5	0	3
ISWS, 2021 (Portugal)	0	0	0	1	0	.5	1	.5	0	3
ISWS, 2021 (Romania)	0	0	0	1	0	.5	1	.5	0	3
ISWS, 2021 (Russia)	0	0	0	1	0	.5	1	.5	0	3
ISWS, 2021 (Slovakia)	0	0	0	1	0	.5	1	.5	0	3
ISWS, 2021 (Sweden)	0	0	0	1	0	.5	1	.5	0	3
ISWS, 2021 (Turkey)	0	0	0	1	0	.5	1	.5	0	3
ISWS, 2021 (USA)	0	0	0	1	0	.5	1	.5	0	3
ISWS, 2021 (South Africa)	0	0	0	1	0	.5	1	.5	0	3
Jackman et al., 2023	0	0	0	1	1	.5	1	.25	0	3.75
Jamshidi et al., 2017	0	0	1	.75	0	.5	1	0	1	4.25
Jing, 2021	0	0	1	1	1	.5	1	.5	0	5
Kaufman, 2004	0	0	0	1	1	.5	1	0	0	3.5
Kılıç & Karahan, 2023	0	0	1	.5	0	.5	1	.25	0	3.25
Kogien et al., 2023	0	0	1	1	0	.5	1	.5	1	5
Kosh, 2014	0	0	.5	.75	1	.5	1	.25	0	4
Kowalczyk et al., 2021	0	0	1	1	1	.5	1	.5	1	6
Krieger et al., 2025	.5	0	1	1	1	.5	1	.5	.25	5.75
Kusurkar et al., 2020	0	0	1	1	1	0	1	.25	1	5.25
Larcombe et al., 2022	.5	0	1	1	1	.5	1	.25	.25	5.5

Lento & Wick, 2024	0	0	0	1	1	0	1	.25	.25	3.5
Levecque et al., 2017	.5	0	1	1	1	.5	1	.5	.25	5.75
Li & Su 2024	0	0	1	1	1	.5	1	.5	0	5
Liu et al., 2019	0	0	1	1	1	.5	1	.5	1	6
Liu et al., 2020	0	0	1	1	0	.5	1	.25	1	4.75
Lowe, 2015	0	0	0	1	1	.5	1	.25	0	3.75
LT et al., 2022	1	1	1	1	1	.5	1	.5	0	7
Lubega et al., 2023	1	0	0	1	1	.5	1	.5	1	6
MacKinnon et al. 2024	0	0	1	1	1	.5	1	.5	0	5
Mao et al., 2022	0	0	1	.75	1	.5	1	.25	0	4.5
Marais et al., 2018	.5	0	0	.75	1	.5	1	.25	0	4
Martinez et al., 2024	0	0	1	1	0	.5	1	.25	.25	4
Mavrogalou-Foti et al., 2024	.5	0	0	1	1	.5	1	.25	0	4.25
McGregor et al., 2008	0	0	0	1	1	.5	1	.25	0	3.75
Miller & Orsillo, 2020	0	0	1	1	1	.5	1	.25	0	4.75
Mills et al., 2024	0	0	1	1	1	.5	1	.25	0	4.75
Moate et al., 2019	0	0	1	1	1	.5	1	.25	0	4.75
Müller et al., 2022	0	0	1	.75	1	.5	1	.5	.25	5
Muro et al. 2022	0	0	0	.75	1	.5	1	.5	0	3.75
Nagy et al., 2019	0	0	0	1	1	1	1	1	0	5
Naumann et al., 2022	.5	0	0	1	1	.5	1	.5	0	4.5
Nelson, 2014	0	0	0	.75	1	.5	1	.25	0	3.5
Nickel, 2024	0	0	0	1	1	.5	1	.5	0	4
Nottingham, 2017	.5	0	0	.75	1	.5	1	.5	0	4.25
Okun et al., 2022	.5	0	1	1	0	.5	1	.5	0	4.5
Oliveira de Freitas et al., 2023	0	0	1	1	0	.5	1	.5	1	5
Panayidou & Priest, 2021	.5	0	0	.5	1	.5	1	.25	0	3.75
Panger et al., 2017	1	1	1	.75	1	.5	1	.5	.25	7
Park et al., 2021	0	0	0	1	0	.5	1	.5	0	3
Pausik et al., 2022	0	0	0	1	1	.5	1	.25	0	3.75
Pervez et al., 2020	0	0	0	.75	1	.5	1	.5	.25	4
Peters, 2007	0	0	0	.75	1	.5	1	.25	1	4.5
Petruziello et al., 2024a	0	0	0	1	1	.5	1	.25	0	3.75
Petruziello et al., 2024b	0	0	0	1	1	.5	1	.25	0	3.75
Pitt et al., 2021	1	0	0	.75	1	.5	1	.25	0	4.5
Pyhältö et al., 2020	.5	0	1	1	1	.5	1	.25	.25	5.5
Pyhältö et al., 2023	.5	0	1	1	1	.5	1	0	.25	5.25
Ramadoss et al., 2022	.5	0	0	.75	1	.5	1	.5	.25	4.5
Ramadoss & Horn, 2022	.5	0	0	.75	1	.5	1	.5	.25	4.5
Rukmanee et al., 2024	0	0	0	.75	0	.5	1	.5	0	2.75
Russell et al., 2025	0	0	1	1	1	.5	1	.5	0	5
Schad et al., 2022	0	0	1	1	1	.5	1	.5	0	5
Scheidler, 2008	0	0	0	1	1	.5	1	.25	0	4.75
Sellali & El Houda Lahiouel, 2022	0	0	0	.5	1	.5	1	.25	0	3.25

Seto et al., 2023	0	0	1	.75	1	.5	1	.25	.25	4.75
Shillington et al., 2023	0	0	0	1	0	.5	1	.25	0	2.75
Smith et al., 2024	0	0	1	.75	1	.5	1	.25	.25	4.75
Solms et al., 2024	.5	0	1	1	1	.5	1	.25	.25	5.5
Stubb et al., 2011	.5	0	1	1	1	.5	1	.25	.5	5.75
Sundström, 2022	0	0	1	1	1	.5	1	.25	.25	5
Sverdlik & Hall, 2019	0	0	1	1	1	.5	1	.25	0	4.75
Sverdlik et al., 2020	1	1	1	1	1	.5	1	.25	0	6.75
Syropoulos et al., 2021	0	0	1	1	1	.5	1	0	0	4.5
Tadee et al., 2025	1	0	.5	1	.5	.5	1	0	.25	4.75
Talarowska et al., 2023	0	0	1	1	1	.5	1	.5	1	6
Talkhi et al., 2020	1	1	0	1	0	.5	1	.25	1	5.75
Tan et al., 2023	0	0	1	1	0	.5	1	.5	1	5
Thiagarajah et al., 2023	1	1	1	1	1	.5	1	.5	0	7
Thompson, 2024	0	0	.5	1	1	.5	1	.5	0	4.5
Tommasi et al., 2022	0	0	0	.75	1	.5	1	.25	.25	3.75
Trang et al., 2024	0	0	1	.75	0	.5	1	.25	0	3.5
Tsybuliak et al., 2024	0	0	1	1	0	.5	1	.25	0	3.75
Tu et al., 2023	0	0	0	1	1	.5	1	.5	1	5
U-DOC, 2021	0	0	1	1	1	.5	1	.5	0	5
Van Beuningen, 2024	0	0	0	1	1	.5	1	0	0	3.5
van der Weijden & Teelken, 2023	0	0	1	1	1	.5	1	.5	0	5
Varley, 2021	0	0	0	1	1	0	1	.25	0	2.75
Vilser et al., 2024a	.5	0	1	1	1	.5	1	.5	.5	6
Vilser et al., 2024b	0	0	1	1	1	.5	1	.5	0	5
Vincent et al., 2023	0	0	0	1	1	.5	1	.5	0	4
Wang et al., 2022	0	0	0	1	0	.5	1	.5	0	3
Wang et al., 2020	.5	0	1	1	1	,5	1	.25	0	5.25
Williams et al., 2014	0	0	0	1	1	.5	1	0	0	3.5
Wright, 2006	0	0	0	1	1	.5	1	0	0	3.5
Yssldyk et al., 2019	0	0	0	1	1	.5	1	.25	0	3.75
Zhang et al., 2024	1	0	1	1	1	.5	1	.5	.25	6.25

Note. This table provides information on the overall study quality, as well as the respective points per category. The study quality was assessed per sample and is based on the operationalization in Supplementary Table 11. More information can be found in the main text (see critical appraisal).

Supplementary Figure 3. Study quality ratings

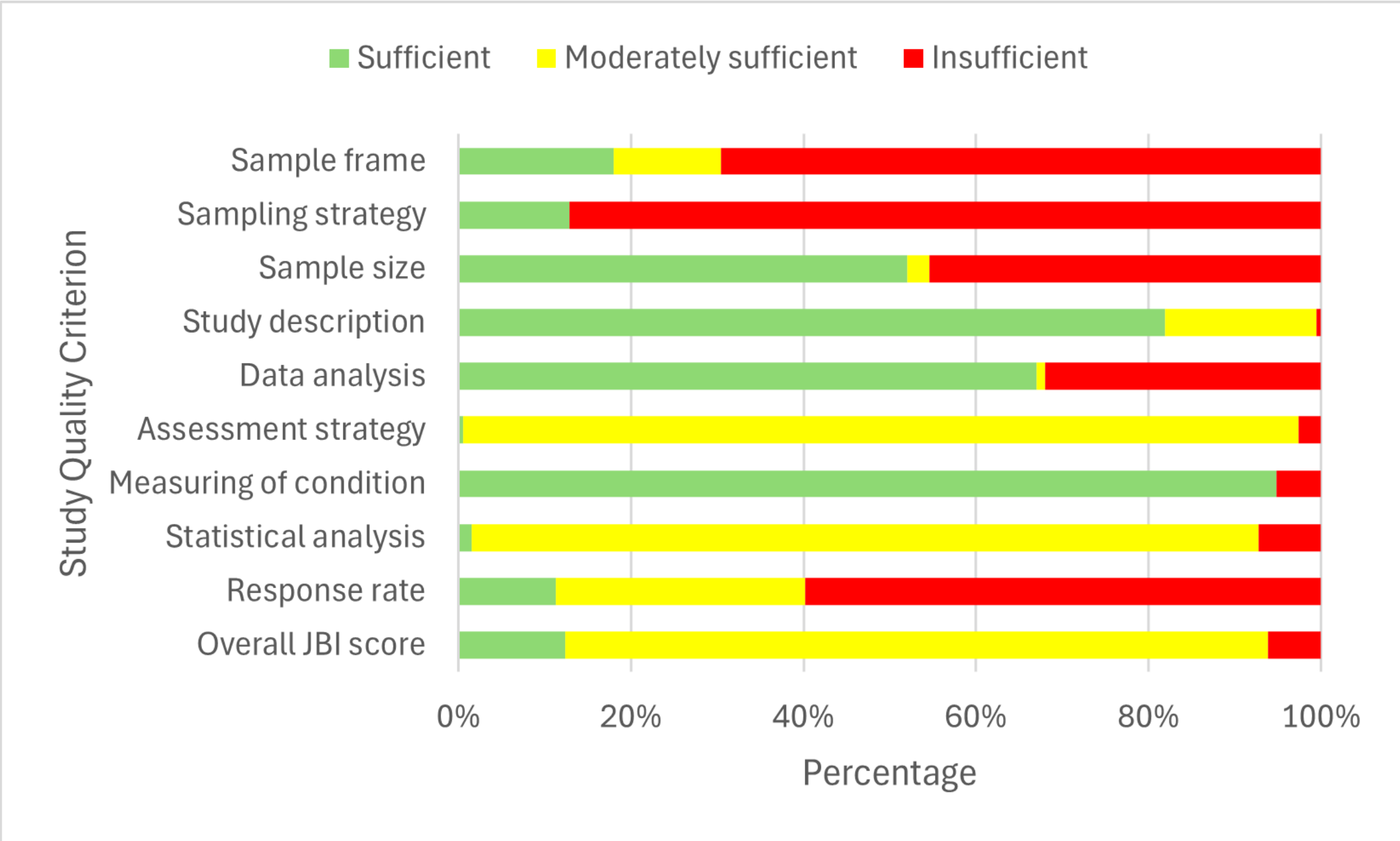

Note. The horizontal bar chart shows the percentage of studies rated as *insufficient* (red), *moderately sufficient* (yellow), and *sufficient* (green) across various methodological quality criteria based on the JBI tool (see Supplementary Table 11 for more information and Supplementary Table 12 for individual ratings). While most criteria were rated as sufficient in the majority of studies—especially for "study description," "measuring of condition," and "data analysis"—certain areas, notably "response rate," "sample strategy," and "sample size," had a high proportion of studies rated as insufficient. This suggests that while reporting and measurement were generally strong, study sampling and response rate were frequently limited.

Supplementary Table 14. Effect size heterogeneity**,** with arcsine-transformed effect sizes for the prevalence model

	I^2			τ	
**Model**	**Level 2**	**Level 3**	**Total**	**Level 2**	**Level 3**
POMP scores	52.96%	46.97%	99.93%	12.23	11.52
Prevalence	27.67%	71.86%	99.53%	0.11	0.18

Note. POMP = percent of maximum possible; τ = variability (standard deviation) of true effect sizes on Levels 2 and 3; I^2 = heterogeneity of effect sizes on Levels 2 and 3.

Supplementary Table 15. Model fit statistics for two- and three-level models

	**AIC**	**BIC**	**LRT**
**POMP SCORES**			
Three-level model	4414.12	4426.97	
Two-level model	4560.04	4568.60	147.92***
**PREVALENCE**			
Three-level model	-303.48	-291.90	
Two-level model	-63.13	-55.40	242.36***

Note. AIC = Akaike information criterion; BIC = Bayesian information criterion; LRT = likelihood ratio test; POMP = percent of maximum possible.

***$p < .001$

Supplementary Figure 4. Forest plot for the POMP-score model by year

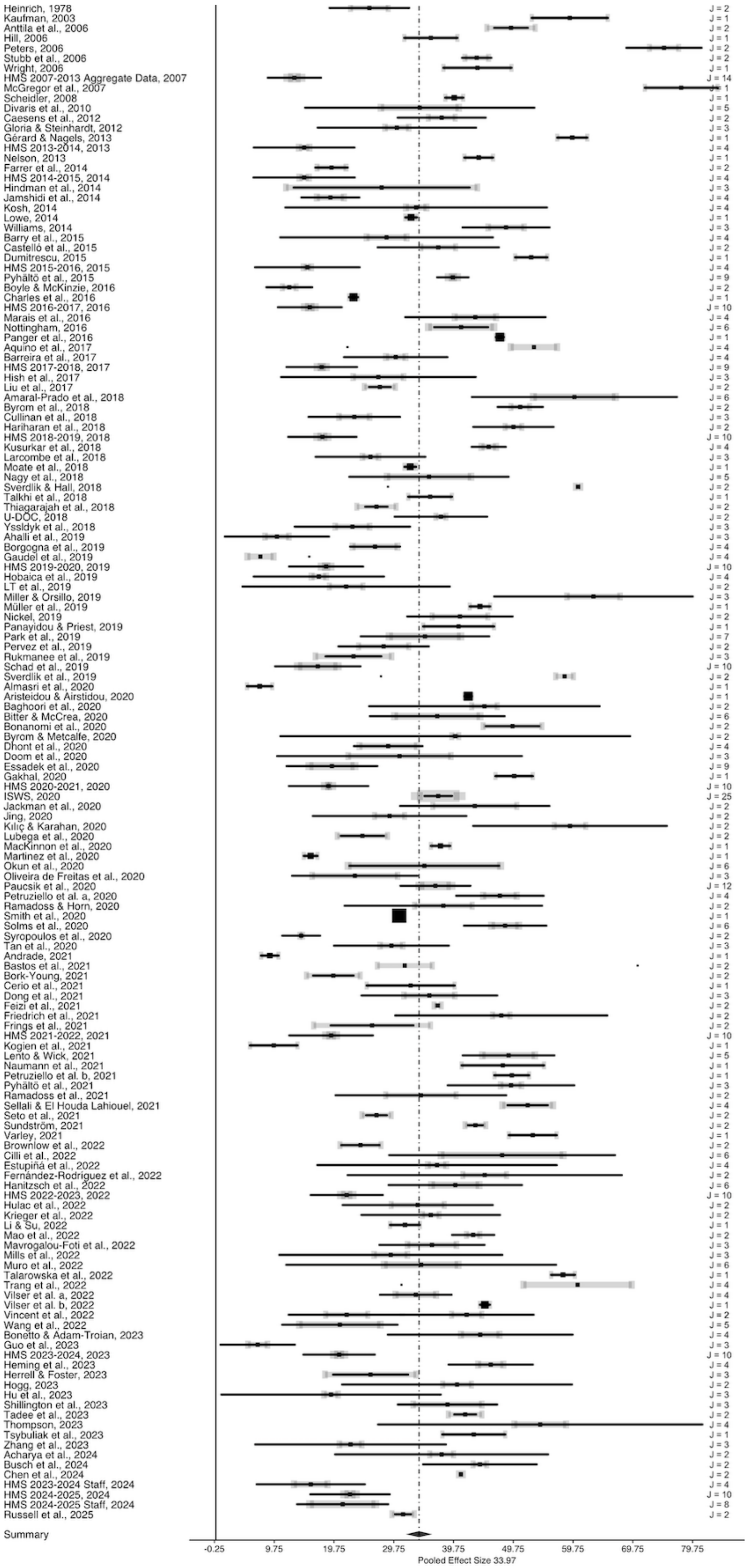

Note. The plot displays pooled estimates (POMP scores) and 95% confidence intervals for each study. The year noted behind the author details refers to the year in which the study was conducted. The diamond at the bottom represents the overall pooled estimate across all subtypes. J = number of effect sizes per study; HMS = healthy minds study; U-DOC = understanding doctoral researcher mental health; ISWS = COVID-19 international student wellbeing study.

Supplementary Figure 5. Forest plot for the POMP-score model by author

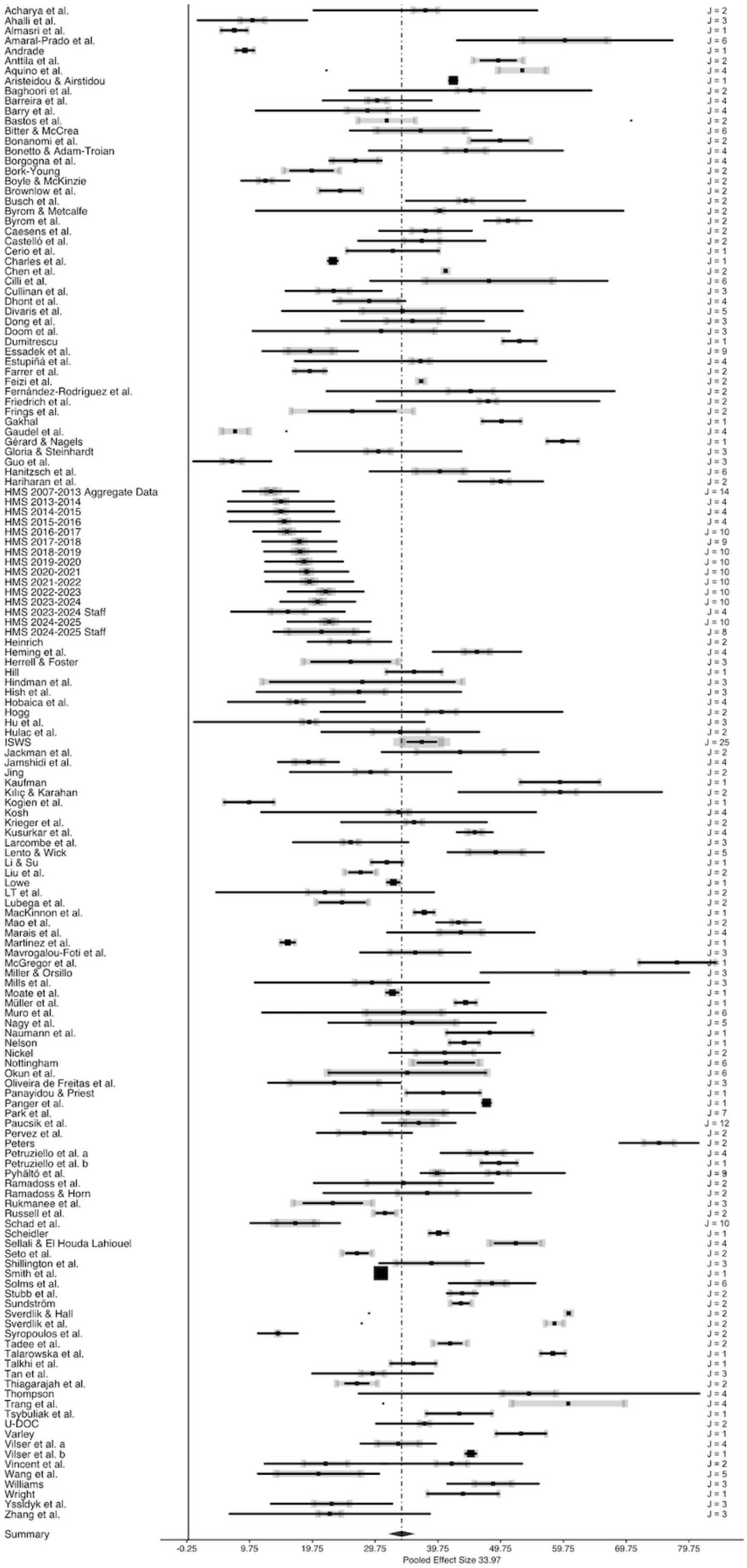

Note. The plot displays pooled estimates (POMP scores) and 95% confidence intervals for each study. The year noted behind the author details refers to the year in which the study was conducted. The diamond at the bottom represents the overall pooled estimate across all subtypes. J = number of effect sizes per study; HMS = healthy minds study; U-DOC = understanding doctoral researcher mental health; ISWS = COVID-19 international student wellbeing study.

Supplementary Figure 6. Forest plot for the POMP-score model by outcome

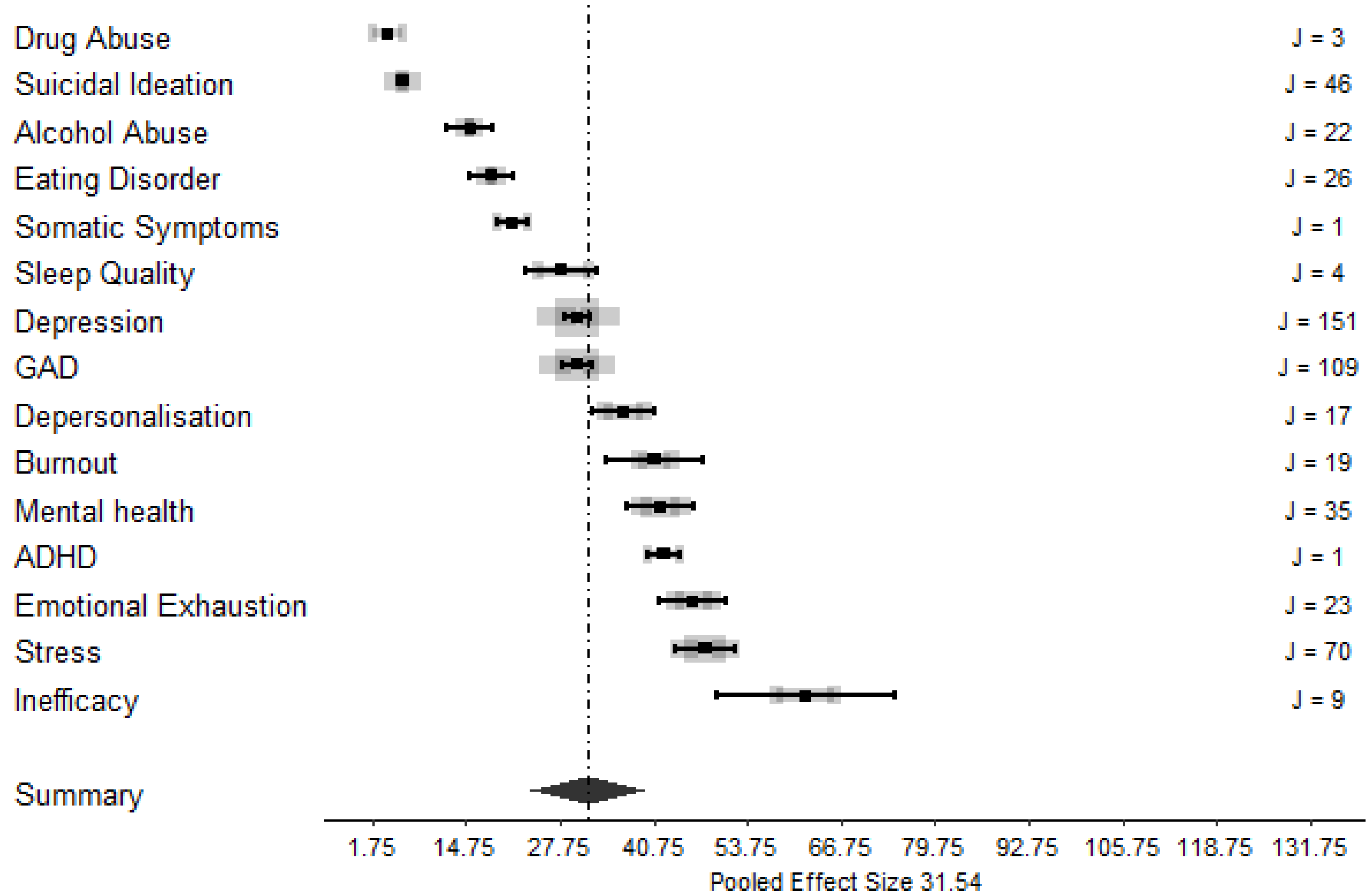

Note. The plot displays pooled estimates (POMP scores) and 95% confidence intervals for each subtype of psychological distress. The diamond at the bottom represents the overall pooled estimate across all subtypes. The vertical dashed line indicates the overall mean effect size (31.68). J = number of effect sizes per study; ADHD = attention deficit hyperactivity disorder; GAD = generalized anxiety disorder.

Supplementary Figure 7. Forest plot for the POMP-score model by instrument

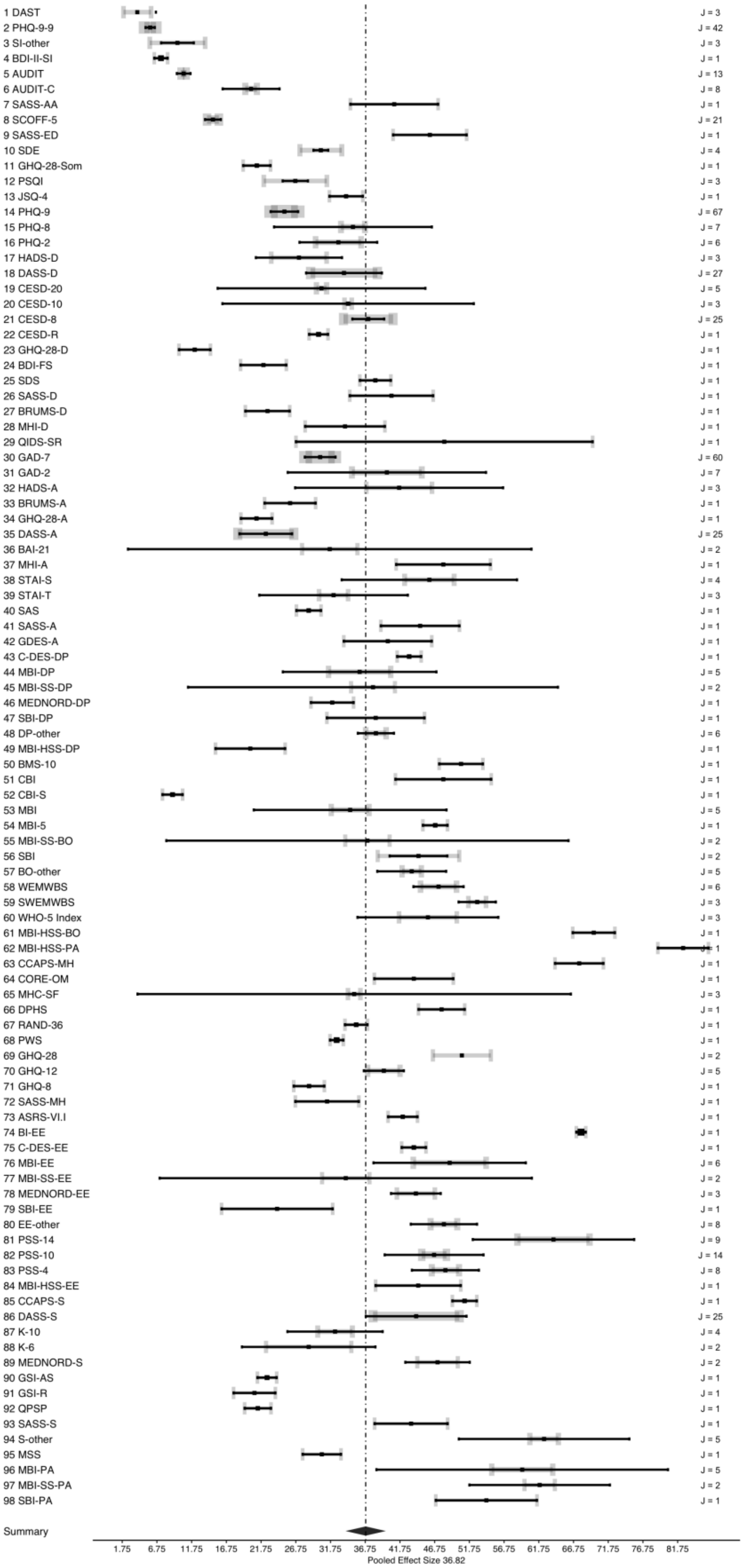

Note. The plot displays pooled estimates (POMP scores) and 95% confidence intervals for each scale ordered by type of syndrome and symptom and following the structure of the previous forest plot. The diamond at the bottom represents the overall pooled estimate across all subtypes. J = number of effect sizes per study; DAST = drug abuse screening test.

Supplementary Figure 8. Caterpillar plots for the POMP-score model

(A) by effect

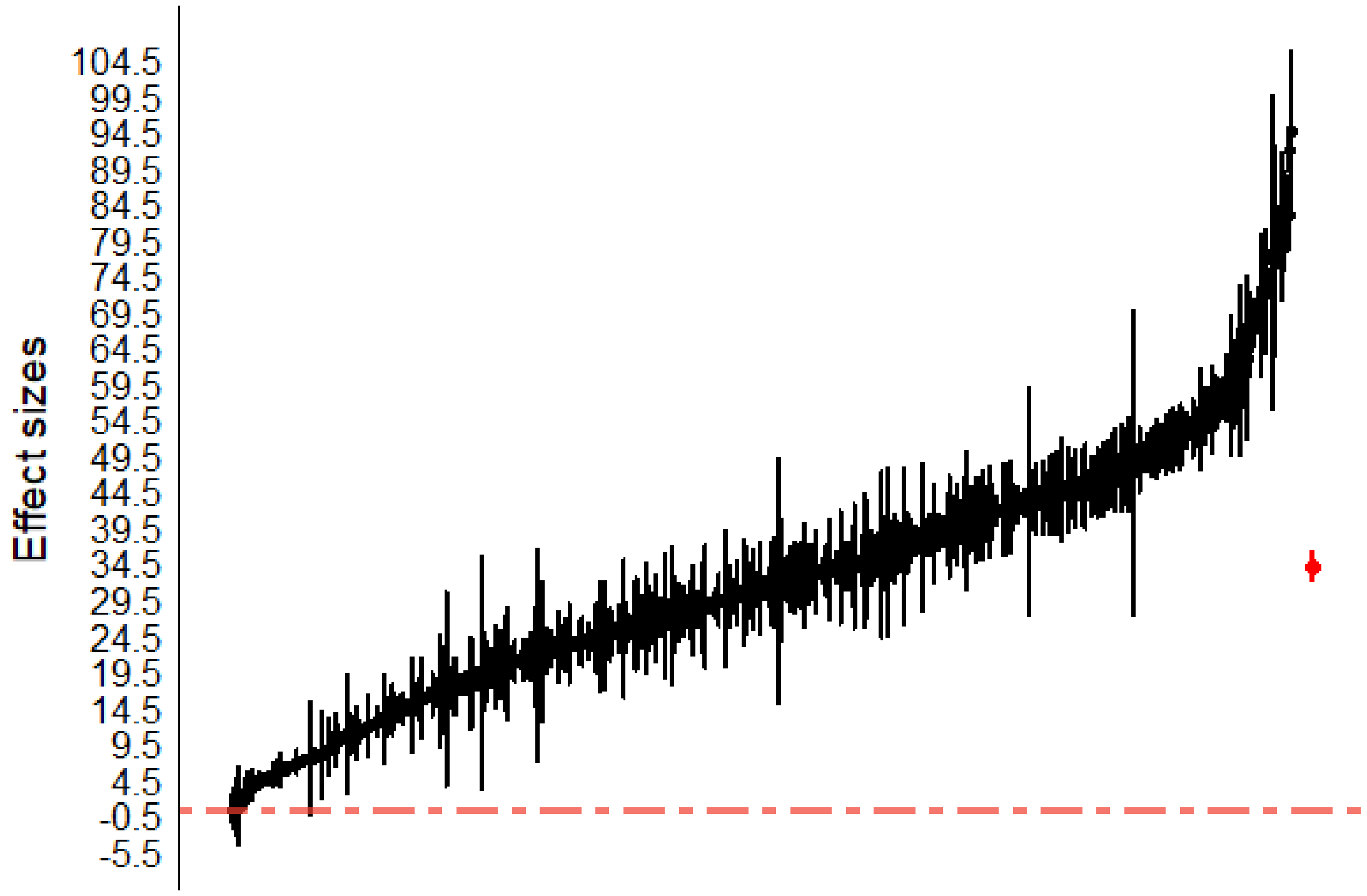

(B) by study effect

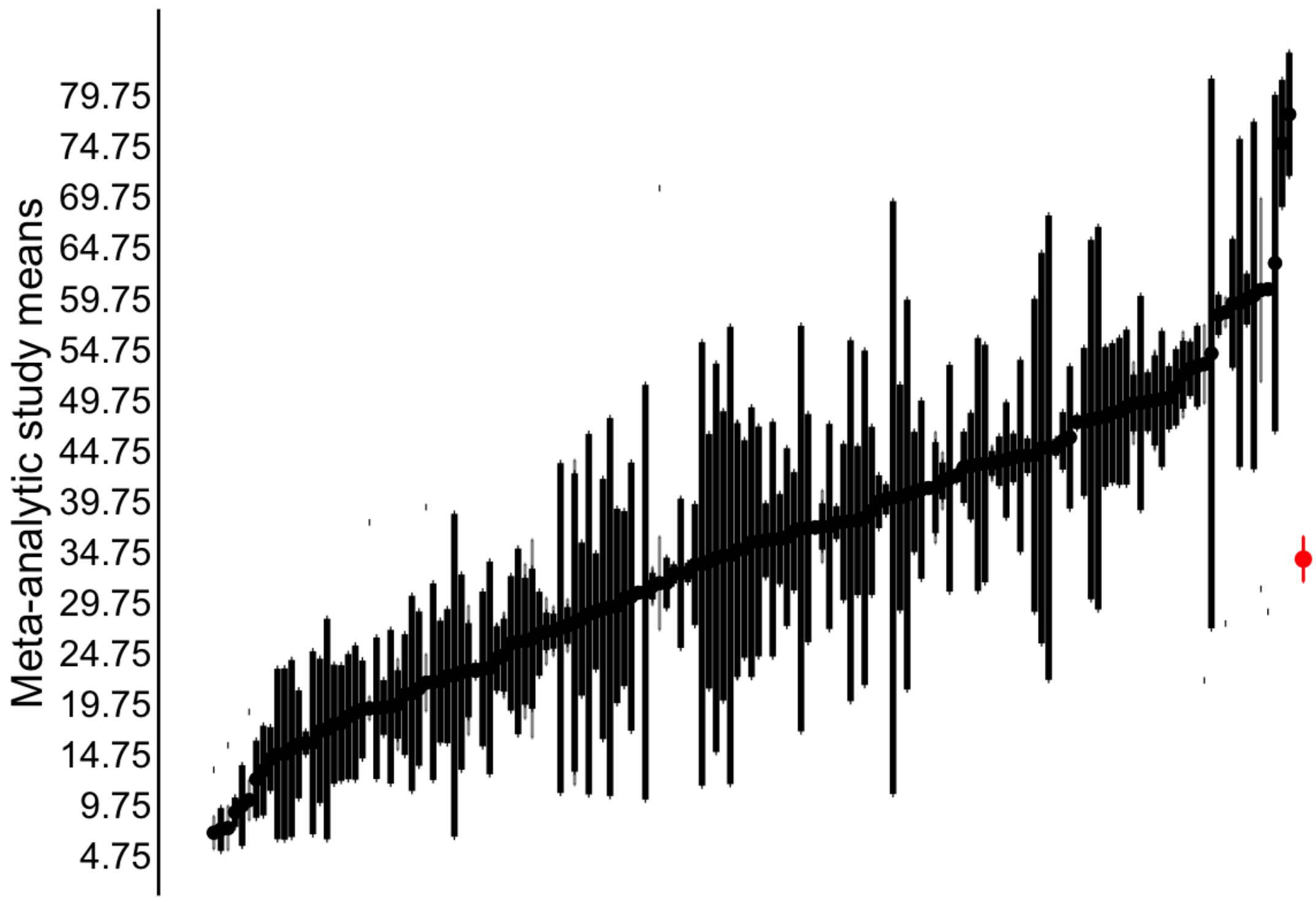

Supplementary Figure 9. Forest plot for the prevalence model by year with arcsine-transformed effect sizes

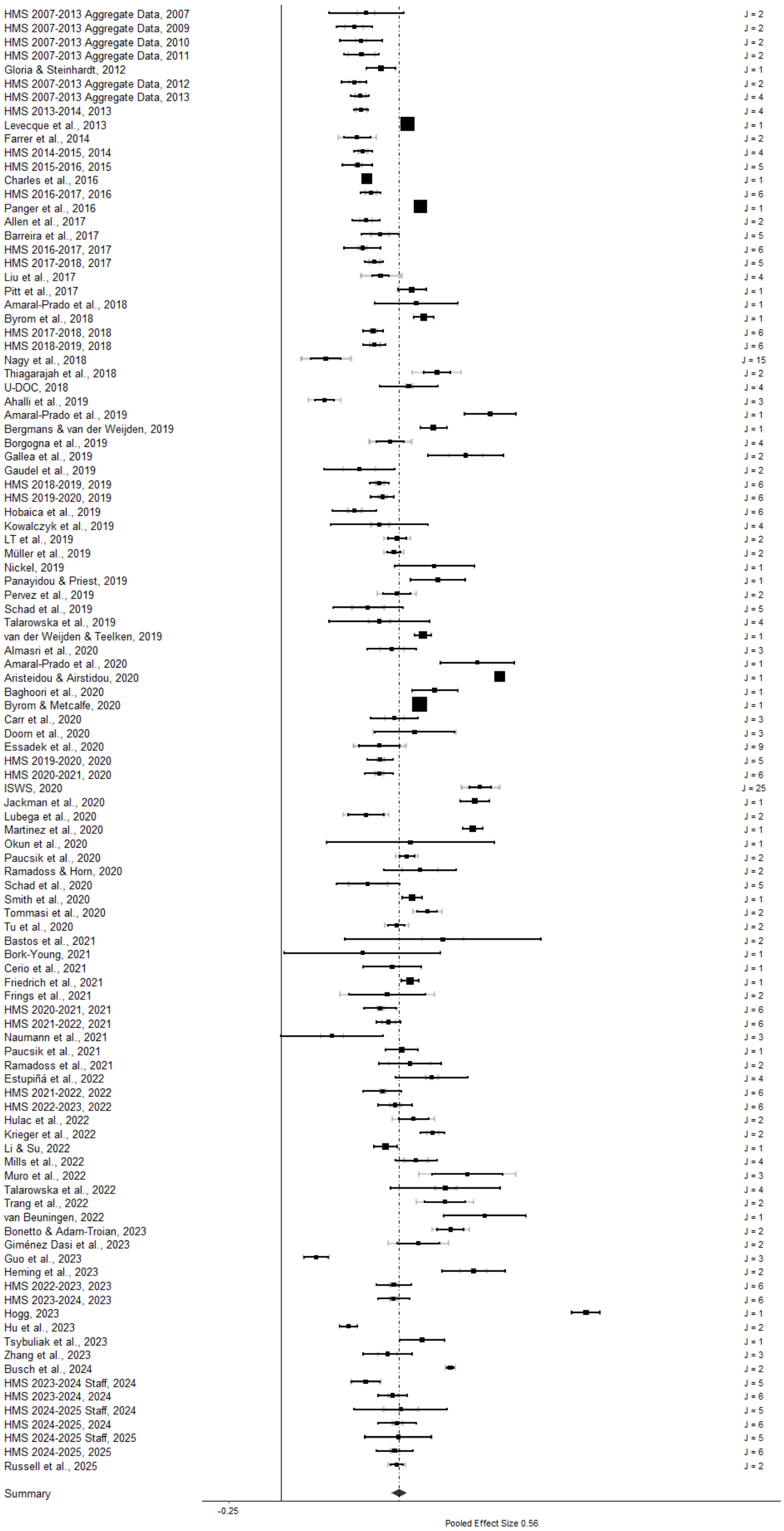

Note. The plot displays pooled estimates (prevalence) and 95% confidence intervals for each study included in the meta-analysis on prevalence. The year noted behind the author details refers to the year in which the study was conducted. The diamond at the bottom represents the overall pooled estimate across all subtypes. J = number of effect sizes per study; Pooled effect sizes can be back-transformed into prevalence using $[\sin(\text{effect size})]^2$; HMS = healthy minds study; U-DOC = understanding doctoral researcher mental health; ISWS = COVID-19 international student wellbeing study.

Supplementary Figure 10. Forest plot for the prevalence model by author with arcsine-transformed effect sizes

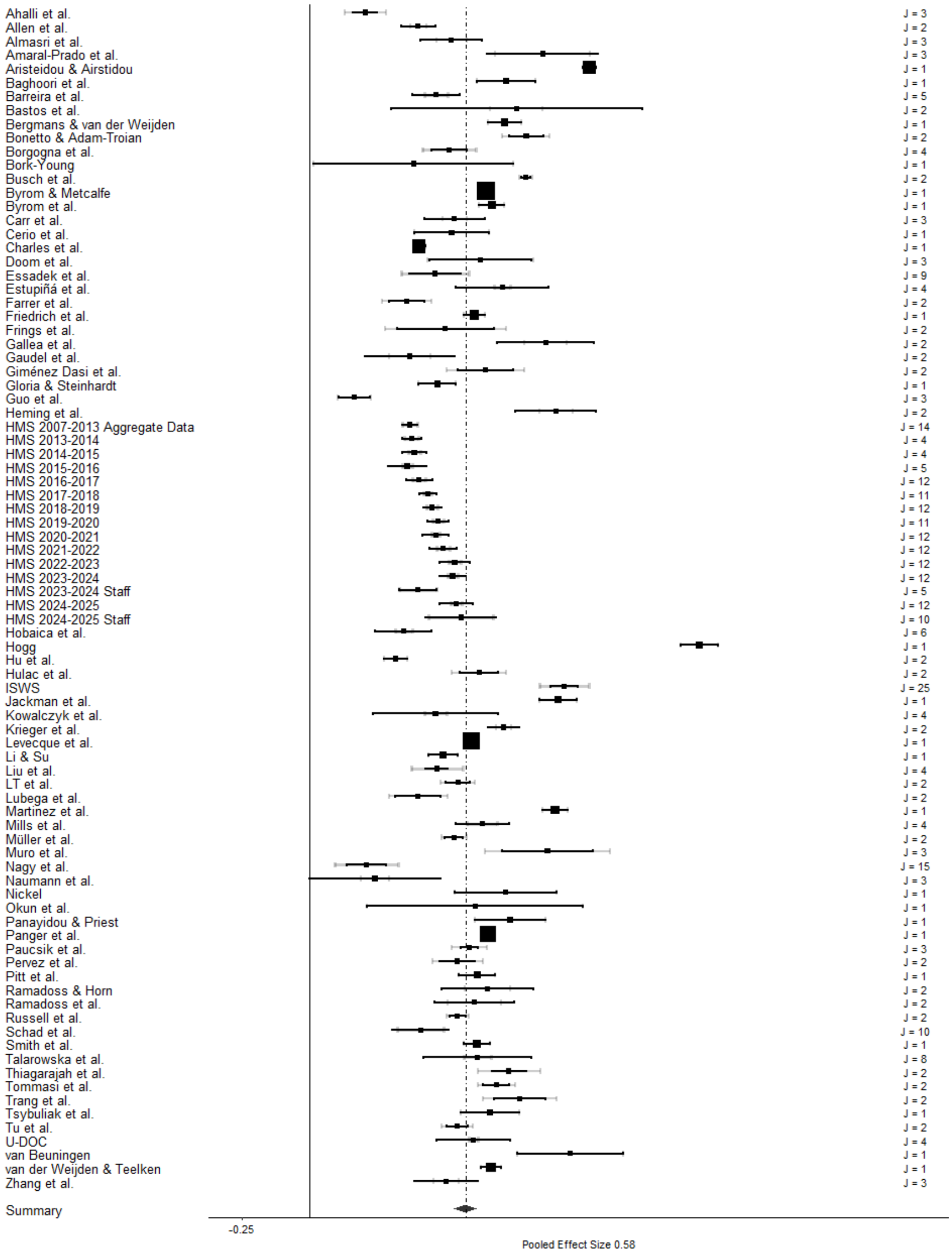

Note. The plot displays pooled estimates (prevalence) and 95% confidence intervals for each study included in the meta-analysis on prevalence. The year noted behind the author details refers to the year in which the study was conducted. The diamond at the bottom represents the overall pooled estimate across all subtypes. J = number of effect sizes per study; Pooled effect sizes can be back-transformed into prevalence using [sin(effect size)]²; HMS = healthy minds study; U-DOC = understanding doctoral researcher mental health; ISWS = COVID-19 international student wellbeing study.

Supplementary Figure 11. Forest plot for the prevalence model by outcomes with arcsine-transformed effect sizes

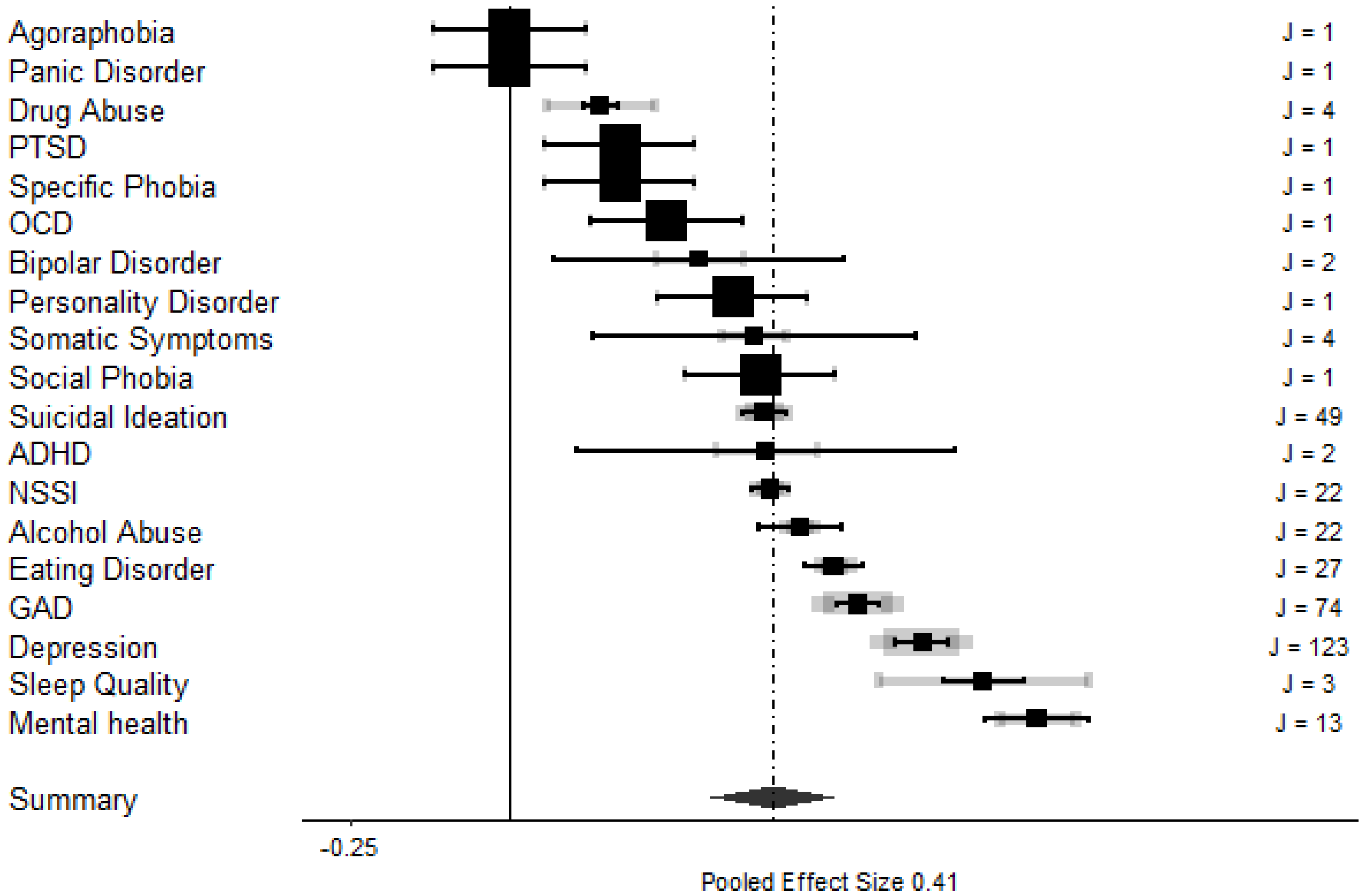

Note. The plot displays pooled estimates (prevalence) and 95% confidence intervals for each subtype of psychological distress. The diamond at the bottom represents the overall pooled estimate across all subtypes. ADHD = attention deficit hyperactivity disorder; GAD = generalized anxiety disorder; NSSI = non-suicidal self-injury; OCD = obsessive-compulsive disorder; PTSD = posttraumatic stress disorder; J = number of effect sizes per study. Pooled effect sizes can be back-transformed into prevalence using $[\sin(\text{effect size})]^2$.

Supplementary Figure 12. Caterpillar plots for the prevalence model with arcsine-transformed effect sizes

(A) per effect

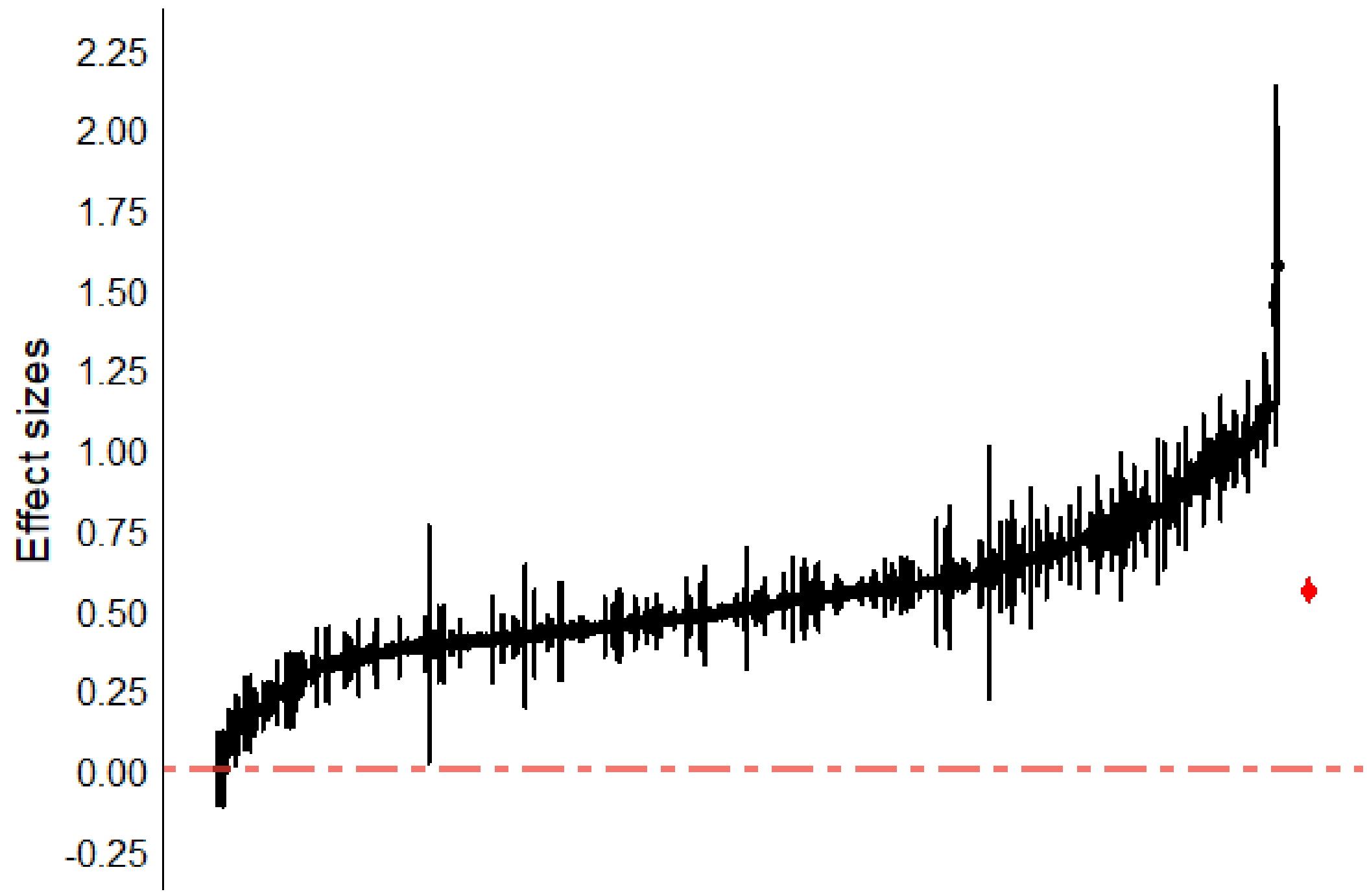

(B) per study effect

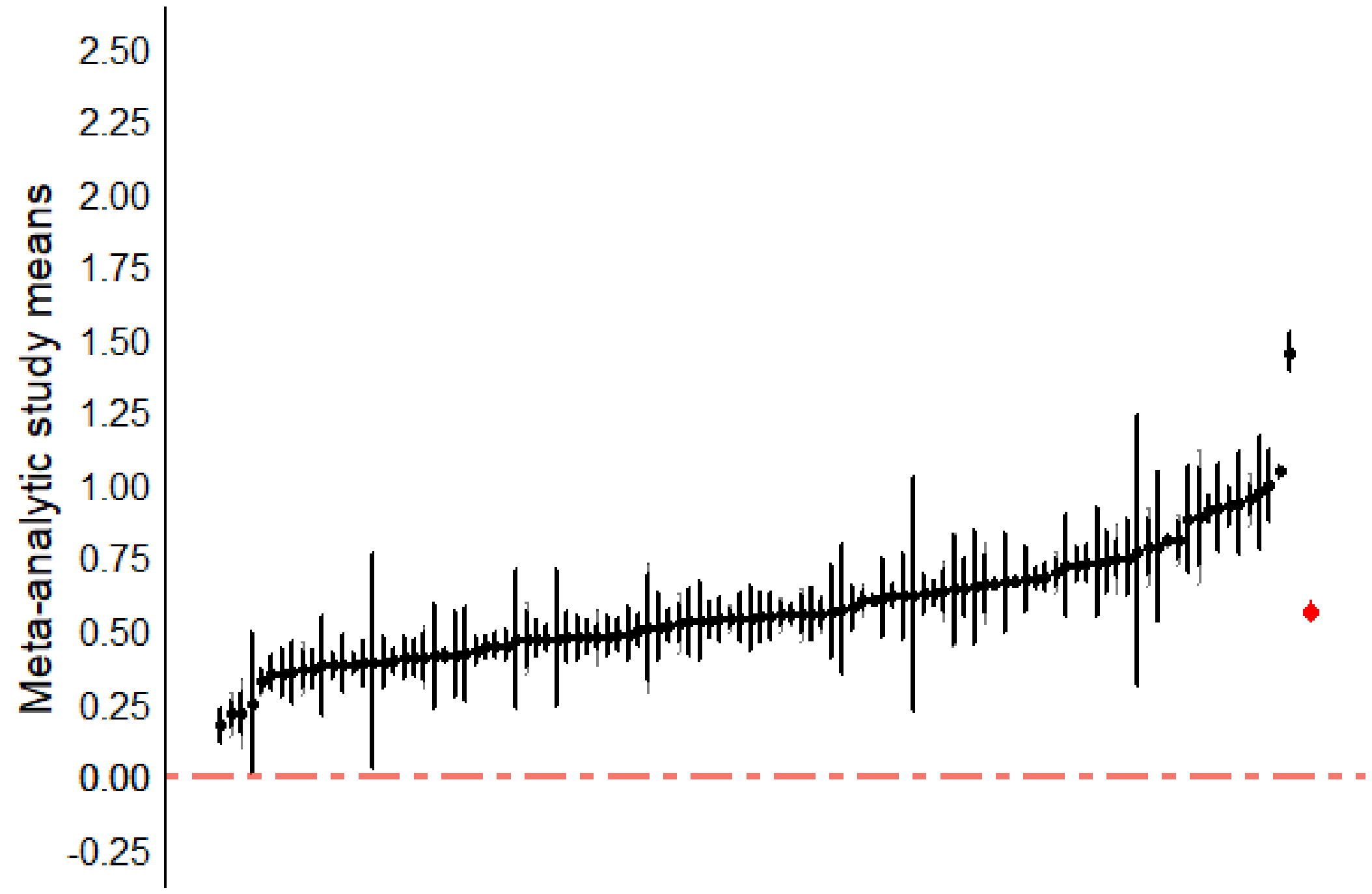

Note. Pooled effect sizes can be back-transformed into prevalence using [sin(effect size)]^2.

Supplementary Table 16. Post-hoc exploratory and sensitivity analyses in the severity and prevalence models

						95% CI					
**Model**	**Moderator**	k_{study}	k_{sample}	**#ES**	**β**	**LL**	**UL**	$F(df_1, df_2)$	p	R^2	Q (df)
**Severity**											
	**PHQ-9**							0.13(1, 534)	.722	0.45%	841,106.98(536)***
	Other scales (intercept)	151	214	469	33.83***	31.55	36.11				
	PHQ-9	44	67	67	0.63	-2.86	4.14				
	**GAD-7**							16.85(1, 534)	.001	2.72%	833,882.77(534)***
	Other scales (intercept)	148	211	476	33.17***	30.82	35.52				
	GAD-7	44	60	60	7.45***	3.88	11.02				
	**Burnout**							15.97(2, 533)	.001	3.96%	698,413.18(533)***
	Emotional exhaustion (intercept)	19	23	23	33.66***	31.44	35.88				
	Depersonalization/cynicism	14	17	17	-3.95	-10.62	2.71				
	Personal accomplishment	9	9	9	23.79***	14.91	32.69				
	**Alternative academic discipline clustering**							0.17(4, 56)	0.950	1.69%	53,139.45(56)***
	Applied sciences (intercept)	12	30	59	35.71***	14.84	56.59				
	Humanities	12	30	59	-0.20	-0.94	0.54				
	Social sciences	12	30	59	-0.06	-0.34	0.22				
	Formal sciences	12	30	59	0.48	-1.43	2.39				
	Natural sciences	12	30	59	0.01	-0.33	0.36				
**Prevalence**											
	**PHQ-9**							13.09(1, 350)	.001	1.68%	31,671.33(350)***
	Other scales (intercept)	84	133	284	28.87***	24.99	32.92				
	PHQ-9	46	68	68	0.38***	0.08	0.91				
	**GAD-7**							3.60(1, 350)	.058	1.31%	32,028.75(350)***
	Other scales (intercept)	82	134	291	29.27***	24.44	33.32				
	GAD-7	47	61	61	0.12	-0.00	0.49				
	**Alternative academic discipline clustering**							0.37(4, 40)	0.83	9.73%	5,963.24(40)***
	Applied sciences (intercept)	10	28	45	35.71***	16.30	57.96				
	Humanities	10	28	45	-0.00	-0.01	0.00				
	Social sciences	10	28	45	-0.00	-0.00	0.00				
	Formal sciences	10	28	45	-0.00	-0.03	0.00				
	Natural sciences	10	28	45	-0.00	-0.00	0.00				

Note. For categorical moderators, baseline categories are indicated by the term ‘intercept’ in parentheses; estimates of the other categories are deviances to the respective baseline categories. Prevalence data were back-transformed into percentages. The alternative academic discipline clustering deviates from the original one found in the supplementary Table 2 in the following way: Psychology is no longer included in the broader discipline of social sciences, but classified as a medical discipline. Thereby falling into the category of applied sciences. No other changes have been undertaken for the sensitivity analysis regarding academic

discipline. k_{study} = number of studies; k_{sample} = number of samples; #ES = number of effect sizes; β = pooled effect size estimate; *CI* = Confidence interval; LL = lower limit; UL = upper limit R^2 = amount of explained true effect-size variance.

*$p < .05$, **$p < .01$, ***$p < .001$

Supplementary Table 17. Results of the moderator analysis

Model	Moderator	k_{study}	k_{sample}	#ES	β	95% CI		$F(df_1, df_2)$	adjusted *p*	R^2	*Q* Test (*df*)
						LL	UL				
**Severity**											
	**Syndrome or symptom domain** (depression in intercept)	151	214	536				31.27(14, 521)	.005	48.67%	250,198.24(521)***
	**Utilized scale** (PHQ-9 in intercept)	151	214	536				10.15(97, 438)	.005	53.47%	95,277.32(438)***
	**Sample Characteristics**										
	**Country clusters**							0.77(5, 530)	.838	0.95%	449,473.67(530)***
	Cluster 1 (intercept)	89	110	321	33.03 ***	30.21	35.85				
	Cluster 2	24	39	86	2.89	-2.23	8.81				
	Cluster 3	15	26	48	4.88	-1.53	11.28				
	Cluster 4	17	22	43	-1.88	-8.57	4.81				
	Cluster 5	11	14	32	2.10	-6.12	10.33				
	Cluster 6	3	3	6	-0.71	-17.18	15.75				
	**US sample**							3.31(1, 534)	.031	2.48%	849,127.79(534)***
	US sample (intercept)	89	130	278	31.58***	28.28	34.87				
	Non-US sample	65	84	258	4.00	-0.32	8.32				
	**Age** (mean-centered)	101	148	398	-0.14	-0.64	0.36	0.29(1, 396)	.838	0.97%	624,796.62(396)***
	**Gender**							3.69(2, 208)	.099	8.01%	253,336.15(208)***
	Male and other (intercept)	51	67	211	1.68	-19.33	22.69				
	Female	51	67	211	0.44**	0.12	0.76				
	Diverse	51	67	211	0.13	-1.11	1.36				
	**ECR status**							3.58(1, 534)	.185	0.12%	862,698.83(534)***
	Pre-doctoral (intercept)	144	198	493	34.43***	32.12	36.74				
	Post-doctoral	14	16	43	-6.38	-13.01	0.24				
	**Ethnicity**							1.61(3, 183)	.457	7.79%	217,212.12(183)***
	Caucasian (intercept)	39	65	187	23.56***	17.63	24.49				
	Asian	39	65	187	0.18	-0.32	0.69				
	Latin	39	65	187	0.51	-0.01	1.03				
	Black	39	65	187	-0.01	-0.07	0.05				
	**Academic discipline**							0.16(4, 56)	.958	5.71%	56,516.81(56)***
	Applied sciences (intercept)	13	30	61	35.30**	18.82	56.78				
	Humanities	13	30	61	-0.20	-0.95	0.54				
	Social sciences	13	30	61	0.05	-0.34	0.24				
	Formal sciences	13	30	61	0.51	-1.39	2.42				
	Natural sciences	13	30	61	0.02	-0.31	0.36				
	**Risk and protective factors**										
	**History of mental health diagnosis** (mean-centered)	19	28	112	-0.06	-0.36	0.24	0.16(1, 110)	.838	5.93%	89,603.96(110)***
	**Loneliness** (POMP Score, mean-centered)	11	16	64	0.12	-0.36	0.59	0.25(1, 62)	.838	2.66%	69,906.66(62)***
	**Social support** (POMP Score, mean-centered)	9	9	22	0.02	-0.36	0.39	0.01(1, 20)	.933	5.07 %	1,946.14(20)***

	**Advisor relationship** (POMP Score, mean-centered)	12	14	37	-0.02	-0.47	0.43	0.01(1, 35)	.933	4.90%	41,651.13(35)***
	**Life satisfaction** (POMP Score, mean-centered)	13	13	28	-0.15	-0.76	0.47	0.23(1, 26)	.838	5.64%	10,981.50(26)***
	**Job satisfaction** (POMP Score, mean-centered)	3	4	8	-0.53	-1.66	0.60	1.32(1, 6)	.614	5.08%	477.60(6)***
	**Study characteristics**										
	**COVID-19**							0.37(2, 533)	.838	0.25%	656,172.29(533)***
	Before COVID-19 (intercept)	68	81	220	33.81***	30.62	36.99				
	During COVID-19	70	108	239	0.82	-3.32	4.97				
	After COVID-19	20	25	77	-1.91	-8.38	4.56				
	**Year**							0.52(2, 533)	.838	0.51%	682,843.53(533)***
	Year (set to zero, mean-centered)	151	214	536	0.32	-1.28	1.91				
	Year^2	151	214	536	-0.01	-0.03	0.02				
	**Peer review**							3.24(1, 534)	.201	1.88%	8469,647.39(534)***
	Peer-reviewed record (intercept)	108	130	322	35.21***	32.56	37.86				
	Non-peer-reviewed record	45	84	214	-4.36	-9.13	0.40				
	**Machine-translated record**							1.04(1, 534)	.614	0.11%	863,102.68(534)***
	Original-language record (intercept)	150	211	530	34.05***	31.79	36.31				
	Machine-translated record	3	3	6	-8.79	-25.70	8.11				
	**Study quality**										
	**Overall study quality**	151	214	536	-3.06***	-4.65	-1.46	14.14(1, 534)	.005	7.69%	842,444.53(534)***
	**Sampling strategy**							41.28(1, 534)	.005	15.31%	863,425.95(534)***
	Convenience sampling (intercept)	134	180	410	36.36***	34.25	38.49				
	Random or full sampling	19	34	126	-17.72***	-23.13	-12.29				
	**Assessment strategy**							0.02(1, 534)	.933	0.48%	864,676.29(534)***
	Self-assessment (intercept)	152	213	531	33.88***	31.63	36.14				
	External assessment	1	1	5	1.72	-23.72	27.15				
**Prevalence**											
	**Syndrome or symptom domain** (depression in intercept)	86	138	352				11.41(18, 333)	.007	21.24%	21,621.66(333)***
	**Utilized scale** (PHQ-9 in intercept)	86	138	352				9.65(49, 302)	.007	34.39%	10,995.34(302)***
	**Sample Characteristics**										
	**Country Clusters**							1.08(4, 347)	.546	0.28%	29,443.89(347)***
	Cluster 1 (intercept)	51	70	245	28.97***	24.29	33.89				
	Cluster 2	16	28	42	0.01	-1.03	0.68				
	Cluster 3	11	21	38	0.49	-0.07	2.79				
	Cluster 4	9	15	22	0.23	-0.47	2.67				
	Cluster 5	3	4	5	0.39	-5.77	1.35				
	Cluster 6	0	0	0							
	**US sample**							3.83(1, 350)	.015	5.12%	26,630.25(350)***
	US sample (intercept)	41	58	224	26.03***	21.01	31.39				
	Non-US sample	47	80	129	0.66	-0.00	2.63				
	**Age** (mean-centered)	61	110	296	-0.01*	-0.03	-0.00	5.20(1, 294)	.048	7.55%	30,333.33(294)***
	**Gender**							0.73(2, 195)	.655	4.08%	19,892.48(195)***
	Male and other (intercept)	38	53	198	17.00**	1.72	43.38				
	Female	38	53	198	0.00	-0.00	0.00				

	Diverse	38	53	198	0.01	-0.01	0.08				
	**ECR status**							0.03(1, 350)	.874	1.05%	32,543.70(350)***
	Pre-doctoral (intercept)	78	127	327	30.02***	25.95	34.25				
	Post-doctoral	9	11	26	0.02	-2.36	1.64				
	**Ethnicity**							0.34(3, 182)	.874	1.21%	17,331.64(182)***
	Caucasian (intercept)	28	54	186	21.32***	14.81	28.76				
	Asian	28	54	186	0.00	-0.00	0.00				
	Latin	28	54	186	-0.00	-0.00	0.00				
	Black	28	54	186	0.00	-0.00	0.02				
	**Academic discipline**							0.10(4, 54)	.874	8.37%	6,410.28(54)***
	Applied sciences (intercept)	14	33	59	40.29***	10.74	47.86				
	Humanities	14	33	59	-0.00	-0.01	0.01				
	Social sciences	14	33	59	-0.00	-0.00	0.00				
	Formal sciences	14	33	59	-0.00	-0.03	0.00				
	Natural sciences	14	33	59	-0.00	-0.00	0.00				
	**Risk and protective factors**										
	**History of mental health diagnosis** (mean-centered)	18	27	135	0.00**	0.00	0.01	7.45(1, 133)	.016	23.46%	11,660.50(133)***
	**Loneliness** (POMP Score, mean-centered)	11	16	78	-0.00*	-0.01	-0.00	4.13(1, 76)	.015	18.75%	8,413.85(76)***
	**Social support** (POMP Score, mean-centered)	3	3	4	-0.10	-1.22	0.25	2.64(1, 2)	.397	66.23%	3,384.57(2)***
	**Advisor relationship** (POMP Score, mean-centered)	7	7	18	-0.00	-0.01	0.00	0.41(1, 16)	.656	8.66%	1,060.54(16)***
	**Life satisfaction** (POMP Score, mean-centered)	6	6	12	-0.01*	-0.03	-0.00	4.70(1, 10)	.096	33.01%	451.81(10)***
	**Study characteristics**										
	**COVID-19**							6.49(2, 349)	.010	8.66%	29,012.68(349)***
	Before COVID-19 (intercept)	38	49	150	24.29***	19.86	29.03				
	During COVID-19	39	70	138	1.19 ***	0.24	2.85				
	After COVID-19	15	19	65	0.99*	0.00	3.66				
	**Year**							5.41(2, 349)	.015	6.14%	28,902.31(349)***
	Year (set to zero, mean-centered)	86	138	352	0.00	-0.12	0.12				
	$Year^2$	86	138	352	0.00	-0.00	0.00				
	**Peer review**							0.29(1, 350)	.686	0.74%	31,353.76(350)***
	Peer-reviewed record (intercept)	58	70	153	30.69***	25.89	35.72				
	Non-peer-reviewed record	29	68	200	-0.06	-1.29	0.42				
	**Machine-translated record**							0.46(1, 350)	.655	0.62%	32,456.37(350)***
	Original-language record (intercept)	85	136	349	30.10***	26.20	34.14				
	Machine-translated record	2	2	4	-0.97	-14.08	3.48				
	**Study quality**										
	**Overall study quality**	86	138	352	-0.14**	-0.40	-0.01	7.56 (1, 350)	.016	11.65%	27,645.71(350)***
	**Sampling strategy**							11.36(1, 350)	.007	9.45%	27,227.51(350)***
	Convenience sampling (intercept)	69	105	209	33.33***	29.07	37.74				
	Random or full sampling	18	33	144	-2.61***	-6.48	-0.46				
	**Assessment strategy**							4.12(1, 350)	.082	3.47%	32,221.08(350)***
	Self-assessment (intercept)	86	137	338	30.31***	26.52	34.24				
	External assessment	1	1	15	-13.08*	-44.30	-0.01				

Note. For categorical moderators, baseline categories are indicated by the term ‘intercept’ in parentheses; estimates of the other categories are deviances to the respective baseline categories. Prevalence data were back-transformed into percentages. Moderators outcome and scale are not provided in detail in this table, only the intercepts of the respective comparisons. k_{study} = number of studies; k_{sample} = number of samples; #ES = number of effect sizes; β = pooled effect size estimate; CI = confidence interval; LL = lower limit; UL = upper limit; adjusted *p* = false-discovery-rate adjusted *p* value; R^2 = amount of explained true effect-size variance. GAD = generalized anxiety disorder; ADHD = attention deficit hyperactivity disorder; EE = emotional exhaustion; DP = depersonalization; PA = personal accomplishment; NSSI = non-suicidal self-injury; Cluster 1 = high government support, structured contracts, and career security; Cluster 2 = flexible and grant-dependent research careers; Cluster 3 = competitive fellowships with limited benefits and short-term contracts; Cluster 4 = emerging research systems with varied funding sources and career paths; Cluster 5 = self-funded or low-funding systems; Cluster 6 = unique or hybrid systems.

$^{*}p < .05$, $^{**}p < .01$, $^{***}p < .001$

Supplementary Table 18. Robustness of moderator effects controlling for publication bias

Model	Moderator	k_{study}	k_{sample}	#ES	β	95% CI		$F(df_1, df_2)$	*p*	R^2	*Q* Test (*df*)
						LL	UL				
**Severity**											
	**Syndrome or symptom domain** (depression in intercept)	151	214	536				31.03(15, 520)	.001	49.34%	225,313.67(520)***
	**Utilized scale** (PHQ-9 in intercept)	151	214	536				10.55(98, 437)	.001	53.56%	87,522.18(437)***
	**Sample Characteristics**										
	**US sample**							13.76(2, 533)	.001	6.91%	692,782.22(533)***
	US sample	89	130	278	26.51***	22.73	30.29				
	Non-US sample	65	84	258	4.42***	0.21	8.63				
	**Study quality**										
	**Overall study quality**	151	214	536	-1.67	-3.43	0.08	13.39(2, 533)	.001	7.22%	689,894.72(533)***
	**Sampling strategy**										
	Convenience sampling (intercept)	134	180	410	32.58***	29.55	35.60	26.08(2, 533)	.001	15.24%	689,831.14(533)***
	Random or full sampling	19	34	126	-15.16***	-20.85	-9.47				
**Prevalence**											
	**Syndrome or symptom domain** (depression in intercept)	86	138	352				10.77(19, 332)	.001	21.65%	21,408.06(332)***
	**Utilized scale** (PHQ-9 in intercept)	86	138	352				9.41(50, 301)	.001	34.55%	10,976.39(301)***
	**Sample Characteristics**										
	**US sample**							3.22(2, 349)	.041	6.63%	26,628.19(349)***
	US sample	41	58	224	23.82***	18.42	29.69				
	Non-US sample	47	80	129	0.62	-0.00	2.54				
	**Age** (mean-centered)	61	110	296	-0.01*	-0.03	-0.00	4.11(2, 293)	.017	7.27%	29,742.41(293)***
	**Risk and protective factors**										
	**History of mental health diagnosis** (mean-centered)	18	27	135	0.002**	0.000	0.007	4.86(2, 132)	.009	27.24%	11,652.27(132)***
	**Loneliness** (POMP Score, mean-centered)	11	16	78	-0.003	-0.01	0.00	2.16(2, 75)	.122	15.39%	8,393.47(75)***
	**Study characteristics**										
	**COVID-19**							4.94(3, 348)	.002	9.81%	28,242.28(348)***
	Before COVID-19 (intercept)	38	49	150	22.57***	17.67	27.86				
	During COVID-19	39	70	138	1.10***	0.19	2.73				
	After COVID-19	15	19	65	0.98*	0.01	3.64				
	**Year**							4.31(3, 348)	.001	7.68%	28,082.97(348)***
	Year (set to zero, mean-centered)	86	138	352	0.00	-0.11	0.12				
	Year²	86	138	352	0.00	-0.00	0.00				
	**Study quality**										
	**Overall study quality**	86	138	352	-0.11*	-0.41	-0.001	3.82(2, 349)	.029	10.95%	27,567.52(349)***
	**Sampling strategy**										
	Convenience sampling (intercept)	69	105	209	31.62***	26.16	37.35	6.12(2, 349)	.002	9.54%	27,044.12(349)***
	Random or full sampling	18	33	144	-2.29**	-6.08	-0.29				

Note. For categorical moderators, baseline categories are indicated by the term ‘intercept’ in parentheses; estimates of the other categories are deviances to the respective baseline categories. Prevalence data were back-transformed into percentages. Moderators outcome and scale are not provided in detail in this table,

only the intercepts of the respective comparisons. k_{study} = number of studies; k_{sample} = number of samples*;* #ES = number of effect sizes; β = pooled effect size estimate; CI = confidence interval; LL = lower limit; UL = upper limit; adjusted *p* = false-discovery-rate adjusted *p* value; R^2 = amount of explained true effect-size variance. GAD = generalized anxiety disorder; ADHD = attention deficit hyperactivity disorder; EE = emotional exhaustion; DP = depersonalization; PA = personal accomplishment; NSSI = non-suicidal self-injury; Cluster 1 = high government support, structured contracts, and career security; Cluster 2 = flexible and grant-dependent research careers; Cluster 3 = competitive fellowships with limited benefits and short-term contracts; Cluster 4 = emerging research systems with varied funding sources and career paths; Cluster 5 = self-funded or low-funding systems; Cluster 6 = unique or hybrid systems.

$^{*}p < .05$, $^{**}p < .01$, $^{***}p < .001$

Supplementary Table 19. Benjamini-Hochberg FDR corrected *p* values

	**POMP scores**		**Prevalence**	
**Moderator**	**Raw *p* (*F* test)**	**BH-adjusted *p***	**Raw *p* (*F* test)**	**BH-adjusted *p***
**Outcome**	.001	.005	.001	.007
**Scale**	.001	.005	.001	.007
**Country cluster**	.570	.838	.364	.546
**US sample**	.007	.031	.005	.015
**Age** (mean-centered)	.588	.838	.023	.048
**Gender**	.027	.099	.485	.655
**ECR status**	.059	.185	.856	.874
**Ethnicity**	.187	.457	.794	.874
**Academic discipline**	.958	.958	.873	.874
**History of mental health diagnosis** (mean-centered)	.686	.838	.007	.016
**Loneliness** (POMP Score, mean-centered)	.616	.838	.005	.015
**Social support** (POMP Score, mean-centered)	.926	.933	.246	.397
**Advisor relationship** (POMP Score, mean-centered)	.933	.933	.531	.656
**Life satisfaction** (POMP Score, mean-centered)	.636	.838	.055	.096
**Job satisfaction** (POMP Score, mean-centered)	.295	.614	-	-
**COVID-19**	.686	.838	.002	.010
**Year** (linear and quadratic term)	.595	.838	.005	.015
**Peer review**	.073	.201	.588	.686
**Translated records**	.307	.614	.499	.655
**Overall study quality**	.001	.005	.006	.016
**Sampling strategy**	.001	.005	.001	.007
**Assessment strategy**	.895	.933	.043	.082

Note. FDR = false-discovery rate; POMP = percent of maximum possible; BH = Benjamini-Hochberg; ECR status = early-career researcher status (pre- or post-doctoral); Outcome = type of syndrome or symptom assessed; Scale = scale used to assess the syndrome or symptom.

Supplementary Table 20. Information on the threshold, psychometrically validated or defined per common practice, and score method per scale used in the included studies measuring depression

		Score Method	None-Minimal	Mild	Moderate	Moderately-Severe	Severe
**PHQ-9**<br>*2 weeks*	• 9-item version<br>• Responses are summed up for a total score.<br>• Higher scores indicate more severe depressive symptoms.	0-3	0-4	5-9	10-14	15-19	≥ 20
**PHQ-8**<br>*2 weeks*	• 8-item version<br>• Responses are summed up for a total score.<br>• Higher scores indicate more severe depressive symptoms.	0-3	0-4	5-9	10-14	15-19	≥ 20
**PHQ-2**<br>*2 weeks*	• 2-item version<br>• Responses are summed up for a total score.<br>• Higher scores indicate more severe depressive symptoms.	0-3	NA		≥ 3		
			**Normal**	**Mild**	**Moderate**	**Severe**	**Extremely Severe**
**DASS-42**<br>*Past week*	• 42-item version, 14 per subscale<br>• Responses are summed up for a total score.<br>• Higher scores indicate more severe symptoms.	0-3	0-9	10-13	14-20	21-27	≥ 28
**DASS-21**<br>*Past week*	• 21-item version, 7 per subscale<br>• Responses are summed up for a total score.<br>• Final item score must be multiplied by two, to ensure comparability with the DASS-42.<br>• Higher scores indicate more severe symptoms.	0-3	0-4	5-6	7-10	11-13	≥ 14
			**None**	**Minimal**	**Mild**	**Moderate**	**Severe**
**BDI-II-21**<br>*2 weeks*	• 21-item version<br>• Higher scores indicate more severe depressive symptoms.	0-3	0-8	9-13	14-19	20-28	≥ 29
**BDI-FS**<br>*2 weeks*	• 7-item version<br>• Responses are summed up for a total score.<br>• Higher scores indicate more severe depressive symptoms.	0-3				≥ 4	
			**Normal**		**Mild**	**Moderate**	**Severe**
**SDS**<br>*past several days*	• 20-item version<br>• Responses are summed up for a total score.<br>• Higher scores indicate more severe depressive symptoms.	1-4	25-49		50-59	60-69	≥ 70
			**Normal**		**Borderline Abnormal**		**Abnormal**
**HADS-D**<br>*Past week*	• 7-item subscale of the 14-item HADS<br>• Responses are summed up for a total score.<br>• Higher scores indicate more severe depressive symptoms.	0-3	0-7		8-10		≥ 21
			**General Depression**			**Severe Depression**	
**CESD/**<br>**CES-D**<br>*Past week*	• 20-item version<br>• Items 1-3, 5-7, 13-15, 17-20 are scored ordinarily<br>• Items 4, 8, 12, and 16 are reverse scored<br>• Responses are summed up for a total score.	0-3				≥ 16	

	• Higher scores indicate more severe depressive symptoms.				
**CESD-R/ CES-D-R** *Past week*	• 20-item version<br>• Items 4, 8, 12, and 16 are reverse scored<br>• Responses are summed up for a total score.<br>• CESD-R can be recoded into the CESD from 1-5 to 0-3.<br>• Higher scores indicate more severe depressive symptoms.	0-4			≥ 29
**CESD-10**	• Higher scores indicate more severe depressive symptoms.	0-3			≥ 10
**CESD-8**	• Higher scores indicate more severe depressive symptoms.	0-3			≥ 7
**GHQ-28**	• a 28-item scaled version – assesses somatic symptoms, anxiety and insomnia, social dysfunction and severe depression.<br>• Higher scores indicate more severe depressive symptoms.	0-3<br>0-0-1-1			NA<br>≥ 5
			**Mild Depression**		**Probable Clinical Depression**
**WEMWBS** *2 weeks*	• 14-item version<br>• Responses are summed up for a total score.<br>• WEMWBS has been bench marked on **CES-D**.<br>• Using CES-D =26 and CES-D = 16 as cut points.<br>• Higher scores indicate better well-being.	1-5	41-44		≤ 41
**SWEMWBS** *2 weeks*	• Raw scores need to be transformed.<br>• 7-item version<br>• SWEMWBS has been benchmarked on PHQ-9 and GAD-7.<br>• Using the cut points of PHQ-9 = 5 and PHQ-9 =10.<br>• Higher scores indicate better well-being.	1-5	18-20		≤ 18
**MHI-D**	• 18-item version<br>• The MHI has 4 subscales (Anxiety, Depression, Behavioral Control, and Positive Affect) and one total score<br>• Higher scores indicate better mental health.	0-100	NA		
**SASS-D**	• 34-item measure<br>• Measure both an individual's wellbeing/assets as well as their overall psychological distress, including issues with depression, anxiety, substance use, eating problems, and negative affect.	0-3<br>(0-15)	NA		
**BSI**	• 6-item Skala<br>• Transformation to T-values<br>• Higher scores indicate more distress and psychopathology.	0-3			≥ 63 T-Wert

1. Anderson, C., Laubscher, S. & Burns, R. Validation of the short form 36 (SF-36) Health Survey Questionnaire among stroke patients. *Stroke* **27**, 1812–1816 (1996).

2. Bean, C. A. L. et al. Improved scoring of the Center for Epidemiologic Studies Depression Scale – revised: An item response theory analysis. *J Psychopathol Behav Assess* **46**, 783–792 (2024).

3. Beck, A. T. An inventory for measuring depression. *Arch Gen Psychiatry* **4**, 561 (1961).

4. Beck, A. T., Steer, R. A., Ball, R. & Ranieri, W. F. Comparison of Beck Depression Inventories-IA and-II in psychiatric outpatients. *Journal of Personality Assessment* **67**, 588–597 (1996).

5. Bianco, D. Performance of the Warwick-Edinburgh Mental Well-Being Scale (WEMWBS) as a screening tool for depression in UK and Italy. (UNIVERSITÀ DI BOLOGNA, Bologna, 2011).

6. Brehaut, E. et al. Depression prevalence using the HADS-D compared to SCID major depression classification: An individual participant data meta-analysis. *Journal of Psychosomatic Research* **139**, 110256 (2020).

7. Derogatis, L. R. & Melisaratos, N. The Brief Symptom Inventory: an introductory report. *Psychol. Med.* **13**, 595–605 (1983).

8. Downs, A., Boucher, L. A., Campbell, D. G. & Dasse, M. Development and initial validation of the Symptoms and Assets Screening Scale. *Journal of American College Health* **61**, 164–174 (2013).

9. Downs, A., Boucher, L. A., Campbell, D. G. & Dasse, M. *Symptoms and Assets Screening Scale*. https://doi.org/10.1037/t42564-000 (2015).

10. Dunstan, D. A. & Scott, N. Clarification of the cut-off score for Zung's self-rating depression scale. *BMC Psychiatry* **19**, 177 (2019).

11. Edelstein, B. A., Drozdick, L. W. & Ciliberti, C. M. Assessment of depression and bereavement in older adults. In: Handbook of Assessment in Clinical Gerontology 3–43. doi:10.1016/B978-0-12-374961-1.10001-6 (2010).

12. Fu, H., Si, L. & Guo, R. What Is the optimal cut-off point of the 10-Item Center for Epidemiologic Studies Depression Scale for Screening Depression among Chinese individuals aged 45 and over? An exploration using latent profile analysis. *Front. Psychiatry* **13**, 820777 (2022).

13. Goldberg, D. P. & Hillier, V. F. A scaled version of the General Health Questionnaire. *Psychol. Med.* **9**, 139–145 (1979).

14. Kliem, S., Beller, J., Nanette Tubibos, A. & Brähler, E. Normierung und Evaluation der Messinvarianz der 8-Item-Kurzform der Center of Epidemiological Studies-Depression Scale (CES-D-8). *Zeitschrift für Psychosomatische Medizin und Psychotherapie* **66**, 259–271 (2020).

15. Kroenke, K., Spitzer, R. L. & Williams, J. B. W. The PHQ-9: Validity of a brief depression severity measure. *Journal of General Internal Medicine* **16**, 606 613 (2001).

16. Kroenke, K., Spitzer, R. L. & Williams, J. B. W. The Patient Health Questionnaire-2: Validity of a Two-Item Depression Screener. *Medical Care* **41**, 1284-1292 (2003).

17. Lovibond, S. H. & Lovibond, P. F. *Manual for the Depression Anxiety Stress Scales.* (Psychology Foundation, Sydney, 1995).

18. Radloff, L. S. The CES-D scale: A self-report depression scale for research in the general population. *Applied Psychological Measurement* **1**, 385–401 (1977).

19. Shah, N., Cader, M., Andrews, B., McCabe, R. & Stewart-Brown, S. L. Short Warwick-Edinburgh Mental Well-being Scale (SWEMWBS): performance in a clinical sample in relation to PHQ-9 and GAD-7. *Health Qual Life Outcomes* **19**, 260 (2021).

20. Veit, C. T. & Ware, J. E. *Mental Health Inventory*. https://doi.org/10.1037/t02354-000 (2011).

21. Zigmond, A. S. & Snaith, R. P. The Hospital Anxiety and Depression Scale. *Acta Psychiatr. Scand.* **67**, 361–370 (1983).

Supplementary Table 21. Information on the threshold, psychometrically validated or defined per common practice, and score method per scale used in the included studies measuring general anxiety disorder

		**Score Method**	**Minimal**	**Mild**	**Moderate**	**Severe**	
**GAD-7**<br>*2 weeks*	• Responses are summed up for a total score.<br>• Higher scores indicate more severe anxiety symptoms.	0-3	0-4	5-9	10-14	≥ 15	
**GAD-2**	• Two-item version	0-3	NA		≥ 3		
			**Low**		**Moderate**	**Potentially concerning levels of anxiety**	
**BAI-21**<br>*Past week*	• Responses are summed up for a total score.<br>• Higher scores indicate more severe anxiety symptoms.	0-3	0-21		22-35	≥ 36	
			**Normal**	**Mild**	**Moderate**	**Severe**	**Extremely-Severe**
**DASS-21**<br>*Past week*	• Responses are summed up for a total score.<br>• Final item score must be multiplied by two, to ensure comparability with the DASS-42.<br>• Higher scores indicate more severe symptoms.	0-3	0-3	4-5	6-7	8-9	≥ 10
**DASS-42**	• Responses are summed up for a total score.<br>• Higher scores indicate more severe symptoms.	0-3	0-7	8-9	10-14	15-19	≥ 20
			**Normal**	**Borderline Abnormal**		**Abnormal**	
**HADS-A**<br>*Past week*	• 7-item subscale of the 14-item HADS<br>• Responses are summed up for a total score.<br>• Higher scores indicate more severe anxiety symptoms.	0-3	0-7	8-10		11 – 21	
**MHI-A**	• 18-item version<br>• The MHI has 4 subscales (Anxiety, Depression, Behavioral Control, and Positive Affect) and one total score.<br>• Higher scores indicate better mental health.	0-100	NA				
			**No or low anxiety**		**Moderate anxiety**	**High anxiety**	
**STAI**<br>*generally versus in this moment*	• Total scale consists of 40-items, 20 per subscale (trait | state)<br>• Responses are summed up for a total score.	1-4	20-37		38-44	45-80	
			**Normal**	**Mild to Moderate**	**Severe**	**Above extreme**	
**SAS**<br>*past several days*	• 20-item scale<br>• Responses are summed up for a total score.<br>• Higher scores indicate more severe anxiety symptoms.	1-4<br>(20-80)	20-44	45-59	60-74	≥ 75	
**GHQ-28**	• a 28-item scaled version – assesses somatic symptoms, anxiety and insomnia, social dysfunction and severe depression.	0-3<br>0-0-1-1			NA<br>≥ 5		
**SASS-A**	• 34-item measure	0-3<br>(0-15)	NA				

	• Measure both an individual's wellbeing/assets as well as their overall psychological distress, including issues with depression, anxiety, substance use, eating problems, and negative affect.				
**BSI**	• 6-item scale<br>• Transformation to T-values	0-3			≥ 63 T-Wert

1. Beck, A. T., Epstein, N., Brown, G. & Steer, R. A. An inventory for measuring clinical anxiety: Psychometric properties. *Journal of Consulting and Clinical Psychology* **56**, 893–897 (1988).

2. Derogatis, L. R. & Melisaratos, N. The Brief Symptom Inventory: An introductory report. *Psychol. Med.* **13**, 595–605 (1983).

3. Downs, A., Boucher, L. A., Campbell, D. G. & Dasse, M. Development and initial validation of the Symptoms and Assets Screening Scale. *Journal of American College Health* **61**, 164–174 (2013).

4. Downs, A., Boucher, L. A., Campbell, D. G. & Dasse, M. *Symptoms and Assets Screening Scale*. https://doi.org/10.1037/t42564-000 (2015).

5. Goldberg, D. P. & Hillier, V. F. A scaled version of the General Health Questionnaire. *Psychol. Med.* **9**, 139–145 (1979).

6. Goldberg, D. P. & Hillier, V. F. A *User's Guide to the General Health Questionnaire.* (Windsor, 1988).

7. Lovibond, S. H. & Lovibond, P. F. *Manual for the Depression Anxiety Stress Scales*. (Psychology Foundation, Sydney, 1995).

8. Spitzer, R. L., Kroenke, K., Williams, J. B. W. & Löwe, B. A brief measure for assessing generalized anxiety disorder: The GAD-7. *Arch Intern Med* **166**, 1092 (2006).

9. Veit, C. T. & Ware, J. E. *Mental Health Inventory*. https://doi.org/10.1037/t02354-000 (2011).

10. Zigmond, A. S. & Snaith, R. P. The Hospital Anxiety and Depression Scale. *Acta Psychiatr. Scand.* **67**, 361–370 (1983).

11. Zung, W. W. K. A rating instrument for anxiety disorders. *Psychosomatics* **12**, 371–379 (1971).

Supplementary Table 22. Information on the threshold, psychometrically validated or defined per common practice, and score method per scale used in the included studies measuring alcohol and drug abuse

<table>
<tr><th colspan="2"></th><th>Score Method</th><th>ZONE I</th><th colspan="2">ZONE II
Hazardous Alcohol Use</th><th>ZONE III
Likely Alcohol Dependence</th><th colspan="2">ZONE IV</th></tr>
<tr><td>AUDIT</td><td>• 10-item version</td><td>0-4</td><td>0-7</td><td colspan="2">8-15</td><td>16-19</td><td colspan="2">≥ 20</td></tr>
<tr><td>AUDIT-C</td><td>• Short-version of the AUDIT, 3-item-version
• Responses are summed up for a total score.</td><td>0-4</td><td></td><td colspan="5">≥ 4 men
≥ 3 women</td></tr>
<tr><td colspan="2"></td><td></td><td>Low Level</td><td colspan="3">Moderate Level</td><td>Substantial level</td><td>Severe Level</td></tr>
<tr><td>DAST
Past year</td><td>• 10-item version</td><td>0-1</td><td>1-2</td><td colspan="3">3-5</td><td>6-8</td><td>9-10</td></tr>
<tr><td colspan="2"></td><td></td><td colspan="2"></td><td colspan="2"></td><td colspan="2"></td></tr>
<tr><td>SASS-SA</td><td>• 34-item measure
• Measure both an individual's wellbeing/assets as well as their overall psychological distress, including issues with depression, anxiety, substance use, eating problems, and negative affect.</td><td>0-3
(0-15)</td><td colspan="6">NA</td></tr>
<tr><td colspan="2"></td><td></td><td colspan="2">Mild</td><td colspan="2">Moderate</td><td colspan="2">Severe</td></tr>
<tr><td>SCID-5-RV</td><td></td><td></td><td colspan="2">2-3 symptoms</td><td colspan="2">4-5 symptoms</td><td colspan="2">6+ symptoms</td></tr>
</table>

1. Babor, T. F., Higgins, J. C., Saunders, J. B. & Monteiro, M. G. *AUDIT. The Alcohol Use Disorders Identification Test Guidelines for Use in Primary Care.* (WHO, 2001).

2. Bush, K. The AUDIT Alcohol Consumption Questions (AUDIT-C). An Effective Brief Screening Test for Problem Drinking. *Arch Intern Med* **158**, 1789 (1998).

3. Downs, A., Boucher, L. A., Campbell, D. G. & Dasse, M. Development and initial validation of the Symptoms and Assets Screening Scale. *Journal of American College Health* **61**, 164–174 (2013).

4. Downs, A., Boucher, L. A., Campbell, D. G. & Dasse, M. *Symptoms and Assets Screening Scale*. https://doi.org/10.1037/t42564-000 (2015).

5. First, M. B., Williams, J. B. W., Karg, R. S. & Spitzer, R. L. *User's Guide for the Structured Clinical Interview for DSM5 Disorders, Research Version*. (American Psychiatric Association, Washington, 2015).

6. Gavin, D. R., Ross, H. E. & Skinner, H. A. Diagnostic validity of the Drug Abuse Screening Test in the assessment of DSM-III drug disorders. *British Journal of Addiction* **84**, 301–307 (1989).

Supplementary Table 23. Information on the threshold, psychometrically validated or defined per common practice, and score method per scale used in the included studies measuring suicidal ideation

		Score Method		Suicidal Ideation	
**PHQ-9**<br>*2 weeks*	• Item 9, the final item of PHQ-9, is generally used in research to determine the existence of suicidal ideation.<br>• Higher scores reflect higher presence of suicidal ideation.	0-3	0	≥ 1	
				**Suicidal Thoughts**	**Active Ideation**
**BDI-II**	• Item 9<br>• Higher scores reflect higher presence of suicidal ideation.	0-3	0	≥ 1	≥ 2
**CESD-R-20**<br>*past week*	• Item 14 and 15 measure suicidal ideation.<br>• Higher scores reflect higher presence of suicidal ideation.	0-4	-	≥ 1	
				**At Risk**	
**SBQ-R**<br>*lifetime, last year*	• Items measure 1) Lifetime suicide ideation and attempts; 2) Frequency of suicidal ideation over the past 12 months; 3) Threat of suicide attempt; 4) Self-reported likelihood of future suicidal behavior.<br>• Higher scores reflect higher presence of suicidal ideation.<br>• Each item has its own scoring rubric, with total scores ranging from 3 to 18.	0-6<br>1-3/4/5	-	≥ 7	

1. Babor, T. F., Higgins, J. C., Saunders, J. B. & Monteiro, M. G. *AUDIT. The Alcohol Use Disorders Identification Test Guidelines for Use in Primary Care*. (WHO, 2001).

2. Bush, K. The AUDIT Alcohol Consumption Questions (AUDIT-C). An Effective Brief Screening Test for Problem Drinking. *Arch Intern Med* **158**, 1789 (1998).

3. Downs, A., Boucher, L. A., Campbell, D. G. & Dasse, M. Development and initial validation of the Symptoms and Assets Screening Scale. *Journal of American College Health* **61**, 164–174 (2013).

4. Downs, A., Boucher, L. A., Campbell, D. G. & Dasse, M. *Symptoms and Assets Screening Scale*. https://doi.org/10.1037/t42564-000 (2015).

5. First, M. B., Williams, J. B. W., Karg, R. S. & Spitzer, R. L. *User's Guide for the Structured Clinical Interview for DSM5 Disorders, Research Version.* (American Psychiatric Association, Washington, 2015).

6. Gavin, D. R., Ross, H. E. & Skinner, H. A. Diagnostic validity of the Drug Abuse Screening Test in the assessment of DSM-III drug disorders. *British Journal of Addiction* **84**, 301–307 (1989).

Supplementary Table 24. Information on the threshold, psychometrically validated or defined per common practice, and score method per scale used in the included studies measuring sleep quality

		Score Method		Poor Sleep Quality
**JSS-4**<br>*past month*	• 4-item questionnaire<br>• The higher the score, the more severe the sleep disturbances.	0-5	NA	
**PSQI**<br>*past month*	• 18-item questionnaire<br>• Responses are summed up for a total score.<br>• The higher the score, the more severe the sleep disturbances.	0-3<br>(0-21)		> 5

1. Buysse, D. J., Reynolds, C. F., Monk, T. H., Berman, S. R. & Kupfer, D. J. The Pittsburgh Sleep Quality Index: A new instrument for psychiatric practice and research. *Psychiatry Research* **28**, 193–213 (1989).

2. Jenkins, C. D., Stanton, B. A., Niemcryk, S. J. & Rose, R. M. A scale for the estimation of sleep problems in clinical research. *Journal of Clinical Epidemiology* **41**, 313–321 (1988).

Supplementary Table 25. Information on the score method per scale used in the included studies measuring burnout

		Score Method				
**MBI-ES**<br>MBI-ES-EE<br>MBI-ES-DP<br>MBI-ES-PA	 9 items<br>5 items<br>8 items	0-6	NA			
**MBI-SS**<br>MBI-SS-EE<br>MBI-SS-DP<br>MBI-SS-PA	 5 items<br>4 items<br>6 items	0-6	NA			
**BMS-10**		1-7	N/A			
**MED NORD**	• Only exhaustion<br>• 4-item version	1-5	NA			
**CBI-S**	• This scale consists of 25 items that represent 4 subscales<br>• The answers are quantified as 100, 75, 50, 25, and 0% respectively, with a reverse scoring for item 10	0-100	0-49	50-74	75-99	≥ 100

1. Lonka, K. et al. MED NORD–A tool for measuring medical students' well-being and study orientations. *Medical Teacher* **30**, 72–79 (2008).

2. Lourel, M., Gueguen, N. & Mouda, F. L'évaluation du burnout de Pines: adaptation et validation en version française de l'instrument Burnout Measure Short version (BMS-10). *Pratiques Psychologiques* **13**, 353–364 (2007).

3. Maslach, C. & Jackson, S. E. The measurement of experienced burnout. *J. Organ. Behavior* **2**, 99–113 (1981).

4. Maslach, C., Leiter, M. P. & Jackson, S. E. *Maslach Burnout Inventory Manual*. (Mind Garden, 2018).

Supplementary Table 26. Information on the score method per scale used in the included studies measuring stress

		**Score Method**	**Low**		**Moderate**		**High**
**PSS-14**<br>-	• Responses are summed up for a total score.<br>• Higher scores indicate higher levels of stress.	0-4	0-18		19-37		38-56
**PSS-10**<br>-	• Responses are summed up for a total score.<br>• Higher scores indicate higher levels of stress.	0-4	0-13		14-26		27-40
**PSS-4**<br>-	• Responses are summed up for a total score.<br>• Higher scores indicate higher levels of stress.	0-4	NA				
		**Score Method**	Normal	Mild	Moderate	Severe	Extremely Severe
**DASS-42**<br>*Past week*	• Higher scores indicate higher levels of stress.	0-3	0-14	15-18	19-25	26-33	≥ 34
**DASS-21**<br>*Past week*	• Higher scores indicate higher levels of stress.	0-3	0-7	8-9	10-12	13-16	≥ 17

1. Cohen, S., Kamarck, T. & Mermelstein, R. A global measure of perceived stress. *Journal of Health and Social Behavior* **24**, 385 (1983).

2. Lovibond, S. H. & Lovibond, P. F. *Manual for the Depression Anxiety Stress Scales*. (Psychology Foundation, Sydney, 1995).

Supplementary Table 27. Information on the score method per scale used in the included studies measuring mental health

		Score Method	Well	Mild Disorder	Moderate Disorder	Severe Disorder
**K-6**<br>*past month*	• Responses are summed up for a total score.<br>• Higher scores suggest more distress and worse psychological well-being.	0-4			≥ 13	
**K-10**<br>*past month*		0-4<br>1-5	10-19	20-24	25-29	≥ 30
**GHQ-28**	• Higher scores suggest more distress and worse psychological well-being.			Of any subscale ≥ 5		
**GHQ-12**				GHQ2+	GHQ3+	GHQ4+
**WEMWBS**	• 14-item version<br>• Responses are summed up for a total score.<br>• Higher scores indicate better well-being but were reverse-scored here to determine psychological distress as the opposite of mental health.	1-5	NA			
**SWEMWBS**	• Raw scores need to be transformed.<br>• 7-item version<br>• Higher scores indicate better well-being but were reverse-scored here to determine psychological distress as the opposite of mental health.	1-5	NA			
						Poor mental wellbeing
**WHO-5 Index**	• The raw score is calculated by totaling the scores on each of the five questions. To get a percentage score ranging from zero to 100, the raw score is multiplied by four.<br>• Higher scores indicate better well-being but were reverse-scored here to determine psychological distress as the opposite of mental health.	0-5				≤ 13 (raw scores)<br>≤ 50 (transformed scores)
**SASS-MH**	• 34-item measure<br>• Measure both an individual's wellbeing/assets as well as their overall psychological distress, including issues with depression, anxiety, substance use, eating problems, and negative affect.	0-3<br>(0-69)	NA			

1. Bianco, D. Performance of the Warwick-Edinburgh Mental Well-Being Scale (WEMWBS) as a screening tool for depression in UK and Italy. (UNIVERSITÀ DI BOLOGNA, Bologna, 2011).

2. Downs, A., Boucher, L. A., Campbell, D. G. & Dasse, M. Development and initial validation of the Symptoms and Assets Screening Scale. *Journal of American College Health* **61**, 164–174 (2013).

3. Downs, A., Boucher, L. A., Campbell, D. G. & Dasse, M. *Symptoms and Assets Screening Scale*. https://doi.org/10.1037/t42564-000 (2015).

4. Goldberg, D. P. & Hillier, V. F. A scaled version of the General Health Questionnaire. *Psychol. Med.* **9**, 139–145 (1979).

5. Goldberg, D. P. & Hillier, V. F. *A User's Guide to the General Health Questionnaire*. (Windsor, 1988).

6. Kessler, R. C. *et al.* Screening for serious mental illness in the general population. *Arch. Gen. Psychiatry* **60**, 184 (2003).

7. Shah, N., Cader, M., Andrews, B., McCabe, R. & Stewart-Brown, S. L. Short Warwick-Edinburgh Mental Well-being Scale (SWEMWBS): performance in a clinical sample in relation to PHQ-9 and GAD-7. *Health Qual Life Outcomes* **19**, 260 (2021).

8. World Health Organization. *The World Health Organization-Five Well-Being Index (WHO-5)*. (2024).

Supplementary Table 28. Information on the threshold, psychometrically validated or defined per common practice, and score method per scale used in the included studies measuring somatic symptomology

		Score Method		
**GHQ-28**		0-3<br>0-0-1-1		≥ 5

1. Goldberg, D. P. & Hillier, V. F. A scaled version of the General Health Questionnaire. *Psychol. Med.* **9**, 139–145 (1979).

2. Goldberg, D. P. & Hillier, V. F. A *User's Guide to the General Health Questionnaire*. (Windsor, 1988).

Supplementary Table 29. Information on the threshold, psychometrically validated or defined per common practice, and score method per scale used in the included studies measuring disordered eating

		Score Method	No Eating Disorder	Eating Disorder
**SCOFF-5**		0-1		≥ 2
**SCID-5-RV**				
**ESP**		0-1		≥ 2
**SASS**				
**SDE**		0-1		≥ 2

1. Botella, J., Sepúlveda, A. R., Huang, H. & Gambara, H. A Meta-analysis of the diagnostic accuracy of the SCOFF. *Span. J. Psychol.* **16**, E92 (2013).

2. First, M. B., Williams, J. B. W., Karg, R. S. & Spitzer, R. L. *User's Guide for the structured clinical interview for DSM5 disorders, research version*. (American Psychiatric Association, Washington, 2015).

3. Cotton, M.-A., Ball, C. & Robinson, P. Four simple questions can help screen for eating disorders. *J Gen Intern Med* **18**, 53–56 (2003).

4. Downs, A., Boucher, L. A., Campbell, D. G. & Dasse, M. Symptoms and assets screening scale. https://doi.org/10.1037/t42564-000 (2015).

5. Maguen, S. *et al.* Screen for disordered eating: Improving the accuracy of eating disorder screening in primary care. *General Hospital Psychiatry* **50**, 20–25 (2018).

Supplementary Table 30. Back-transformed POMP scores of the severity estimates

Instrument	$k_{records}$	#ES	*n*	*M**	LL CI 95 %	UL CI 95 %	Mean level of severity
**Substance Abuse**							
AUDIT	7	13	33.882	4.14	1.13	5.16	Zone I (normal use)
AUDIT-C	5	8	14.835	2.40	1.68	3.13	Not above threshold (≥ 3 women, ≥ 4 men)
DAST	2	3	1.182	0.42	-0.51	1.35	Not above threshold (≥ 1)
**Depression**							
PHQ-9	44	67	72.239	6.99	6.12	7.86	Mild
PHQ-8	6	7	3.518	8.05	6.12	9.98	Mild
PHQ-2	5	6	3.882	1.73	1.19	2.27	Not above threshold (≥ 3)
CESD-20	5	5	8.120	18.53	12.40	24.65	Severe depression
CESD-10	3	3	4.751	10.66	7.31	14.01	Severe depression
CESD-8	1	25	4.812	8.95	3.70	14.21	Severe depression
DASS-D	23	27	5.184	13.68	11.69	15.66	Moderate
HADS-D	2	3	273	6.16	2.49	9.82	Normal
SDS	1	1	285	42.93	27.58	56.27	Normal
BDI-FS	1	1	124	2.11	-1.43	5.64	Not above threshold (≥ 4)
**General Anxiety Disorder**							
GAD-7	44	60	69.850	6.33	5.64	7.02	Mild
GAD-2	5	7	3.391	2.33	1.82	2.84	Not above threshold (≥ 3)
STAI-T	3	3	835	46.64	39.97	53.31	Higher scores indicate worse anxiety (20-80)
STAI-S	3	4	793	53.74	47.73	59.75	Higher scores indicate worse anxiety (20-80)
DASS-A	21	25	4.621	9.33	7.32	11.34	Mild/ moderate
BAI-21	2	2	209	20.77	12.53	29.02	Low
HADS-A	2	3	273	9.24	5.56	12.91	Borderline abnormal
SAS	1	1	285	37.16	21.84	52.49	Normal
**Eating Disorder**							
SCOFF-5	13	21	52.365	0.77	0.56	0.99	Not above threshold (≥ 2)
SDE	2	4	4.654	1.64	1.22	2.06	Not above threshold (≥ 2)
**Mental Health**							
K-6	2	2	4.474	6.57	3.03	10.11	Not above threshold (≥ 13)
K-10	3	4	1.115	16.07	13.15	20.98	Well
WHO-5 Index	2	3	534	11.15	8.05	14.26	Higher scores indicate worse wellbeing (reverse scored)
WEMWBS	4	6	1.359	39.30	34.97	43.64	Higher scores indicate worse wellbeing (reverse scored)
SWEMWBS	3	3	4.787	21.73	18.28	25.17	Higher scores indicate worse wellbeing (reverse scored)
**Sleep Quality**							
PSQI	2	3	245	5.43	2.33	8.54	Significant sleep difficulties
**Suicidal Ideation**							
PHQ-9, item 9	25	42	63.756	0.21	0.11	0.32	Not above threshold (≥ 1)
**Stress**							
DASS-S	21	25	4.041	17.67	15.65	19.69	Mild/ moderate
PSS-14	7	9	965	32.93	28.76	37.10	Moderate
PSS-10	13	14	6.472	20.06	17.80	22.32	Moderate
PSS-4	7	8	3919	7.18	5.89	8.46	Higher scores indicate higher stress
**Burnout**							
MBI-ES	5	5	716	51.36	39.39	63.33	Higher scores indicate higher burnout level (0-132)
MBI-ES-EE	6	6	932	28.67	23.93	33.41	Higher scores indicate higher emotional exhaustion (0-54)
MBI-ES-DP	5	5	716	14.49	11.38	17.99	Higher scores indicate higher depersonalization (0-36)
MBI-ES-PA	5	5	716	30.22	25.80	34.64	Higher scores indicate higher inefficacy (0-48)

Note. This table does not include instruments for which *n* < 2. * = calculated via back-transforming the POMP score according to the respective scale's minimum and maximum scores: Back-transformed score = (POMP/100) * (max – min) + min; $k_{records}$ = number of studies that used this instrument; *n* = number of effect sizes for this instrument; CI = confidence interval; LL = lower limit; UL = upper limit; AUDIT = Alcohol use disorder identification test; AUDIT-C = Alcohol use disorder

identification test – consumption; DAST = Drug abuse screening test; PHQ-9 = Patient health questionnaire, 9 items; PHQ-8 = Patient health questionnaire, 8 items; PHQ-2 = Patient health questionnaire, 2 items; CESD-20 = Center for epidemiological disease, 20 items; CESD-10 = Center for epidemiological disease, 10 items; CESD-8 = Center for epidemiological disease, 8 items; DASS-D = Depression, anxiety and stress scale, depression subscale, 42-item version; HADS-D = Hospital anxiety and depression scale, depression subscale; SDS = Zung self-rating depression scale; BDI-FS = Beck depression inventory, fast screen; GAD-7 = Generalized anxiety disorder, 7 items; GAD-2 = Generalized anxiety disorder, 2 items; STAI-T = State-trait-anxiety inventory, trait subscale; STAI-S = State-trait-anxiety inventory, state subscale; DASS-A = Depression, anxiety and stress scale, anxiety subscale, 42-item version; BAI-21 = Beck anxiety inventory, 21 items; HADS-A = Hospital anxiety and depression scale, anxiety subscale; SAS = Zung self-rating anxiety scale; SCOFF-5 = Sick-control-one stone-fat-food, 5 items; K-6 = Kessler psychological distress scale, 6 items; K-10 = Kessler psychological distress scale, 10 items; WHO-5 Index = WHO wellbeing index, 5 items; WEMWBS = Warwick Edinburgh mental wellbeing scale; SWEMWBS = Short Warwick Edinburgh mental wellbeing scale; PSQI = Pittsburgh sleep quality index; DASS-S = Depression, anxiety, and stress scale, stress subscale, 42-item version; PSS-14 = Perceived stress scale, 14 items; PSS-10 = Perceived stress scale, 10 items; PSS-4 = Perceived stress scale, 4 items; MBI-ES = Maslach burnout inventory – educator survey; MBI-ES-EE = Maslach burnout inventory – educator survey, emotional exhaustion subscale; MBI-ES-DP = Maslach burnout inventory – educator survey, depersonalization subscale; MBI-ES-PA = Maslach burnout inventory – educator survey, personal accomplishment subscale

Supplementary Table 31. Severity estimates per scale and severity categorization

						95 % CI		
**Syndrome**	**Scale**	***k***	***#ES***	***n***	**β**	**LL**	**UL**	**Comparison**
**Depression**	**PHQ-9/ PHQ-8**	35/ 3	218/ 11	69,741/ 2,282				**Guo et al. (2021)** (solely PhD)
	None				(36.96)			
	Mild				33.12	30.80	35.47	Mild: 36%
	Moderate				17.03	15.22	18.93	Moderate: 12%
	Moderately-severe				8.13	6.83	9.54	
	Severe				4.76	3.76	5.87	Severe: 9%
	**DASS**	14	68	3,135				
	Normal				(51.96)			
	Mild				12.14	9.16	15.47	
	Moderate				18.14	14.56	22.02	
	Severe				8.06	5.62	10.89	
	Extremely-severe				9.70	7.02	12.75	
**GAD**	**GAD-7**	29	126	63,725				**Chi et al. (2023)**
	None				(43.40)			(solely PhD)
	Mild				30.89	28.44	33.41	Mild: 22.9%,
	Moderate				16.00	14.08	18.02	Moderate: 16.5%,
	Severe				9.71	8.17	11.35	Severe: 13.5%
	**DASS**	13	64	2,624				
	Normal				(59.63)			
	Mild				7.14	4.77	9.94	
	Moderate				16.32	12.78	20.20	
	Severe				6.83	4.52	9.59	
	Extremely-severe				10.08	7.26	13.31	
**Stress**	**DASS**	13	61	2,624				
	Normal				(58.48)			
	Mild				11.68	8.52	15.26	
	Moderate				13.82	10.40	17.64	
	Severe				11.96	8.89	15.41	
	Extremely-severe				4.06	2.24	6.38	

Chi, T., Cheng, L., & Zhang, Z. Global prevalence and trend of anxiety among graduate students: A systematic review and meta-analysis. *Brain and Behavior* **13**(4), e2909. https://doi.org/10.1002/brb3.2909 (2023).

Guo, L., Fan, H., Xu, Z., Li, J., Chen, T., Zhang, Z., & Yang, K. Prevalence and changes in depressive symptoms among postgraduate students: A systematic review and meta-analysis from 1980 to 2020. *Stress and Health* **37**(5), 835–847. https://doi.org/10.1002/smi.3045 (2021).

Supplementary Table 32. Mean (*SD*) of age and risk and protective factors per moderator and model

	**POMP scores**				**Prevalence**			
**Moderator**	k_{study}	***n***	**#ES**	***M*(*SD*)**	k_{study}	***n***	**#ES**	***M*(*SD*)**
**Sample Characteristics**								
Age (mean)	100	64,374	398	30.61 (4.51)	61	90,635	297	29.67 (4.22)
**Risk and Protective Factors**								
Job Satisfaction (POMP score)	3	1,175	8	61.94 (7.04)	1	589	1	58.83 (-)
Life Satisfaction (POMP score)	13	5,798	28	58.02 (15.47)	6	3,534	12	57.98 (11.73)
Advisor Relationship (POMP score)	11	7,625	37	63.34 (17.36)	7	7,208	18	62.99 (22.08)
Social Support (POMP score)	9	1,431	22	68.45 (13.63)	3	405	4	74.27 (10.40)
Loneliness (POMP score)	11	18,164	64	39.94 (10.39)	11	36,711	78	40.07 (8.55)
History of Mental Disorders (% with diagnosis)	19	23,984	112	37.54% (14.06%)	18	23,272	135	29.58% (12.56%)

Note. Due to the high heterogeneity among the instruments and scales used to assess job and life satisfaction, advisor relationship, social support, and loneliness, these were transformed into POMP scores. History of mental disorders was given in percent among the whole sample. Thus, the higher the scores on the protective factors, namely job satisfaction, life satisfaction, advisor relationship, and social support, the higher the satisfaction, perceived support provided or better the relationship quality. The higher, though, the scores on the other risk factors, namely loneliness and history of mental disorders, the greater the experienced feeling of loneliness and higher prevalence of already existing mental health diagnosis prior to the study. Summary scores could differ between the two models, as not all studies were included in both models. POMP = percent of maximum possible; *k* = number of samples; *n* = cumulative sample size; #ES = number of effect sizes; *M* = mean; *SD* = Standard deviation.

Supplementary Figure 13. Graphical display of psychological distress for pooled severity over the years

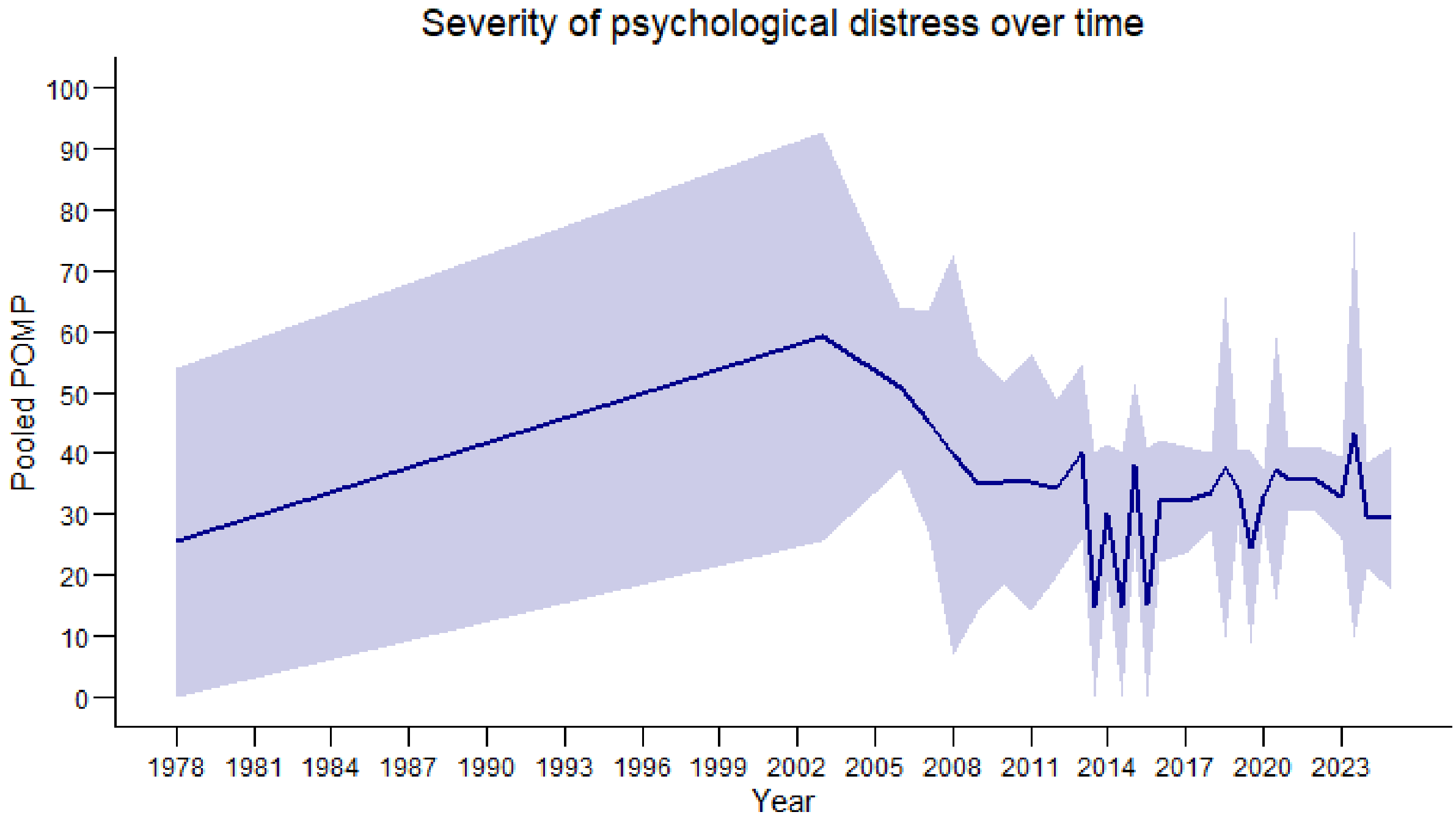

Note. This line graph illustrates the pooled severity of psychological distress expressed as POMP (percentage of maximum possible) scores from 1978/2007 to 2025. The dark blue line represents the annual pooled severity estimate and the shaded region is the 95% confidence band. The confidence band is wider from 1978 to 2003, because there were no studies available for the 25 years between the oldest study from 1978 and the next oldest study from 2003. There are also wider confidence bands in later years, indicating greater uncertainty around the pooled point estimates. POMP = percent of maximum possible scores, ranging from 0 to 100.

Supplementary Figure 14. Funnel plot for the prevalence model by study effect

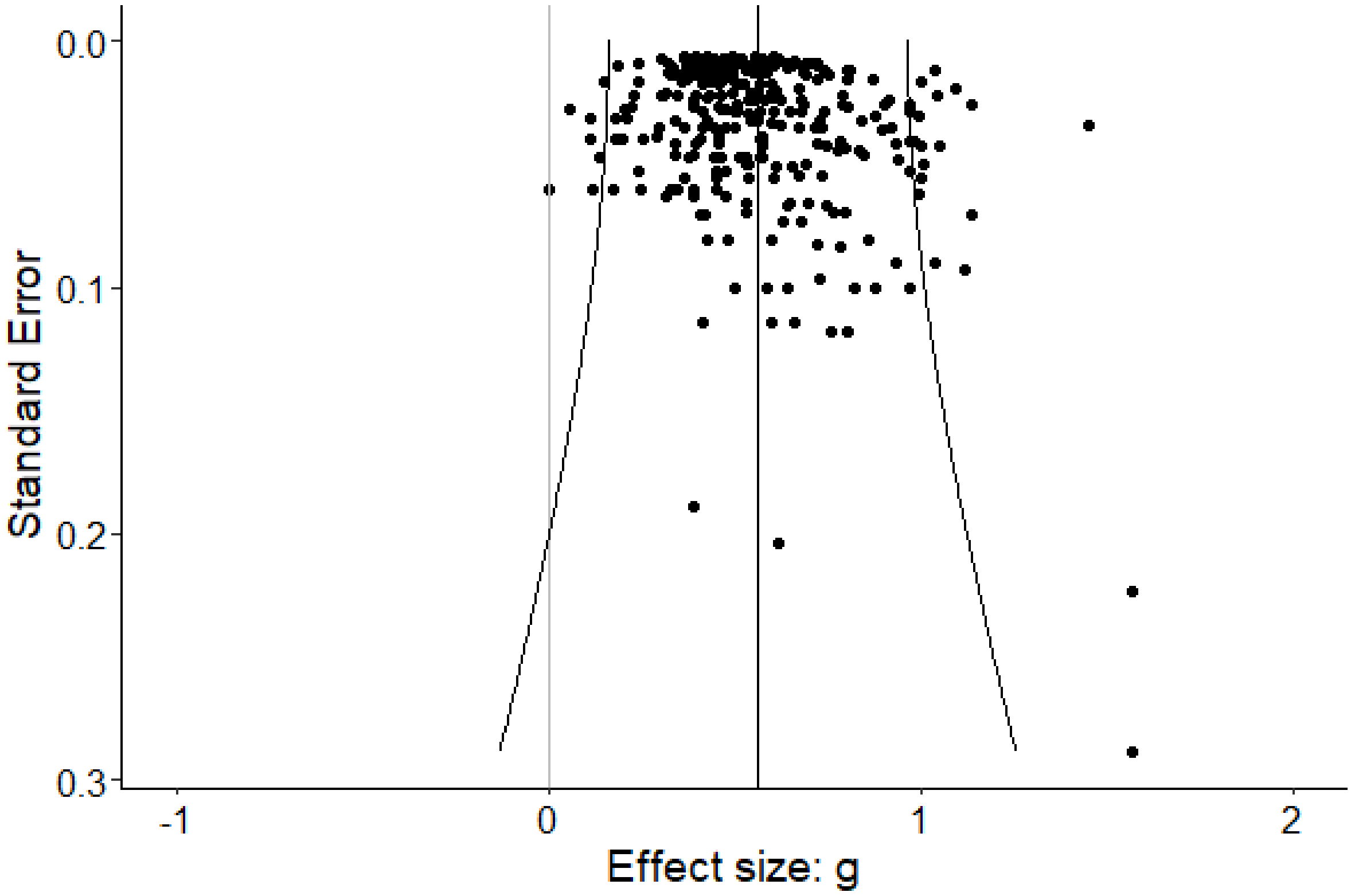

Note. Pooled effect sizes can be back-transformed into prevalence using [sin(effect size)]².

Supplementary Figure 15. Funnel plot for the POMP-score model per effect

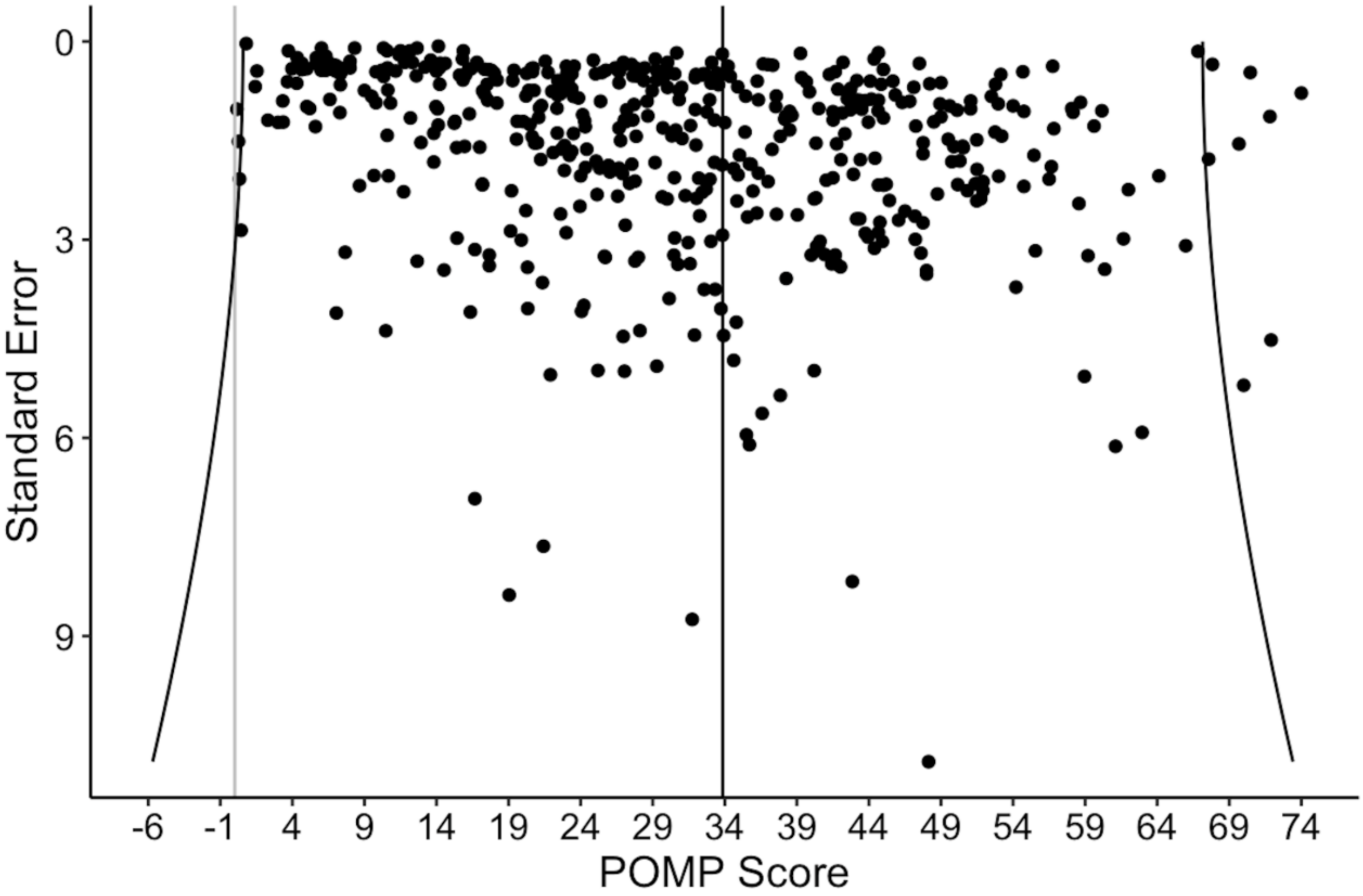

Supplementary Table 33. Preregistration deviations

#	Details		Original Wording	Deviation Description	Reader Impact, Feasibility and Appropriateness
**Deviations**					
1	Type	Variables	The effects of certain risk and protective factors of said mental health (COVID-19, workload, social support, work-life balance, work or life satisfaction, type of employment, stress, loneliness, etc.) as well as changes in prevalence over time on these outcomes will also be accounted for, if sufficient data can be gathered.	After thorough discussion we decided to keep stress, similar to suicidal ideation, and non-suicidal self-injury, as an outcome variable ('additional diagnostic criteria'). This better reflected the original idea of the meta-analysis to assess psychological distress in a broad manner.	When speaking of the overall estimated prevalence, it is necessary to keep in mind that this prevalence is made up of outcomes on the syndrome level (depression, GAD), symptom level (suicidal ideation, non-suicidal self-injury, stress), and diagnostic-criteria level (burnout).<br><br>The originally planned analysis was considered inappropriate, so it was not carried out any further
	Reason	Miscommunication			
	Timing	Before data collection			
2	Type	Variables	Mean and standard deviation, percentage/raw numbers above the clinical threshold or percentage/ raw numbers for each rung.	Mean and standard deviations were transformed into percent of maximum possible (POMP) scores, in order to align the varying scoring methods of the different scales to a common metric.	Outcomes in the POMP analyses are not on the original metric of each scale, but on a normalized metric from 0 to 100 to make outcomes comparable across different scales. POMP scores can range between 0 and 100. To interpret POMP scores, these must be back transformed into their respective original metrics.<br><br>The originally planned analysis was considered inappropriate, so it was not carried out any further.
	Reason	New knowledge			
	Timing	During data collection			
3	Type	Variables	To compare prevalence across different instruments with different rungs and thus non-binary diagnostic specification per disorder, we will also create a binary measure for estimating prevalence (0 = no/minimal depressive symptomatology, 1 = at least mild depressive symptomatology). For example, for depressive symptomatology, the Beck Depression Inventory (BDI) and Patient Health Questionnaire (PHQ9) use slightly different rungs.	To compare prevalence across different instruments, we utilized the psychometrically validated thresholds reported in the respective manuals, or thresholds which are defined per common practice. Not only do numeric thresholds vary per scale, but also the descriptive cut-offs. A detailed overview can be found in Supplementary Tables 20–29.	"Prevalence" in our analysis refers to the percent of persons above a predefined severity cutoff. For example, for the PHQ-9, a prevalence of 10% would indicate that 10% of persons were above the threshold for receiving a diagnosis of depression. This use of "prevalence" does not imply that, say, 10% had moderate-to-severe symptoms. Due to the different numeric and descriptive thresholds across the different scales included in analysis, such conclusions may not be drawn from the provided results.
	Reason	New knowledge			
	Timing	During data collection			

					The originally planned analysis was considered inappropriate, so it was not carried out any further.
4	Type	Variables	We will conduct moderator analyses for type (or class) of symptom or disorder, utilized scale or outcome measure, publication date, study quality and geographical origin of the sample.	The moderator variable publication date was replaced by year of data collection to gain insight into whether data were collected during the COVID-19 years. To this end, we also trichotomized year of data collection into three distinct phase: before (1979-2019), during (2020-2022) and after COVID-19 (2023-2024).<br><br>Additionally, we decided to create a categorization system clustering countries based on their similarity of pre- and post-doctoral research positions (contract, working hours, duration, etc.). For more information, see Supplementary Material 1 and Supplementary Tables 3–7.	These changes have a moderate impact. Most importantly, moderator analyses were conducted based on the accessibility and availability of moderators.<br><br>Using country clusters instead of countries increased the power of the moderator analysis without losing important information. Using distinct phases with regards to COVID-19 similarly increased power and did not rely on the assumption of linear associations of effect sizes with year of data collection.<br><br>The originally planned analysis was considered inappropriate, so it was not carried out any further.
	Reason	New knowledge			
	Timing	Before data access			
5	Type	Sample	The following databases were preregistered to be searched:<br><br>• Web of Science<br><br>• Scopus<br><br>• PubMed<br><br>• ERIC* (educational sciences)<br><br>• SAGE<br><br>• Google Scholar (first 300 hits)<br><br>• Backward reference search of prior meta-analytical reviews on mental health of early career researchers (Chi et al., 2023; Guo et al., 2021; Hazell et al., 2020; Satinsky et al., 2021)<br><br>• Forward reference search of prior reviews on mental health of early career researchers (Chi et al., 2023; Guo et al., 2021; Hazell et al., 2020; Jackman	The final search utilized the following databases:<br><br>• Web of Science<br><br>• Scopus<br><br>• PubMed<br><br>• ERIC*<br><br>• Backward reference search of prior meta-analytical reviews on mental health of early career researchers (Chi et al., 2023; Guo et al., 2021; Hazell et al., 2020; Satinsky et al., 2021)<br><br>• Forward reference search of prior reviews on mental health of early career researchers (Chi et al., 2023; Guo et al., 2021; Hazell et al., 2020; Jackman et al., 2022; Mackie & Bates, 2019; Martínez-García et al., 2024; Satinsky et al., 2021).<br><br>The screening of Scopus and backward and forward reference searches were done by author VG,	SAGE and Google Scholar were not searched, but this likely does not overly impact on readers' interpretation of the obtained results. However, as no Google Search was conducted, there might especially be gray literature which was not identified with the present approach.<br><br>SAGE and Google Scholar were not searched, but this likely does not overly impact on readers' interpretation of the obtained results. We do not expect that a search on SAGE would have identified further studies beyond those already identified. However, as no Google Search was conducted, there might especially be gray literature which was not identified with the present approach.
	Reason	Plan not possible			
	Timing	During data collection			

			et al., 2022; Mackie & Bates, 2019; Martínez-García et al., 2024; Satinsky et al., 2021)	searches in ERIC, PubMed and Web of Science were conducted by authors DF and VG.	
6	Type	Research Q(s)	- What is the state of mental health among early career researchers?<br><br>a) What is the prevalence of mental health syndromes [and diagnostic criteria] among early career researchers?<br><br>b) What factors mitigate/ aggravate mental health syndromes [and diagnostic criteria] among early career researchers?<br><br>c) How has the prevalence of certain mental health syndromes changed over time?	- What is the state of psychological distress among ECR?<br><br>a) What is the prevalence of psychological distress among ECR?<br><br>b) What factors mitigate/aggravate psychological distress among ECR?<br><br>We decided to alter the terminology from 'mental health' to 'psychological distress' to better encompass all assessed outcomes (on the level of syndromes, symptoms, and diagnostic criteria).<br><br>Additionally, we deleted the last research question, and instead investigate the COVID-19 years, which can be considered a temporal risk factor (point b).	Changes entailed clarifications so no negative impact is expected.<br><br>Changes entailed clarifications, so no negative impact is expected and originally planned analyses were considered inappropriate, which is why they were not carried out any further.
	Reason	New knowledge			
	Timing	Before data access			
**Unregistered Steps**					
#	Details		Original Wording	Unregistered Step Description	Reader Impact
1	Type	Sample	The following databases were preregistered to be searched:<br><br>[…]	During the screening of the articles, it became apparent that several publications utilized the same datasets. We therefore decided to directly include the primary data instead of the articles reporting on these data, resulting also in a screening of the databases.<br>Four databases were included in analysis (Buffel & Velde, 2022; Byrom & Metcalfe, 2021; Hazell et al., 2020; Healthy Minds Network et al., 2024).	There is no direct impact on the readers' interpretation of the study or the results.<br><br>The originally planned analysis was considered inappropriate, because it would have combined the results of different papers using the same data sources, instead of using these sources directly; so it was not carried out any further.
	Timing	During data collection			
2	Type	Variables		Non-suicidal self-injury (NSSI) behavior was an additional diagnostic criterion that was included as	The outcome variables encompassed not only syndromes, but also symptoms, and additional

	Reason	New knowledge	Additional diagnostic criteria for certain mental disorders will also be included, for example suicidal ideation as a symptom of major depression.	an outcome variable. Like suicidal ideation, NSSI can be understood as a symptom (e.g., of depression or borderline personality disorder).	diagnostic criteria, which we subsumed more broadly under the term 'psychological distress.'<br><br>The originally planned analysis was carried out, with the addition of a further outcome variable. All information on this ouctome variable can be found in the main text.
	Timing	During data collection			
3	Type	Analysis	-	A random forest analysis was utilized to assess variable importance and determine the explained variance. Due to the high missingness of many variables a multivariate moderator model was run additionally. The random forest analysis is only presented in Supplementary Material 2 and Supplementary Figs. 16 and 17.	There is no direct impact on the readers' interpretation of the study or the results, other than the reader should know the exact statistical procedure and decisions taken.<br><br>This analysis was added to the originally planned analysis Full results can be found in Supplementary Material 2.
	Reason	New knowledge			
	Timing	After results known			

Willroth, E. C., & Atherton, O. E. Best laid plans: A guide to reporting preregistration deviations. *Advances in Methods and Practices in Psychological Science* **7**(1). (2024). https://doi.org/10.1177/25152459231213802

Supplementary Table 34. Scopus, ERIC, Web of Science and PubMed Search String

**Type of research position**	"early*career researcher" OR “ecr” OR "doctor* candidate" OR "doctor* level" OR "doctor* researcher" OR "graduate student" OR "phd candidate" OR "phd student" OR "pgr" OR "Ph.D." OR "phd" OR "post-graduate research student" OR "postgraduate research student" OR "postgraduate researcher" OR "post-graduate researcher" OR "post-doc" OR "research student" OR “postdoc*”
	AND
**Syndromes and/ or symptoms of psychological distress**	"mental illness" OR "psychiatric illness" OR "psychiatric problems" OR "psychiatric symptom*" OR "psychological well-being" OR "psychopathology" OR "disorder" OR "psychopathology" OR "mental health" OR "post-traumatic" OR "stress" OR "burnout" OR "distress" OR "PTSD" OR "PSS" OR "OCD" OR "obsessive-compulsive" OR "obsessive compulsive" OR "addiction" OR "abuse" OR "substance abuse" OR "addictive behavior" OR "anorexia" OR "eating disorder" OR "ED" OR "body dysmorph*" OR "binge eating" OR "bulimia" OR "depress*" OR "cyclothymic" OR "mood disorder" OR "suicide" OR "dysthymic" OR "dysphoric" OR "mania" OR "manic" OR "bipolar" OR "affective disorder" OR "anxiety" OR "panic attacks" OR "GAD" OR "general anxiety disorder" OR "anxiety disorder" OR "panic disorder" OR "social phobia" OR "phobia" OR "insomnia" OR "sleep-wake disorder" OR "sleep disorder" OR "delus*" OR "dissociative" OR "hallucinat*" OR "psychosis" OR "psychotic" OR "SAD" OR "shizo*" OR "personality disorder" OR "PD" OR "hoarding" OR "hypochondriasis" OR "loneliness" OR "MDD" OR “self-harm” OR “suicidality” OR “suicide*” OR “drug abuse” OR “DAST” OR “AUDIT” OR “NSSI”
	AND
**Type of study**	“quant*” OR “survey” OR “cross-sectional” OR “questionnaire”

Supplementary Table 35. Mailing Template

**General template**	Dear [enter name],<br>I hope this message finds you well.<br>I am a MSc student of Psychology at the University of Vienna, and I'm conducting a meta-analysis on mental health of Early-career researchers, along with my co-authors Dr. Aljoscha Dreisörner, Univ.-Prof. Dr. Dr. Dr. Martin Voracek, Univ.-Prof. Dr. Urs Markus Nater and Dr. Ulrich Tran.<br>…<br>As you have published studies relevant to this topic, we are getting in touch to see if you have any unpublished/file-drawer data, or paper in-press, which we may have missed through database searching, and which you would like to have included in the meta-analysis.<br>We are hoping to include as many relevant studies as possible, so any additional data is greatly appreciated.<br>Thank you in advance & kind regards,<br>Vanessa Götz
**Call for paper**	Your paper [enter title] came up in my search. Unfortunately, I have no access to the paper via my affiliation with the University of Vienna. Would it thus be possible for you to share the paper with me?
**Call for information**	Your paper [enter title] came up in my search. Unfortunately, I could not determine whether or not you have PhD students included in your sample. Would it be possible for you to clarify, and if you have collected data differentiating PhD [alternatively post-doctoral researchers] from MA students? And if so, would it be possible for you to provide me with the relevant data (mean, SD, % per rung, % above clinical cut-off) of the PhD [alternatively post-doctoral researchers] subsample on the [scales] as well as socio-demographics?<br>Feel free to email either the raw data or the summary scores themselves. While any raw data emailed to us will of course remain confidential, please know that summary scores included in the meta-analysis will be made publicly available in a dataset on the OSF.
**Call for unpublished data**	Your paper [enter title] came up in my search. Unfortunately, I am missing data – would it be possible for you to provide the following to me:<br>- [enter scale and missing data]<br>Feel free to email either the raw data or the summary scores themselves. While any raw data emailed to us will of course remain confidential, please know that summary scores included in the meta-analysis will be made publicly available in a dataset on the OSF.

Supplementary Table 36. Coding guide

**Source Characteristics**			
**Source**		0<br>1<br>2<br>3	Bibliography<br>Search String<br>Other<br>Forward Search
**Study ID**	Give each study a unique three-digit study ID in the following format:<br>Use the first two digits to number the reports and the last digit to identify the study within the report (if applicable). If the report only consists of one study, use 0 as the last digit.<br>*Example:*<br>*Report A (2 studies) → Study ID: 011 (for study 1) and 012 (for study 2)*<br>*Report B (1 study) → Study ID: 020 (since there is only one study)*<br>*Report C (3 studies) → Study ID: 031, 032, and 033 (for study 1, 2, and 3, respectively)*		
**Authors**	Name the authors as you would in an in-text citation:<br>If there is one author, enter their last name.<br>If there are two authors, enter both their last names (separated by " & ").<br>If there are three or more authors, enter the first author's name followed by " et al.".		
**Type of Report**	Please indicate what kind of report this was. The report categories are mutually exclusive.	JA<br>BOOK<br>DISS<br>MA<br>PR<br>GR<br>CP<br>WP<br>PP<br>DATA<br>?	Journal Article<br>Book<br>Dissertation<br>Master Thesis<br>Private Report<br>Government Report<br>Conference Paper<br>Working Paper<br>Preprint<br>Database<br>Other, Unknown
**Peer-Review**	Has the report been published in a peer-reviewed journal?	0<br>1<br>?	Yes<br>No<br>Unknown, N/A
**Year of Publication**	Enter the year of the publication.<br>*Example: 2022*		
**COVID-19 Characteristics**			
**Year of Data Collection**	Please enter the year in which the data was collected, this can deviate from the year of publication and might not always be provided. If not provided, we use an estimate based on when the article was received, the study was approved or whether COVID-19 was mentioned.		

	Example: 2022		
**Point in Time**	Some studies might capture a pre- post-assessment of mental health before and after the COVID-19 pandemic or throughout several lockdowns. Some studies have also captured several points in time of data collection in the course of one year, to differentiate within the dataset, points in time have been introduced.	T1<br>T2<br>T3	First point of time in which data was collected<br>Second point in time in which data was collected<br>Third point in time in which data was collected
**Sample Characteristics**			
**Country**	Enter the three-letter country code according to ISO 3166-1 alpha-3. Countries included in the original meta-analysis are listed below, others can be looked up here: https://en.wikipedia.org/wiki/ISO_3166-1_alpha-3 and should be added to the list below upon use.<br>In case the study has collected data from researchers in different countries, the "INT" category is to be used.	AUS<br>BEL<br>CAN<br>ESP<br>GBR<br>INT	Australia<br>Belgium<br>Canada<br>Spain<br>UK, GB, and Northern Ireland<br>International
**Sample Size**	Enter the total number of participants as stated in the report. To capture the distinction between more than one study conducted in one paper (or so) provide information on the sample size (and subsequent information) per study conducted according to the respective ID and respective year in which the data was collected.<br>To capture the distinction between more than one sample groups of interested, namely PhD and post-doctoral students, provide information on the sample size (and subsequent information) per study conducted on the respective sub-samples according to the respective ID.<br>In case PhD students or post-doctoral researchers constitute a sub-sample within a greater sample, only the information on the respective sub-groups is of interest.		
**Type of Participants**	Which population was sampled?	0<br>1<br>?	PhD<br>Post-Doc<br>Other
**Inclusion Criteria**	In case special inclusion or exclusion criteria was applied to the sample, please indicate which this was.<br>*Example: [PhD in political science]*		
**Age Distribution**	Enter the mean (SD) age of the sample. In case the sample is "graduate", our sample of interest are only those studying to obtain a PhD, thus all further socio-demographic and mental health information shall only be collected in conjunction to the sample of interest (PhD, post-doc).<br>*Example: 25,8 (2,7)*		
**Gender Distribution**	Enter the percentage of students identifying as female within the population of interest.<br>Enter the percentage of students identifying as diverse (trans, queer, a, etc.) within the population of interest.		
**Ethnicity**	Please enter the ethnicity of the sample in percentage.	MidEast<br>Latin<br>Caucasian<br><br>Asian<br>Black<br>Other	Middle Eastern<br>South American, Hispanic, Latin American, Caribbean,<br>White, European, North American (excl. Native American), Oceania (excl. Native Islanders)<br>South, North, East and West Asia (excl. Russia)<br>African, African-American<br>Other such as Native American, Pacific Islanders, etc.

**Years in Academia**	Please provide information on the mean (SD) years in academia.		
**Faculty Affiliation**	Provide information with which faculties the sample is affiliated in percentage per field of doctoral study. If a distinction is not clear, find more information here: https://en.wikipedia.org/wiki/List_of_fields_of_doctoral_studies_in_the_United_States<br>If no information is given, please leave blank or contact the author(s).	LS<br>ENG<br>CIS<br>MAT<br>PS<br>PSY<br>SS<br>HU<br>EDU<br>BMA<br>COM<br>MED<br>LAW<br>ETC	Life Sciences<br>Engineering<br>Computer and Information Sciences<br>Mathematics<br>Physical Sciences<br>Psychology<br>Social Sciences<br>Humanities<br>Education<br>Business Management/ Administration<br>Communication<br>All medical fields of study (incl. dentistry, veterinary, nursing, etc.)<br>Law<br>Fields not elsewhere classified
**Workload per Week**	Please provide information on the mean (SD) workload per week in h/week.		
**History of Psychiatric Disorders**	Please provide information on the percentage of students having been diagnosed with any kind of psychiatric disorder before having partaken in the study.<br>Sometimes information is provided on when they have gotten their diagnosis, if this is before their PhD/ post-doctoral position enter in the column "before", if during their PhD/ post-doctoral research enter in "after". If it is not provided or unclear, enter in "unclear".		
**Measurement Characteristics**			
Please provide information on the respective outcome variables.<br>Information should be provided for the respective scale in general as well as the designated columns for this scale. Information should be provided for the mean (SD), and the percentage above threshold, if the scale permits for it.<br>In case a scale is used which assesses a multitude of mental health aspects, only that sub-scale is of relevance that measures depression (example DASS, GHQ-28, HADS, etc.).<br>If information on several scales is provided, enter the information for each scale in the designated columns.<br>In case a scale is lacking, as it only appears once and only mean (SD) is provided, it is sufficient to enter it only in the general columns.<br>More information on each scale can be found in the scale guide.			
**ADHD**		ASRS-VI.I<br>SCID-5-RV<br>Other	Adult ADHD Self-Reporting Scale<br>Structured Clinical Interview for DSM-5, Revised<br>In case the scale is not clearly stated, and the source is referred to or the item-number.
**Alcohol Abuse**		AUDIT<br>AUDIT-C	Alcohol Use Disorder Identification Test, 10-item version<br>Alcohol Use Disorders Identification Test-Concise, 3-item version

		SCID-5-RV<br>SASS<br><br>Other	Structured Clinical Interview for DSM-5, Revised<br>Symptoms and Assets Screening Scale,<br><br>In case the scale is not clearly stated, and the source is referred to or the item-number.
**Anxiety**		BAI<br>BSI<br>DASS<br>DASS-21<br>GAD-7<br>GAD-2<br>GHQ-28<br>HADS<br>MHI<br>SASS<br>SCID-5-RV<br>STAI<br><br>Other	Beck Anxiety Inventory, 21-item version<br>Brief Symptom Inventory, 53-item version<br>Depression, Anxiety and Stress Scale, 42-item version<br>Depression, Anxiety and Stress Scale, 21-item version<br>Generalized Anxiety Disorder, 7-item version<br>Generalized Anxiety Disorder, 2-item version from PHQ-4<br>General Health Questionnaire, 28-item version<br>Hospital Anxiety and Depression Scale, 14-item version<br>Mental Health Inventory<br>Symptoms and Assets Screening Scale<br>Structured Clinical Interview for DSM-5, Revised<br>State-Trait Anxiety Inventory, 40-item version<br><br>In case the scale is not clearly stated, and the source is referred to or the item-number.
**Burnout**	Please provide information on overall burnout. Information should be provided for the respective scale in the designed columns for this scale. Information should be provided for the mean (SD), and the percentage above threshold, if the scale permits for it.	MBI-ES-22<br>MBI-22<br>MBI-SS<br>MBI<br>MBI-9<br>MBI-12<br>BMS<br>SBI-9<br>CBI<br>MED NORD<br><br>Other	Maslach-Burnout Inventory,<br>Maslach-Burnout Inventory, 22-item version<br>Maslach-Burnout Inventory Student Version, 15-item version<br>Maslach-Burnout Inventory,<br>Maslach-Burnout Inventory, 9-item version<br>Maslach-Burnout Inventory, 12-item version<br>Burnout Measure Scale, 10-item version<br>School Burnout Inventory, 9-item version<br>Copenhagen Burnout Inventory, 13-item version<br>MED NORD<br><br>In case the scale is not clearly stated, and the source is referred to or the item-number.

**Depression**		BDI<br>BDI-SF<br>BSI<br>CESD or CES-D<br>CESD-R or<br><br>CES-D-R<br>CESD-10 or<br>CES-D-10<br>CESD-R-10<br>DASS-42<br>DASS-21<br>GHQ-12<br>GHQ-28<br>HADS-D<br>MHI<br>PHQ-9<br>PHQ-8<br>PHQ-2<br><br>SASS<br>Other	Beck Depression Inventory, 21 items<br>Beck Depression Inventory, Short Form<br>Brief Symptom Inventory, 53-item version<br>Center for Epidemiological Studies Depression, 20-item version<br>Center for Epidemiological Studies Depression Revised, 20-item version<br>Center for Epidemiological Studies Depression, 10-item version<br>Center for Epidemiological Studies Depression Revised, 10-item version<br>Center for Epidemiological Studies Depression, 8-item version<br>Depression, Anxiety and Stress Scale, 42 items<br>Depression, Anxiety and Stress Scale, short version<br>General Health Questionnaire, 12-item version<br>General Health Questionnaire, 28-item version<br>Hospital Anxiety and Depression Scale, 14-item version<br>Mental Health Inventory<br>Patient Health Questionnaire, with item 9 for suicidal ideation<br>Patient Health Questionnaire, without item 9<br>Patient Health Questionnaire, known as PHQ-4 but contains only two items on depression<br>Symptoms and Assets Screening Scale<br>In case the scale is not clearly stated, and the source is referred to or the item-number.
**Drug Abuse**		DAST<br>Other	Drug Abuse Screening Test, 10-item version<br>In case the scale is not clearly stated, and the source is referred to or the item-number.
**Eating Disorder**		BSI<br>SASS<br>SCOFF<br>SCID-5-RV<br>Other	Brief Symptom Inventory, 53-item version<br>Symptoms and Assets Screening Scale<br>Sick, Control, One, Fat, Food Questionnaire, 5-item version<br>Structured Clinical Interview for DSM-5, Revised<br>In case the scale is not clearly stated, and the source is referred to or the item-number.
**Mental Health**		GHQ-12<br>GHQ-28<br>K6<br>K10<br>MHC-SF<br>PWBS<br>PWS<br>SASS<br>SF-36	General Health Questionnaire, 12 items<br>General Health Questionnaire, 28 items<br>Kessler Psychological Distress Scale, 6 items<br>Kessler Psychological Distress Scale, 10 items<br>Mental Health Continuum – Short Form<br>Personal Wellbeing Scale<br>Perceived Wellness Survey<br>Screening, Assessment and Support Services<br>36-Item Short Form Survey Instrument (RAND)

		SWEMWBS<br>WEMWBS<br>WHO-5 index	Short Warwick Edinburgh Mental Wellbeing Scale<br>Warwick-Edinburgh Mental Well-Being Scale<br>World Health Organization Well-Being Index, 5-items
**Mood Disorder**		SCID-5-RV<br>Other	Structured Clinical Interview for DSM-5, Revised<br>In case the scale is not clearly stated, and the source is referred to or the item-number.
**OCD**		BSI<br>Other	Brief Symptom Inventory, 53-item version<br>In case the scale is not clearly stated, and the source is referred to or the item-number.
**Personality Disorder**		SCID-5-PD<br>Other	Structured Clinical Interview for DSM-5, Personality Disorder<br>In case the scale is not clearly stated, and the source is referred to or the item-number.
**Suicidal Ideation**		PSQ-9, item 9<br>SBQ-R<br>BDI-II, item 9<br>Other	Patient Health Questionnaire, item 9<br>Suicide Behaviors Questionnaire – Revised<br>Beck Depression Inventory, item 9<br>In case the scale is not clearly stated, and the source is referred to or the item-number.
**Sleep Quality**	Please provide information on overall sleep quality. Information should be provided for the respective scale in the designed columns for this scale. Information should be provided for the mean (SD), and the percentage above threshold, if the scale permits for it.	JSQ-4<br>PSQI<br>Other	Jenkins Sleep Evaluation Questionnaire, 4-item version<br>Pittsburgh Sleep Quality Index<br>In case the scale is not clearly stated, and the source is referred to or the item-number.
**Stress**	Please provide information on overall perceived stress. Information should be provided for the respective scale in the designed columns for this scale. Information should be provided for the mean (SD), but also percentage of those individuals scoring above a certain threshold. More information on the cut-offs can be found in the Manual on Scales.	DASS<br>DASS-21<br>PSQ<br>PSS-4<br>PSS-10<br>PSS-14<br>BAROMAS<br>SASS<br>Other	Depression, Anxiety and Stress Scale, 42-item version<br>Depression, Anxiety and Stress Scale, 21-item version<br>Parental Stress Questionnaire<br>Perceived Stress Scale, 4-item version<br>Perceived Stress Scale, 10-item version<br>Perceived Stress Scale, 14-item version<br>Bandura-Rosenthal Metrics for Assessing Stress<br>Screening, Assessment and Support Services<br>In case the scale is not clearly stated, and the source is referred to or the item-number.
**Potential Protective Factors**			
**Advisor Relationship**	Please provide information on overall perceived advisor relationship. Information should be provided for the respective scale in the designed columns for this scale. Information should be provided for the mean (SD).	AWAI-30<br>AWAI-S<br>ARI-11<br>Other	Advisory Working Alliance Inventory<br>Advisory Working Alliance Inventory – Student perspective<br>Advisor Relationship Index, 11-item version

			In case the scale is not clearly stated, and the source is referred to or the item-number.
**Life Satisfaction**	Please provide information on overall perceived life satisfaction. Information should be provided for the respective scale in the designed columns for this scale. Information should be provided for the mean (SD).	SWLS<br>QoL<br>QOLI<br>Other	Satisfaction with Life Scale, 5-item version<br>Quality of Life<br>Quality of Life Inventory<br>In case the scale is not clearly stated, and the source is referred to or the item-number.
**Job Satisfaction**	Please provide information on overall perceived job satisfaction. Information should be provided for the respective scale in the designed columns for this scale. Information should be provided for the mean (SD).<br>If the scale is not provided on this list, just enter the source as follow: Author (Year)	Other	In case the scale is not clearly stated, and the source is referred to or the item-number.
**Social Support**	Please provide information on overall perceived social support. Information should be provided for the respective scale in the designed columns for this scale. Information should be provided for the mean (SD).	MSPSS<br>SPOS-4<br>QSDI<br>SSRS<br>ISEL<br>Other	Multidimensional Scale for Perceived Social Support<br>Survey of Perceived Organizational Support, 4-item version<br>Questionnaire on Supervisor–Doctoral Student Interaction<br>Social Support Rate Scale<br>Interpersonal Support Evaluation List, 12-item version<br>In case the scale is not clearly stated, and the source is referred to or the item-number.
**Work-Life Balance**	Please provide information on overall perceived work-life balance. Information should be provided for the respective scale in the designed columns for this scale. Information should be provided for the mean (SD).<br>If the scale is not provided on this list, just enter the source as follow: Author (Year)	Other	In case the scale is not clearly stated, and the source is referred to or the item-number.
**Potential Risk Factors**			
**Intention to Quit**	Please provide information on overall intention to quit. Information should be provided for the respective scale in the designed columns for this scale. Information should be provided for the mean (SD), and the percentage above threshold, if the scale permits for it.	ITQ<br>Other	Intention to Quit Scale, 7-item version<br>In case the scale is not clearly stated, and the source is referred to or the item-number.
**Loneliness**	Please provide information on overall perceived loneliness. Information should be provided for the respective scale in the designed columns for this scale. Information should be provided for the mean (SD), and the percentage above threshold, if the scale permits for it.	UCLA-3<br>UCLA-8<br>UCLA-20<br>DJGLS-11<br>Other	University of California Loneliness Scale, 3-item version<br>University of California Loneliness Scale, 8-item version<br>University of California Loneliness Scale, 20-item version<br>De Jong Gierveld Loneliness Scale, 11-item version<br>In case the scale is not clearly stated, and the source is referred to or the item-number.

Supplementary Table 37. Algebraic recalculation of missing summary scores

Calculation	Study
Means and standard deviations based on percentages per rung(s) for the respective scales	• Ahalli et al., 2022<br>• Almasri et al., 2022<br>• Bastos et al., 2025<br>• Doom et al., 2021<br>• Bork-Young, 2023<br>• Estupiñá et al., 2024<br>• Fernández-Rodríguez et al., 2024<br>• Gaudel et al., 2024<br>• Hu et al., 2020<br>• Hulac et al., 2024<br>• Li & Su, 2024<br>• Lt et al., 2022<br>• Marais et al., 2018<br>• Mavrogalou-Foti et al., 2024<br>• Mills et al., 2024<br>• Panayidou & Priest, 2021<br>• Ramadoss et al., 2022<br>• Ramadoss & Horn, 2022<br>• Schad et al., 2022<br>• Wang et al., 2022

Supplementary Material 2. Multivariate exploration of important moderators with a random forest analysis

To assess the relative importance of the moderators, a machine-learning approach based on random forests was used. The relative importance is based on the respective proportion each moderator explains of the effect-size heterogeneity (van Lissa, 2020a). Further advantages of MetaForest are that it may capture non-linear relationships and is robust to overfitting. However, MetaForest requires complete data. Only moderators with missing values < 10% were included in the random-forest analysis (see Supplementary Table 10 for % of missingness per variable). The default settings of the random-forest analysis were set to 100-fold replicated feature selection, retaining only moderators with positive variable importance in > 50% of replications, and 10,000 regression trees with random-effect weights.

In both models, the type of syndrome and symptom, the scale used, overall study quality, sampling strategy, COVID-19 period, year, country cluster, US sample, language of records, peer review status, and position of ECRs were included in the analysis. In the prevalence model, language was excluded by the procedure (Supplementary Fig. 16) and the combined remaining moderators explained R^2_{OOB} = 31.49% of the variance. In the severity model, the moderators COVID-19 period and country cluster were excluded by the procedure (Supplementary Fig. 17) and the combined remaining moderators explained R^2_{OOB} = 42.22% of the variance.

Supplementary Figure 16. Convergence and recursive variance importance plot for the prevalence model

(A) Convergence plot

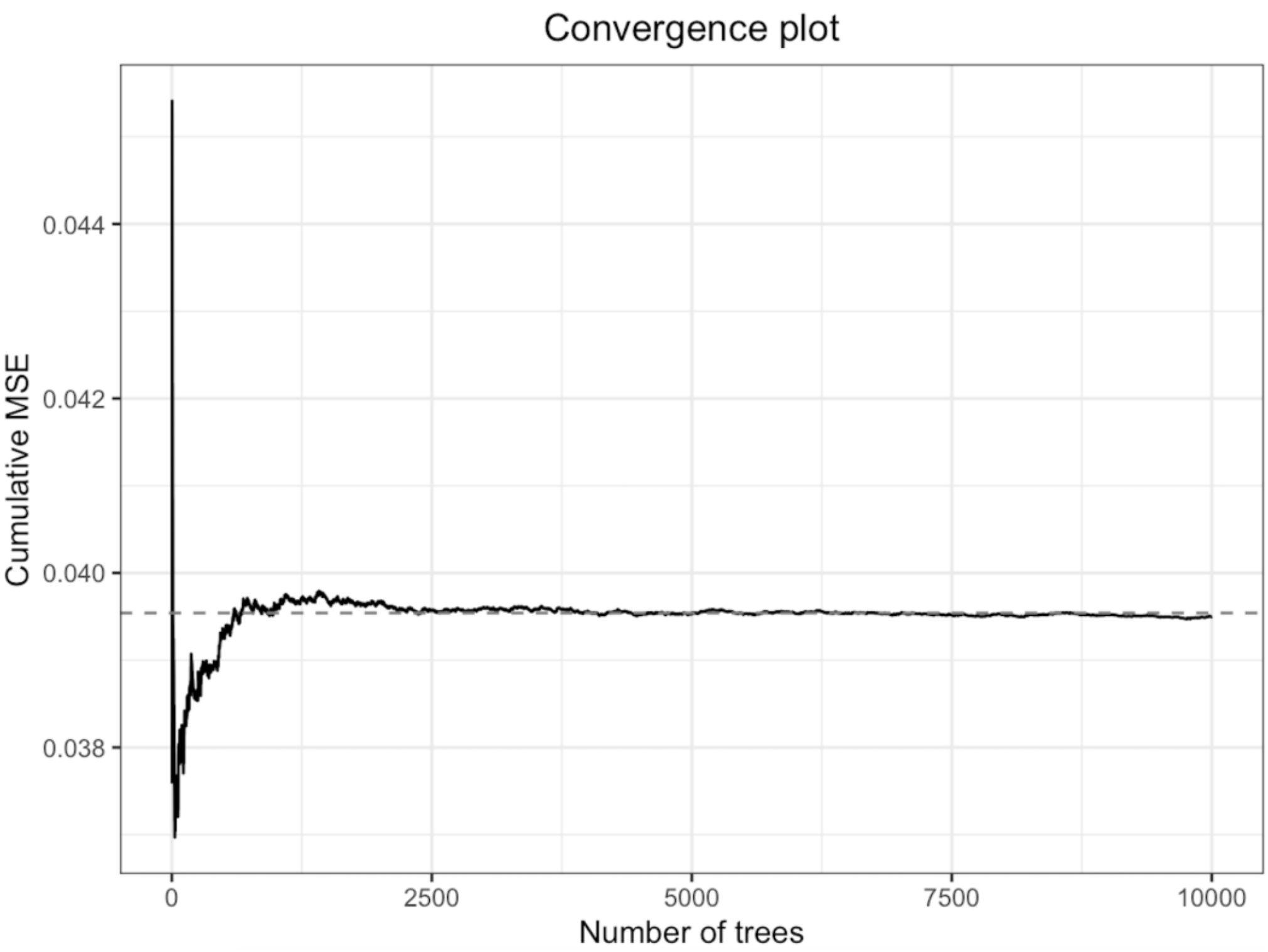

(B) Recursive variable importance plot

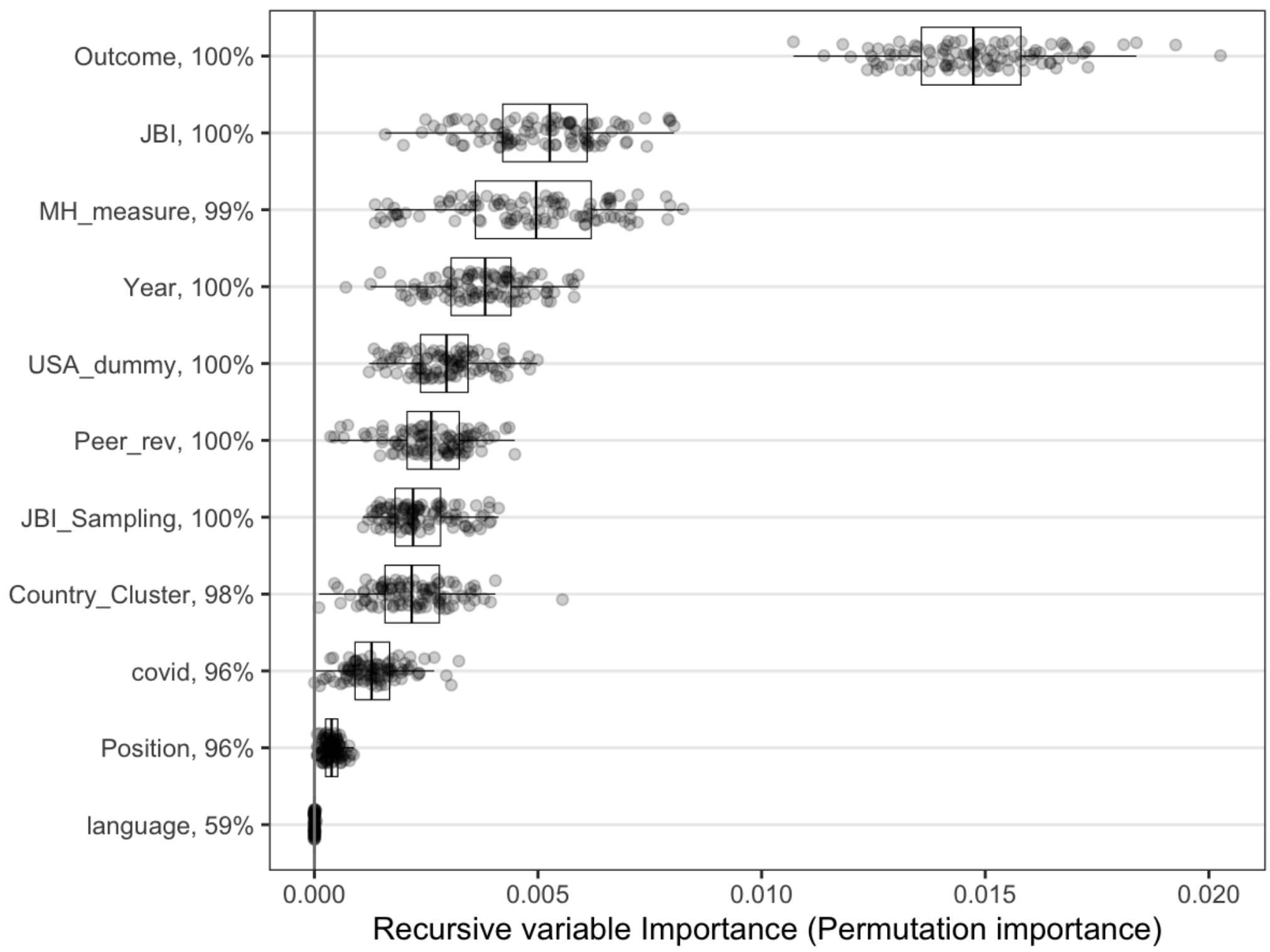

Note. Outcome = mental health syndrome or symptom; JBI = critical appraisal of study quality; Year = year of data collection; JBI_Sampling = use of convenience versus random sampling; Position = pre-doctoral versus post-doctoral ECR status; covid = whether the study was conducted during the

COVID-19 period (2020–2022); MH_Measure = scale used to assess the mental health outcome; Country_Cluster = grouping based on similar working conditions for early career researchers; Language = English versus machine-translated record; Peer_rev = peer-reviewed publication status.

Supplementary Figure 17. Convergence and recursive variance importance plot for the POMP-score model

(A) Convergence plot

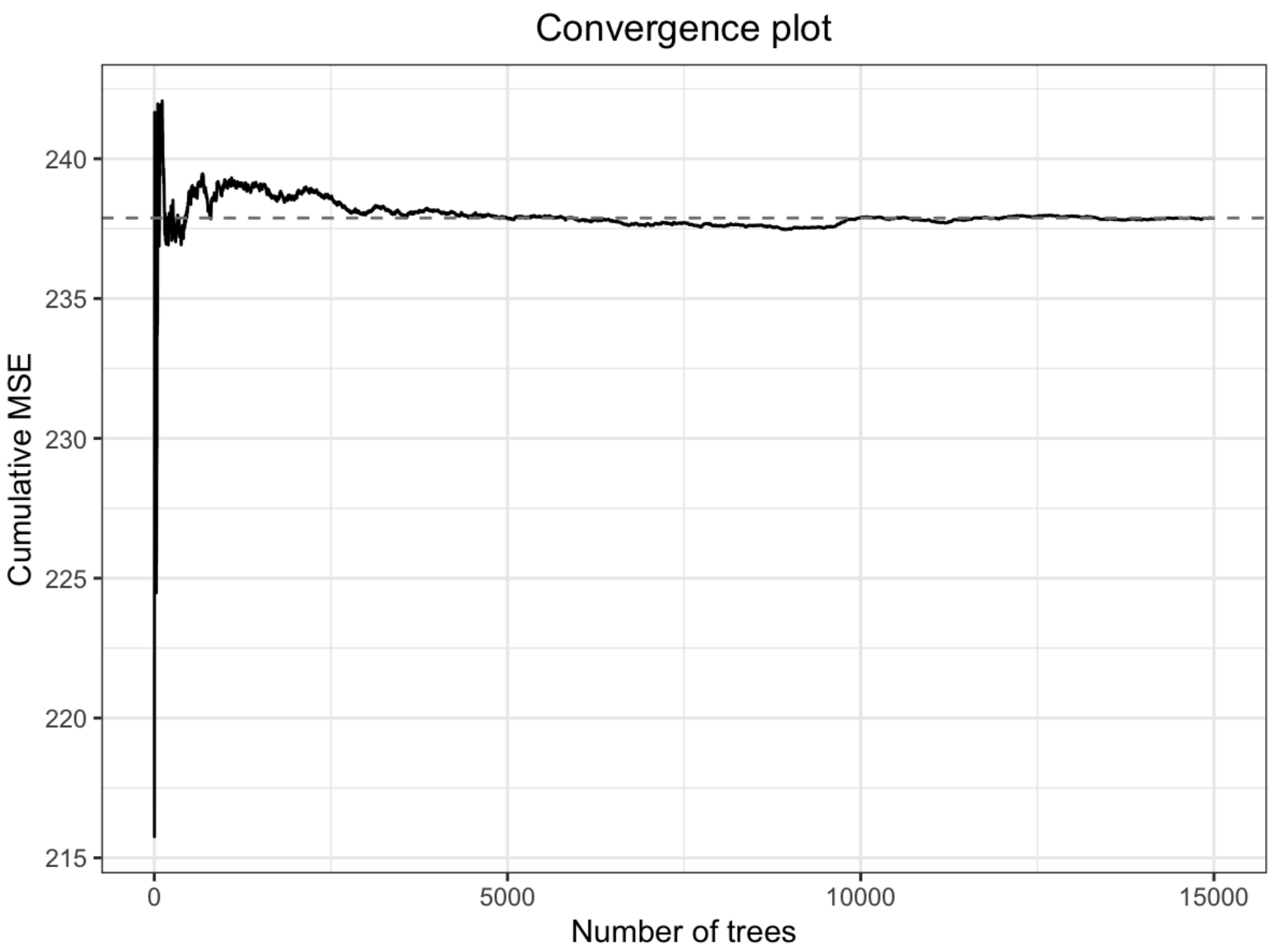

(B) Recursive variable importance plot

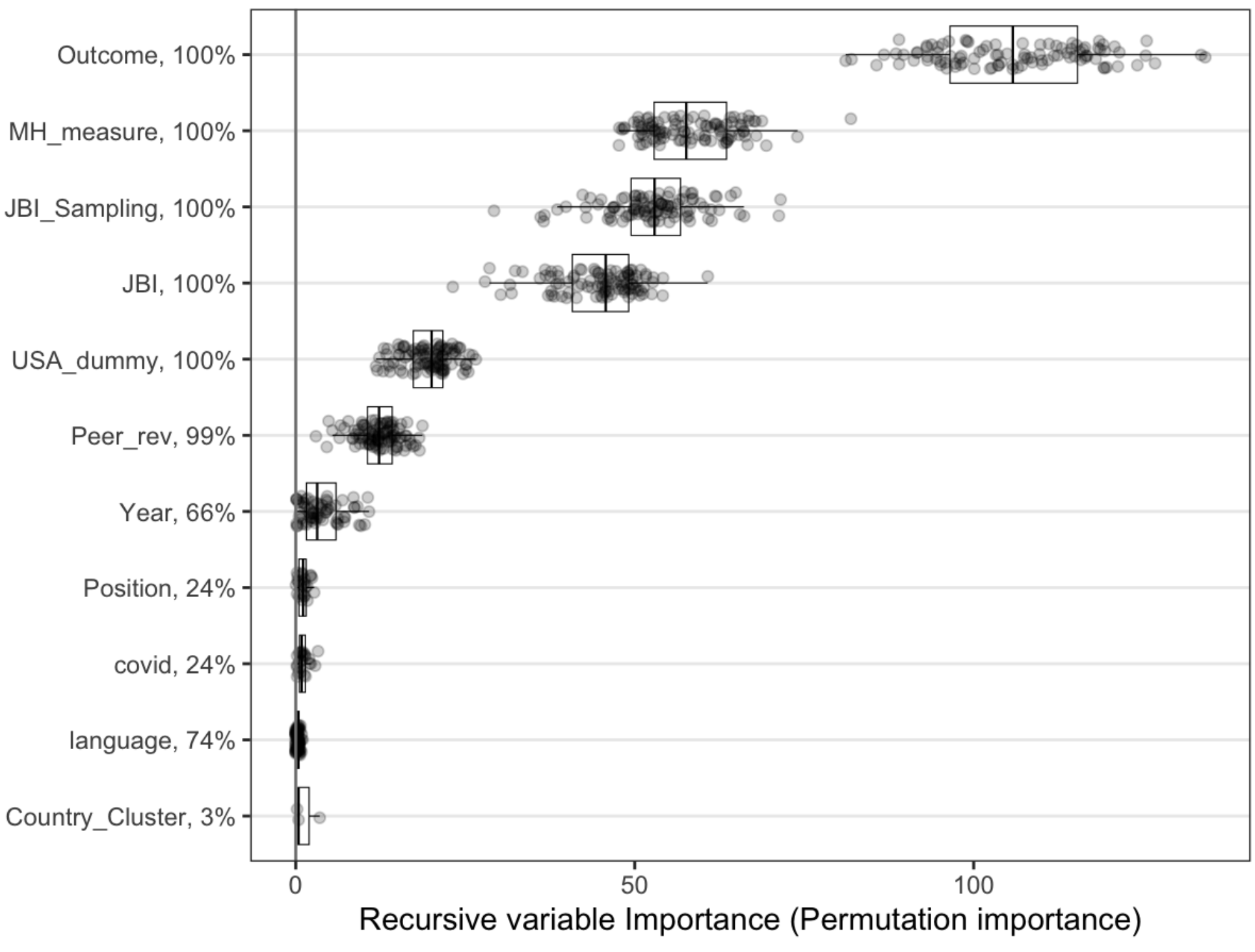

Note. Outcome = mental health syndrome or symptom; JBI = critical appraisal of study quality; Year = year of data collection; JBI_Sampling = use of convenience versus random sampling; Position = pre-doctoral versus post-doctoral ECR status; covid = whether the study was conducted during the COVID-19 period (2020–2022); MH_Measure = scale used to assess the mental health outcome; Country_Cluster = grouping based on similar working conditions for early career researchers; Language = English versus machine-translated record; Peer_rev = peer-reviewed publication status.

Supplementary Figure 18. Outlier and sensitivity analysis with Cook's Distance for the

(A) prevalence model

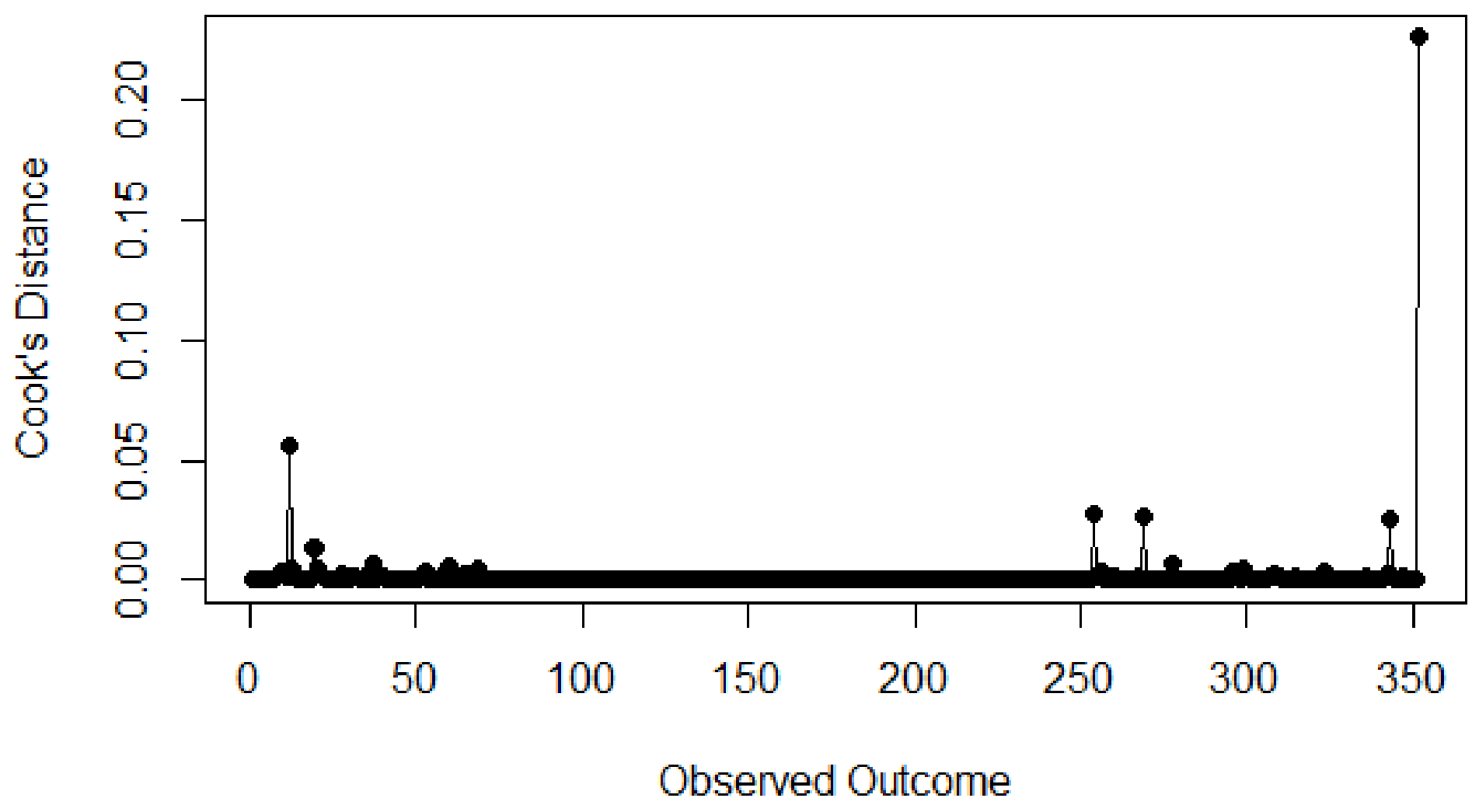

(B) severity model

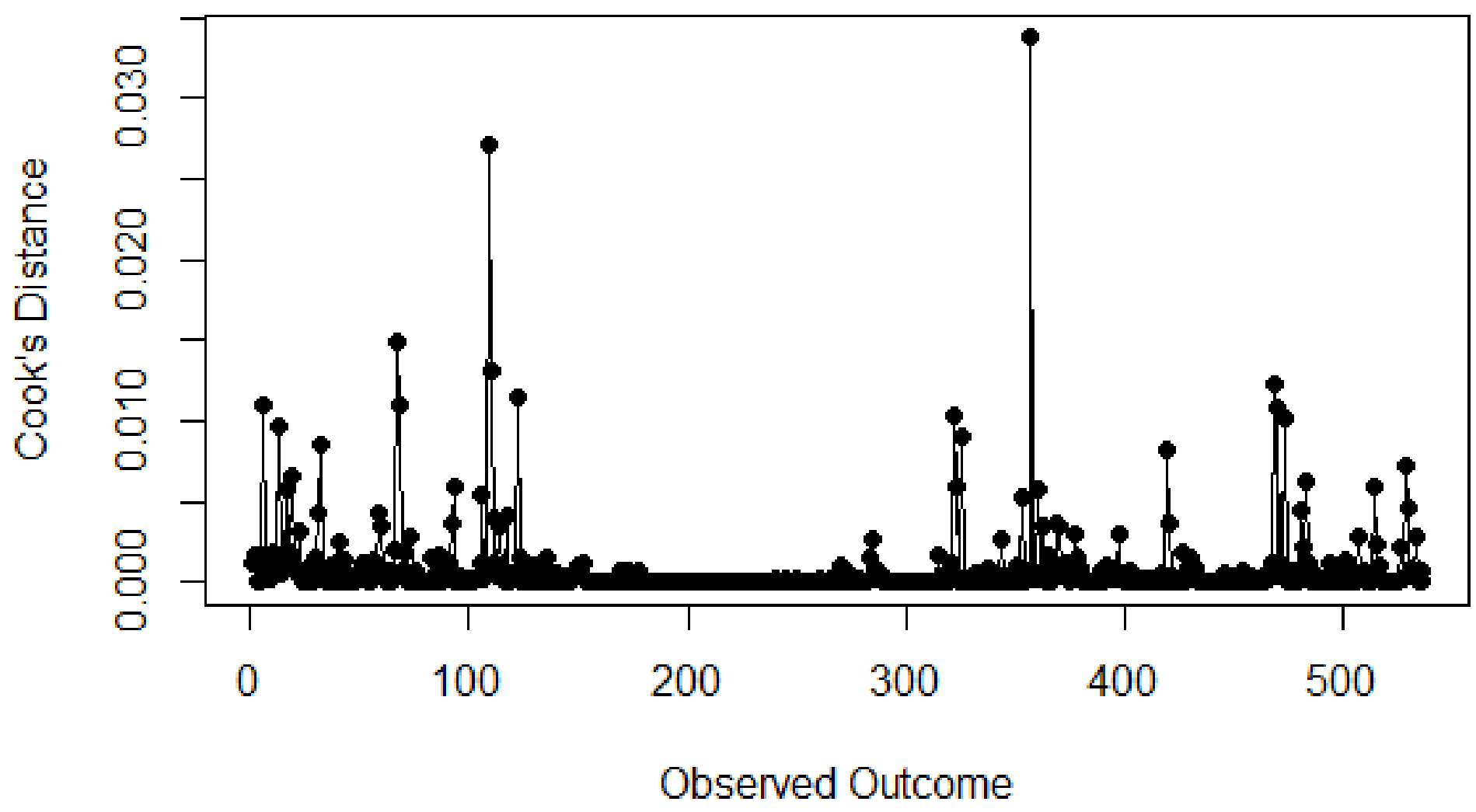

Supplementary Table 38. Comparison of models with and without outliers

	**Pooled Effect β (95% CI)**	**Residual Heterogeneity**
**POMP scores**		
Model with outliers	33.89 [31.66–36.13]***	*Q*(535) = 864,833.18***
Model without outliers	33.13 [30.96–32.29]***	*Q*(517) = 694,931.03***
**Prevalence**		
Model with outliers	29.88 [26.06–33.86]***	*Q*(351) = 32,550.35***
Model without outliers	27.25 [23.95–30.68]***	*Q*(344) = 29,086.59***

***$p < .001$.

Supplementary Table 39. Information on studies excluded from the meta-analysis based on reasons for exclusion

Publication	Reason for exclusion	Publication	Reason for exclusion
Aalim & Ambily, 2019	not available	Lin et al., 2023	mail delivery failed
Shakir et al., 2023	sample	Rini & Provident, 2024	sample
Abelson et al., 2022	no primary research	Lindstrom et al., 2023	no primary research
Abu Ruz et al., 2018	sample (BA, MA)	Ling & Qian, 2010	insufficient response
Abuelezam et al., 2022	no primary research (HMS)	Lipskaya-Velikovsky et al., 2025	no response
Addonizio, 2011	sample (MA)	Lipson & Sonneville, 2017	no response
Adhikary et al., 2024	no response	Lipson et al., 2016	no primary research (HMS)
Afridi et al., 2020	no response	Lipson et al., 2018	no primary research (HMS)
Agroia et al., 2024	wrong focus	Lipson et al., 2019	no primary research (HMS)
Ahorsu et al., 2020	sample: MA	Lister et al., 2024	not available
Ahuja & Kumar, 2015	no response	Liu et al., 2023	no response
Akram et al., 2021	no response	Liu et al., 2022	wrong focus
Al-Zain & Abdulsalam, 2022	sample: no PhD	Liu et al., 2023	insufficient response
Alageel, 2024	no response	Liu et al., 2024	no response
Alalalmeh et al., 2024	sample: undergraduate students	Liu, 2018	no response
Alamu et al., 2023	wrong focus	Llosa et al., 2024	no response
Alberts et al., 2014	no primary research	Loissel E., 2019	no primary research
Aleksic et al., 2024	sample	Lonka et al., 2019	insufficient response
Alexander et al., 2024	no response	Lorenzetti et al., 2020	qualitative methodology
Ryan & Hulac, 2025	no response	Lourenco et al., 2022	sample: undergraduate students
Alkhatib, 2020	sample	Lovitts, 2001	no primary research
Allen et al., 2020	mail delivery failed	Lufler & McNulty, 2022	sample
Allen et al., 2021	mail delivery failed	Lynn et al., 2018	no data
Amalu et al., 2023	no response	Lyoo et al., 2014	no response
Amanvermez et al., 2024	sample: BA/MA	Ma et al., 2024	no response
Amber Mosley, 2022	sample	Macalli et al., 2021	no response
Amiri et al., 2025	outcome	Macaulay, Michael, 2003	mail delivery failed
Anderson et al., 2007	outcome	Machado et al., 2020	no response
Andrade et al., 2024	insufficient response	Machado et al., 2024	sample: undergraduate students
Anewr et al., 2021	no response	Macrynikola et al., 2018	sample: no PhD
Angstmann et al., 2022	not available	Madhan et al., 2011	outcome
Antoniadou et al., 2023	sample	Madhan et al., 2012	sample: Master of Dental Surgery (M.D.S.) in Orthodontics and Dentofacial Orthopedics
Appel et al., 2003	no mail found	Mairte et al., 2023	sample: no differentiation between MA & PhD
Appelseth et al., 2023	no response	Majeed et al., 2022	sample: post-graduate residents
Arafat et al., 2022	sample: no PhD	Malaska et al., 2022	sample
Aranda Beltran et al., 2003	not available	Malik et al., 2023	no response
Aria et al., 2024	sample	Maranda & Yakubovich, 2020	mail delivery failed
Armstrong-Carter et al., 2022	sample: no differentiation between MA & PhD	Marelli et al., 2020	no response
Arnold, 2014	no primary research	Martow et al., 2024	no response
Arora et al., 2016	sample: MBBS, MD/MS	Martínez-Nicolás et al., 2022	no response
Arsandaux et al., 2021	sample: no differentiation between MA & PhD	Mason et al., 2023	outcome
Arvandi et al., 2016	sample	Matthew Carter, 2024	no response
Atiyeh et al., 2023	wrong focus	Mazurek Melnyk et al., 2016	no response
Attia & Shata, 2013	no response	McAlpine et al., 2018	outcome
Aubin et al., 2025	sample	McChesney et al., 2024	wrong focus
Bach, Sarah Christine, 2017	outcome	McDowell et al., 2014	wrong focus
Bager-Charleson et al., 2022	qualitative methodology	McFarland et al., 2010	wrong focus
Baker & Chambers, 2011	insufficient response	McGee et al., 2019	wrong focus
Balling et al., 2021	sample: no differentiation between MA & PhD	McKenzie et al., 2024	no primary research (HMS)
Barredo et al., 2023	wrong focus	Medina et al., 2023	sample: undergraduates

Barredo et al., 2023	wrong focus	Meetei et al., 2019	not available
Barreira & Bolotonyy, 2024	no primary research	Meghani & Harvey, 2016	no response
Barreto et al., 2022	no response	Messina et al., 2010	sample: students of physiotherapy
Barrile et al., 2023	outcome	Michałowska et al., 2022	no response
Barry et al., 2019	duplicate data	Micheli, Amelia C., 2014	no mail found
Barton & Bulmer, 2017	no primary research (HMS)	Milicev et al., 2021	sample: no differentiation between MA & PhD
Bathelemy & Knaub, 2020	wrong focus	Miller & Orsillo, 2022	duplicate data
Batistoni et al., 2011	no response	Mirabelli et al., 2023	qualitative methodology
Bauer, 2016	mail delivery failed	Mohebbi et al., 2017	no response
Beattie, 2017	qualitative methodology	Mondal et al., 2021	wrong focus
Becerra et al., 2021	no response	Mongrain & Blackburn, 2005	no data
Beck et al., 2021	no response	Mongrain & Leather, 2006	no response
Begun et al., 2017	no response	Monzonís-Carda et al., 2021	sample: adolescents
Benesek, 1998	no data	Morganti et al., 2022	mail delivery failed
Benghasheer & Saub, 2020	mail delivery failed	Morin et al., 2022	wrong focus
Bergheul et al., 2022	no response	Moser et al., 2024	sample: undergrad
Bergin & Pakenham, 2015	sample	Moss & Mahmoudi, 2021	wrong focus
Bergvall et al., 2025	outcome	Moss et al., 2022	no response
Bergvall et al., 2024	wrong focus; but use for discussion	Motevalli et al., 2022	sample: undergraduate students
Bernanke et al., 2017	sample: no differentiation	Motseke & Masilonyana, 2016	wrong focus
Bernstein et al., 2021	no response	Munz, 2022	no response
Bernstein et al., 2023	no response	Musa et al., 2020	no response
Berry et al., 2020	qualitative methodology	Mushtaq et al., 2024	no response
Berry et al., 2021	no primary research (U-DOC)	Nadvi et al., 2024	no response
Berry et al., 2021	no primary research (U-DOC)	Nakano et al., 2024	sample: MA
Berry et al., 2023	no primary research (U-DOC)	Narita et al., 2022	no primary research (HMS)
Best, 2009	wrong focus	Nasr et al., 2024	insufficient response
Bhad et al., 2023	wrong focus	Nath & Pradhan, 2012	no response
Bhatta et al., 2018	no response	National Research Council, 2011	wrong focus
Bilodeau et al., 2021	no primary research (COVID-19 International Student Well-being Study)	National Union of Students, 2013	not available
Birmachu et al., 2021	sample	Negash et al., 2021	no primary research (COVID-19 International Student Well-being Study
Bittar et al., 2022	outcome	Negi et al., 2019	no response
Bogardus et al., 2022	mail delivery failed	Neher T, Ryan K, Thapa P, Porter A 3rd., 2025	no response
Bojanic, 2014	wrong focus	Nelson et al., 2008	outcome
Bolliger et al., 2012	outcome	Nelson et al., 2009	sample: no differentiation
Bonetto et al., 2024	wrong focus	Nelson, 2021	not available
Boone et al., 2022	no data	Nelson, Desarae E., 2021	sample
Bork & Mondisa, 2019	no primary research (HMS)	Neu T, Rode J, Hammer S., 2024	no response
Bork & Mondisa, 2020	no primary research (HMS)	Nguyen et al., 2022	no response
Bork & Mondisa, 2021	duplicate data	Ni Gao et al., 2024	no data
Bork et al., 2022	no primary research (HMS)	Noda et al., 2021	sample: only MA
Bosanquet et al., 2020	wrong focus	Nodine et al., 2021	wrong focus
Bou-Hamad et al., 2023	no response	Nodoushan et al., 2021	sample
Bouchard & Gallant, 2024	sample: MA PsyD	Nogueira-Martins et al., 2004	no response
Bozeman & Gaughan, 2011	sample: University professors	Nori et al., 2020	wrong focus
Bradley et al., 2021	sample: no PhD	Nottage et al., 2022	sample: no PhD
Branquinho et al., 2022	outcome	O'Connor et al., 2024	no primary research
Broc et al., 2020	no response	O'Connor et al., 2024	no primary research
Broglia et al., 2019	no response	Obradovic et al., 2024	wrong focus

British Columbia Council on Admissions and Transfer, 2020	not available	Oddone Paolucci et al., 2021	wrong focus
Brooker et al., 2020	no response	Oducado et al., 2021	wrong focus
Brouwer et al., 2021	sample: no differeniation between MA & PhD	Oducado et al., 2021	outcome
Brown et al., 2016	sample: Master of Science in Nursing	Ogilvie et al., 2020	sample: no differentiation between MA & PhD
Brown et al., 2022	no response	Ogunsanya et al., 2018	no response
Browning et al., 2021	sample: no differeniation between MA & PhD	Oh et al., 2021	no primary research
Browning & Brandon Rex, 2012	no response	Oh et al., 2024	no primary research (HMS)
Brownson et al., 2011	sample: no differentiation between MA/PhD	Ohayon & Roberts, 2021	no response
Brownson et al., 2016	wrong focus	Okoro et al., 2022	no primary research
Brubaker & Beverly, 2020	sample	Okoth, 2023	sample: undergraduate students
Bulut & Tuncay, 2020	no response	Orer, 2020	wrong focus
Burke et al., 2021	no response	Orozco, 2014	no response
Burns et al., 2023	wrong focus	Ostermiller et al., 2024	no primary research (NCHA)
Burton Denmark et al., 2012	mail delivery failed	Oswalt et al., 2018	sample: undergrad
Butler & Eartha, 2017	outcome	Oswalt et al., 2007	sample
Byrom & Metcalfe, 2020	no primary research	Paldanius et al., 2020	sample: undergraduate or Master's
Byrom, 2020	duplicate data	Pallos et al., 2004	mail delivery failed
Cader et al., 2021	not available	Pallos et al., 2005	mail delivery failed
Campbell et al., 2015	no response	Pallos et al., 2007	mail delivery failed
Can et al., 2021	no data	Panahi et al., 2016	no response
Cantoni et al., 2024	sample: MA	Pape et al., 2024	sample: no Phd
Cao et al., 2024	no response	Parakash et al., 2023	sample: no differentiation between MA & PhD
Caravaca-Sánchez & Muyor-Rodríguez, 2022	no response	Parke et al., 2022	no response
Cardilini et al., 2022	wrong focus	Parker et al., 2005	no mail found
Carrasco, 2022	no primary research (HMS)	Parpottas et al., 2024	wrong focus
Carson et al., 2025	no response	Pashayan et al., 2023	wrong focus
Casey et al., 2022	no response	Pasic et al., 2020	no primary research
Casey et al., 2023	no primary research	Patsali et al., 2020	sample: no differentiation between MA & PhD
Casey et al., 2024	no response	Peltonen et al., 2016	outcome
Liardet, 2024	methodology	Peluso, 2011	no data
Celikkol Sadic et al., 2024	no response	Peng & Zhang, 2004	not available
Chakraverty, 2020	wrong focus	Peng et al., 2022	no response
Chavez, NW; Srikant, D, 2025	outcome	Peng et al., 2024	sample
Chen et al., 2014	no response	Perlus, Jessamyn G., 2019	outcome
Chen et al., 2021	no response	Pertiwi et al., 2023	sample
Chi et al., 2021	no response	Petersen et al., 2012	wrong focus
Chu et al., 2024	no response	Peynenburg et al., 2020	no response
Chu, 2010	no response	Pfau et al., 2024	sample: undergraduate students
Cislo et al., 2021	wrong focus	Pham & Lui, 2020	sample: no differentiation between MA & PhD
Clark et al., 2012	no response	Phillips & Corocran, 2024	sample: nursing doctoral students (doctor of nursing practice)
Clément et al., 2024	no response	Picardi et al., 2005	sample: no differentiation between MA/PhD
Coakley et al., 2021	sample: no differentiation between PhD/MA	Pinzón et al., 2020	insufficient response
Coffino et al., 2021	mail delivery failed	Pinzón et al., 2024	insufficient response
Cole, 2008	outcome	Pizunska et al., 2021	insufficient response
Comparcini et al., 2016	wrong focus	Pokowitz et al., 2024	no response
Concerto et al., 2017	sample: first year, podiatry students	Polinghorne et al., 2023	no primary research
Cornér et al., 2021	no data	Poodineh et al., 2016	no mail found
Corral-Frías et al., 2019	insufficient response	Pourghaznein et al., 2022	wrong focus
Corvino et al., 2022	wrong focus	Powell et al., 2021	sample: PharmD

Costa-Cordella et al., 2022	sample: idk	Prabhat et al., 2022	outcome
Cotterall, 2013	qualitative methodology	Prado et al., 2022	no response
Coughenour et al., 2021	sample: no differentiation	Prakash et al., 2023	sample: no differentiation between MA & PhD
Covington et al., 2022	outcome	Prakash et al., 2024	sample: no differentiation
Cowie et al., 2018	no response	Prasad & Prasad, 2021	wrong focus
Creaton & Gower, 2024	no primary research	Priestley, 2023	no response
Creaton, 2021	no primary research	Prieto et al., 2022	wrong focus
Cromley et al., 2022	insufficient response	Prieto et al., 2023	wrong focus
Cromley et al., 2023	wrong focus	Prieto et al., 2023	wrong focus
Cromley et al., 2023	insufficient response	Prieto-Vila et al., 2024	duplicate data
Crone et al., 2023	wrong focus	Punia et al., 2021	wrong focus
Crook et al., 2021	no response	Pychyl & Little, 1998	no response
Crosmer, 2009	sample	Pychyl, 1995	no mail found
Cullinan et al., 2024	duplicate data	Pyhältö et al., 2012	outcome
Daniel et al., 2022	no response	Pyhältö et al., 2017	duplicate data
Danowitz & Beddoes, 2018	sample: 96% of respondents are undergraduates, while the remaining 4% are from Master's programs	Pyhältö et al., 2023	duplicate data
Das et al., 2024	no response	Qin et al., 2023	mail delivery failed
DeVylder et al., 2021	no primary research (HMS)	Qiu et al., 2024	no response
Delgado et al., 2024	wrong focus	Quarterman & Jerome, 2008	not available
Demir et al., 2023	sample	Quick et al., 2018	wrong focus
Deng et al., 2022	no response	Rabkow et al., 2020	sample
Mohamed & George, 2025	sample	Rad et al., 2014	wrong focus
Derwahl et al., 2022	insufficient response	Ramiz et al., 2021	sample
Deshpande et al., 2023	no response	Ramona Palo?, 2024	no response
Deznabi et al., 2021	no response	Ramón-Arbués et al., 2020	sample
Di Pierro, 2017	wrong focus	Rasouli et al., 2022	wrong focus
Dietz et al., 2023	no response	Rawlings et al., 2022	sample: no differentiation between MA/PhD
Divaris et al., 2012	sample: dental residents	Ray et al., 2019	insufficient response
Dompe, 2011	no mail found	Rayani et al., 2024	wrong focus
Dong et al., 2022	no mail found	Reddy, 2020	outcome
Dong et al., 2024	not available	Reevy & Deason, 2014	no response
Dong et al., 2024	no response	Reminick, Dominique, 2023	no mail found
Dong et al., 2024	insufficient response	Richardson et al., 2020	sample
Downs & Eisenberg, 2012	mail delivery failed	Rico & Bunge, 2021	insufficient response
Doyle et al., 2021	qualitative methodology	Rigler et al., 2017	no primary research
Drake, 2010	no response	Riley & Mallouk, 2023	qualitative methodology
Drum et al., 2009	not available	Rind & Arain, 2023	wrong focus
Drum et al., 2017	sample: no differentiation between MA and PhD	Rind et al., 2023	insufficient response
Du et al., 2020	sample: no differentiation between MA & PhD	Rocha-singh, 1994	no mail found
Du et al., 2021	sample: no differentiation	Rodriguez Sanchez & Laguna Martínez, 2023	sample: undergraduate students
Duan et al., 2022	no response	Rodriguez, 2023	sample: postgraduate professionals
Duffy et al., 2021	no primary research	Rogers et al., 2014	sample
Duffy et al., 2024	sample: no differentiation between MA & PhD	Rogers-Shaw et al., 2018	qualitative methodology
Duvall, 2008	no primary research	Rompf et al., 1993	not available
Ebrahimi et al., 2017	sample: MA program	Rood et al., 1994	no data
Eisenberg et al., 2007	no primary research (HMS)	Rosa-Rodríguez et al., 2015	insufficient response
Eisenberg et al., 2009	no primary research (HMS)	Rosenthal et al., 2021	no response
El-Ghoroury et al., 2012	wrong focus	Rosenthal et al., 2008	no response
Elliot Grace, 2024	no mail found	Rowe, 2022	no mail found
Elliot et al., 2024	qualitative methodology	Ruffalo Noel Levitz, 2022	not available
Ellison et al., 2020	sample	Rummell, 2015	no response
		Rönkökknen et al., 2023	wrong focus
Ely et al., 2022	no response	Sahu et al., 2019	sample: BA/ MA
Frank, 2023	no response	Sahu et al., 2020	sample: no PhD

Enav et al., 2024	sample: BA / MA	Sajadi et al., 2021	no response
Enns et al., 2018	no response	Saleem et al., 2022	no response
Eranti et al., 2025	wrong focus	Salzer, 2012	wrong focus
Espiritu et al., 2024	sample: professional students	Sandberg et al., 2022	sample: undergraduate
Esplin et al., 2025	sample	Santana, Lizette, 2017	mail delivery failed
European Students' Union, 2022	outcome	Santos Alves Peixoto et al., 2022	no response
Evans et al., 2017	not available	Santos et al., 2017	no response
Evans et al., 2018	no response	Sarah et al., 2021	wrong focus
Facundes & Ludermir, 2005	sample	Sato et al., 2020	no response
Samawi & Samawi, 2025	no response	Saura et al., 2011	no response
Falvey et al., 2021	no primary research (HMS)	Sayis & Gunes, 2024	insufficient response
Fan et al., 2024	no response	Schad et al., 2021	duplicate data
Fang et al., 2019	sample (master students)	Schiff et al., 2023	no response
Faramarzi & Khafri, 2017	no response	Schmickler et al., 2023	sample: graduate students in Germany
Faro, 2013	mail delivery failed	Schulte et al., 2008	no response
Senyametor et al., 2022	sample	Schultz, Katherine, 2023	sample
Fernanda et al., 2021	sample: pediatric dentists and postgraduate pediatric dentistry students	Scorsolini-Comin et al., 2021	no response
Fernández-Rodríguez et al., 2023	no response	Scott et al., 2020	no primary research
Filippou & Jokila, 2024	sample	Scott et al., 2024	no response
Fisher et al., 2019	no data	Scrubb, 1997	outcome
Forquer et al., 2008	mail delivery failed	Seay et al., 2008	wrong focus
Fowler et al., 2024	no primary research (NCHA-III)	Seckin & Varol, 2022	wrong focus
Francis et al., 2020	sample: no PhD	Selvaraj, 2016	not available
Fried et al., 2019	sample: no differentiation	Serras et al., 2010	mail delivery failed
Fried et al., 2022	no response	Shadowen et al., 2019	no response
Friedensen et al., 2024	qualitative methodology	Shafaei et al., 2018	no response
Fu et al., 2021	no response	Shanachilubwa et al., 2023	wrong focus
Fynn & van der Walt, 2023	no response	Shao et al., 2021	no response
Gafforelli et al., 2020	no response	Sharma et al., 2023	no response
Gandhi et al., 2024	no response	Shearin & Brewer-Mixon, 2020	no response
Gao et al., 2016	not available	Shekhar, 2023	wrong focus
Gao et al., 2024b	no response	Shelby N. Wilson, 2024	no data
Garcia-Williams et al., 2014	no response	Sheldon, 1994	no mail found
García-Salido et al., 2025	sample	Sherman et al., 2023	wrong focus
Gardner, 2013	no primary research	Shete & Garkal, 2015	no response
Garg & Bhardwaj, 2024	wrong focus	Shetter et al., 2017	sample: dental or medical
Garvick et al., 2022	no response	Shi et al., 2023	no response
Gasman et al., 2008	qualitative methodology	Shillington et al., 2024	no data
Gater, Cheryl L., 2019	outcome	Sifri et al., 2022	wrong focus
Gee et al., 2022	no primary research	Sivertsen et al., 2021-2023	wrong focus
Geiger et al., 2024	sample	Sivertsen et al., 2023	no response
Gewin V., 2025	no primary research	Smiljanic, Iskra, 2017	no data
Ghandour et al., 2013	no mail found	Smith et al., 2015	no response
Ghandour et al., 2020	wrong focus	Smith et al., 2024	no data
Ghogare et al., 2021	sample	Snellgrove & Gatling, 2024	sample
Giancristofaro et al., 2009	wrong focus	Solms et al., 2024	wrong focus
Girardi A., 2025	no response	Song & Yang, 2023	qualitative methodology
Girmay, Mehrete, 2017	not available	Sonmez LO, Gul M., 2021	sample
Gogoi, 2016	no mail found	Sonneville & Lipson, 2018	no response
Golberstein et al., 2008	no primary research (HMS)	Soomro et al., 2019	mail delivery failed
Goldberg et al., 2019	wrong focus	Cunningham et al., 2023	sample
Gollust et al., 2008	no primary research (HMS)	Steinbaugh et al., 2025	sample
Gong et al., 2007	not available	Stevanovic et al., 2021	wrong focus
Gong et al., 2023	wrong sample	Stoa, 2022	sample
González-Betancor & Dorta-González, 2020	wrong focus	Stouder A, Ayars CL., 2024	sample
Goodboy et al., 2015	insufficient response	Stoykov et al., 2017	sample
Gooding et al., 2023	insufficient response	Stubb et al., 2012	duplicate data
Gowen et al., 2023	no response	Sturman & Mongrain, 2005	no response
Grant et al., 2022	no primary research	Sturman et al., 2006	no response

Greeson et al., 2015	sample	Su & Feng, 2018	not available
Grogan, 2021	mail delivery failed	Suart et al., 2021	wrong focus
Gross et al., 2024	insufficient response	Suchikova et al., 2024	duplicate data
Guidotti Breting et al., 2020	no response	Sun et al., 2022	sample: undergraduate college students
Guo et al., 2021	no response	Sunindijo & Kamardeen, 2020	sample: PhD or Master by Research excluded
Gust et al., 2021	no primary research	Sunny et al., 2020	qualitative methodology
Gérard & Nagels, 2017	mail delivery failed	Szkody et al., 2023	duplicate data
Gómez-Ospina & García-Perdomo, 2024	insufficient response	Takeuchi & Sakagami, 2022	no response
Gökalp, 2023	not available	Talapko et al., 2021	sample
Hagerty & Bathish, 2018	sample	Tan et al., 2024	sample
Hahn et al., 2021	mail delivery failed	Tancredi et al., 2022	no primary research (COVID-19 International Student Well-being Study
Halperin et al., 2010	no response	Hassem et al., 2024	sample
Han & Pistole, 2014	no response	Teixeira et al., 2021	sample: undergraduate students
Han & Wang, 2024	no response	Teng et al., 2024	no primary research
Han et al., 2013	no response	Thorley, 2017	no primary research
Harri et al., 2013	mail delivery failed	Tikkanen et al., 2024	sample: doctoral supervisors
Harrop et al., 2021	outcome	Toews et al., 1993	no response
Harvey et al., 2011	sample	Toews et al., 1997	no mail found
Hazell et al., 2021	no primary research (U-DOC)	Tontodimamma et al., 2024	insufficient response
Hazell et al., 2021	no primary research (U-DOC)	Torales et al., 2025	sample
Hazell et al., 2024	sample	Trivedi & Prakasha, 2021	wrong focus
He et al., 2025	no response	Troutman et al., 2022	not available
Helbing et al., 1998	no mail found	Tsai et al., 2016	no primary research
Helmers et al., 1997	mail delivery failed	Tsybuliak et al., 2024	duplicate data
Hermida, 2017	wrong focus	Turner et al., 2000	sample
Higher School of Economics (HSE University), Russia, 2020	no primary research	U-DOC, 2018-2019	no primary research
Hill & Lance, 2002	wrong focus	Ueno et al., 2024	no primary research (U-DOC)
Hill et al., 2022	no response	Underwood et al., 2024	sample: no PhD
Hill et al., 2023	no response	University of California Office of the President, 2017	insufficient response
Hirai et al., 2015	insufficient response	Uristemova et al., 2024	no response
Holahan, 1979	outcome	Usher & McCormack, 2021	wrong focus
Holtz et al., 2022	wrong focus	Usman et al., 2022	wrong focus
Homer et al., 2021	wrong focus	Vafaee-Najar et al., 2023	no response
Hong et al., 2020	sample	Valóta et al., 2023	wrong focus
Honnekeri et al., 2025	sample	Van Benthem et al., 2020	no primary research (CAPS)
Hooda & Choudhary, 2021	outcome	Van Wingerden, 2024	wrong focus
Hoque, 2024	wrong focus	Varadarajan et al., 2021	no response
Horiuchi et al., 2018	wrong focus	Vasugi & Hassan, 2019	not available
Hou et al., 2024	no response	Veilleux et al., 2012	wrong focus
Huang et al., 2022	no response	Ventura et al., 2008	no response
Huang et al., 2024	sample: college students, too young	Villatoro et al., 2023	sample
Humphreys, 2013	sample (masters' degrees of professional psychology)	Vincent et al., 2022	wrong focus
Hunter & Devine, 2016	wrong focus	Vineyard et al., 2021	sample
Hyeon Jean Yoo, 2021	no response	Volkert et al., 2018	wrong focus
Hysenbegasi et al., 2005	sample (undergraduate)	Vucic et al., 2024	sample: no differentiation between MA & PhD
Hyun et al., 2007	no mail found	Vuelvas-Olmos et al., 2023	no response
Ignacchiti et al., 2011	no response	Vuuren et al., 2021	sample: postgraduate accounting students
Iida et al., 2021	no response	Waaijer et al., 2016	wrong focus
Indartono et al., 2020	wrong focus	Walsh et al., 2022	wrong focus
Jabbari et al., 2023	no response	Wan et al., 2023	sample: no PhD
Jachim et al., 2023	no response	Wan et al., 2024	no response
Jackman et a., 2022	wrong focus	Wang & Huang, 2021	sample: no differentiation between MA & PhD
Preston et al., 2021	wrong focus	Wang & Wang, 2006	sample
Jacob et al., 2020	no response	Wang et al., 2025	sample
Jaiswal et al., 2025	sample	Wang et al., 2010	qualitative methodology

Jamaludin & You, 2019	sample: educators	Wang et al., 2017	no response
Jamshidian et al., 2014	not available	Wang et al., 2018	not available
Jankowski et al., 2013	sample: masters-level students	Wang et al., 2020	sample
Jansen et al., 2023	wrong focus	Wang et al., 2022	mail delivery failed
Jardon & Choi, 2024	no response	Wang et al., 2022	mail delivery failed
Jauniaux et al., 2017	wrong focus	Wang et al., 2022	mail delivery failed
Javier, 2022	no response	Wang et al., 2024	no response
Jelleli et al., 2024	no response	Wang et al., 2024	no response
Jenaabadi et al., 2017	not available	Wang, 2008	not available
Jensen et al., 2023	wrong focus	Wao, 2010	wrong focus
Jeong & Koh, 2021	no response	Wash et al., 2022	no response
Jia et al., 2022	sample: undergraduate students	Washington et al., 1990	not available
Jiang & Mazlan, 2024	sample: no doctoral students	Watson et al., 2014	mail delivery failed
Jiang et al., 2024	no response	Watson et al., 2024	wrong focus
Jingsha Zhou, 2020	no primary research	Weber et al., 2020	no response
Joeng et al., 2017	no data	Weber et al., 2022	no response
Johnson, 2015	sample	White et al., 2006	sample can't be differentiated
Jones-White et al., 2022	insufficient response	White et al., 2022	wrong focus
Pinzon et al., 2024	no response	White et al., 2022	wrong focus
Jung, 2019	outcome	Whitlock et al., 2006	not available
Juniper et al., 2012	wrong focus	Whitwer et al., 2023	no primary research (HMS)
Justice, 2017	sample: PsyD or MA	Wichmann-Hansen et al., 2023	no data
Júnior et al., 2024	wrong focus	Wijesekara, 2022	sample: no differentiation
Kahn et al., 2021	no response	Wijesekara, 2022	sample: no differentiation
Kalenjuk et al., 2024	no primary research	Wildey et al., 2022	sample
Kaler et al., 2021	not available	Wilkinson et al., 2014	no response
Kamardeen & Sunindijo, 2018	sample: no PhD	Willgens & Palombaro, 2019	mail delivery failed
Kandlur et al., 2024	sample: no differentiation	Williams-York et al., 2024	sample
Kanters et al., 2002	sample: freshmen from classes for a Doctor of Veterinary Medicine	Winter et al., 2021	no response
Kao et al., 2014	no data	Wolfe et al., 2024	no response
Karagiorgakis & Blaker, 2021	not available	Wolniewicz, Rebecca, 1996	outcome
Karstens & Whitesell, 2022	no primary research	Woloshuchuk et al., 2022	wrong focus
Forbes-McKay & Henderson, 2025	no response	Womack et al., 2020	outcome
Bignold & Budd, 2024	sample	Woolston, 2017	outcome
Kaufman, 2006	duplicate data	Woolston, 2018	not available
Kaura & Lodhwal, 2024	not available	Woolston, 2019	no primary research
Kawaida et al., 2021	no response	Woolston, 2019	outcome
Keating SR, Fairbanks C., 2025	no response	Woolston, C, 2020	outcome
Kenty, 2000	wrong focus	Wu et al., 2022	mail delivery failed
Kernan et al., 2011	not available	Wu et al., 2022	sample: no differentation between MA & PhD
Keszei et al., 2020	wrong focus	Wu et al., 2024	no response
Kewallal, 2013	no response	Wyatt & Oswalt, 2013	sample: no differentiation between MA/ PhD
Khadakkar & van Nouhuys, 2024	wrong focus	Xiao et al., 2020	sample
Khan & Parveen, 2020	not available	Xie et al., 2019	no mail found
Khan, 2021	wrong focus	Xu et al., 2020	sample: BA/MA
Khodabakhsh et al., 2024	wrong focus	Xu et al., 2018	no response
Kim et al., 2022	no response	Yacoby et al., 2023	wrong focus
Kim et al., 2023	wrong focus	Yadav et al., 2021	no response
Kinman, 2001	methodology	Yamasaki et al., 2009	no response
Kinouani et al.., 2024	no response	Yang et al., 2018	mail delivery failed
Kishita & Shimanda, 2011	sample	Yao et al., 2023	sample
Klein et al., 2011	no response	Yao et al., 2023	no response
Klinefelter et al., 2022	sample	Yary & Azami, 2011	no data
Koebeli, 2023	sample: undergraduate psychology students	Yazici Gülec et al., 2012	no response
Kortoglu et al., 2021	no response	Yeh et al., 2023	sample
Krause & Harris, 2019	no primary research	Yin, 2021	mail delivery failed
Kuchyn et al., 2024	wrong focus	Yoo & Marshall, 2024	no response
Kumar et al., 2022	wrong focus	Yoosefi Lebni et al., 2023	no response

Kumar, 2023	no response	YouGov, 2016	wrong focus
Kundu et al., 2021	sample: MA	Yuan et al., 2024	sample: no differentiation between MA & PhD
Kundu et al., 2022	sample: no PhD	Yüce Esen et al., 2021	wrong focus
Kunz et al., 2024	wrong focus	Zahedi et al., 2024	sample
LaBelle et al., 2024	qualitative methodology	Zahniser et al., 2017	no response
Labbaf et al., 2024	sample: no information available in regard to whether doctoral students were included	Zancu et al., 2022	no response
Laethem et al., 2017	outcome	Zeng et al., 2018	no response
Lamberti, 2024	no primary research	Zhang & Duan, 2023	wrong focus
Lambrechts & Smith, 2020	wrong focus	Zhang & Ma, 2023	not available
Lange et al., 2023	wrong focus	Zhang et al., 2020	outcome
Langin, 2019	wrong focus	Zhang et al., 2022	no response
Lanier et al., 2001	not available	Zhang et al., 2022	no response
Larcombe et al., 2014	sample (undergraduate and master students or Juris Doctor)	Zhang et al., 2024	wrong focus
Latha & Karalam, 2021	no response	Zhao et al., 2021	no primary research
Lazarevic et al., 2016	sample: no differentiation between MA & PhD	Zhao et al., 2021	wrong focus
Leanna Billings, 2022	sample	Zhao et al., 2022	wrong focus
Lee & Jeong, 2014	no response	Zhen et al., 2024	wrong focus
Lee et al. 2024	wrong focus	Zheng L, Deng W., 2025	outcome
Lee & Kim, 2019	sample	Zhong et al., 2024	no response
Lee et al., 2021	wrong focus	Zhou et al., 2018	no mail found
Lee et al., 2024	wrong focus	Zhou et al., 2018	not available
Lemos-Santos et al., 2023	no response	Zhou et al., 2021	no response
Leonelli et al., 2024	no response	Zhou et al., 2021	no response
Leshner, 2015	no primary research	Zickgraf et al., 2022	no primary research (HMS)
Li et al., 2021	no response	Zile, 2023	wrong focus
Li et al., 2022	wrong focus	da Silva et al., 2022	no response
Li et al., 2023	sample: professional degree graduate students	das Chagas Valóta et al., 2023	wrong focus
Lian & Wallace, 2020	wrong focus	de Alexandre et al., 2021	wrong focus
Lian et al., 2020	wrong focus	de Moura et al., 2023	mail delivery failed
Liang et al., 2021	no response	de Oliveira & Meireles, 2024	no primary research
Liang et al., 2022	no response	de Oliveira et al., 2022	mail delivery failed
Liang et al., 2024	no response	nature, 2020	no primary research
Liao & Wei, 2014	mail delivery failed	van Berkel et al., 2017	sample
Lightstone et al., 2006	sample	van der Velde et al., 2021	no primary research
Lilly et al., 2018	not available	Ülkü-Steiner et al., 2000	wrong focus
Flavius et al., 2018	no mail found	Şahin et al., 2024	no response
Limas et al., 2022	no primary research	金红昊, 杨钋, 2021	no response
Lin et al., 2022	no response	Unknown authorship, 2024	sample: no differentiation

Note. This table includes all studies excluded after inspection of either full text, after having attempted contacting the researchers or unavailability of the paper. This table does not include studies excluded based on title- or abstract-screening or simply duplicates. More information per study and exclusion can be found on the OSF database, specifically the file "Literature screening". Several categories of exclusion exist, such as duplicate data, namely the data was already used in another published study. A separate category 'no primary research' was created for studies that used data from greater research databases, e.g. the Healthy Minds Network. If a study was conducted using a methodology that could not be integrated in the analysis, they received the exclusion reason 'qualitative methodology' or 'methodology'. 'Wrong focus' reflects the fact that the study did not collect data on the main outcome variables of this study, namely prevalence or severity of symptom domains and syndromes. Similarly, the label 'wrong outcome' reflects the usage of scales incompatible with the scales assessed in this study. If a contact attempt was not possible, it was either categorized as 'no mail found', 'mail delivery failed' or 'no response'. In case the response was insufficient, e.g., authors of the study did reply at first, could or did not provide sufficient data for the study to have been included in the analysis, the study received the label 'insufficient response'. If data was not available in text and also not after a contact attempt it received the exclusion reason 'no data'.
