# Supplementary material for: Prevalence and severity of mental health problems in early-career researchers: a systematic review and meta-analysis
Source: Nat Hum Behav. 2026 Jun 29;10(9):1698–717. doi: 10.1038/s41562-026-02505-5 (PMC13590413; doi:10.1038/s41562-026-02505-5)
Supplement: Supplementary file 2 [file 41562_2026_2505_MOESM2_ESM.pdf]

nature portfolio

Corresponding author(s): Aljoscha Dreisoerner, Urs M. Nater

Last updated by author(s): Apr 28, 2026

Reporting Summary

Nature Portfolio wishes to improve the reproducibility of the work that we publish. This form provides structure for consistency and transparency in reporting. For further information on Nature Portfolio policies, see our Editorial Policies and the Editorial Policy Checklist.

Statistics

For all statistical analyses, confirm that the following items are present in the figure legend, table legend, main text, or Methods section.

n/a	Confirmed	
☐	☒	The exact sample size (*n*) for each experimental group/condition, given as a discrete number and unit of measurement
☐	☒	A statement on whether measurements were taken from distinct samples or whether the same sample was measured repeatedly
☐	☒	The statistical test(s) used AND whether they are one- or two-sided<br>*Only common tests should be described solely by name; describe more complex techniques in the Methods section.*
☐	☒	A description of all covariates tested
☐	☒	A description of any assumptions or corrections, such as tests of normality and adjustment for multiple comparisons
☐	☒	A full description of the statistical parameters including central tendency (e.g. means) or other basic estimates (e.g. regression coefficient) AND variation (e.g. standard deviation) or associated estimates of uncertainty (e.g. confidence intervals)
☐	☒	For null hypothesis testing, the test statistic (e.g. *F*, *t*, *r*) with confidence intervals, effect sizes, degrees of freedom and *P* value noted<br>*Give P values as exact values whenever suitable.*
☒	☐	For Bayesian analysis, information on the choice of priors and Markov chain Monte Carlo settings
☐	☒	For hierarchical and complex designs, identification of the appropriate level for tests and full reporting of outcomes
☐	☒	Estimates of effect sizes (e.g. Cohen's *d*, Pearson's *r*), indicating how they were calculated

Our web collection on statistics for biologists contains articles on many of the points above.

Software and code

Policy information about availability of computer code

Data collection	Data was collected through publically available literature databases Scopus, ERIC, Web of Science, and PubMed, as well as from authors of primary studies. Data was collected in Microsoft Excel.
Data analysis	Data was analyzed in R (v4.5.2) and RStudio (2026.01.2+418), using the packages metafor(4.8-0), tidyverse(2.0.0), readxl(1.4.5), metaforest(0.1.5), ggplot2(4.0.0), randomForest(4.7-1.2), plyr(1.8.9), dplyr(1.1.4),gridExtra(2.3), metaSEM(1.5.0), ggrepel(0.9.6), caret(7.0-1), pwr(1.3-0), fastDummies(1.7.5). Prevalence and severity estimates were collected from primary studies through effect size transformations that are explained in detail in the manuscript. Data and analysis code are fully available at the following link: https://osf.io/r9nkd/.

For manuscripts utilizing custom algorithms or software that are central to the research but not yet described in published literature, software must be made available to editors and reviewers. We strongly encourage code deposition in a community repository (e.g. GitHub). See the Nature Portfolio guidelines for submitting code & software for further information.

Data

Policy information about availability of data

All manuscripts must include a data availability statement. This statement should provide the following information, where applicable:

- Accession codes, unique identifiers, or web links for publicly available datasets
- A description of any restrictions on data availability
- For clinical datasets or third party data, please ensure that the statement adheres to our policy

Data are publicly available in the Open Science Framework under the following link: https://osf.io/r9nkd/. Data was collected through the databases Scopus, ERIC, Web of Science, and PubMed, as well as email communication with authors of primary studies. There are no restrictions regarding data availability.

Research involving human participants, their data, or biological material

Policy information about studies with human participants or human data. See also policy information about sex, gender (identity/presentation), and sexual orientation and race, ethnicity and racism.

Reporting on sex and gender	Gender was used as the relevant population characteristic because the primary studies asked participants to report the gender with which they most identified.
Reporting on race, ethnicity, or other socially relevant groupings	Ethnicity, when reported in the included studies, was coded into predefined categories (Middle Eastern, Latin, Caucasian, Asian, Black, Other) for descriptive and moderator analyses. It was treated as a socially relevant sample characteristic and not as a proxy for other variables.
Population characteristics	Population characteristics included age, gender, ethnicity, academic position/career stage (predoctoral vs postdoctoral), academic discipline, country/region, and prior mental health diagnosis where reported. Additional sample-level characteristics examined as moderators included social support, supervisor relationship quality, job satisfaction, life satisfaction, and loneliness.
Recruitment	No recruitment was part of the study.
Ethics oversight	The current meta-analysis did not require ethical approval.

Note that full information on the approval of the study protocol must also be provided in the manuscript.

Field-specific reporting

Please select the one below that is the best fit for your research. If you are not sure, read the appropriate sections before making your selection.

☐ Life sciences ☒ Behavioural & social sciences ☐ Ecological, evolutionary & environmental sciences

For a reference copy of the document with all sections, see nature.com/documents/nr-reporting-summary-flat.pdf

Behavioural & social sciences study design

All studies must disclose on these points even when the disclosure is negative.

Study description	The present study is a three-level meta-analysis and systematic review of the prevalence and severity of mental health problems among early-career researchers, and of the sample- and study-level factors associated with these prevalence and severity estimates.
Research sample	The sample consisted 228 samples, drawn from 148 primary studies and 4 primary databases, resulting in a final sample of 138,446 early-career researchers (134,572 doctoral, 3,522 postdoctoral; pre- or postdoctoral status was not reported for 352 participants). Participants were drawn from a wide range of academic disciplines, were 63.3% female, and had a mean age of 30.5 years (SD = 4.3). The sample was diverse, but the current evidence still includes a disproportionate amount of data drawn from Western samples. Detailed demographic information is provided in the manuscript and Supplementary Information. As this study is a meta-analysis, data were drawn from previously published studies. The primary studies included in the present meta-analysis were identified through searches of the publicly available databases Scopus, ERIC, Web of Science, and PubMed, with additional data obtained from authors of primary studies via email communication.
Sampling strategy	Eligible studies were identified through systematic searches of Scopus, Web of Science, ERIC, and PubMed. The search string combined a broad set of terms for early-career researchers (e.g., doctoral students, graduate student, postdoc), various mental health syndromes and symptoms (e.g., mental illness, depression, stress, anxiety), and quantitative study designs (e.g., questionnaire, survey); the full search string is reported in the manuscript and Supplementary Information. Database searches were supplemented by backward and forward citation searching of relevant reviews and meta-analyses, and by email contact with authors where additional information was required.
Data collection	Data were extracted and coded in Excel spreadsheets. Researchers were not blinded to the study aims or hypotheses during data collection. Blinding to experimental condition does not apply, as no experimental conditions were used in this meta-analysis.

Timing	The databases were searched through October 28, 2025. Data extraction started on June 8, 2024, and was finished on December 1, 2025.
Data exclusions	Exclusion criteria were established a priori and are detailed in the preregistration and manuscript. The PRISMA flow diagram lists all exclusion reasons. Overall, 800 reports were excluded at the full-text stage.
Non-participation	We did not directly collect data from participants, but analyzed data from existing primary studies instead.
Randomization	Participants in the present meta-analysis were not allocated to different groups.

Reporting for specific materials, systems and methods

We require information from authors about some types of materials, experimental systems and methods used in many studies. Here, indicate whether each material, system or method listed is relevant to your study. If you are not sure if a list item applies to your research, read the appropriate section before selecting a response.

Materials & experimental systems

n/a	Involved in the study
☒	☐ Antibodies
☒	☐ Eukaryotic cell lines
☒	☐ Palaeontology and archaeology
☒	☐ Animals and other organisms
☒	☐ Clinical data
☒	☐ Dual use research of concern
☒	☐ Plants

Methods

n/a	Involved in the study
☒	☐ ChIP-seq
☒	☐ Flow cytometry
☒	☐ MRI-based neuroimaging

Plants

Seed stocks	*Report on the source of all seed stocks or other plant material used. If applicable, state the seed stock centre and catalogue number. If plant specimens were collected from the field, describe the collection location, date and sampling procedures.*
Novel plant genotypes	*Describe the methods by which all novel plant genotypes were produced. This includes those generated by transgenic approaches, gene editing, chemical/radiation-based mutagenesis and hybridization. For transgenic lines, describe the transformation method, the number of independent lines analyzed and the generation upon which experiments were performed. For gene-edited lines, describe the editor used, the endogenous sequence targeted for editing, the targeting guide RNA sequence (if applicable) and how the editor was applied.*
Authentication	*Describe any authentication procedures for each seed stock used or novel genotype generated. Describe any experiments used to assess the effect of a mutation and, where applicable, how potential secondary effects (e.g. second site T-DNA insertions, mosiacism, off-target gene editing) were examined.*
