# Supplementary material for: Prevalence and severity of mental health problems in early-career researchers: a systematic review and meta-analysis
Source: Nat Hum Behav. 2026 Jun 29;10(9):1698–717. doi: 10.1038/s41562-026-02505-5 (PMC13590413; doi:10.1038/s41562-026-02505-5)
Supplement: Supplementary file 3 [file 41562_2026_2505_MOESM3_ESM.pdf]

nature portfolio

Peer Review File

Prevalence and severity of mental health problems in early-career researchers: a systematic review and meta-analysis

Corresponding Author: Professor Urs M. Nater

Version 0:

Decision Letter:

21st October 2025

Dear Professor Nater,

Thank you once again for your manuscript, entitled "Prevalence and severity of psychological distress among early-career researchers: a systematic review and multilevel meta-analysis", and for your patience during the peer review process.

Your Article has now been evaluated by 3 referees. You will see from their comments copied below that, although they find your work of considerable potential interest, they have raised quite substantial concerns. In light of these comments, we cannot accept the manuscript for publication, but would be interested in considering a revised version if you are willing and able to fully address reviewer and editorial concerns.

We hope you will find the referees' comments useful as you decide how to proceed. If you wish to submit a substantially revised manuscript, please bear in mind that we will be reluctant to approach the referees again in the absence of major revisions. We are committed to providing a fair and constructive peer-review process. Do not hesitate to contact us if there are specific requests from the reviewers that you believe are technically impossible or unlikely to yield a meaningful outcome.

To guide the scope of the revisions, the editors discuss the referee reports in detail within the team, including with the chief editor, with a view to (1) identifying key priorities that should be addressed in revision and (2) overruling referee requests that are deemed beyond the scope of the current study. We hope that you will find the prioritised set of referee points to be useful when revising your study. Please do not hesitate to get in touch if you would like to discuss these issues further.

1. Reviewer 2 and 3 raise concerns about the search having been conducted using a single database only, despite the preregistration plan including the plan to search several databases. Following the feedback of the reviewers, we ask that you expand your search to at least one additional database. Please also update your flow diagram and transparently report all search strings.

2. Conduct targeted sensitivity and robustness checks as requested by Reviewer 2.

3. Following Reviewer 2’s feedback, please validate or demote the AI-based clustering.

4. Address all remaining concerns, except for Reviewer 3’s request to move the Methods section as this would violate Nature style and would need to be reformatted at a later stage.

Finally, your revised manuscript must comply fully with our editorial policies and formatting requirements. Failure to do so will result in your manuscript being returned to you, which will delay its consideration. To assist you in this process, I have attached a checklist that lists all of our requirements. If you have any questions about any of our policies or formatting, please don't hesitate to contact me.

If you wish to submit a suitably revised manuscript, we would hope to receive it within 4 months. I would be grateful if you could contact us as soon as possible if you foresee difficulties with meeting this target resubmission date.

With your revision, please:

• Include a “Response to the editors and reviewers” document detailing, point-by-point, how you addressed each editor and referee comment. If no action was taken to address a point, you must provide a compelling argument. When formatting this document, please respond to each reviewer comment individually, including the full text of the reviewer comment verbatim followed by your response to the individual point. This response will be used by the editors to evaluate your revision and sent back to the reviewers along with the revised manuscript.

• Highlight all changes made to your manuscript or provide us with a version that tracks changes.

Please use the link below to submit your revised manuscript and related files:

Link Redacted

Note: This URL links to your confidential home page and associated information about manuscripts you may have submitted, or that you are reviewing for us. If you wish to forward this email to co-authors, please delete the link to your homepage.

Thank you for the opportunity to review your work. Please do not hesitate to contact me if you have any questions or would like to discuss the required revisions further.

Sincerely,

[redacted]

[redacted]
Nature Human Behaviour

Reviewer expertise:

Reviewer #1: ECRs and mental health

Reviewer #2: ECRs and mental health

Reviewer #3: evidence-synthesis ; education

REVIEWER COMMENTS:

Reviewer #1 (Remarks to the Author):

Thank you for the opportunity to read this paper. What an enormous amount of work has gone into this. I believe your work here is a really powerful contribution to the sector, Providing much needed data-driven insights into what's going on for the mental health of our early career researchers.

I have a number of reflections on the manuscript. I hope you don't mind me walking through these in the order in which they appear. I must note, this is a huge piece of work to read, it is very possible that at points I have missed things, so your answer to some of my questions may simply be we've already said that in this place here. I do think though that there is work perhaps that can be done to help make your manuscript Easier to navigate and digest for Other readers. This work is really important and we need Audiences within And beyond the academic community to understand your findings

I have a long list of suggestions here, some of which are larger than others. I want to stress, that I am sharing these comma, in the attempt to help you improve you make this as accessible as possible manuscript. I think this is a really sound piece of work, that needs publication, my questions and critiques are around how you are presenting the work , And how you make this as accessible as possible

Abstract
Line 30 - here for the first time you use the word symptom. You do this at multiple points across the paper. I'm not sure when you're saying that an ECR meets the criteria for at least one mental health symptom, that you have ever used scales that measure a single symptom on their own, they might measure a cluster of symptoms. I wonder if you could look at this again.

Line 40- your brief summary here about the effect of age, doesn't tell us the direction.

Introduction.
1. in the introduction You talk about issues for ecr's and PhD students and you bring in doctoral attrition. Can we have much more clarity early on about any differentiation you see between what is going on for postdoctoral researchers doctoral researchers and pre doctoral researchers. I must note that I did not originally understand that you included doctoral researchers in your study. I think this could be clearer both in the introduction methods and result
2. Unit for individual studies suggest a significant proportion of doctoral students have completed suicide. Can you contextualise this for us, to look at the prevalence of suicide in Among doctoral students among among doctoral students In the context of the prevalence for age matched control
3. Your introduction told us a lot about what you were going to do, in terms of reviewing mental health issues beyond anxiety and depression. But in focusing on explaining what you were going to do, your argument for the rationale of why it was important was lost
4. You know there's a high Prevalence of psychological distress among a ECR's underscoring an urgent need. Please can you present this in the context of prevalence of mental distress among an age much cohort. Is this specific to ECR's?
5. Across the introduction in general I think the rationale for this study And how it's going to tell us something more and new than previous work is done, could be better communicated.
Methods
1. I've never understood why journals asked for these to be at the end of the manuscript. This isn't your problem but some people

would like to read the methods before they read the results. There are certain things that you don't explain in the methods because you have already explained in the results. I would encourage you to explain these including acronyms, in the method section for the reader who wants to understand what you did before they go on to read what you found
2. I'm interested that you manual search in Scopus will stop I haven't seen this done before, I wonder if you could say a bit more about why you pivoted to take this approach
3. In studyIn study selection an eligibility, can you tell us more about how many researchers were involved in the screening, and how any disagreements between researchers were resolved
4. You note that you included primary datasets, can you tell us a bit more about the availability of primary datasets, and the extent to which you were able to tap in to original data sets.
5. You note at the end of the section on data extraction that any inconsistencies were discussed and agreed upon. Can you tell us more about the scale of inconsistencies that you found. Perhaps you could say a little bit more about what gave you confidence that simply reviewing and checking into code of reliability in 30 articles was sufficient
6. In effect size measures, you introduce pomp scores. You don't explain what these are. Please could you do so here. As I read your results I could see that you had done something really sensitive and nuanced to the variation across the scales that you were working with. This sensitivity and care does not come across in the methods.
7. P-31 line 646, you say that PHQ 9 can only be between nought and one. I think there's a typo here. And you meant nought and 3.
8. On the same point, I have never seen an normally distributed PHQ 9 data set, The population is usually negatively skewed on this scale. So I wonder why you made the assumption that scores above 0 would be equally distributed.
9. In critical appraisal, line 688, I don't understand what you mean by a response rate. Did papers reliably tell you what the target population was and how large that was? How have you in interpreted a response rate if there is no recruitment sample specified?
10. Line 700 and line 705 are 2 examples of points where you're referencing seems strange. Sometimes the references are in numbers in brackets. And I don't understand how we interpret ref.
11. Line 735 the acronym FDR has not been defined
Results
1. Did your sample include any pre doctoral research assistance? Your introduction implied as much, but I don't see any mention in the results.
2. I would like to know more about how you defined academic disciplines, wrangling the different ways disciplines of their participants and how you have managed to cluster them into neat categories. One problem here that jumps out at me, is you note that psychology accounts for 32% of social sciences. In the UK psychology is defined as a medical science, and would be reported as part of a population group of Medical Sciences. I'm not sure I understand how you will have managed data across studies where for example they may have only reported the percentage of students studying Medical Sciences rather than the types of subjects within that categorisation. I can see this as being unbelievably complicated for you to have managed.
3. I think given the focus that you have on outcome measures later on in the results and then in the discussion, it would be helpful in your section on the characteristics of studies to give us a breakdown summary of the outcome measures used so common syndromes
4. I have to say, I'm sorry the section on pooled prevalence and severity estimates by syndromes and symptoms is really dense and hard to follow.
5. I haven't understood the rationale for why you focused in on different burnout scales why has this got more attention?
6. Figure 3 is fascinating. But complex. I think it could be really helpful to spend a bit of time in the text talking the reader through how to understand these graphs. Perhaps you could walk through an example with a POMP score
7. Line 289. Can you be specific about how you have classified the COVID-19., what were the years or months, that you used to cut off
8. You discuss the moderating effects of study design and quality before risk of bias. There is something disjointed in this as it would be helpful to understand the shape and nature of the data in terms of study quality before you tell us the impact of study quality on prevalence and severity estimates
9. I am afraid I really struggled with this section of your findings. You are telling us that study quality significantly moderates prevalence and severity estimates. Something actually that we knew from previous research, as you wove it into the introduction. And you are confirming that there is considerable variability in the study quality, of studies included in your meta analysis. This comes after a lot of analysis on the prevalence and severity, which has presumably used all of the available data. I can see from Table 1, That you have included study quality in your regression model. Can you confirm consistently across the results, that the findings you are reporting are off are after having controlled for study quality?
10. In table one, I don't see you including the country in which the study took part, or your clever clustering of countries. I wonder why? I would have thought there were important differences between types of country. i
Discussion
1. You are comparing your results of prevalence estimates to the general population. Estimates of prevalence of depression and anxiety in the general population, especially for young adults, vary wildly. To me 5 and 6% look incredibly low. I would be grateful if you would recognise the variability that exists in population level prevalence estimates, the difference between ecr's and the general population may be much smaller then you were suggesting
2. I wonder if across the manuscript in general, and in the discussion in particular, you could think about how and where you could avoid using acronyms. This is a really complex paper. The use of acronyms adds an unnecessary level of complexity. For example the use of the acronym Ed, and nSSI, In the discussion felt unnecessary
3. I don't feel your paper, your analysis, justifies the text in the paragraph starting 476 in the discussion. Your analysis hasn't really told us anything about what's actually going to help. So I feel this section is an overreach, beyond your findings, but also feels unnecessary.
4. line 501, can you tell us a bit more about the evidence that clinical diagnostic interviews lead to more reliable data then self reported rating scales. I'm not convinced they're better, just rather different.
5. Line 511, you note that many studies have not reported the exact sample composition, of whether their participants are pre doctoral ecr's or PhD students, this raised a question for me, of how have you actually managed this in the data? If it's not clear in the original papers, how have you differentiated between these groups?

I intentionally sign all my reviews,
Nicola Byrom

Reviewer #2 (Remarks to the Author):

Manuscript background information
This manuscript reports a preregistered systematic review and three-level meta-analysis of psychological distress in ECRs, covering 212 samples (N=128,684) through 31 Dec 2024. It estimates pooled prevalence for six outcomes and pooled severity (POMP) scores, examines multiple moderators, and discusses implications for institutional policy/support.

Remarks to the authors

1. Significance, originality, and fit for NHB
The topic of ECR mental health has clear societal and policy relevance, and a comprehensive, quantitatively rigorous synthesis is timely. The scale of aggregation and three-level modeling are strengths. With stronger robustness work and clearer communication around measurement/interpretation, this paper could be an impactful contribution for science and NHB's audience.

2. Study design and methods
(a) Search strategy & prereg deviations. The protocol lists "several databases," but the final search relied on Scopus plus backward/forward chaining. Please provide a quantitative coverage check (e.g., precision tests against numbers from PsycINFO/MEDLINE/Web of Science) or replicate the search in at least one psychology/biomed database to mitigate single-database bias. If DOI overlap is higher than ~75%, report it and justify there's no need to conduct the entire search. Also, please ensure the deviation is mirrored in the Reporting Summary (not only Supplementary Table 26).

(b) Inclusion criteria & grey literature. Including non-peer-reviewed records and multiple languages broadens coverage but does it increase heterogeneity to worrisome levels? Please consider adding sensitivity analyses excluding grey literature and machine-translated records, and report whether key pooled estimates meaningfully change, especially heterogeneity estimates. This is an aspect not as explored as it should IMHO.

(c) Outcome harmonization & modeling. Building on the above, given substantial instrument heterogeneity, your threshold mappings and POMP transforms are appropriate but can be influential. Please add instrument-specific analyses and perhaps prevalence and severity (if this was done, I missed it, so please make sure to highlight it). As above, please comment more and report more on variance components (including level-2/level-3) for key models to illuminate where heterogeneity sits. Also clarify, expand, and explain interpretability.

(d) Geography & cohort concentration. Several outcomes draw predominantly from US cohorts raising representativeness concerns. Look at the work of Sakshi Ghai. Please identify any overlapping cohorts, conduct leave-cohort-out sensitivity analyses, and report non-US-only estimates where possible.

(e) Country clustering. I am honestly not sure I understood about this section but in the Supplementary materials you seem to have AI-based clusters. Please explain Please justify this approach. Why is needed? Does it have to be AI? Why not dummy coded? Or take in some advice by Economists/Sociologists/PolSci? So unless there are very good reasons for it, and I am sure there may be, I would advise to move AI-derived clusters to exploratory status (unless externally validated). And please ensure AI use is declared in Methods and Reporting Summary.

3. Results and interpretation

(a) Prevalence vs severity narrative. The abstract conveys high prevalence while stating severities remain below clinical cutoffs. This can be misread as downplaying burden (which would be counter to your results' goals). Please reconcile these messages by clarifying instrument properties and explicitly distinguishing "case-identification thresholds" from "mean severity" signals. I would also be more clear on the abstract and try to compare the levels of prevalence between the general population to highlight prevalence/incidence and contextualize risk for ERCs.

(b) Moderator interpretation & power. You conclude most individual-level moderators are null. Given power limitations and measurement heterogeneity, please soften the language ("no reliable evidence given current data") and include a table of marginal power across moderators (k, effective n, ICC). The small negative age effect (≈1% per decade) should be framed cautiously, too.

(c) Outcome coverage. You note thin evidence for sleep quality and other outcomes; consider flagging burnout and related constructs explicitly as gaps (many ECR surveys seldom burnout) to guide the field's data collection priorities.

4. Presentation, clarity, and completeness

Please include a PRISMA flow diagram and place key extraction/threshold tables (now only in the Supplement) into the main text or Extended Data for accessibility. Label figures to distinguish prevalence vs severity panels MORE clearly; add footnotes noting when estimates are dominated by specific cohorts (e.g., Healthy Minds).

5. Data, code, and reproducibility

Include a README with session info and exact package versions, and archive a release (e.g., Zenodo DOI) at acceptance. A CSV

with harmonized study-level variables (cutoffs, instruments, sample frame) would be highly useful. I will make the point again that I believe you are not doing enough work to 'sell' your work. Please improve your approach to scientific communication, this is such an important study, find ways to break information down for popular media and non-experts. Perhaps create a dashboard or website?

6. Ethics and reporting
NA

7. Minor points

Provide the variance explained by “scale type” vs “syndrome type” (R^2 analogues) and their implications.

Clarify whether duplicate samples across multiple reports were deduplicated or modeled via three-level random intercepts; if not, please address.

Maybe define POMP, arcsine or other transforms at first mention; add plain-language glosses in figure captions.

8. General remarks:

I would love to see a bit of discussion on impostor syndrome and how it may or may not influence the results.

I would also love to read more about what (systemic) factors you think lead to heightened levels of anxiety-related feelings in ECRs. Perhaps expanding/exploring on career advancement uncertainty, neo-liberalization and commodification of education and scientific production, hidden curriculum, lack of equitable authorship practices, lack of effective DEAI practices in academia, and so on. Here's a couple of papers from my work that might be useful but there's so much out there making much better arguments:

https://doi.org/10.1038/s41562-022-01508-2
https://doi.org/10.1038/s41562-023-01572-2

Reviewer #3 (Remarks to the Author):

The manuscript is generally well-written and offers a clear, comprehensive analysis of the topic. The authors effectively present their findings, and the structure of the paper is logical and easy to follow. However, placing the methods section at the end of the manuscript appears to be an oversight and should be reconsidered.
The study addresses a significant issue in academia and provides valuable insights into the mental health challenges faced by early-career researchers. Nonetheless, several areas could be improved to further strengthen the manuscript.
Significance
This article presents a detailed and up-to-date meta-analysis of psychological distress among early-career researchers, addressing a broad range of mental health issues beyond depression and anxiety. It includes data from before, during, and after the COVID-19 pandemic, offering valuable insights into long-term trends and the pandemic’s impact. The findings underscore the importance of study quality, sampling methods, and measurement tools in shaping results. The study highlights the urgent need for evidence-based interventions and systemic reforms to address mental health challenges in academia.
Data and Methodology
The authors relied solely on Scopus for their literature search. Most meta-analyses in this area utilize multiple databases—such as PubMed, Web of Science, or ERIC—to ensure broader coverage. Limiting the search to a single database could result in missing relevant studies, particularly in certain disciplines. The authors should consider incorporating additional databases or provide a clear justification for using only Scopus.
Additionally, the manuscript does not include the full search string or clarify its scope, which reduces transparency.
There are also inconsistencies in the PRISMA flowchart: the transition from 794 records to 651 excluded and 139 included leaves four records unaccounted for.
In the results section, the cumulative sample size is reported as 128,684 participants (125,642 doctoral and 2,690 postdoctoral researchers). However, the remaining 352 participants are not explained and should be accounted for.
Analytical Approach
The authors did not analyze the moderating effects of specific syndromes—such as depression or anxiety—separately, instead combining all symptoms into a single model. This approach may have diluted potential moderating effects. It is recommended that the authors conduct a separate analysis of all studies using a common instrument, such as the PHQ-9, which has a large sample size and standardized measurement. This could help clarify moderating effects more precisely.
The results also indicate that severity variations are strongly influenced by the choice of measurement tool. Conducting separate analyses based on specific tools may provide deeper insights into moderating factors.
Clarity and Context
The discussion section offers general suggestions—such as expanding mental health services and reducing academic pressure—but these recommendations are neither novel nor accompanied by specific implementation strategies. The authors could strengthen the discussion by proposing more concrete, actionable interventions tailored to institutional and policy-level reforms.

Version 1:

Decision Letter:

Our ref: NATHUMBEHAV-25094568A

17th March 2026

Dear Dr. Nater,

Thank you for submitting your revised manuscript "Prevalence and severity of mental health problems in early-career researchers: a systematic review and meta-analysis" (NATHUMBEHAV-25094568A). It has now been seen by the original referees and their comments are below. As you can see, the reviewers find that the paper has improved in revision. We will therefore be happy in principle to publish it in Nature Human Behaviour, pending minor revisions to comply with our editorial and formatting guidelines.

Importantly, please note that our preregistration policy (https://www.nature.com/nathumbehav/editorial-policies/preregistration-policy) asks authors to be transparent about any deviations from their preregistered plan and to report the originally planned analyses, except in cases where those analyses are no longer feasible or would be inappropriate to conduct. These planned analyses do not necessarily need to appear in the main text; where relevant, they can be reported in the Supplementary Information. This approach reflects a longstanding community expectation that preregistered analyses—particularly those yielding less favourable or less clear results—should not be omitted from the research record.

For consistency with our policy, we ask that you classify each deviation from the preregistered plan into one of two categories—“inappropriate” or “unfeasible”—providing brief justification as needed (as you have begun to do in the ‘Deviation description’ column of your SI table). For any deviations that do not fall into either category, please report the corresponding preregistered analyses in the revised manuscript or Supplementary Information.

We are now performing detailed checks on your paper and will send you a checklist detailing our editorial and formatting requirements within two weeks. Please do not upload the final materials and make any revisions until you receive this additional information from us.

Please do not hesitate to contact me if you have any questions.

Sincerely,

[redacted]

[redacted]
Nature Human Behaviour

Reviewer #2 (Remarks to the Author):

Thank you for addressing all issues.

Reviewer #3 (Remarks to the Author):

The authors have addressed my previous comments. I hereby recommend accepting the manuscript.

Version 2:

Decision Letter:

Dear Professor Nater,

We are pleased to inform you that your Article "Prevalence and severity of mental health problems in early-career researchers: a systematic review and meta-analysis", has now been accepted for publication in Nature Human Behaviour.

Authors may need to take specific actions to achieve compliance with funder and institutional open access mandates. If your research is supported by a funder that requires immediate open access (e.g. according to Plan S principles or the NIH public access policy) then you should select the gold OA route, and we will direct you to the compliant route where possible. Because authors warrant under our subscription licensing terms that they haven’t committed to licensing any version of their article under a licence inconsistent with the terms of our agreement – including the applicable embargo period – publication under the subscription model isn’t suitable for authors whose funders require no embargo.

Once your manuscript is typeset and you have completed the appropriate grant of rights, you will receive a link to your electronic proof via email with a request to make any corrections within 48 hours. If, when you receive your proof, you cannot meet this deadline, please inform us at rjsproduction@springernature.com immediately. Once your paper has been scheduled for online publication, the Nature press office will be in touch to confirm the details.

Acceptance of your manuscript is conditional on all authors' agreement with our publication policies (see http://www.nature.com/nathumbehav/info/gta). In particular your manuscript must not be published elsewhere and there must be no announcement of the work to any media outlet until the publication date (the day on which it is uploaded onto our web site).

If you have posted a preprint on any preprint server, please ensure that the preprint details are updated with a publication reference, including the DOI and a URL to the published version of the article on the journal website.

An online order form for reprints of your paper is available at https://www.nature.com/reprints/author-reprints.html. All co-authors, authors' institutions and authors' funding agencies can order reprints using the form appropriate to their geographical region.

We welcome the submission of potential cover material (including a short caption of around 40 words) related to your manuscript; suggestions should be sent to Nature Human Behaviour as electronic files (the image should be 300 dpi at 210 x 297 mm in either TIFF or JPEG format). Please note that such pictures should be selected more for their aesthetic appeal than for their scientific content, and that colour images work better than black and white or grayscale images. Please do not try to design a cover with the Nature Human Behaviour logo etc., and please do not submit composites of images related to your work. I am sure you will understand that we cannot make any promise as to whether any of your suggestions might be selected for the cover of the journal.

You can now use a single sign-on for all your accounts, view the status of all your manuscript submissions and reviews, access usage statistics for your published articles and download a record of your refereeing activity for the Nature journals.

To assist our authors in disseminating their research to the broader community, our SharedIt initiative provides you with a unique shareable link that will allow anyone (with or without a subscription) to read the published article. Recipients of the link with a subscription will also be able to download and print the PDF.

As soon as your article is published, you will receive an automated email with your shareable link.

In approximately 10 business days you will receive an email with a link to choose the appropriate publishing options for your paper and our Author Services team will be in touch regarding any additional information that may be required.

If you have any questions about our publishing options, costs, Open Access requirements, or our legal forms, please contact ASJournals@springernature.com

We look forward to publishing your paper.

With best regards,

[redacted]

[redacted]
Nature Human Behaviour

P.S. Click on the following link if you would like to recommend Nature Human Behaviour to your librarian http://www.nature.com/subscriptions/recommend.html#forms

** Visit the Springer Nature Editorial and Publishing website at www.springernature.com/editorial-and-publishing-jobs for more information about our career opportunities. If you have any questions please click here.**

Open Access This Peer Review File is licensed under a Creative Commons Attribution 4.0 International License, which permits use, sharing, adaptation, distribution and reproduction in any medium or format, as long as you give appropriate credit to the original author(s) and the source, provide a link to the Creative Commons license, and indicate if changes were made.
In cases where reviewers are anonymous, credit should be given to 'Anonymous Referee' and the source.
The images or other third party material in this Peer Review File are included in the article's Creative Commons license, unless indicated otherwise in a credit line to the material. If material is not included in the article's Creative Commons license and your intended use is not permitted by statutory regulation or exceeds the permitted use, you will need to obtain permission directly from the copyright holder.
To view a copy of this license, visit https://creativecommons.org/licenses/by/4.0/

Comments by Reviewer 1 (R1)

R1.0

Thank you for the opportunity to read this paper. What an enormous amount of work has gone into this. I believe your work here is a really powerful contribution to the sector, Providing much needed data-driven insights into what's going on for the mental health of our early career researchers.

I have a number of reflections on the manuscript. I hope you don't mind me walking through these in the order in which they appear. I must note, this is a huge piece of work to read, it is very possible that at points I have missed things, so your answer to some of my questions may simply be we've already said that in this place here. I do think though that there is work perhaps that can be done to help make your manuscript Easier to navigate and digest for Other readers. This work is really important and we need Audiences within And beyond the academic community to understand your findings

I have a long list of suggestions here, some of which are larger than others. I want to stress, that I am sharing these comma, in the attempt to help you improve you make this as accessible as possible manuscript. I think this is a really sound piece of work, that needs publication, my questions and critiques are around how you are presenting the work , And how you make this as accessible as possible

We thank the reviewer for their detailed reading of the paper and have provided responses to each comment below. We appreciate the positive feedback on the significance and the effort put into the work. To make the paper more readable for general audiences, we have made several changes (e.g., removing some technical abbreviations, tightening the Results section, and contextualizing mental health prevalence rates).

R1.1

Abstract

Line 30 - here for the first time you use the word symptom. You do this at multiple points across the paper. I'm not sure when you're saying that an ECR meets the criteria for at least one mental health symptom, that you have ever used scales that measure a single symptom on their own, they might measure a cluster of symptoms. I wonder if you could look at this again.

Our meta-analyses included both clinically defined syndromes and symptom domains. We agree that many of these domains do not represent single symptoms but rather clusters or domains of related symptoms, which are generally assessed using multi-item scales (e.g., the Pittsburgh Sleep Quality Index, PSQI). We now define symptom domains as encompassing both grouped or composite symptoms (e.g., stress, sleep disturbance) and individual symptoms. In the more technical sections of the manuscript (Methods and Results), we use the term symptom domain, and in the more narrative sections (Abstract, Introduction, and Discussion), we use the term symptom for readability (p. 19).

> "We use the term symptom domains to refer to both composite or grouped symptoms (e.g., stress, sleep disturbance) and individual symptoms (e.g., suicidal thoughts). In the more technical parts of the article, we use "symptom domain" for precision. In the narrative parts of this article (Abstract, Introduction, and Discussion), we use the term "symptom" for readability."

R1.2

Line 40- your brief summary here about the effect of age, doesn't tell us the direction.

With the inclusion of additional data from the databases ERIC, Web of Science, and PubMed, we find a negative significant association between prevalence and age (β = -0.01, CI -0.03 – -0.00, *p* adjusted = .339). Thus, per increase in one year in age, the overall prevalence for mental health syndromes and

symptoms decreases by 0.01%. Hence, every ten years the prevalence decreases by 1%. In line with this, we have updated the table of moderators in the main text and provided detailed information in the Supplementary Material, Supplementary Table 17.

R1.3

1. in the introduction You talk about issues for ecr's and PhD students and you bring in doctoral attrition. Can we have much more clarity early on about any differentiation you see between what is going on for postdoctoral researchers doctoral researchers and pre doctoral researchers. I must note that I did not originally understand that you included doctoral researchers in your study. I think this could be clearer both in the introduction methods and result

We have clarified our language in the very first sentence to emphasize that predoctoral researchers were in our meta-analysis.

> "Mental health concerns among early-career researchers (ECRs), including predoctoral (PhD students) and early postdoctoral researchers, have been gaining attention and point towards a potential mental health crisis in academia[1–7]." (p. 4)

The Methods section already clearly defines ECRs as including both predoctoral (PhD students, doctoral candidates) and postdoctoral researchers, consistent with the European Council's framework on R1 and R2 researchers (p. 22), so no additional revisions were necessary.

We agree that a clearer distinction between which stressors affect predoctoral and postdoctoral researchers more strongly would improve the introduction. We now list the most common stressors faced by ECRs overall and specify which are more typical for predoctoral and postdoctoral researchers. We also slightly reworded the preceding sentence to improve flow with this distinction. In addition, we note that research on postdoctoral mental health remains limited, as most studies have focused exclusively on PhD students, and we highlight this as a future research gap in the discussion.

> "Doctoral students and postdoctoral researchers share many of these challenges, though some stressors may be more pronounced in doctoral students (e.g., supervisor dependency, thesis progress pressure, financial strain, impostor feelings)[2,13,19] and in postdoctoral researchers (e.g., short-term contracts, career uncertainty, pressure to publish or secure funding)[7,12]." (p. 4)

R1.4

2. Unit for individual studies suggest a significant proportion of doctoral students have completed suicide. Can you contextualise this for us, to look at the prevalence of suicide in Among doctoral students among among doctoral students In the context of the prevalence for age matched control

We thank the reviewer for this important comment. We would like to clarify that the statement in the Introduction section referred to current suicidal ideation (i.e., current thoughts of killing oneself), not completed suicide. A recent review by Poli et al. (2025) on suicide-related outcomes among medical residents and PhD students from various disciplines found current attempted suicides (0.8%) and lifetime suicide attempts (3.1%) to be substantially lower than rates of suicidal ideation (8.6%) but still higher than those observed in age-matched general-population samples. In response to the reviewer's comment, we have revised the manuscript to more clearly contextualize suicidal ideation among doctoral students relative to age-matched general-population samples using comparable time frames (p. 4).

> "A recent meta-analysis on doctoral students and medical residents estimated that 8.6% reported suicidal ideation in the past year[10]. Population-based studies report lower prevalence in comparable age groups (3.9–4.9%)[11–13], suggesting that suicidal ideation among doctoral students is roughly two times higher than in the general population."

R1.5

3. Your introduction told us a lot about what you were going to do, in terms of reviewing mental health issues beyond anxiety and depression. But in focusing on explaining what you were going to do, your argument for the rationale of why it was important was lost

As part of the revisions we have made in response to comment R1.7, we have updated our rationale for the current study and its contribution beyond previous work. Specifically, we now emphasize that an up-to-date and comprehensive synthesis of psychological distress among ECRs is needed to extend existing research on depression and GAD and to identify factors that influence prevalence and severity estimates.

R1.6

4. You know there's a high Prevalence of psychological distress among a ECR's underscoring an urgent need. Please can you present this in the context of prevalence of mental distress among an age much cohort. Is this specific to ECR's?

We have added estimates for age-matched controls in the general population to the introduction (see also our response to R1.29). (p. 4)

> "Existing meta-analyses estimate that 21–24% of predoctoral researchers exceed screening cut-offs for depressive symptoms and 17–29% for anxiety symptoms. When compared to age-matched groups in the general population, prevalence rates for ECRs are markedly elevated, with population-based studies reporting prevalence rates from 7.9%–16.6% for depressive symptoms and 6.4%–10.1% for anxiety symptoms[31–33]."

R1.7

5. Across the introduction in general I think the rationale for this study And how it's going to tell us something more and new than previous work is done, could be better communicated.

We have expanded our final paragraph in the Introduction section to summarize the rationale and clarify the scope relative to previous work, outlining the outcomes and moderators included in the present study. We also aimed to balance our response to this comment with NHB's guideline to refrain from novelty and priority claims (p. 6).

> "To summarize the available evidence on a wide range of mental health issues among ECRs and to identify factors that modulate this burden, we conducted a systematic review and multilevel meta-analysis[49] that quantifies the prevalence and severity of a broad range of mental health syndromes and symptoms included under the umbrella term psychological distress."

R1.8

Methods

1. I've never understood why journals asked for these to be at the end of the manuscript. This isn't your problem but some people would like to read the methods before they read the results. There are certain things that you don't explain in the methods because you have already explained in the results. I would encourage you to explain these including acronyms, in the method section for the reader who wants to understand what you did before they go on to read what you found

We have expanded our Methods section to provide all necessary definitions and explanations for readers who want to read the Methods first. Specifically, we included detailed definitions and explanations on all mental health outcomes and their abbreviations, explained how screening thresholds were determined, detailed the individual-level and study-level factors included as

moderators, specified how COVID-19 years were coded, and described the procedure used to generate the country clusters. We kept the Methods section at the end of the main text to comply with the journal's formatting guidelines.

R1.9

2. I'm interested that you manual search in Scopus will stop I haven't seen this done before, I wonder if you could say a bit more about why you pivoted to take this approach

We thank the reviewer for this comment. We are not certain to what is referred here, as the Methods section does not mention a "manual" search in Scopus. If this reviewer was referring to our preregistration deviation – we initially (i.e., preregistration) planned searching Scopus (including backward and forward citation searches) as the primary database – we have now expanded the search strategy to include additional databases as well (i.e., Web of Science, PubMed, and ERIC).

R1.10

3. In study selection and eligibility, can you tell us more about how many researchers were involved in the screening, and how any disagreements between researchers were resolved

One researcher (VG) was involved in the original SCOPUS screening and in the forward and backward reference searches up to January 2024. Two researchers (DF, VG) were responsible for the screening of PubMed, Web of Science, and ERIC, which was added in carrying out the revision of the originally submitted manuscript. Disagreements were resolved through discussion and consensus, drawing on a third researcher (UT) when necessary.

R1.11

4. You note that you included primary datasets, can you tell us a bit more about the availability of primary datasets, and the extent to which you were able to tap in to original data sets.

Data from four primary databases were included in the meta-analysis and systematic review. First, several individual datasets (14 on students and two on staff) were included from the Healthy Minds Network. Datasets from this network can be requested online (hmn-datateam@umich.edu) and were shared with author VG via Dropbox. Second, data from the COVID-19 International Student Well-Being Study was accessed via Zenodo. Third, Nicola Byrom was contacted by author VG and shared the primary data for her paper "COVID-19 and the Research Community: The challenges of lockdown for early-career researchers" via a link that makes the data publicly available to researchers. Lastly, data on the "Nationwide assessment of the mental health of UK Doctoral Researchers" by Hazell and colleagues (2021) was provided by Jeremy Niven. All primary datasets were analyzed by author VG, the R code for the analysis is available on OSF (MA Analysis_Raw data).

R1.12

5. You note at the end of the section on data extraction that any inconsistencies were discussed and agreed upon. Can you tell us more about the scale of inconsistencies that you found. Perhaps you could say a little bit more about what gave you confidence that simply reviewing and checking into code of reliability in 30 articles was sufficient

Thirty-two non-metric coding items were assessed, including items on the scale used to assess a syndrome or symptom, publication year, type of report, and the country in which the study took place. The mean percentage agreement between the two raters (VG and DF) was 94.33%. Cohen's kappa was considered equally substantial (mean = .65) but also appeared to be downwardly biased by the unequal distributions of categories in some of the rated items (which is why we considered mean percentage the more appropriate statistic here). All coding items for outcome variables, expressed as percentages and summary scores (mean, SD), yielded high ICCs. However, we nonetheless carefully reevaluated,

revised, and clarified all information in our coding manual for coding items with low Cohen's kappa coefficients or ICCs, which included variables on the names of the different scales used to assess a syndrome or symptom, four variables coding the JBI quality criteria, and participants' ethnicity. For example, the double coding revealed differences in the coding of the 'type of report', as one coder (DF) coded one paper as a pre-print, whereas the other coder coded the same paper as a 'journal article'. However, we also realized a category for 'book chapter' was missing; thus we added this to the coding manual. Similarly, a category for 'international' was missing in regard to the country where the data was collected, and the coding manual adapted as well. Other differences were seen in the rounding of numbers, or that one paper was falsely classified as having used non-external assessment tools when, in fact, it had. We have highlighted this in the coding manual by providing additional examples of what constitutes an external assessment. Detailed information on coding deviations is provided on OSF (Intercoder Reliability).

A second independent coding of all studies was found to be prohibitively time-consuming and thus infeasible in the present study. However, the random selection of a subset of articles that were independently coded a second time (a common practice in meta-analysis, if the total set of studies is large; see, e.g., Cooper et al., 2019, *Handbook of research synthesis and meta-analysis*, p. 169) provided us with the opportunity to generalize the observed interrater reliability to all other (single-coded) studies as well. We concede that thirty studies did not allow us to estimate agreement statistics with high accuracy (i.e., obtaining narrow confidence intervals). However, we were not interested in conducting inferential statistical tests here but rather in gaining insight into the observable agreement and possible problems with the coding of individual items. For this, we drew on the point estimates of the agreement statistics themselves, rather than doing inferential statistical tests with these coefficients (but see, e.g., Sim & Wright, 2005, https://doi.org/10.1093/ptj/85.3.257, Table 8, which shows that 30 independently rated objects provide at least 90% power in a 2-tailed test against 0 for dichotomous rating systems and proportions of positive ratings as low as 10%). Against the background of a large number of investigated coding items, such an alternative inferential statistical approach would also have been prone to problems of repeated testing and, hence, type 1 error accumulation. Thus, we did not deem such an alternative approach informative.

R1.13

6. In effect size measures, you introduce pomp scores. You don't explain what these are. Please could you do so here. As I read your results I could see that you had done something really sensitive and nuanced to the variation across the scales that you were working with. This sensitivity and care does not come across in the methods.

We agree that the original description of POMP scores in the Methods section did not sufficiently convey the rationale for using and the interpretation of this effect size. In the revised manuscript, we have therefore expanded the effect-size section to provide a clearer explanation of what POMP scores represent and how they should be interpreted. POMP scores transform the scores of individual scales into a normalized scale range from 0 to 100. A POMP score of 50 thus represents the midpoint of the original scale; values above 50 indicate higher, and values below 50 indicate lower symptom severity than the midpoint. We fully integrated this explanation into the Methods section to improve clarity and interpretability (p. 23).

> "The extent of psychological distress among ECRs was investigated separately with respect to prevalence and severity. Prevalence was estimated for outcomes using psychometrically validated or commonly used screening thresholds (e.g., PHQ-9 ≥ 10), or binary thresholds (yes/no) response formats (Supplementary Tables 1, 20–29). Symptom severity was estimated with summary statistics (means and standard deviations) of continuous measures. Because primary studies employed a wide range of instruments to assess psychological distress and

related syndromes and symptoms, the summary statistics were not directly comparable across studies. To address this heterogeneity, and following previous meta-analyses[147,148], we converted summary statistics into percent of maximum possible (POMP) scores[149]. POMP scores transform different, arbitrary scores (of tests, scales, surveys, etc.) into a standardized scale ranging from 0 to 100. For example, if a respondent scores a 5 on a scale ranging from 1 to 7, this corresponds to a POMP score of 66.67 (see below for details and calculation). A POMP score of 50 corresponds to the midpoint on the original scale, whereas POMP scores above 50 indicate higher and below 50 indicate lower symptom severity than the midpoint of the original scale."

R1.14

7. P-31 line 646, you say that PHQ 9 can only be between nought and one. I think there's a typo here. And you meant nought and 3.

We fixed it and have expanded our clarification on the probability distribution for Item 9 of the PHQ-9. (p. 24)

R1.15

8. On the same point, I have never seen an normally distributed PHQ 9 data set, The population is usually negatively skewed on this scale. So I wonder why you made the assumption that scores above 0 would be equally distributed

Thank you for your attention to detail. You are correct that PHQ-9 scores are typically negatively skewed, a feature that was not adequately accounted for in our original statistical approach for this scale. Thus, for the revised manuscript, we derived an expectation for the shape of the PHQ-9 score distribution from the largest dataset available to us (Healthy Minds Network, with over 50,000 participants). The exact analysis of these raw data has been uploaded to OSF and can thus be fully reproduced (MA_raw data analysis.qmd), and we elaborate now on this in the main text of the manuscript (headline: Calculation of POMP scores, p. 24f).

"Where no probability distribution was provided, such as in the case of item 9 of the PHQ-9, it was necessary to first determine the items' probability distribution. We used the largest available database (Healthy Minds Network, n > 50,000), which provided the following score distribution: 0 = 87.16%, 1 = 9.40%, 2 = 2.13%, and 3 = 1.32%. From this, we determined that the probability of scoring 2 is 1/4.41 times the probability of scoring 1, and the probability of scoring 3 is 1/7.12 times the probability of scoring 1. The proportion to score 1 was thus determined via the following formula, solving for x:

$$\%\text{at or above cut} - \text{off}\ (\geq 1) = 0 * x + 1 * x + \left(\frac{1}{4.41}\right) * x + \left(\frac{1}{7.12}\right) * x \approx 1.37 * x$$

For example, in the case of ref. 84, 3.1% scored above the cut-off of ≥ 1. From this, we concluded that 96.9% must have scored 0, and based on the above formula, we estimated that 2.27% scored 1, 0.51% scored 2, and 0.44% scored 3. With this, we then used the above formulas for *M* and *SD* again. The exact analysis with the raw data is available on OSF (MA_raw data analysis.qmd)."

R1.16

9. In critical appraisal, line 688, I don't understand what you mean by a response rate. Did papers reliably tell you what the target population was and how large that was? How have you in interpreted a response rate if there is no recruitment sample specified?

First, it is important to differentiate between the terms ‘target population’ and ‘recruitment sample.’ According to the JBI, the criteria ‘sample frame’ addresses the following question: Was the sample frame appropriate to address the target population? And hence, “relies upon knowledge of the broader characteristics of the population of interest and the geographical area.” This information was considered appropriately provided if the study reported whether the target population matched the recruitment sample. The exact criteria can be traced in the Supplementary Table on Risk of bias (JBI) assessment coding scheme. However, most studies did not provide precise information on the target population, let alone conduct a comparison between the target population and the recruitment sample based on socio-demographic information and adjust the sample data to the target population in analysis (e.g., by assigning weights) in case of differences. In cases where random or stratified sampling was used, we assumed that the sample corresponded to the target population. We used the term ‘response rate’ solely with reference to the recruitment sample, but not the target population (i.e., the rate of persons contacted by the study authors, who also consented to participate in the study). If no data was provided, authors were contacted directly to provide information on the response rate. It was not considered sufficient for the authors to simply state the response rate to receive a full point on the JBI quality criteria rating. The response rate needed to be larger than > 45 %, and if lower, a non-response analysis or justification by the authors needed to be provided. The exact points awarded per information provided are given in the Supplementary Table 12 on Risk of bias (JBI) assessment coding scheme.

> “In order to evaluate the adequacy of response rates (i.e., the rate of persons contacted by the study authors, who also consented to participate in the study), an external cut-off criterion of 45% was employed, based on the average online survey response rate169. Studies could score a full point if the response rate was adequate (≥ 45%), a quarter point if the response rate was provided but below 45%, and no points if no information was provided.” (p. 26)

R1.17

10. Line 700 and line 705 are 2 examples of points where you're referencing seems strange. Sometimes the references are in numbers in brackets. And I don't understand how we interpret ref.

We thank the reviewer for noting these inconsistencies. For the refs[17,18] we have changed it in the revised manuscript. For any instances where we used “ref. #” to highlight a particular reference, we have kept them because they correspond to the journal's referencing style. We also reviewed the manuscript for additional inconsistencies in in-text citations and the bibliography and fixed minor errors.

R1.18

11. Line 735 the acronym FDR has not been defined

We added the definition at first mention, both in the main text and in the Methods section.

> “Moderators that remained statistically significant after correcting for a false-discovery rate (FDR) of 5% are reported in Table 1 and in-text.” (p. 4)

> “In the subsequent step, we conducted separate multivariate moderator analyses for the prevalence and severity models, including only moderators that remained significant after controlling for a false-discovery rate (FDR) in the bivariate analyses.” (p. 27)

R1.19

Results

1. Did your sample include any pre doctoral research assistance? Your introduction implied as much, but I don't see any mention in the results.

We thank the reviewer for noting this ambiguity. Assuming the reviewer refers to PhD students or doctoral candidates, yes, the meta-analysis mostly consisted of these predoctoral researchers (*N* = 134,572 doctoral). Following the definition of the European Council[102], we defined this group as researchers enrolled in or working for higher education institutions and who are actively pursuing a PhD. If you are referring to a population of students who work as research assistants (e.g., master's students) but are not actively pursuing a PhD, then this group was not included.

> "Our analysis includes both predoctoral researchers who are actively pursuing a PhD degree and postdoctoral researchers and…" (p. 6)

> "Predoctoral research assistants (e.g., master's students) who were involved in research projects but were not actively enrolled in a PhD program were not included." (p. 19)

R1.20

2. I would like to know more about how you defined academic disciplines, wrangling the different ways disciplines of their participants and how you have managed to cluster them into neat categories. One problem here that jumps out at me, is you note that psychology accounts for 32% of social sciences. In the UK psychology is defined as a medical science, and would be reported as part of a population group of Medical Sciences. I'm not sure I understand how you will have managed data across studies where for example they may have only reported the percentage of students studying Medical Sciences rather than the types of subjects within that categorisation. I can see this as being unbelievably complicated for you to have managed.

Information on how different academic disciplines were classified is available in Supplementary Table 2 in Supplementary Materials. We clustered psychology with the social sciences, following Wikipedia's classification, which also aligned with our expectation of defining psychology in most of continental Europe and the Anglosphere. We were unaware that, specifically in the UK, psychology is defined as a medical science. We have retained our original categorization in the revised manuscript, but have emended the sensitivity analysis in the Supplementary Materials (Table 16) to categorize psychology as a medical science. This did not change the results as the moderator remained non-significant in the prevalence ($F[4, 40] = 0.37$, $p = .083$) as well as the severity model ($F[4, 56] = 0.17$, $p = .950$).

R1.21

3. I think given the focus that you have on outcome measures later on in the results and then in the discussion, it would be helpful in your section on the characteristics of studies to give us a breakdown summary of the outcome measures used so common syndromes

In our breakdown of the characteristics of the studies included in the current meta-analysis, we have summarized the different outcomes and the number of samples for each syndrome or symptom. For the revision, we included three additional databases (ERIC, Pubmed, and Web of Science) and updated the counts per outcome accordingly.

> "Samples provided data on depression ($k = 166$), GAD ($k = 121$), stress ($k = 69$), suicidal ideation ($k = 49$), psychological distress in general ($k = 39$), burnout and eating disorders ($k = 28$ each), non-suicidal self-injury ($k = 23$), alcohol abuse ($k = 24$), sleep quality ($k = 9$), somatic symptoms ($k = 4$), drug abuse ($k = 4$), ADHD, OCD, bipolar disorder ($k = 2$ each), social, specific and agoraphobia, panic disorder, PTSD and personality disorders ($k = 1$ each)." (p. 7)

R1.22

4. I have to say, I'm sorry the section on pooled prevalence and severity estimates by syndromes and symptoms is really dense and hard to follow.

In the revised manuscript, we have shortened this section and made efforts to improve the writing to make the section more readable and easier to follow (p. 8f).

> "**Pooled prevalence and severity estimates**
>
> Across all outcomes, nearly one in three ECRs reported elevated psychological distress (pooled prevalence = 29.9%; 95% CI, 26.1–33.9%; *k* = 86). Symptom severity across all outcomes was 33.9 (95% CI, 31.7–36.1; *k* = 151) on a normalized scale from 0–100, using percent of maximum possible (POMP) scores (see Methods for details). Heterogeneity was high (roughly equal amounts of heterogeneity within and between samples for severity estimates; for prevalences, heterogeneity was higher between than within samples; see Supplementary Tables 14 and 15), with 95% prediction intervals ranging from 0.2–71.1% for prevalence and 0.8–67% for severity estimates. This indicated wide ranges for the expected results of future individual studies. Forest and caterpillar plots are available in Supplementary Figs. 4-12.
>
> **Prevalence and severity estimates from the PHQ-9, item 9 of the PHQ-9, and GAD-7**
>
> The pooled prevalence of depressive symptoms in the most used scale, the PHQ-9, was 28.5% (95% CI, 24.3–32.9) and severity estimates indicated that 33.2% (95% CI, 30.7–35.8) of ECRs reported mild symptoms, 17.3% (95% CI, 15.3–19.3) moderate, 8.2% (95% CI, 6.8–9.8) moderately severe, and 4.8% (95% CI, 3.7–6.1) severe depressive symptoms. The pooled prevalence of suicidal ideation (item 9 of the PHQ-9) was 17.4% (95% CI, 13.8–21.4). The pooled prevalence of anxiety symptoms in the most used scale, the GAD-7, was 27.6% (95% CI, 23.4–31.9), with 27.1% (95% CI, 23.6–30.7) reporting mild, 16.1% (95% CI, 14.0–18.3) moderate, and 9.6% (95% CI, 7.9–11.4) severe anxiety."

R1.23

5. I haven't understood the rationale for why you focused in on different burnout scales why has this got more attention?

We conducted these analyses because burnout is a multi-dimensional construct and ECRs may experience some subdimensions of burnout (emotional exhaustion, depersonalization, and personal accomplishment) more strongly than others. This was indeed the case, with ECRs reporting that they struggled more with personal accomplishment than with exhaustion and depersonalization, perhaps because of the strong identification that researchers often have with their work. In providing these more detailed results, we strove to make the interpretation and the likely implications of the available evidence on burnout more tangible and focused.

> "Since burnout is a multidimensional construct, and ECRs may experience its subdimensions to varying degrees, we conducted post-hoc exploratory analyses (without FDR control) examining the severity across the three burnout subscales (emotional exhaustion, depersonalization, and personal accomplishment; Supplementary Table 16)." (p. 9)

R1.24

6. Figure 3 is fascinating. But complex. I think it could be really helpful to spend a bit of time in the text talking the reader through how to understand these graphs. Perhaps you could walk through an example with a POMP score

We agree that Figure 3 is complex and could benefit from additional guidance. In the revised manuscript, we have therefore added plain-language descriptions to each figure (see also our response to R2.13) explaining how to interpret the displayed estimates. We now also explain how, for this figure, severity estimates were back-transformed to ease interpretation. In addition, we have added an example with the PHQ-9 to illustrate how severity estimates can be understood on their original scales.

Finally, we provide additional material in the Supplementary Material with detailed information on scale ranges, scoring methods, and severity thresholds for all measurement scales used in the analysis.

> “As shown in Fig. 3, severity levels varied widely depending on which measurement scale was used. To facilitate interpretation, severity estimates displayed in Fig. 3 were back-transformed into their original units of measurement. For example, the pooled severity for depressive symptoms as assessed with the PHQ-9 (44 studies, 67 effect sizes; n = 72.239) was 6.99 (95% CI, 6.12–7.86), indicating symptom levels in the mild range. Additional details on each respective scale, including scale ranges, scoring method, and severity thresholds, are available in Supplementary Tables 20 - 29.“ (p. 10)

> “Dots indicate pooled severity estimates for each scale, displayed on a normalized (POMP) scale ranging from 0-100, to allow comparisons across measurement scales, with bars representing 95% confidence intervals. Colored bands indicate the severity categories derived from the original measurement scale. The pooled severity estimates per measurement scale, including 95% confidence intervals, are also shown on the right.” (p. 32)

R1.25

7. Line 289. Can you be specific about how you have classified the COVID-19., what were the years or months, that you used to cut off

To analyze the impact of the COVID-19 pandemic, we classified studies by year of data collection. Studies with data collection were classified as before COVID-19 (years before 2020), during COVID-19 (2020-2022), or after COVID-19 (data collection after 2022). For studies that did not explicitly report the year of data collection, we assumed it was 1 year prior to the publication year. We have updated the revised text to make our approach clearer.

R1.26

8. You discuss the moderating effects of study design and quality before risk of bias. There is something disjointed in this as it would be helpful to understand the shape and nature of the data in terms of study quality before you tell us the impact of study quality on prevalence and severity estimates

We agree that the risk of bias should be discussed first if study quality is used in subsequent moderator analyses. Therefore, we now present the risk of bias section directly after our description of the study characteristics in the revised manuscript. There were some small moderating effects of study quality, but these did not substantially affect prevalence and severity estimates and, hence, are reported in full in Supplementary Table 17. We hope this change in the order of presentation now makes the moderator analyses' narrative flow more coherent.

R1.27

9. I am afraid I really struggled with this section of your findings. You are telling us that study quality significantly moderates prevalence and severity estimates. Something actually that we knew from previous research, as you wove it into the introduction. And you are confirming that there is considerable variability in the study quality, of studies included in your meta analysis. This comes after a lot of analysis on the prevalence and severity, which has presumably used all of the available data. I can see from Table 1, That you have included study quality in your regression model. Can you confirm consistently across the results, that the findings you are reporting are off are after having controlled for study quality?

Pooled estimates for prevalence rates and symptom/syndrome severity were based on data from all eligible studies, irrespective of study quality. Study quality was subsequently entered as a moderator in bivariate and multivariate analyses to assess its association with prevalence and severity estimates.

We have added clarifications to both the Methods and Results sections to make this analytical sequence explicit.

> "All pooled prevalence and severity estimates reported above were based on data from all eligible studies, irrespective of study design or quality. Study quality was a significant moderator, such that higher study quality was associated with lower prevalence (-0.14% per JBI score point; 95% CI, -0.40 to -0.01; R^2 = 11.6%) and severity estimates (-3.06 POMP score points per JBI score point; 95% CI, -4.65 to -1.46; R^2 = 7.7%)." (p. 12)

> "Unless otherwise specified, models estimating prevalence and severity used data from all eligible studies, irrespective of study quality. Study quality (overall JBI score) was added later as a moderator in bivariate and multivariate moderator analyses to assess its association with prevalence and severity estimates." (p. 27)

R1.28

10. In table one, I don't see you including the country in which the study took part, or your clever clustering of countries. I wonder why? I would have thought there were important differences between types of country. I

Because of the larger number of investigated moderators and for ease of presentation, Table 1 contains only information on moderators that remained significant after controlling for the FDR (which was not the case for the country clusters). Information on all other moderators is presented in Supplementary Table 17. Empirically, country clusters apparently did not differ in reported prevalence or severity estimates.

R1.29

Discussion

1. You are comparing your results of prevalence estimates to the general population. Estimates of prevalence of depression and anxiety in the general population, especially for young adults, vary wildly. To me 5 and 6% look incredibly low. I would be grateful if you would recognise the variability that exists in population level prevalence estimates, the difference between ecr's and the general population may be much smaller then you were suggesting

We agree that there is substantial heterogeneity in population-based estimates of depression and GAD, which depend on several factors such as differences in assessment methods, diagnostic thresholds, timeframes, and national contexts (see also our response to R1.6). To address this and to better contextualize our findings on prevalences among ECRs, we have revised the discussion to include a range of prevalence estimates from large population-based studies of young adults and have updated the corresponding comparisons to reflect this variability.

> "When compared to age-matched groups in the general population, our results indicate that depressive (29%) and anxiety symptoms (29%) were markedly elevated among ECRs. Although the magnitude of this difference depends on the reference data[19], population-based studies of young adults using comparable screening thresholds (e.g., PHQ-9 ≥ 10; GAD-7 ≥ 10) typically report prevalences of 8–17% for depressive symptoms and 6–10% for anxiety symptoms[14–16]. This suggests that depressive symptom prevalence among ECRs is approximately two to three times higher, and anxiety symptom prevalence about three to five times higher, than among their age peers in the general population." (p. 4)

R1.30

2. I wonder if across the manuscript in general, and in the discussion in particular, you could think about how and where you could avoid using acronyms. This is a really complex paper. The use of

acronyms adds an unnecessary level of complexity. For example the use of the acronym Ed, and nSSI, In the discussion felt unnecessary

We have removed the abbreviations for general anxiety disorder, eating disorders, and non-suicidal self-injury throughout the manuscript. We have retained all standard abbreviations for disorders where the acronym is almost more well-known than the full term (e.g., ADHD, OCD). We also spell out the Clinical Interview Schedule, which was added for this revision. In addition, we have defined abbreviations in the Methods that were previously defined in the preceding sections, as some readers might prefer to read the Methods before the Results (see also our response to R1.8).

R1.31

3. I don't feel your paper, your analysis, justifies the text in the paragraph starting 476 in the discussion. Your analysis hasn't really told us anything about what's actually going to help. So I feel this section is an overreach, beyond your findings, but also feels unnecessary.

We thank the Reviewer for this comment. The goal of the current meta-analysis was to provide an accurate picture of the current status quo of mental health for ECRs, including a broad overview of outcomes beyond depression and anxiety, and to analyze moderating factors. We agree that recommendations about what stakeholders could do, do not directly follow from the results, as we did not test interventions. However, our meta-analysis found limited evidence for associations of individual-level moderators with psychological distress, underscoring the role of systemic factors. In addition, our goal was not just to describe the status quo but also to offer ideas for improving mental health among ECRs. Because the results underscore the poor mental health status quo among ECRs, we believe the implications for various stakeholders follow indirectly from our results and thus deem it important to offer practical suggestions. Reviewer 3 (Comment 8) also discusses this paragraph but recommended improving it by providing more concrete, actionable advice, and more concrete examples and practical recommendations. Finally, it is common for systematic reviews and meta-analyses to offer recommendations that follow indirectly from the results, including reviews on ECR mental health (Levecque et al., 2017; Satinsky et al., 2021).

We have thus amended this paragraph in the revised manuscript, clarifying that the implications do not follow directly from our results but rather reflect a summary of the literature on systemic change and individual interventions, as well as our own suggestions.

> "The present meta-analysis has several implications for research institutions and universities, funders and policymakers, supervisors, and ECRs themselves. Although this meta-analysis did not evaluate the effects of specific interventions, the modest associations of individual-level factors with psychological distress suggest that systemic factors likely play a substantial role and therefore require structural and institutional changes." (p. 16)

Levecque, K., Anseel, F., De Beuckelaer, A., Van Der Heyden, J., & Gisle, L. (2017). Work organization and mental health problems in PhD students. *Research Policy*, *46*(4), 868–879. https://doi.org/10.1016/j.respol.2017.02.008

Satinsky, E. N., Kimura, T., Kiang, M. V., Abebe, R., Cunningham, S., Lee, H., Lin, X., Liu, C. H., Rudan, I., Sen, S., Tomlinson, M., Yaver, M., & Tsai, A. C. (2021). Systematic review and meta-analysis of depression, anxiety, and suicidal ideation among Ph.D. students. *Scientific Reports*, *11*(1), 14370. https://doi.org/10.1038/s41598-021-93687-7

R1.32

4. line 501, can you tell us a bit more about the evidence that clinical diagnostic interviews lead to more reliable data then self reported rating scales. I'm not convinced they're better, just rather different.

We agree that self-administered mental health scales and clinical diagnostic interviews serve different purposes. Self-administered mental health scales are commonly used as screening tools to identify potential cases of, for example, general anxiety disorder or major depression in a two-step diagnostic process. Because self-administered scales are designed to identify potential cases, they cast "a broad net" (Zimmerman, 2024), so that individuals with the disorder are captured as potential cases during screening. In the second step, clinician-led semi-structured interviews may then identify actual cases and make a definitive diagnosis. The self-administered scales are thus designed to minimize missed diagnoses (false negatives), which means they tend to overestimate the true prevalence of the disorder (Levis et al., 2019).

In addition, self-administered mental health scales in epidemiological studies are used to estimate the prevalence of symptoms rather than the prevalence of diagnosable mental disorders (e.g., depressive symptoms instead of depressive disorder; Zimmerman, 2024). We have revised the manuscript to clarify this distinction.

> "To obtain a more accurate estimate of the prevalence and severity of psychological distress among ECRs, future studies should incorporate semistructured clinical interviews conducted by trained professionals to establish clinical diagnoses of mental disorders, as self-administered mental health scales only screen for potential cases[71]." (p. 15)

Levis, B., Yan, X. W., He, C., Sun, Y., Benedetti, A., & Thombs, B. D. (2019). Comparison of depression prevalence estimates in meta-analyses based on screening tools and rating scales versus diagnostic interviews: A meta-research review. *BMC Medicine, 17*(1), 65. https://doi.org/10.1186/s12916-019-1297-6

Zimmerman, M. (2024). The value and limitations of self-administered questionnaires in clinical practice and epidemiological studies. *World Psychiatry, 23*(2), 210–212. https://doi.org/10.1002/wps.21191

R1.33

5. Line 511, you note that many studies have not reported the exact sample composition, of whether their participants are pre doctoral ecr's or PhD students, this raised a question for me, of how have you actually managed this in the data? If it's not clear in the original papers, how have you differentiated between these groups?

When original papers did not report the exact sample composition, we contacted the corresponding authors twice using a standardized email template (Supplementary Table 34) to ask whether the sample consisted of predoctoral (PhD) researchers, postdoctoral researchers, or a mix of both. If the sample composition could not be verified, the study was excluded from the dataset.

> "In cases where the original report was not accessible, or the report lacked information on the exact sample composition, the corresponding authors were contacted via a standardized mail template (Supplementary Table 34) up to two times, and their responses were documented in Excel. If the exact sample composition (pre- and postdoctoral or MA students) could not be clarified, the study was excluded." (p. 22)

I intentionally sign all my reviews,

Nicola Byrom

Comments by Reviewer 2 (R2)

R2.1

1. Significance, originality, and fit for NHB

The topic of ECR mental health has clear societal and policy relevance, and a comprehensive, quantitatively rigorous synthesis is timely. The scale of aggregation and three-level modeling are strengths. With stronger robustness work and clearer communication around measurement/interpretation, this paper could be an impactful contribution for science and NHB's audience.

We thank the reviewer for their careful reading of our paper and for their thoughtful suggestions to improve the communication and robustness of the findings. For this revision, we have added three additional databases (ERIC, PubMed, and Web of Science), provided various robustness and sensitivity analyses, and expanded our discussion on systematic factors driving anxiety among ECRs (e.g., lack of permanent jobs for ECRs, high degrees of competitiveness, neo-liberalization of higher education, imposter syndrome, etc.).

R2.2

2. Study design and methods

(a) Search strategy & prereg deviations. The protocol lists “several databases,” but the final search relied on Scopus plus backward/forward chaining. Please provide a quantitative coverage check (e.g., precision tests against numbers from PsycINFO/MEDLINE/Web of Science) or replicate the search in at least one psychology/biomed database to mitigate single-database bias. If DOI overlap is higher than ~75%, report it and justify there's no need to conduct the entire search. Also, please ensure the deviation is mirrored in the Reporting Summary (not only Supplementary Table 26).

For the revised manuscript, two researchers (VG, DG) have now additionally screened ERIC, PubMed, and Web of Science. This new search yielded nine additional records, which were included in the analysis. Furthermore, a novel dataset from the Healthy Minds Network for 2024/2025 became available for students (PhD) and staff (postdoc), and was included as well. To document these changes, we have updated the main text and the PRISMA 2020 flow diagram in the revised manuscript All tables in the Supplementary Material that provide information on the study sample’s sociodemographic composition were updated as well. There remains a slight deviation from the preregistration (database SAGE and Google Scholar were not searched), which we elaborate on in Supplementary Table 32.

R2.3

(b) Inclusion criteria & grey literature. Including non-peer-reviewed records and multiple languages broadens coverage but does it increase heterogeneity to worrisome levels? Please consider adding sensitivity analyses excluding grey literature and machine-translated records, and report whether key pooled estimates meaningfully change, especially heterogeneity estimates. This is an aspect not as explored as it should IMHO.

In the revised manuscript, sensitivity analyses have been conducted for both the prevalence and severity models, concerning non-peer-reviewed records and machine-translated records. We provide detailed information in Supplementary Table 17. In case of the prevalence model, we found no significant differences between peer-reviewed and non-peer-reviewed records (-0.06%; 95% CI, -1.29 to 0.42, adjusted *p* = .686) and records coded in their original language (German, English, and French) and machine-translated records (-0.97%, 95% CI -14.08 – 0.42, adjusted *p*= .656). Similarly, we found no significant differences between peer-reviewed and non-peer-reviewed records (-4.36, 95% CI -9.13

- 0.40, adjusted *p* = .201) and records coded in their original language and machine-translated records (-8.79, 95% CI -25.70 - 8.11, adjusted *p* = .614) for the severity model, controlling for the FDR. We now also provide estimates of attributable true effect-size variance (R^2) throughout, both in the various tables and in-text. For the mentioned variables, R^2 values ranged from 0.11% to 53.47%.

R2.4

(c) Outcome harmonization & modeling. Building on the above, given substantial instrument heterogeneity, your threshold mappings and POMP transforms are appropriate but can be influential. Please add instrument-specific analyses and perhaps prevalence and severity (if this was done, I missed it, so please make sure to highlight it). As above, please comment more and report more on variance components (including level-2/level-3) for key models to illuminate where heterogeneity sits. Also clarify, expand, and explain interpretability.

We have added instrument-specific analyses for both the prevalence and severity models, concerning the PHQ-9 and GAD-7, contrasting summary estimates from these two scales with summary estimates of all other scales measuring depression and anxiety. This analysis showed that the estimated prevalence of depression was slightly higher in the PHQ-9, compared to other scales, and that the estimated severity of anxiety was slightly higher in GAD-7, compared to other scales. Still, both the outcome and the instrument continued to explain a large proportion of the variance in both models. Outcome explained 48.67% of the variance in the severity model and 21.24% in the prevalence model, whereas scale explained 53.47% in the severity model and 34.39% in the prevalence model. We elaborate on the heterogeneity attributable to the respective moderators in the various tables and in-text by providing information on R^2. We refrained from presenting heterogeneity estimates at all levels for all analyses, as this would have rendered the presentation of results cluttered and decreased their readability and comprehensibility.

R2.5

(d) Geography & cohort concentration. Several outcomes draw predominantly from US cohorts raising representativeness concerns. Look at the work of Sakshi Ghai. Please identify any overlapping cohorts, conduct leave-cohort-out sensitivity analyses, and report non-US-only estimates where possible.

We conducted additional analyses and found significant differences between US and non-US samples in the severity ($F[1, 534] = 3.31$, adjusted *p* = .031) and the prevalence model ($F[1, 350] = 3.83$, adjusted *p* =.015). Studies with non-US samples reported higher severity levels (by 4.00 POMP score points, 95% CI -0.32 - 8.32) and prevalence levels (by 0.66%, 95% CI -0.00 – 2.63) than studies with US samples. This information is now included and elaborated on in the main text and in Table 1. Further information can be found in Supplementary Table 17. Finally, we have expanded our limitations to point out the lack of diverse samples and the need to move beyond Western samples in research on psychological distress among ECRs, incorporating references on representativeness concerns (Ghai, 2021; Ghai et al., 2025).

> “Samples from the United States reported a lower prevalence of psychological distress (by 0.66%, 95% CI -0.00 – 2.63) and severity (by 4.00 POMP score points, 95% CI -0.32 - 8.32) than all other samples (R^2 = 5.1% and 2.5%, respectively).” (p. 11)

> “Future research should prioritize lesser studied outcomes and expand to geographically diverse samples, as the current evidence disproportionately draws on Western (especially US-based) samples, which may obscure variation in ECR mental health across academic systems and cultural contexts[96,97]. (p. 18)

Ghai, S. (2021). It’s time to reimagine sample diversity and retire the WEIRD dichotomy. *Nature Human Behaviour, 5*(8), 971–972. https://doi.org/10.1038/s41562-021-01175-9

Ghai, S., Thériault, R., Forscher, P., Shoda, Y., Syed, M., Puthillam, A., Peng, H. C., Basnight-Brown, D., Majid, A., Azevedo, F., & Singh, L. (2025). A manifesto for a globally diverse, equitable, and inclusive open science. *Communications Psychology, 3*(1), 16. https://doi.org/10.1038/s44271-024-00179-1

R2.6

(e) Country clustering. I am honestly not sure I understood about this section but in the Supplementary materials you seem to have AI-based clusters. Please explain Please justify this approach. Why is needed? Does it have to be AI? Why not dummy coded? Or take in some advice by Economists/Sociologists/PolSci? So unless there are very good reasons for it, and I am sure there may be, I would advise to move AI-derived clusters to exploratory status (unless externally validated). And please ensure AI use is declared in Methods and Reporting Summary.

We did not use dummy-coding for the countries, as this would have introduced an overly large number of predictors in the meta-regression model (the available data stemmed from 43 countries) and, more importantly, would have ignored the structural and systemic similarities between the countries we were interested in identifying and investigating in this analysis. We utilized AI for classification, because, currently, there is no published classification scheme available that could have been readily used in our study. Our reasoning was that AI should be able to draw on relevant information (being trained on large amounts of data) and provide us with a classification that could, at least, be used as a working model. Our original approach already tried to validate such a classification by using two LLMs, ChatGPT and DeepSeek, and combining their (already similar) results in a single classification scheme through discussion and consensus.

In the revised manuscript, we now present two further attempts at validating our original results (for details, see Supplementary Material 1, and Supplementary Tables 3–7). First, we created a categorization using GPT 5.1 by OpenAI, incorporating in the prompt this time explicitly the directive to use the OECD report *Benchmarking Higher Education System Performance* (2019) as a primary source. This resulted in a country grouping (Supplementary Table 6) that was broadly consistent with the clustering used in the original analysis (agreement rate 76%; Cohen $\kappa = .71$, $p < .001$). Major differences were that ChatGPT-5.1 identified a new cluster (southern European catch-up systems) with Greece, Italy, Spain, Portugal, and Ukraine (all formerly part of Cluster 3: self-funded or low-funded systems) and put all former Cluster-5 countries (unique or hybrid systems) into other pre-existing clusters (Cyprus and Iran into Cluster 3, Iceland into Cluster 2, and Algeria into Cluster 4). We interpreted this result as mostly confirming the original classification.

Second, we instructed Gemini 2 Flash by Google to create a third AI-based country clustering. This country clustering was again (only) based on the predefined criteria used for DeepSeek and ChatGPT. This third clustering (Supplementary Table 7) identified only 5 clusters, putting former Cluster-5 countries into Clusters 3 and 4, but was otherwise even slightly more consistent with the original classification than the ChatGPT-5.1 result (81% agreement rate of the Gemini-clustering with the original clustering; Cohen $\kappa = .77$, $p < .001$).

Both validation attempts, using other (versions of) LLMs than the original approach (and, in the first validation attempt, also a slightly altered prompt, augmenting the LLM with a source [the OECD report] that can be considered highly relevant to such a categorization), broadly confirmed our original classification, which is why we kept it in the revised manuscript We highlight in the main text that the country clustering was exploratory, as suggested by the reviewer, and note in the Limitations section that further research on optimal ways to classify countries is still needed. The use of AI was also declared in the Methods and Resulting Summary.

We hope that our validation of the AI-based country clustering allays this reviewer's concerns.

R2.7

3. Results and interpretation

(a) Prevalence vs severity narrative. The abstract conveys high prevalence while stating severities remain below clinical cutoffs. This can be misread as downplaying burden (which would be counter to your results' goals). Please reconcile these messages by clarifying instrument properties and explicitly distinguishing "case-identification thresholds" from "mean severity" signals. I would also be more clear on the abstract and try to compare the levels of prevalence between the general population to highlight prevalence/incidence and contextualize risk for ERCs.

We thank the reviewer for this important comment. We agree that in the abstract of the originally submitted manuscript, there was a mismatch in the narrative between prevalence and severity. We have added clarification to the abstract of the revised manuscript that prevalence was based on binary classification, whereas mean severity represented estimates from continuous scales. The reviewer raises an important point on prevalence rates in the general population to contextualize the findings on ECRs (see also our response to R1.7). As suggested, we have added prevalence rates for depressive and anxiety symptoms of age-matched samples in the general population to the abstract and the Introduction and Discussion sections. We have also removed the statement noting that severity estimates for eating disorders and suicidal ideation fell below clinical cutoffs, as a nuanced explanation would exceed the scope and word limit of the abstract. This clarification is now provided in the Discussion section and briefly repeated in the Conclusions.

In addition, self-administered mental health questionnaires in epidemiological studies are used to estimate the prevalence of symptoms rather than the prevalence of diagnosable mental disorders (e.g., depressive symptoms instead of depressive disorder; Zimmerman, 2024). We have revised the manuscript to clarify this distinction.

> "Prevalence of depressive symptoms was approximately two to three times higher, and anxiety symptoms three to five times higher than in age-matched general-population samples. Average symptom severity was mild for depressive and anxiety symptoms and moderate for stress." (p. 13)
>
> "Because prevalence is estimated as the proportion of individuals in a sample scoring above case-identification thresholds, whereas symptom severity reflects whole-sample mean scores on continuous scales, these two metrics capture distinct, but complementary, information about ECR mental health. The high prevalence of psychological distress is accompanied by, on average, generally mild symptom severity in the overall investigated samples, which reflects that mental-health issues are widespread rather than focused on subgroups with high symptom severity. In other words, even mild or subclinical symptom severity can indicate cause for concern and elevated future risk[64,69]. (p. 14)
>
> "These results indicate a widespread and systemic burden of psychological distress among ECRs, with mild overall symptom severity nonetheless signaling heightened risk for future mental problems." (p. 18)

R2.8

(b) Moderator interpretation & power. You conclude most individual-level moderators are null. Given power limitations and measurement heterogeneity, please soften the language ("no reliable evidence given current data") and include a table of marginal power across moderators (k, effective n, ICC). The small negative age effect (≈1% per decade) should be framed cautiously, too.

Information on the studies providing information per moderator, and the number of effect sizes within these studies, as well as the overall sample size, is provided in the Supplementary Tables 16 and 17. After including additional studies from searches in three additional databases (see also our response to R1.2), the small age effect was no longer significant. Instead, the effects of a history of mental health diagnoses and loneliness became significant in the prevalence model, but the effect sizes were very small, and we interpreted them as such.

R2.9

(c) Outcome coverage. You note thin evidence for sleep quality and other outcomes; consider flagging burnout and related constructs explicitly as gaps (many ECR surveys seldom burnout) to guide the field's data collection priorities.

We have expanded our list of outcomes. We now distinguish between outcomes for which evidence is drawn only from US samples, outcomes with limited overall evidence, and outcomes with some evidence, where additional data is still needed.

> "Third, for some syndromes and symptoms, the available evidence was drawn exclusively from studies conducted in the United States (e.g., eating disorders, non-suicidal self-injury, alcohol abuse), and for syndromes or symptoms, it was limited overall (e.g., sleep quality)." (p. 18)

R2.10

4. Presentation, clarity, and completeness

Please include a PRISMA flow diagram and place key extraction/threshold tables (now only in the Supplement) into the main text or Extended Data for accessibility. Label figures to distinguish prevalence vs severity panels MORE clearly; add footnotes noting when estimates are dominated by specific cohorts (e.g., Healthy Minds).

The PRISMA 2020 flow diagram was moved to the main text in the revised manuscript. Plain-language explanations were added to each figure. The Supplementary Material has been updated by an additional table that provides information on the effect size and sample size attributable to datasets stemming from the Healthy Minds Network, and the C19 ISWS dataset. We additionally provide information on the respective proportions attributable to the Healthy Minds Network datasets per syndrome and symptom for the prevalence model and per instrument used for the severity model below the figures. Figure labels have been updated.

R2.11

5. Data, code, and reproducibility

Include a README with session info and exact package versions, and archive a release (e.g., Zenodo DOI) at acceptance. A CSV with harmonized study-level variables (cutoffs, instruments, sample frame) would be highly useful. I will make the point again that I believe you are not doing enough work to 'sell' your work. Please improve your approach to scientific communication, this is such an important study, find ways to break information down for popular media and non-experts. Perhaps create a dashboard or website?

Datasets in both wide and long format, as well as the annotated analysis code, are available in the OSF repository. We will archive a Zenodo DOI of the dataset after acceptance. We have created and uploaded a ReadMe file with final session information and package versions to OSF.

We also thank the reviewer for their suggestion regarding scientific communication. We have discussed this point among the authors and have made several changes to improve the readability for non-expert audiences. In particular, we have removed abbreviations for general anxiety disorder and eating

disorder. We have also condensed the Results section on pooled prevalence and severity estimates to make it more readable and less technical. Finally, we have shortened the Discussion section by reducing details on study-level moderators, while expanding the section on systemic factors driving poor mental health among ECRs (lack of permanent jobs, metric-driven performance indicators, competitiveness, and additional burden for members from underrepresented groups).

R2.12

7. Minor points

Provide the variance explained by "scale type" vs "syndrome type" (R^2 analogues) and their implications.

Information on the explained variance by scale and syndrome type per model has been added in Table 1 and in the main text of the revised manuscript. The numbers are substantial (R2 of up to 51%) and indicate that the greatest heterogeneity is attributable to these two variables (what was investigated and how). (p. 15)

> "Notably, the type of measurement scale and the syndrome or symptom domain explained a substantial proportion of the variance (R^2 of up to 51%), indicating that what is measured and how it is measured strongly shapes prevalence and severity estimates"

R2.13

Clarify whether duplicate samples across multiple reports were deduplicated or modeled via three-level random intercepts; if not, please address.

Duplicate samples were excluded beforehand; to the best of the authors ' knowledge, no duplicate samples exist in the dataset used for analysis. We have aimed to clarify this in the manuscript (p. 22).

> "First, all screened articles were documented in Excel, and duplicates were identified by using the 'duplicate values' function. In case of duplicate records or records that used the same data, only one record was included."

R2.14

Maybe define POMP, arcsine or other transforms at first mention; add plain-language glosses in figure captions.

To improve readability for readers who begin with results and figures, we have added plain-language descriptions to each figure explaining how to interpret the displayed estimates. In addition, we have added a short plain-language definition of POMP scores at first mention and linked to the Methods section. Detailed definitions, rationales, and calculations for all transformations (including arcsine transformations) remain in the Methods section, where these Methods are first introduced.

> "Fig 2. Bars represent pooled prevalence estimates based on validated screening thresholds, with error bars indicating 95% confidence intervals." (p. 32)

> "Fig 3. Dots indicate pooled severity estimates for each scale, displayed on a normalized scale ranging from 0-100, to allow comparisons across measurement scales, with bars representing 95% confidence intervals. Colored bands indicate the severity categories derived from the original measurement scales. The pooled severity per measurement scale, including its 95% confidence interval, is shown on the right." (p. 32)

> "Fig 4. The line graph illustrates the pooled prevalences of psychological distress for publication years 2007 to 2025. The dark blue line represents the annual pooled prevalence and the shaded region is the 95% confidence band. The confidence band is wider prior to 2017

and for several later years, indicating greater uncertainty around pooled point estimates." (p. 33)

"Symptom severity across all outcomes was 33.9 (95% CI, 31.7–36.1; k = 151) on a normalized scale from 0–100 using percent of maximum possible (POMP) scores (see Methods for details)." (p. 8)

R2.15

8. General remarks:

I would love to see a bit of discussion on impostor syndrome and how it may or may not influence the results.

We thank the reviewer for this suggestion and have expanded our discussion of individual-level moderators. Our results indirectly reflect feelings related to the impostor phenomenon, as indicated by the subscale *personal accomplishment* of the burnout scale.

"For example, the impostor phenomenon (or impostor syndrome)[73], characterized by persistent self-doubt despite objective success, has been linked to increased stress and depressive symptoms in ECRs[58]. Impostor phenomena may also overlap with a reduced sense of personal accomplishment (a subscale of burnout), which was observed in this meta-analysis." (p. 15)

R2.16

I would also love to read more about what (systemic) factors you think lead to heightened levels of anxiety-related feelings in ECRs. Perhaps expanding/exploring on career advancement uncertainty, neo-liberalization and commodification of education and scientific production, hidden curriculum, lack of equitable authorship practices, lack of effective DEAI practices in academia, and so on. Here's a couple of papers from my work that might be useful but there's so much out there making much better arguments:

https://doi.org/10.1038/s41562-022-01508-2

https://doi.org/10.1038/s41562-023-01572-2

We agree that discussing the systemic factors driving stress and anxiety levels helps contextualize the results, and we have added a paragraph to address this point. We also condensed the Discussion section to make room for these additions. (p. 16)

"The modest associations of individual-level moderators with psychological distress suggest that the primary drivers of poor mental health among ECRs are structural rather than personal. The most frequently cited systemic factor is the mismatch between the growing number of PhD graduates and the comparatively limited number of permanent positions in academia[4,25–28]. As a result, most ECRs work on short-term or part-time contracts that involve career uncertainty, frequent relocations, and financial insecurity. At the same time, norms about research productivity have shifted toward metric-based performance indicators (e.g., h-index, citation count, external funding, publication output), which increasingly shape hiring, tenure, and funding decisions[29]. Critics argue that universities themselves are increasingly structured around market-oriented or neo-liberal principles[25,30], such as performance-based funding, external audits, and university rankings, which further reinforce metric-driven evaluations[31,32]. These shifts incentivize research that is safe, short-term, and likely to generate high citation counts, while discouraging riskier, long-term, or niche projects[4,28,33,34]. The result of these shifts in productivity norms, combined with the lack of permanent positions and stable career paths, is a climate of heightened competition[35] and a work culture that valorizes overwork (e.g.,

"publish or perish" narratives), characterized by blurred boundaries between work and leisure time, lack of breaks, and guilt about taking them[25,36,37]. Finally, for ECRs from underrepresented groups, additional barriers may exist, such as discrimination, limited mobility, and unequal access to mentorship and resources[25,38,39]. Together, these structural factors create substantial psychological strain and likely contribute to elevated levels of anxiety, stress, and sleep problems among ECRs."

Comments by Reviewer 3 (R3)

R3.0

The manuscript is generally well-written and offers a clear, comprehensive analysis of the topic. The authors effectively present their findings, and the structure of the paper is logical and easy to follow. However, placing the methods section at the end of the manuscript appears to be an oversight and should be reconsidered.

The study addresses a significant issue in academia and provides valuable insights into the mental health challenges faced by early-career researchers. Nonetheless, several areas could be improved to further strengthen the manuscript.

We thank the reviewer for their careful reading of our paper and for their thoughtful suggestions. We have addressed each of the reviewer's comments and provided our responses to each comment below. We appreciate the positive feedback on our writing and analytical approach. To comply with the journal's editorial policies, we had to place the methods at the end of the manuscript.

R3.1

Significance

This article presents a detailed and up-to-date meta-analysis of psychological distress among early-career researchers, addressing a broad range of mental health issues beyond depression and anxiety. It includes data from before, during, and after the COVID-19 pandemic, offering valuable insights into long-term trends and the pandemic's impact. The findings underscore the importance of study quality, sampling methods, and measurement tools in shaping results. The study highlights the urgent need for evidence-based interventions and systemic reforms to address mental health challenges in academia.

We thank the reviewer for their appreciation of our work and its significance for early-career researchers.

R3.2

Data and Methodology

The authors relied solely on Scopus for their literature search. Most meta-analyses in this area utilize multiple databases—such as PubMed, Web of Science, or ERIC—to ensure broader coverage. Limiting the search to a single database could result in missing relevant studies, particularly in certain disciplines. The authors should consider incorporating additional databases or provide a clear justification for using only Scopus.

We agree and have screened three additional databases, which has resulted in the inclusion of 9 additional records. This yielded 9 additional samples, which were included in the analysis. Furthermore, a novel dataset from the Healthy Minds Network for the years 2024/2025 became available for students (PhD) and staff (postdoc), which was included as well. We have updated the PRISMA 2020 flow diagram, as well as the main text and all relevant tables that can be found in the Supplementary Material, and provide information on the samples' sociodemographic composition.

With the inclusion of these new samples, estimates of pooled prevalence and severity remained similar, but minor changes were observed in the results of the moderator analyses. First, age no longer significantly moderated prevalence estimates. Second, both the history of mental disorders and loneliness emerged as significant moderators in the prevalence model, albeit with very small effect sizes.

R3.3

Additionally, the manuscript does not include the full search string or clarify its scope, which reduces transparency.

We agree and have adjusted the manuscript accordingly by including the full search string in the Methods section. The full search string for PubMed, ERIC, Web of Science and SCOPUS is also still available in Supplementary Table 33.

R3.4 x

There are also inconsistencies in the PRISMA flowchart: the transition from 794 records to 651 excluded and 139 included leaves four records unaccounted for.

The four additional records correspond to the primary databases from which we obtained raw data: the Healthy Minds Network (2024), the Cross-Country C19 ISWS dataset (Buffel & Velde, 2022), the Impact of COVID-19 on Doctoral and Early Career Researchers study (Byrom & Metcalfe, 2021), and the Nationwide Assessment of the Mental Health of UK Doctoral Researchers (Hazell et al., 2021). For transparency, we distinguished between published records included in the review and primary databases that provided raw data, which explains why these four items do not appear in the count of included papers. We hope this clarifies the misunderstanding. The reason for this apparent number misalignment is now also noted in the figure notes of the PRISMA 2020 flow diagram in the revised manuscript.

As part of this revision, we searched three additional databases (Web of Science, ERIC, and PubMed) and identified nine additional records/studies, all of which are now reflected in the updated PRISMA 2020 flow diagram. The total number of included studies, therefore, consists of 148 published records plus the four primary datasets listed above.

R3.5

In the results section, the cumulative sample size is reported as 128,684 participants (125,642 doctoral and 2,690 postdoctoral researchers). However, the remaining 352 participants are not explained and should be accounted for.

The remaining 352 participants are individuals we are certain are ECRs, but their pre- vs postdoctoral status was not reported. We amended the sentence in the results section (p. 7).

> "The final sample for meta-analysis consisted of 138,446 individuals (134,572 doctoral, 3,522 postdoctoral; pre- or postdoctoral status not reported for 352 ECRs)."

R3.6

The authors did not analyze the moderating effects of specific syndromes—such as depression or anxiety—separately, instead combining all symptoms into a single model. This approach may have diluted potential moderating effects. It is recommended that the authors conduct a separate analysis of all studies using a common instrument, such as the PHQ-9, which has a large sample size and standardized measurement. This could help clarify moderating effects more precisely.

We have included information on the separate moderating effects of PHQ-9 and GAD-7 per model in the main text of the revised manuscript. We found no significant differences between effect sizes

gathered via the PHQ-9, and effect sizes gathered via other scales for the severity model ($F[1, 534] = 0.13$, $p = .722$), yet for the prevalence model ($F[1, 350] = 13.09$, $p < .001$). We did further find differences between GAD-7 effect sizes and all other scales for the severity model ($F[1, 534] = 16.85$, $p < .001$), but not for the prevalence model ($F[1, 350] = 3.60$, $p = .058$). In case of the severity model, the usage of the GAD-7 increased the overall pooled severity of psychological distress by 7.45 POMP-score points (95 % CI, 3.88 - 11.02), whereas the increase in the overall pooled prevalence of psychological distress was marginal by 0.38% (95 % CI, 0.08 - 0.91). Attributable variance (R^2) of these two significant effects was rather small (2.7% and 1.7%).

R3.7

The results also indicate that severity variations are strongly influenced by the choice of measurement tool. Conducting separate analyses based on specific tools may provide deeper insights into moderating factors.

Please refer to our previous response.

R3.8

Clarity and Context

The discussion section offers general suggestions—such as expanding mental health services and reducing academic pressure—but these recommendations are neither novel nor accompanied by specific implementation strategies. The authors could strengthen the discussion by proposing more concrete, actionable interventions tailored to institutional and policy-level reforms.

We agree that specifying concrete, actionable steps and implementation strategies strengthens the discussion. We have therefore revised this section to make our recommendations more operational. In particular, we now specify examples of institutional support services that may benefit ECRs, as well as the content and formal requirements of supervisor training. We also clarify implementation pathways for career mentoring from institutions (e.g., structured individual development plans embedded in annual performance reviews). In addition, we now explicitly note the current scarcity of intervention research targeting mental health among ECRs and offer some examples for future research, drawing on evidence from the general population emerging studies in ECRs.

> "The present meta-analysis has several implications for research institutions and universities, funders and policymakers, supervisors, and ECRs themselves. Although this meta-analysis did not evaluate the effects of specific interventions, the modest associations of individual-level factors with psychological distress suggest that systemic factors likely play a substantial role and therefore require structural and institutional changes. For institutions, the findings underscore the need to improve working conditions. Institutions should expand access to support services (e.g., structured coaching programs, confidential ombudspersons) and dedicated mental health counseling, for example, through collaborations with existing medical or psychology departments. They could also mandate supervisor training focused on mentoring quality, expectation-setting, effective feedback, communication, and early recognition of distress as part of professional development requirements. Annual institutional- or faculty-level surveys could monitor mental health and evaluate the effects of implemented policies. Academic institutions should also provide career guidance[40] (e.g., structured individual development plans integrated into annual performance reviews) to prepare ECRs for careers inside and outside of academia. Policymakers and funders can address structural issues by increasing the number of permanent positions, promoting fairness and transparency in promotion criteria, and establishing safeguards against power abuse and discrimination, such as independent whistleblower channels. Nationwide surveys on mental health and academic working conditions could be established or expanded to monitor systemic progress

over time[2,41]. Supervisors can actively address mental health issues, participate in relevant skills training, and foster working environments in which employees feel safe to voice their concerns and discuss challenges[42]. Finally, for ECRs themselves, the findings can help normalize conversations about mental health issues, destigmatize seeking help, and encourage engagement with individual-focused interventions. At the same time, evidence on interventions targeting mental health in ECRs remains limited and is much needed. Potentially helpful interventions include stress management, cognitive behavior therapy[43], mindfulness- and compassion-based interventions[44–46], or social support interventions (e.g., peer support networks[47])." (p. 17)
